# Omnipolyphilins A and B: Chlorinated Cyclotetrapeptides and Naphtho-α-pyranones from the Plant Nematode-Derived Fungus *Polyphilus sieberi*

**DOI:** 10.1021/acs.jafc.4c00572

**Published:** 2024-03-20

**Authors:** Jan-Peer Wennrich, Sherif S. Ebada, Ellen Sepanian, Caren Holzenkamp, Syeda J. Khalid, Hedda Schrey, Wolfgang Maier, Attila Mándi, Tibor Kurtán, Samad Ashrafi, Marc Stadler

**Affiliations:** † Department of Microbial Drugs, Helmholtz Centre for Infection Research GmbH (HZI) and German Centre for Infection Research (DZIF), Inhoffenstraße 7, 38124 Braunschweig, Germany; ‡ Institute of Microbiology, Technische Universität Braunschweig, Spielmannstraße 7, 38106 Braunschweig, Germany; § Department of Pharmacognosy, Faculty of Pharmacy, Ain Shams University, 11566 Cairo, Egypt; ∥ Institute for Epidemiology and Pathogen Diagonstics, Julius Kühn Institut (JKI) - Federal Research Center for Cultivated Plants, Messeweg 11-12, 38104 Braunschweig, Germany; ⊥ Department of Organic Chemistry, University of Debrecen, P.O. Box 400, 4002 Debrecen, Hungary; # Institute for Crop and Soil Science, Julius Kühn Institute (JKI) − Federal Research Centre for Cultivated Plants, Bundesallee 58, 38116 Braunschweig, Germany

**Keywords:** helotiales, cyclotetrapeptides, naphthopyranones, antimicrobial, nematicidal

## Abstract

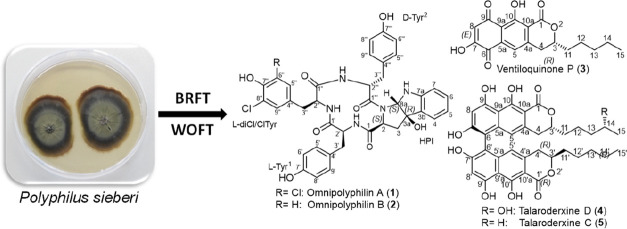

Chemical exploration for two isolates of the recently described ascomycete species *Polyphilussieberi*, derived from the eggs of the plant parasitic nematode *Heterodera filipjevi*, afforded the identification of many compounds that belong to various metabolite families: two previously undescribed chlorinated cyclotetrapeptides, omnipolyphilins A (**1**) and B (**2**), one new pyranonaphthoquinone, ventiloquinone P (**3**), a 6,6′-binaphto-α-pyranone dimer, talaroderxine D (**4**) in addition to nine known metabolites (**5**–**13**) were isolated from this biocontrol candidate. All isolated compounds were characterized by comprehensive 1D, 2D NMR, and HR-ESI-MS analyses. The absolute configurations of the cyclotetrapeptides were determined by a combination of advanced Marfey’s method, ROE correlation aided by conformational analysis, and TDDFT-ECD calculations, while ECD calculations, Mosher’s method, and experimental ECD spectra were used for ventiloquinone P (**3**) and talaroderxine D (**4**). Among the isolated compounds, talaroderxine D (**4**) showed potent antimicrobial activities against *Bacillus subtilis* and *Staphylococcus aureus* with MIC values of 2.1 and 8.3 μg mL^–1^, respectively. Additionally, promising inhibitory effects on talaroderxine D (**4**) against the formation of *S. aureus* biofilms were observed up to a concentration of 0.25 μg mL^–1^. Moreover, ophiocordylongiiside A (**10**) showed activity against the free-living nematode *Caenorhabditis elegans*.

## 1 Introduction

Plant parasitic nematodes (PPNs) are known to reduce the yield and health of their host plants, making them agricultural pathogens of global importance. The annual cost is estimated to be around 80 billion USD.^1^ Due to their complex biotrophic parasitism and multiple developmental stages, cyst-forming nematodes, such as *Heterodera* and *Globodera* spp., are among the most destructive plant parasitic nematodes.^2^ Based on their sedentary lifestyle, cyst nematodes are susceptible to fungal infections.^3^ Cyst parasitizing fungi were first described by Julius Kühn, who isolated *Catenaria auxiliaris*, first described as a *Tarichium* species, from the sugar beet cyst nematode *Heterodera schachtii*.^4^ Since then, a variety of different fungi associated with plant parasitic nematodes were found.^5^ They include but are not limited to the following: *Ijuhya vitellina*,^6^*Niesslia gamsii*,^7^*Polydomus karssenii*,^8,9^ and *Pochonia chlamydosporia*.^10^ Studies of the secondary metabolites of these fungi showed that they can be prolific producers of biologically active secondary metabolites. As they are about to be studied for their potential as biocontrol agents, they must be checked carefully for production of potentially detrimental toxins before further development, such as a scale-up of production, formulation, and registration. For instance, *Ijuhya vitellina* produces cytotoxic cytochalasans of the chaetoglobosin type and the oligopeptides named leucinostatins with nematicidal activity against *Caenorhabditis elegans*.^6,11^ Arthrichitins A and B produced by *P. karssenii* showed moderate activity against the host cyst nematode *Heterodera filipjevi*.^8,9^ The present study deals with the secondary metabolism of two further, hitherto unexplored strains of the recently described species *Polyphilus sieberi* that were isolated from eggs of the cereal cyst nematode *H. filipjevi* collected from wheat fields in Turkey.^12^ Herein, we describe the purification and structure elucidation up to the absolute configurations of two previously undescribed chlorinated cyclotetrapeptides (**1**–**2**), a new pyranonaphthoquinone (**3**) and a new dimeric 6,6′-binaphtho-α-pyranone derivative (**4**) together with nine known metabolites, namely, talaroderxine C (**5**),^13^ gliovirin-like compounds outovirins A (**6**) and C (**7**)^14^ along with trichodermamide C (**8**),^15^ peniciadametizine B (**9**),^16^ ophiocordylongiiside A (**10**),^17^ two fusidane triterpenes fusidic acid (**11**),^18,19^ 3-ketofusidic acid (**12**),^18,19^ and lumichromone (**13**)^20^ from different total extracts of the two nematode eggs-associated strains *P. sieberi* 17A and 17C. Selected compounds were subjected to antimicrobial, antibiofilm, cytotoxicity, and nematicidal activities, and herein we report their results.

## 2 Materials and Methods

### 2.1 General Experimental Procedures

Shimadzu UV–Vis spectrophotometer UV-2450 (Shimadzu, Kyoto, Japan) and a Jasco J-815 spectropolarimeter (JASCO, Pfungstadt, Germany) were used to measure UV and ECD spectra, respectively. Anton Paar MCP-150 polarimeter (Anton Paar Opto Tec GmbH, Seelze, Germany) was used to determine the optical rotation at 20 °C. Electrospray ionization mass spectra (ESI-MS) were measured with an UltiMate 3000 Series uHPLC (Thermo Fisher Scientific, Waltman, MA) using a C_18_ Acquity UPLC BEH column (50 × 2.1 mm, 1.7 μm, Waters, Milford, MA), solvent A: H_2_O + 0.1% formic acid; solvent B: acetonitrile (MeCN) + 0.1% formic acid, gradient: 5% B for 0.5 min increasing to 100% B in 19.5 min, maintaining 100% B for 5 min, flow rate 0.6 mL min^–1^, UV/Vis detection 190–600 nm) in combination with amaZon speed ESI Iontrap mass spectrometer (ESI-MS, amaZon, Bruker, Billerica, MA). High-resolution electrospray ionization mass spectra (HR-ESI-MS) were measured with a combination of an Agilent 1200 Infinity Series HPLC-UV system (Agilent Technologies, Santa Clara, CA) using identical column and LC parameters mentioned for ESI-MS measurements and a time-of-flight mass spectrometer (ESI-TOF-MS, maXis, Bruker, Billerica, MA), with the following parameters: scan range 100–2500 *m*/*z*, rate 2 Hz, capillary voltage 4500 V, dry temperature 200 °C. Compounds were dissolved in deuterated methanol-*d*_4_ or DMSO-*d*_6_ and NMR spectra were recorded on a Bruker Avance III 500 MHz spectrometer utilizing a BBGO (Plus) Smartprobe (^1^H: 500 MHz; ^13^C: 125 MHz) and a Bruker Avance III 700 MHz spectrometer utilizing a 5 mm TCI cryoprobe (^1^H: 700 MHz; ^13^C 175 MHz).

### 2.2 Fungal Material

The strains of *Polyphilus sieberi* 17A (DSM 106517) and 17C (JKI 73000) were isolated from the eggs of the plant parasitic nematode *Heterodera filipjevi*. Isolation procedure, morphological description, and multigene phylogeny were published by Ashrafi et al.^12^

### 2.3 Fermentation and Extraction

Strains were grown on YM6.3 agar (d-glucose 4 g L^–1^, malt extract 10 g L^–1^, yeast extract 4 g L^–1^, agar 20 g L^–1^ pH 6.3, before autoclaving) at 23 °C in the dark. Five mycelial plugs (0.5 × 0.5 cm) were transferred to a 500 mL Erlenmeyer flask containing 200 mL of Q6/2 media (d-glucose 2.5 g L^–1^, glycerol 10 g L^–1^, cottonseed flour 5 g L^–1^, pH 7.2) and incubated at 23 °C and 140 rpm^–1^. The culture broth was homogenized with an Ultra-Turrax (T25 easy clean digital, IKA), equipped with an S 25 N-25F dispersing tool, at 10,000 rpm for 10 s. This homogenized culture was used as an inoculum for YM6.3 (d-glucose 4 g L^–1^, malt extract 10 g L^–1^, yeast extract 4 g L^–1^, pH 6.3, autoclaved), ZM/2 (CaCO_3_ 1.5 g L^–1^, d-glucose 1.5 g L^–1^, lactalalbumin enzymatic hydrolysate (edamine) 0.5 g L^–1^, mannitol 4 g L^–1^, molasses 5 g L^–1^, (NH_4_)_2_SO_4_ 0.5 g L^–1^, oatmeal 5 g L^–1^, sucrose 4 g L^–1^, pH 7.2) in addition to BRFT and WOFT media (K_2_HPO_4_ 0.5 g L^–1^, sodium tartrate 0.5 g L^–1^, yeast extract 1 g L^–1^, 100 mL of solution mixed with 28 g brown rice or whole oat, autoclaved).

### 2.4 Solid-State Fermentation

Brown rice-based (BRFT) and whole oat-based (WOFT) solid-state fermentation was conducted as described before.^13^

### 2.5 Liquid Fermentation

For each of YM6.3 or ZM/2 media, twelve 1L Erlenmeyer flasks were used, each containing 400 mL of the respective medium, were inoculated each with 2 mL (0.5% of total volume) of the homogenized seed culture. That made in total a volume of 4.8 L fermentation broth per medium. Fermentation was stopped after 5 days of glucose depletion following the described protocol.^13^

### 2.6 Analytical HPLC

Extracts were dissolved in acetone:methanol (1:1) to achieve a concentration of 4.5 mg mL^–1^. A 2-μL injection was introduced into the ESI-MS system. A sample concentration of 1.0 mg mL^–1^ was used for HR-ESI-MS spectra.

### 2.7 Isolation of Compounds

*P. sieberi* 17C (JKI 73000) was cultivated on BRFT, WOFT, YM6.3, and ZM/2 culture media. Total methanol extracts of BRFT and WOFT (861 mg) were combined based on their ESI-MS profiles and purified with a FlashPure ID Silica 24 g cartridge on a Grace Reveleris X2 flash chromatography system, followed by several more purification steps with a Büchi Pure C-850 FlashPrep using a Gemini C_18_ (250 × 21.2/50 mm, 10 μm; Phenomenex, Aschaffenburg, Germany) and an Agilent Technologies 1200 Infinity Series semipreparative HPLC using a XBridge BEH C_18_ column (250 mm × 10 mm, 5 μm; Waters, Eschborn, Germany). The purification steps led to the isolation of compounds **1** (3.2 mg, *t*_R_ = 41.0 min), **2** (0.8 mg, *t*_R_ = 37.4 min), **3** (0.8 mg, *t*_R_ = 19.7 min), **4** (0.3 mg, *t*_R_ = 22.7 min), **5** (2.4 mg, *t*_R_ = 14.8 min), **6** (1.9 mg, *t*_R_ = 22.0 min), **10** (4.4 mg, *t*_R_ = 55.0 min), and **13** (0.7 mg, *t*_R_ = 14.8 min). A detailed description of the separation conditions is provided in the Supporting Information appendix (Figure S90, Tables S6–S10).

Supernatant extracts of YM6.3 (62 mg) were purified with a Büchi Pure C-850 FlashPrep using a Gemini C_18_ (250 mm × 21.2 mm, 10 μm; Phenomenex, Aschaffenburg, Germany), solvent A: H_2_O + 0.1% formic acid and solvent B: MeCN + 0.1% formic acid, flow rate: 20 mL min^–1^ affording compounds **11** (5.8 mg, *t*_R_ = 16.1 min) and **12** (0.2 mg, *t*_R_ = 14.3 min) by applying a gradient of 60% B for 5 min, increasing to 80% B in 30 min, raising to 100% B in 5 min, holding for 10 min at 100% B.

The extracts of *P. sieberi* 17A (DSM 106517) cultivated in BRFT and WOFT (724 mg) were combined and purified with FlashPure ID Silica 40 g cartridge using the same system and under the same condition as mentioned before. Fractions F5 (157 mg, *t*_R_= 22–32 min) and F6 (75 mg, *t*_R_= 32.3–33.8 min) were selected for further purifications on a Büchi Pure C-850 FlashPrep equipped with a Gemini C_18_ (250 mm × 21.2 mm, 10 μm; Phenomenex, Aschaffenburg, Germany). A detailed description of the separation conditions is provided in the Supporting Information appendix (Figure S91, Tables S11–S13). This purification yielded the known natural products **7** (0.7 mg, *t*_R_ = 36.9 min), **8** (25.2 mg, *t*_R_ = 28.5 min), and **9** (1.4 mg, *t*_R_ = 25.8 min).

### 2.8 Advanced Marfey’s Analysis of Compound **1**

For determining the absolute configuration of tyrosine and dichlorotyrosine residues in new chlorinated cyclotetrapeptide (**1**), Marfey’s method was used following the previously described protocol.^21^ The hydrolyzation of 0.5 mg of omnipolyphilin A (**1**) was performed under 90 °C with 500 μL 6 N HCl for 18 h. The acid was removed under vacuum, the residue was dissolved in 200 μL of H_2_O, mixed with 20 μL of 1 M NaHCO_3_, then 100 μL of acetone with 1% derivatization agent N_α_-(2,4-dinitro-5-fluorophenyl)-l-alaninamide (FDAA), N_α_-(2,4-dinitro-5-fluorphenyl)-l-valinamide (L-FDVA), or N_α_-(2,4-dinitro-5-fluorphenyl)-d-valinamide (D-FDVA) was added. This mixture was incubated for 40 min at 40 °C. Finally, the mixture was evaporated to dryness, dissolved in 1000 μL of MeOH, and analyzed with LC-ESI-MS implementing the same parameters mentioned above. Authentic standards of d-tyrosine, l-tyrosine, and 3,5-dichloro-l-tyrosine were treated like omnipolyphilin A (**1**). The absolute configuration of amino acids was determined by comparing retention times between hydrolysate of **1** and amino acid standards.

### 2.9 Preparation of (*R*)- and (*S*)-MTPA Ester Derivatives of **4**

An aliquot of compound **4** (0.4 mg) was dissolved in 220 μL of deuterated pyridine. Afterwards, 4 μL of (*R*)-(−)-α-methoxy-α-(trifluoromethyl)phenylacetyl chloride were added to the solution and incubated for 6 h at 23 °C. After conversion into the corresponding (*S*)-Mosher ester, the sample was transferred to a 3.0-mm NMR tube and subjected to ^1^H and ^1^H–^1^H COSY NMR measurements (Figures S92–S97). The same procedure was followed using the (*S*)-(+)-α-methoxy-α-(trifluoromethyl)phenylacetyl chloride to produce (*R*)-Mosher ester. The Δδ^SR^ values (Table S1) were calculated, and the configuration was assigned as previously described.^22^

### 2.10 Maintaining and Synchronization of *Caenorhabditis elegans* Population

*C. elegans* was kept on nematode growth media (NGM). For the preparation of NGM plates, the additional ingredients were added when cooled down to 55 °C after autoclaving as previously described.^23^ As a food source for nematodes, *Escherichia coli* plated on NGM media was used. For the synchronization of the nematode population, a time with high egg density (∼120 h) was chosen to bleach them. NGM plates were washed three times with 4 mL of 0.9% NaCl solution and transferred to a 15 mL conical centrifuge tube. The nematode suspension was centrifuged at 1100 g for 2 min and the supernatant was discarded. The procedure was repeated several times until a clear nematode suspension was obtained. After the last washing step, 3 mL of the suspension was kept and mixed with 3 mL of bleaching solution (1 mL of NaClO, 1 mL of 5 M NaOH, 6.25 mL of ultrapure H_2_O). Digestion of *C. elegans* was controlled under the microscope and stopped after disappearance of hatched nematodes. The bleaching was stopped by adding 0.9% NaCl solution, centrifuged, and washed as mentioned above. After the last washing step, the supernatant was removed and replaced by 7 mL of 0.9% NaCl solution. The tube was incubated at 23 °C on a rotary shaker with 80 rpm to hatch the eggs. The hatched nematodes were transferred to fresh NGM plates with *E. coli* lawn after 18 h. After 50–70 h, the J4 and adult *C. elegans* were washed from the plate, centrifuged, and washed as mentioned above. The amount of nematodes was determined and diluted to the desired concentration.

### 2.11 Biological Assays

Isolated natural products were assessed for their effects on different organisms and cell lines including Gram-positive, Gram-negative bacteria, filamentous fungi, yeasts, *Caenorhabditis elegans*, *Staphylococcus aureus* biofilm formation, and mammalian cell lines (Tables S2–S5). The effect on microbial growth was assessed by performing a serial dilution assay, which resulted in the determination of their minimum inhibitory concentration (MIC). A detailed description of the antimicrobial and cytotoxicity assays can be found in the Supporting Information. The cytotoxic effect of isolated compounds was tested against the cell lines KB3.1 (human endocervical adenocarcinoma), L929 (mouse fibroblasts), A431 (human epidermoid carcinoma), A549 (human lung carcinoma), PC-3 (human prostate adenocarcinoma), and MCF-7 (human breast adenocarcinoma). The nematicidal activity of the isolated compounds (Table S4) was evaluated against *C. elegans* in a 48-well flat-bottom plate. The methanol solutions of the tested compounds were filled in the wells, and subsequently, the solvent was evaporated and 300 μL of 0.9% NaCl solution with 1000 J4 and adult nematodes per mL was added per well. After 15 min, the wells were checked for immediate effects. Finally, the mortality rate was evaluated after 18 h of incubation on a plate shaker with 150 rpm at 23 °C. Biofilm inhibition activities of omnipolyphilin A (**1**) and talaroderxines C (**5**) and D (**4**) were evaluated against *Staphylococcus aureus* (DSM 1104) according to a previously described protocol.^24^ Briefly, a preculture of *S. aureus* (DSM 1104) was made from stock kept at −20 °C. After 18 h incubation, a media suspension for biofilm assay was made and OD_600_ of culture was adjusted to 0.001 McFarland standard. *S. aureus* was then incubated along with the serially diluted compounds (omnipolyphilin A (**1**): 125–0.9 μg mL^–1^; talaroderxine D (**4**): 33–0.001 μg mL^–1^; talaroderxine C (**5**): 125–0.003 μg mL^–1^) to produce biofilms in 96-well microtiter plates for 24 h. The biofilm inhibition activity of compounds **1**, **4**, and **5** was assessed by crystal violet staining with subsequent washing steps and analyzing the stained biomass with a microtiter plate reader (Synergy 2, BioTek). Methanol was used as a solvent control and taken as 100% of no inhibition against biofilm formation of *S. aureus*. Microporenic acid A (MAA) was used as a positive control.^24,25^ All experiments were performed twice with duplicates. Differences between samples and control group were determined by two-tailed Student’s *t*-test. Statistical significance was defined as *p* < 0.05. Analysis was carried out using Excel 2016 (Microsoft).

### 2.12 Computational Section

Mixed torsional/low-mode conformational searches were carried out by means of the Macromodel 10.8.011 software using the MMFF with an implicit solvent model for CHCl_3_ applying a 21 kJ mol^–1^ energy window.^26^ Geometry reoptimizations of the resultant conformers [ωB97X/TZVP with the PCM solvent model for MeCN or MeOH] and TDDFT-ECD calculations were performed with Gaussian 16.^27^ For TDDFT-ECD, various functionals (B3LYP, BH&HLYP, CAM-B3LYP, PBE0) and the TZVP basis set were used with the same solvent model as in the preceding DFT optimization step. The sTDA spectra were calculated with the CAM-B3LYP, LC-BLYP, and ωB97X functionals and the sTDA 1.6 package.^28^ ECD spectra were generated as the sum of Gaussians with 2400–2700 cm^–1^ half-height widths, using dipole-velocity computed rotational strengths.^29^ Boltzmann distributions were estimated from the ωB97X energies. The MOLEKEL program was used for visualization of the results.^30^

## 3 Results and Discussion

**Figure 1:**
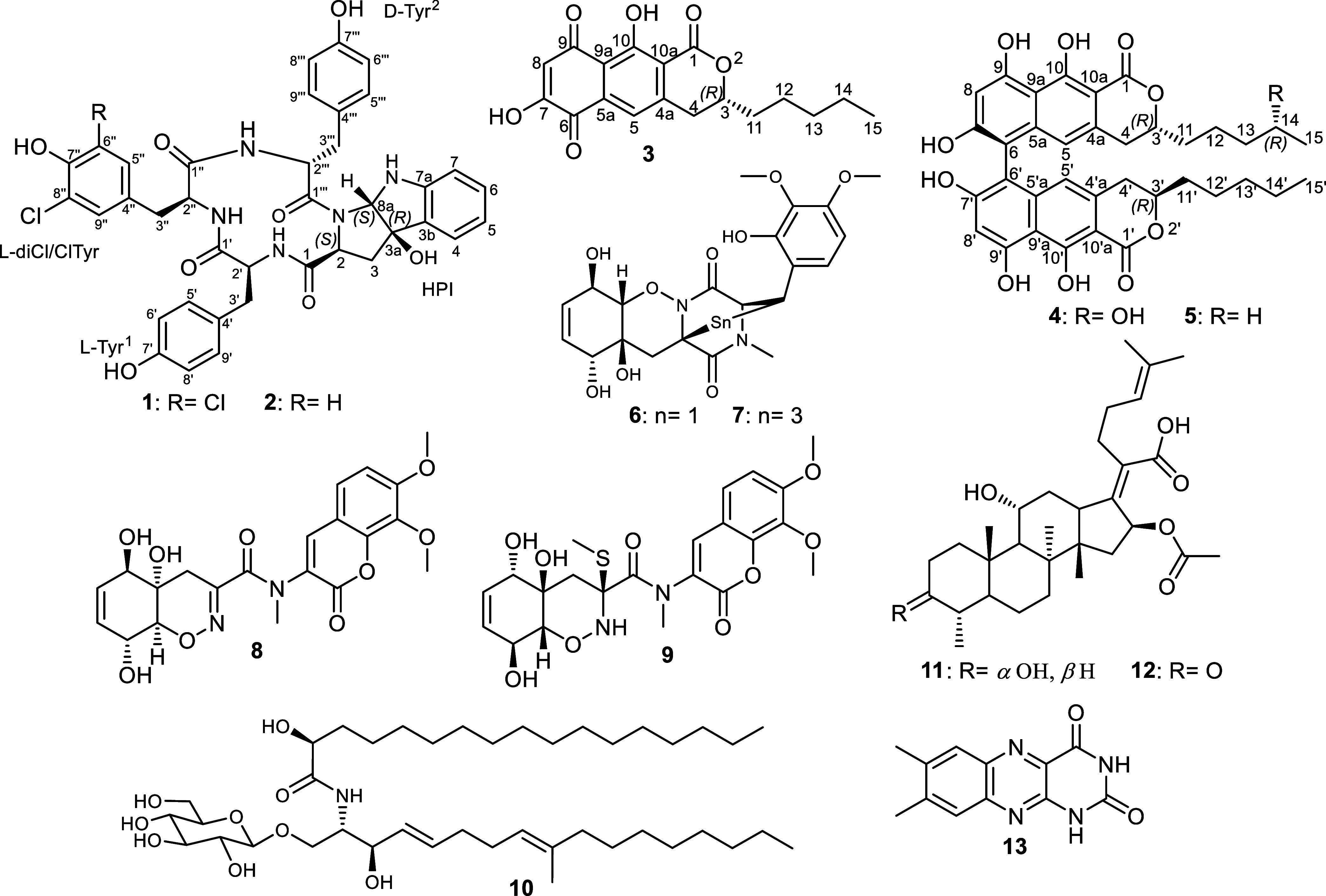
Chemical structures of **1**–**13**.

**Figure 2:**
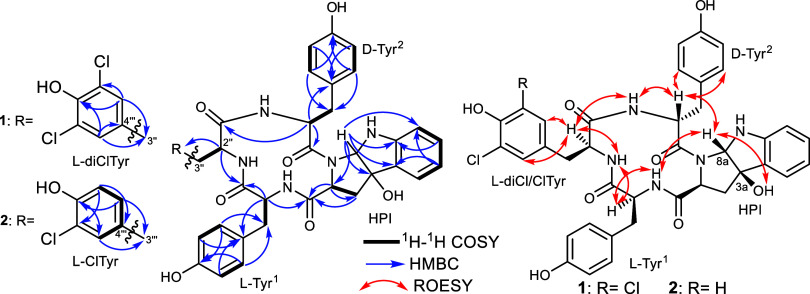
Key ^1^H–^1^H COSY, HMBC, and ROESY correlations of **1** and **2**.

Compound **1** was isolated as a pale yellow amorphous solid that revealed in its LR-ESI-MS spectrum a characteristic pattern of halogenated compound with a pseudomolecular ion peak cluster of *m*/*z* 760.22, 762.20, and 764.21 [M + H]^+^ in relative abundance of 9:6:1, suggesting the compound to incorporate two chlorine atoms in its structure. The molecular formula of **1** was established to be C_38_H_35_Cl_2_N_5_O_8_ based on HR-ESI-MS that exhibited a protonated molecule peak at *m*/*z* 760.1930 [M + H]^+^ (calculated 760.1935) and a sodium adduct at *m*/*z* 782.1749 [M + Na]^+^ (calculated 782.1755) indicating the presence of twenty-three degrees of unsaturation. The 1D (^1^H and ^13^C) NMR spectra of **1** (Table 1) suggested **1** to be a peptide based on the presence of four exchangeable NH protons (δ_H_ 6.98–7.38), four amino acid α-protons (δ_H_ 4.19–5.02, H-2 ∼ H-2‴), four diastereotopic β-methylene proton pairs at δ_H_ 2.37/2.54 (H_2_-3), δ_H_ 2.82/2.93 (H_2_-3‴), δ_H_ 2.84/2.93 (H_2_-3′), and δ_H_ 2.94/3.05 (H_2_-3″) ppm. In addition, ^1^H NMR and ^1^H–^1^H COSY spectra (Figure 2) revealed the presence of two 1,4-disubstituted aromatic rings each featuring two-doublet proton signals each with an integration index of two at δ_H_ 6.73/7.10 (*J =* 8.5 Hz, 2H-6‴,8‴/2H-5‴,9‴) for the first aromatic ring and at δ_H_ 6.63/6.91 (*J =* 8.5 Hz, 2H-6′,8′/2H-5′,9′) for the second one indicating the presence of two tyrosine residues in the peptide. The aromatic region also revealed a singlet-proton resonance with an integration index of two at δ_H_ 7.17 (2H-5″,9″), suggesting its inclusion in 1,3,4,5*-*tetrasubstituted aromatic ring. Alongside, the aromatic region in the ^1^H NMR and ^1^H–^1^H COSY spectra (Figure 2) of **1** also revealed a 1,2-disubstituted aromatic ring through a spin system that extended over four aromatic protons at δ_H_ 7.25 (d, *J =* 7.5 Hz, H-4), δ_H_ 6.81 (t, *J =* 7.5 Hz, H-5), δ_H_ 7.12 (t, *J =* 7.5 Hz, H-6), and δ_H_ 6.47 (d, *J =* 7.5 Hz, H-7). Based on the obtained results, compound **1** was suggested to include two tyrosine amino acid residues (Tyr^1^/Tyr^2^) together with a third one that presumably incorporates two identical substituents at 6″,8″-positions on its aromatic ring imparting a deshielding effect on their carbon atoms that appeared at δ_C_ 130.0 instead of δ_C_ 116.0 ppm in Tyr^1^ and Tyr^2^. These findings suggested that the third tyrosine moiety is probably seen as 6,8-dichlorotyrosine residue (diClTyr). In addition, the ^13^C NMR spectrum of **1** disclosed further unassigned carbon resonances together with the insufficient degrees of unsaturation (15 from three Tyr residues) provided some clues for the existence of another highly conjugated functionality in **1** that comprises the 1,2-disubstituted aromatic ring. To elucidate its structure, the HMBC spectrum of **1** was measured revealing key correlations that helped together with the ^1^H–^1^H COSY spectrum (Figure 2) to elucidate 3a-hydroxypyrroloindolinone (HPI) moiety fulfilling the lacking seven degrees of unsaturation and characterizing **1** as a cyclotetrapeptide comprising two tyrosines (Tyr^1^ and Tyr^2^), one dichlorotyrosine (diClTyr), and 3a-hydroxypyrroloindolinone (HPI) residues. The HMBC spectrum of **1** (Figure 2) has also defined the amino acid sequence through the key correlations from the α-proton of Tyr^1^ at δ_H_ 4.43 (dd, *J =* 9.8, 7.0 Hz, H-2′) to the adjacent carbonyl carbon of HPI at δ_C_ 174.3 (C-1) and to its own carbonyl carbon (δ_C_ 175.0, C-1′). The α-proton of dichlorotyrosine moiety at δ_H_ 4.54 (dd, *J =* 13.7, 7.6 Hz, H-2″) in turn revealed key correlations to its own carbonyl carbon (δ_C_ 173.6, C-1″) in common with the second tyrosine amino acid α-proton at δ_H_ 5.02 (dd, *J =* 8.8, 6.6 Hz, H-2‴). Finally, H-2‴ showed key correlations in the HMBC spectrum to its own carbonyl carbon at δ_C_ 175.4 (C-1‴). These key correlations suggested the cyclotetrapeptide to be HPI-Tyr^1^-diClTyr-Tyr^2^, as depicted in Figure 1. Further confirmation for the depicted amino acid arrangement in **1** was provided by ROESY spectral analysis (Figure 2) that unveiled key ROE correlations from NH protons of the amino acid residues, Tyr^1^ at δ_H_ 7.07 (d, *J =* 8.3 Hz) and diClTyr at δ_H_ 7.38 (d, *J =* 9.6 Hz) to H-2″ that in turn revealed key ROE correlations with NH proton of Tyr^2^ amino acid residue at δ_H_ 7.14 (d, *J =* 8.4 Hz). The H-8a in HPI at δ_H_ 5.85 (s) revealed key ROE correlations to H-2‴ of Tyr^2^ at δ_H_ 5.02 (dd, *J =* 8.8, 6.6 Hz) and the OH-3a proton at δ_H_ 6.31 (br s) indicating cofacial orientation of H-2‴, H-8a and OH-3a. As a conclusion, the obtained results indicated the amino acid sequence in **1** to be HPI-Tyr^1^-diClTyr-Tyr^2^. A literature search on cyclic peptides featuring the HPI moiety revealed the plant-derived cyclopentapeptide melicoptelines D and E comprising HPI residues with (2*S*,3a*S*,8a*R*) and (2*S*,3a*R*,8a*S*) absolute configurations, respectively, that were recently reported as potent antiviral derivatives against influenza A virus.^31^ The HPI moiety can be only produced with *cis* ring junction to afford either (3a*R*,8a*S*) or (3a*S*,8a*R*) absolute configuration.^32^ By comparing ^1^H and ^13^C NMR data of HPI in **1** to those reported for melicoptelines D and E,^31^ it could be deduced that HPI moiety in **1** adopts the *cis*-(3aR*,8aS*) relative configuration. The H-8a/OH-3a ROE correlation of **1** observed in DMSO-*d*_6_ (Figures 2 and S8a) confirmed their *cis* orientation, while ROE correlation of H-8a and the NH proton of the C-2 carboxamide group suggested the (2S*,3aR*,8aS*) relative configuration. In addition, four stereoisomers of the HPI residue including the (2*S*,3a*R*,8a*S*) and (2*S*,3a*S*,8a*R*) ones were previously synthesized and their chemical shifts of H-2 and H-8a revealed clear differences appearing at δ_H_ 3.86 (q, *J* = 12.0, 7.0 Hz) and 5.40 (s) rather than at δ_H_ 4.34 (t, *J* = 7.0 Hz) and 5.31 (s), respectively.^33^ Reported ECD spectra of the four synthetic stereoisomeric HPI revealed that the configuration of the chiral carbon atoms C-3a and C-8a of the *cis*-annulated 2,3-dihydro-1*H*-indole governs the ECD spectrum.^33^ Stereoisomers of HPI with (3a*R*,8a*S*) absolute configuration showed two negative Cotton effects (CEs) at about 300 and 240 nm deriving from the π–π* transitions of the substituted benzene chromophore. The ECD spectra of compound **1** in acetonitrile and methanol (Figure S9) also showed two negative transitions in that region, on the basis of which (3a*R*,8a*S*) absolute configuration was assigned to the HPI residue. Considering the known relative configuration, this allowed determining (2*S*,3a*R*,8a*S*) absolute configuration for HPI residue in **1**, implying that it was derived from L-tryptophan amino acid. The absolute configuration of α-carbon atoms for each amino acid was unambiguously determined using the advanced Marfey’s method (see Supporting Information Figures S83 and S84).^21^ Based on the comparison of retention times between authentic amino acid standards and the hydrolyte of **1**, the results disclosed that the dichlorotyrosine residue had L-configuration and there were one D- and one l-tyrosine amino acid residues that could be distinguished on the basis of ROE correlations between the α-proton of Tyr^2^ and H-8a suggesting (l-Tyr^1^,d-Tyr^2^) assignment. In order to confirm the proposed positions of l- and d-tyrosine, the characteristic ROE correlation between H-8a and H-2‴ of Tyr^2^ was checked on the computed low-energy MMFF and DFT-optimized conformers of (l-Tyr^1^,d-Tyr^2^)-**1**, in which the two protons were found to be on the same face and their interatomic distance was 2.20 Å (Figure S98a). Moreover, the low-energy MMFF conformers of the diastereomeric (d-Tyr^1^,l-Tyr^2^)-**1** were also computed, in which the H-8a and H-2‴ of Tyr^2^ are facing the opposite direction with a distance of 4.12 Å (Figure S98b), which would not enable the characteristic ROE correlation. In order to confirm further the configurational assignment of the HPI, l-Tyr^1^, and d-Tyr^2^ residues, TDDFT-ECD spectrum of **1** was computed with different methods. Since the initial conformational search resulted in a large number of conformer clusters in the applied 21 kJ mol^–1^ energy window, the 959 MMFF conformers of **1** (Figure S99) were optimized first at the B3LYP/6-31G(d) PCM/MeCN level, reclustered and the resulting 219 conformers were reoptimized at the ωB97X/TZVP PCM/MeCN level. In the 13 low-energy ωB97X/TZVP PCM/MeCN conformers, the characteristic H-8a and H-2‴ of Tyr^2^ had the same orientation as those in the lowest-energy MMFF conformer (Figure S99).

**Table 1:** ^1^H and ^13^C NMR Data of **1** and **2**

	**1**		**2**	
Pos.	δ_C_,^a^^,^^d^ type	δ_H_^b^ (multi, *J*[Hz])	δ_C_,^d^ type	δ_H_^c^ (multi, *J*[Hz])
HPI				
1	174.3, CO		174.4, CO	
2	63.7, CH	4.19 dd (8.7, 6.2)	63.9, CH	4.17 dd (8.8, 6.4)
3	40.3, CH_2_	α 2.37 dd (13.7, 6.2)	40.5, CH_2_	α 2.39 dd (13.8, 6.4)
		β 2.54 dd (13.7, 8.7)		β 2.56 dd (13.8, 8.8)
3a	89.2, C		89.3, C	
3b	131.9, C		131.8, C	
4	123.6, CH	7.25 d (7.5)	123.8, CH	7.25 d (7.1)
5	120.8, CH	6.81 t (7.5)	121.0, CH	6.81 t (7.1)
6	130.7, CH	7.12 t (7.5)	131.0, CH	7.12 t (7.1)
7	112.2, CH	6.47 d (7.5)	112.4, CH	6.47 d (7.1)
7a	149.4, C		149.4, C	
8-NH		6.98 d (8.1)^e^		
8a	85.2, CH	5.85 s	85.3, CH	5.85 s
3a–OH		6.31 br s^e^		
Tyr^1^				
1′	175.0, CO		175.0, CO	
2′	59.2, CH	4.43 dd (9.8, 7.0)	59.4, CH	4.43 dd (9.6, 7.0)
3′	36.6, CH_2_	α 2.84 dd (13.6, 7.0)	36.7, CH_2_	α 2.83 dd (14.3, 7.0)
		β 2.93 dd (13.6, 9.8)		β 2.93 dd (14.3, 9.6)
4′	128.2, C		128.2, C	
5′,9′	130.8, CH	6.91 d (8.5, 2H)	131.0, CH	6.91 d (8.5, 2H)
6′,8′	116.0, CH	6.63 d (8.5, 2H)	116.3, CH	6.63 d (8.5, 2H)
7′	157.2, C		157.2, C	
NH		7.07 d (8.3)^e^		
DiClTyr/ClTyr				
1″	173.6, CO		174.0, CO	
2″	55.7, CH	4.54 dd (13.7, 7.6)	56.1, CH	4.55 dd (15.5, 7.7)
3″	34.0, CH_2_	α 2.94 dd (14.4, 8.7)	34.3, CH_2_	α 2.94 dd (14.4, 8.7)
		β 3.05 dd (14.4, 7.4)		β 3.05 dd (14.4, 7.4)
4″	123.3, C		121.6, C	
5″	130.2, CH	7.17 s	129.5, CH	6.96 dd (8.3, 2.4)
6″	130.0, C		117.7, CH	6.81 d (8.3)
7″	149.6, C		153.3, C	
8″	130.0, C		130.3, C	
9″	130.2, CH	7.17 s	131.5, CH	7.20 d (2.4)
NH		7.38 d (9.6)		
Tyr^2^				
1‴	175.4, CO		175.5, CO	
2‴	54.2, CH	5.02 dd (8.8, 6.6)	54.5, CH	5.01 dd (8.8, 7.0)
3‴	36.1, CH_2_	α 2.82 dd (13.4, 6.6)	36.4, CH_2_	α 2.83 dd (13.9, 7.0)
		β 2.93 dd (13.4, 8.8)		β 3.05 dd (13.8, 2.8)
4‴	128.9, C		128.9, C	
5‴,9‴	131.4, CH	7.10 d (8.5, 2H)	131.6, CH	7.10 d (8.5, 2H)
6‴,8‴	116.3, CH	6.73 d (8.5, 2H)	116.4, CH	6.74 d (8.5, 2H)
7‴	157.6, C		157.5. C	
NH		7.14 d (8.4)^e^		

a Measured in methanol-*d*_4_: ^*a*^ At 125 MHz.

b At 500 MHz.

c At 700 MHz.

d Assignment confirmed by HMBC and HSQC spectra.

e Measured in DMSO-*d_6_* at 500 MHz.

**Figure 3:**
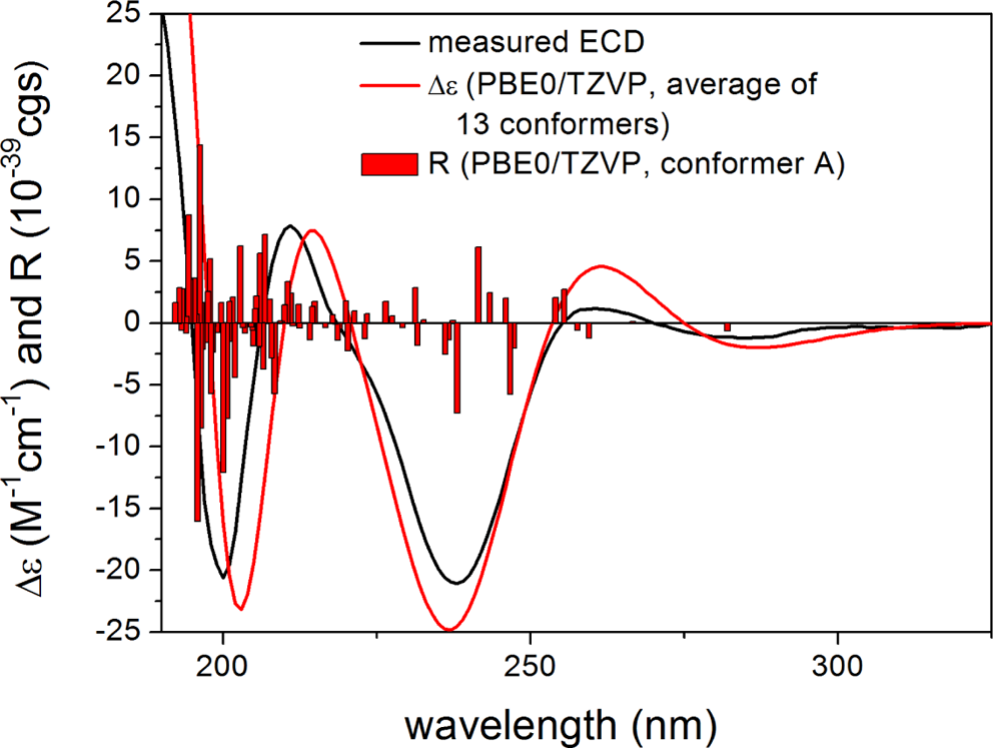
Experimental ECD spectrum of **1** (black) compared with the PBE0/TZVP PCM/MeCN ECD spectrum of (l-Tyr^1^,d-Tyr^2^)-**1** (red). Level of DFT optimization: ωB97X/TZVP PCM/MeCN. Bars represent the computed rotational strengths of the lowest-energy conformer.

TDDFT-ECD^34^ and sTDA-ECD^35^ calculations were performed on the 13 low-energy (≥1% Boltzmann population) conformers, which gave good agreements with the experimental ECD spectrum allowing verification of the absolute configuration (Figures 3 and S100). Since there are only a few examples of sTDA calculations on natural products,^36^ this may serve as a further example of the successful application of a natural cyclic peptide derivative. Based on the obtained results, compound **1** was unambiguously confirmed to represent a previously undescribed dichlorinated cyclotetrapeptide with the amino acid sequence of l-Tyr^1^-L-diClTyr-d-Tyr^2^-HPI, and it was given a trivial name omnipolyphilin A.

Compound **2** was obtained as a colorless amorphous solid that revealed a characteristic pattern of a halogenated compound in its LR-ESI-MS with a pseudomolecular ion peak cluster of *m*/*z* 726.28 and 728.24 [M + H]^+^ in relative abundance of 3:1 suggesting the compound to possess one chlorine atom in its structure that explains its smaller molecular weight by 35 atomic units compared to **1**. The HR-ESI-MS spectrum of **2** confirmed this notion by establishing its molecular formula as C_38_H_36_ClN_5_O_8_ based on its pseudomolecular ion peaks at *m*/*z* 726.2310 [M + H]^+^ (calculated 726.2325) and *m*/*z* 748.2133 [M + Na]^+^ (calculated 748.2145) indicating the existence of twenty-three degrees of unsaturation equal to those in **1**. The 1D (^1^H and ^13^C) NMR data (Table 1) and HSQC spectrum (Figure S15) of **2** revealed a close similarity to **1** with one major difference is the presence of an additional aromatic proton on the chlorinated tyrosine residue and hence turned it into 1,3,4-trisubstituted aromatic ring bearing three protons at δ_H_ 6.81 (d, *J =* 8.3 Hz, H-6″; δ_C_ 117.7), δ_H_ 6.96 (dd, *J =* 8.3, 2.4 Hz, H-5″; δ_C_ 129.5), and δ_H_ 7.20 (d, *J =* 2.4 Hz, H-9″; δ_C_ 131.5). Apart from that difference, compound **2** revealed a close resemblance to **1** in both its 1D (^1^H/^13^C NMR) and 2D (^1^H–^1^H COSY, HMBC, HSQC, and ROESY) spectral data (Table 1, Figure 2) that suggested compound **2** to comprise three amino acids recognized into two tyrosines (Tyr^1^/Tyr^2^) and one chlorotyrosine (ClTyr) along with an HPI moiety. The ROESY spectrum of **2** (Figure 2) exhibited comparable key ROE correlations to those in **1** in particular those from H-2‴ of Tyr^2^ at δ_H_ 5.01 (dd, *J =* 8.8, 7.0 Hz) to H-8a at δ_H_ 5.85 (s) in HPI and the latter revealed no ROE correlation to H-2 at δ_H_ 4.17 (dd, *J =* 8.8, 6.4 Hz), hence suggesting the same (2*S*,3a*R*,8a*S*) absolute configuration for the HPI residue as in **1** and hence to be similarly derived from L-tryptophan amino acid.^31−33^ Based on the close coherence between **1** and **2** in their depicted structures, spectral data, and biosynthetic origin, the absolute configuration of the three amino acids in **2** were concluded to be identical to those in **1** namely, l-Tyr^1^-L-ClTyr-d-Tyr^2^-HPI, and it was identified as a previously undescribed monochlorinated cyclotetrapeptide that was trivially named as omnipolyphilin B.

**Figure 4:**
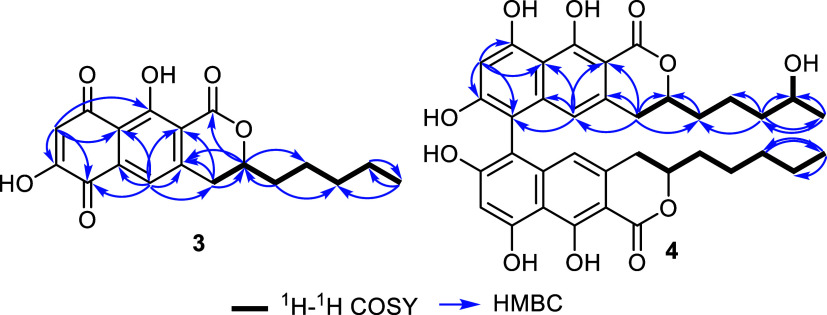
Key ^1^H–^1^H COSY and HMBC correlations of **3** and **4**.

Compound **3** was purified as an orange solid powder whose molecular formula was determined to be C_18_H_18_O_6_ based on the HR-ESI-MS spectrum that revealed a pseudomolecular ion peak at *m*/*z* 331.1173 [M + H]^+^ (calculated 331.1176) and a sodium adduct at *m*/*z* 353.0993 [M + Na]^+^ (calculated 353.0996) indicating the presence of ten degrees of unsaturation. The ^13^C NMR and HSQC spectra of **3** (Table 2) revealed the presence of 18 different carbon resonances that can be differentiated into nine quaternary carbon atoms including three carbonyl carbons (δ_C_ 190.0, 181.0, and 167.9), two oxygenated olefinic carbon atoms (δ_C_ 161.8 and 160.7) and four olefinic carbon atoms (δ_C_ 148.4, 133.6, 117.8, and 114.0) in addition to three tertiary carbon atoms (δ_C_ 116.3, 110.3, and 77.3), five secondary carbon atoms (δ_C_ 33.8, 33.6, 31.0, 24.0, and 22.0), and one primary carbon (δ_C_ 13.9). The ^1^H NMR, ^1^H–^1^H COSY, and HSQC spectra of **3** (Table 2, Figure 4) revealed the presence an extended spin system beginning at two diastereotopic methylene protons (H_2_-4) at δ_H_ 2.96 (dd, *J* = 16.8, 11.4 Hz) and δ_H_ 3.17 (dd, *J =* 16.8, 2.8 Hz) both directly correlated to a secondary sp^3^ carbon at δ_C_ 33.6 (C-4) then extends to an oxygenated aliphatic methine at δ_H_ 4.49 (m, H-3; δ_C_ 77.3) and continues over four methylene groups at δ_H_ 1.65/1.72 (H_2_-11; δ_C_ 33.8), δ_H_ 1.38/1.45 (H_2_-12; δ_C_ 24.0), δ_H_ 1.30 (m, H_2_-13; δ_C_ 31.0), and δ_H_ 1.30 (m, H_2_-14; δ_C_ 22.0) ending by a terminal triplet methyl group at δ_H_ 0.88 (t, *J =* 6.7 Hz, H_3_-15; δ_C_ 13.9) indicating the presence of *n*-pentyl aliphatic side chain as a substituent on a 6-membered lactone ring. According to the obtained results, compound **3** was deduced to have a naphthoquinone moiety in its structure supported by the key HMBC correlations (Figure 4) from aromatic protons H-5 at δ_H_ 7.42 (s; δ_C_ 116.3) and H-8 at δ_H_ 6.06 (d, *J =* 3.3 Hz; δ_C_ 110.3) to two naphthoquinone carbonyl at δ_C_ 190.0 (C-9) and δ_C_ 181.0 (C-6). By searching the reported literature, compound **3** was found to have naphtho[2,3-*c*]pyran-6,9-qunione nucleus as its core structure related to ventiloquinones F-G, that were previously reported as plant metabolites derived from the root bark of *Ventilago maderaspatana*.^37−39^

**Table 2:** ^1^H and ^13^C NMR Data of **3** and **4**

**3**			**4**		
pos.	δ_C_,^a^^,^^c^ type	δ_H_^b^ (multi, *J*[Hz])	Pos.	δ_C_,^a^^,^^c^ type	δ_H_^b^ (multi, *J*[Hz])
1	167.9, CO		1/1′	170.7, CO	
3	77.3, CH	4.49 m	3/3′	79.5, CH	4.50 dtd (11.2, 7.6, 4.2)
4	33.6, CH_2_	α 2.96 dd (16.8, 11.4)	4/4′	32.4, CH_2_	α 2.73 dd (16.5, 11.1)
		β 3.17 dd (16.8, 2.8)			β 2.81 dd (16.5, 3.5)
4a	148.4, C		4*a*/4a′	133.5, C	
5	116.3, CH	7.42 s	5/5′	112.9, CH	6.21 s
5a	133.6, C		5*a*/5a′	139.9, C	
6	181.0, CO		6/6′	107.7, C	
7	161.8, C		7/7′	157.7, C	
8	110.3, CH	6.06 d (3.3)	8/8′	101.7, CH	6.58 s
9	190.0, CO		9/9′	158.9, C	
9a	114.0, C		9*a*/9a′	107.5, C	
10	160.7, C		10/10′	162.9, C	
10a	117.8, C		10*a*/10a′	98.7, C	
11	33.8, CH_2_	α 1.65 m	11	34.2, CH_2_	α 1.56 m
		β 1.72 m			β 1.68 m
			11′	34.0, CH_2_	α 1.56 m
					β 1.68 m
12	24.0, CH_2_	α 1.38 m	12	20.6, CH_2_	1.24 m
		β 1.45 m	12′	23.8, CH_2_	1.39 m
13	31.0, CH_2_	1.30 m	13	38.6, CH_2_	α 1.29 m
					β 1.33 m
			13′	31.0, CH_2_	1.25 m
14	22.0, CH_2_	1.30 m	14	65.5, CH	3.54 m
			14′	21.9, CH_2_	1.25 m
15	13.9, CH_3_	0.88 t (6.7)	15	23.6, CH_3_	1.00 d (6.1)
			15′	13.8, CH_3_	0.84 t (7.0)
7-OH			7/7′–OH		10.00 br s
9-OH			9/9′–OH		9.65 s
10-OH		13.87 br s	10/10′–OH		13.46 br s
			14-OH		4.31 d (4.7)

a Measured in DMSO-*d*_6_ at 175 MHz

b at 700 MHz.

c Assignment confirmed by HMBC and HSQC spectra.

Further confirmation of the suggested structure of compound **3** (Figure 1) was provided by its HMBC spectral results (Figure 4) that revealed key correlations from H-3 to C-4a (δ_C_ 148.4) and C-12 (δ_C_ 24.0) that confirmed the *n*-pentyl to be present as a substituent at C-3 on an α-pyranone ring that is fused to a naphthoquinone moiety.

Compound **3**, containing a hydroxylated 1,4-naphthoquinone subunit, is the oxidized analogue of the related semitalaroderxine C,^13^ which has a trihydroxynaphthalene moiety. Based on the structural similarity and the common biosynthetic origin between **3** and (*R*)-semitalaroderxine C, recently reported from different strains of the genus *Polyphilus*, the absolute configuration of C-3 was suggested to be (*R*).^13^ The absolute configuration of **3** was determined to be (*R*) based on the negative and positive CEs observed in the ECD spectrum (Figure S85) at 301 and 266 nm, which were in accordance with those revealed by the structurally related derivative, (*R*)-semixanthomegnin at 305 and 272 nm, respectively.^40^

**Figure 5:**
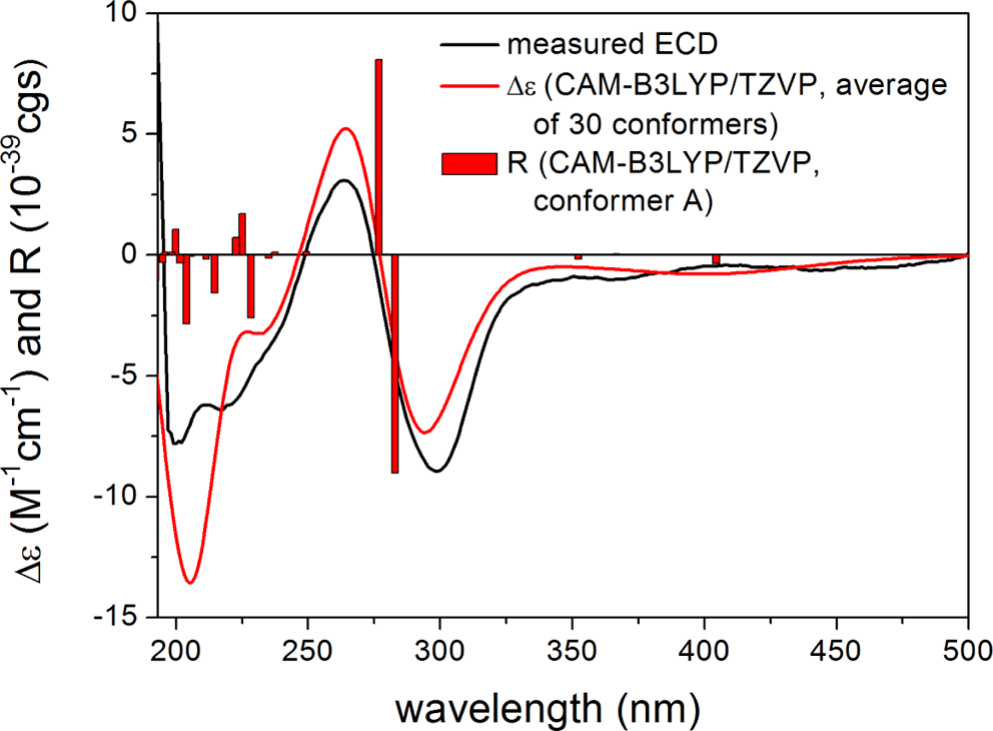
Experimental ECD spectrum of **3** (black) compared with the CAM-B3LYP/TZVP PCM/MeOH ECD spectrum of (*R*)-**3** (red). Level of DFT optimization: ωB97X/TZVP PCM/MeOH.

In order to confirm the configurational assignment independently, TDDFT-ECD calculations were performed on the 30 low-energy ωB97X/TZVP PCM/MeOH conformers of (*R*)-**3** (Figure S101) obtained by the reoptimization of the 205 initial MMFF conformers. The Boltzmann-weighted CAM-B3LYP/TZVP PCM/MeOH ECD spectrum of (*R*)-**3** gave a good agreement with the experimental ECD spectrum of **3** (Figure 5) confirming our assignment. Based on the obtained results, compound **3** was determined to be a new natural compound, and it was named ventiloquinone P.

Compound **4** was isolated as a yellow solid powder with a molecular formula of C_36_H_38_O_11_, determined via its HR-ESI-MS spectrum that revealed pseudomolecular ion peaks at *m*/*z* 647.2486 [M + H]^+^ (calculated 647.2487) and *m*/*z* 669.2308 [M + Na]^+^ (calculated 669.2306) indicating its possession of eighteen degrees of unsaturation. The ^1^H and ^13^C NMR data of **4** in DMSO-*d*_6_ (Table 2) revealed a notably lower number of resonances in both spectra compared to those established in its molecular formula that suggested the probability to be of a dimeric structure. However, its ^1^H–^1^H COSY and HSQC spectra (Figure 4) undoubtedly confirmed the existence of two different aliphatic side chains, one is elucidated as *n*-pentyl resembling that present in **3** extending over four methylene groups at δ_H_ 1.56/1.68 (H_2_-11′; δ_C_ 34.0), δ_H_ 1.39 (H_2_-12′; δ_C_ 23.8), δ_H_ 1.25 (m, H_2_-13′; δ_C_ 31.0) and δ_H_ 1.25 (m, H_2_-14′; δ_C_ 21.9) ending by terminal triplet methyl group at δ_H_ 0.84 (t, *J =* 7.0, H_3_-15′; δ_C_ 13.8). The other aliphatic side chain in the ^1^H–^1^H COSY spectrum revealed an extended spin system over four methylene groups at δ_H_ 1.56/1.68 (H_2_-11; δ_C_ 34.2), δ_H_ 1.24 (H_2_-12; δ_C_ 20.6), δ_H_ 1.29/1.33 (H_2_-13; δ_C_ 38.6), and δ_H_ 3.54 (m, H-14; δ_C_ 65.5) ending by a terminal doublet methyl group at δ_H_ 1.00 (d, *J =* 6.1, H_3_-15; δ_C_ 13.8). Apart from the two side chains, ^1^H, ^13^C NMR and HSQC spectral data of **4** (Table 2) revealed a single set of proton peaks each representing an integration index of two including two aromatic proton singlets, at δ_H_ 6.21 (s, H-5/5′; δ_C_ 112.9) and at δ_H_ 6.58 (s, H-8/8′; δ_C_ 101.7) together with one aliphatic methine at δ_H_ 4.50 (dtd, *J =* 11.2, 7.6, 4.2 Hz, H-3/3′; δ_C_ 79.5) that revealed spin–spin coupling to two pairs of diastereotopic methylene protons at δ_H_ 2.73 (dd, *J =* 16.5, 11.1 Hz, Hα-4/4′)/δ_H_ 2.81 (dd, *J =* 16.5, 3.5 Hz, Hβ-4/4′) directly correlated to a secondary sp3 carbon at δ_C_ 32.4 and at δ_H_ 1.56/δ_H_ 1.68 (m, H_2_-11/11′; δ_C_ 34.0/34.2).

By comparing the spectral data of **4** and the reported literature, it was obvious to be structurally comprising 6,6′-binaphtho-α-pyranone skeleton similar to that found in talaroderxines A-C,^13,41^ pigmentosins,^24,42^ and asteromine.^43^ The closest similarity in spectral data was found between **4** and talaroderxine C (**5**)^13^ where the only difference was the presence of a *sec*-hydroxyl group at C-14 in **4** rather than being a methylene group in **5**.

**Figure 6:**
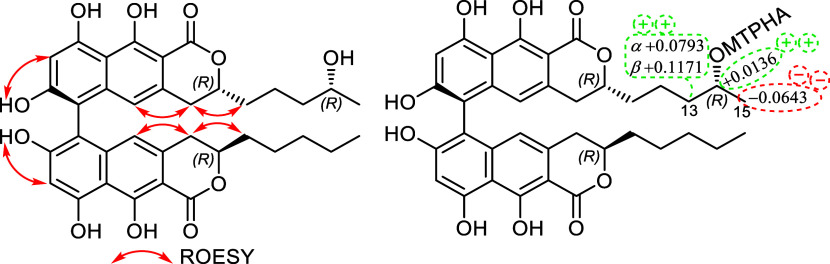
Key ROESY correlations and the Δδ^*SR*^ values of (*S*)/(*R*) MTPA esters obtained from talaroderixne D (**4**) diagnostic for (*aS*,3*R*,14*R*,3′*R*).

The 6,6′-linked biaryl-type heterodimer **4** had three isolated blocks of chirality, which could not be correlated by experimental NMR methods: (i) axial chirality due to the stereogenic *ortho*-tetrasubstituted 6,6′-biaryl axis, (ii) C-3 chirality centers of the naphthopyrone subunits, and (iii) C-14 chirality center of the flexible 4-hydroxypent-1-yl side chain. The ECD spectra of biaryl natural products containing both axial and central chirality elements are dominantly governed by the axial chirality element.^44−47^ The sign and magnitude of the biaryl dihedral angle of the two aryl units in axially chiral biaryls are reflected in the interaction of the two aromatic chromophores, giving rise to exciton-coupled ECD bands. The signs of the exciton-coupled ECD couplets (Figure S86) are characteristic of the axial chirality as exemplified by the related 6,6′-linked *bis*-naphthopyrones pigmentosins A and B^24^ and talaroderxine A.^41^ Due to the intense positive exciton couplet of **4** centered at 260 nm, the axial chirality was determined as (a*S*). On the basis of common biosynthetic origin with the related monomeric units semitalaroderxine C^13^ and ventiloquinone P, (3*R*,3′*R*) absolute configuration was assigned to the naphthopyrone subunits. The absolute configuration of the secondary 14-OH group of the side chain was established by implementing the Mosher’s method, which implied the synthesis of (*S*)- and (*R*)-MTPA esters using (*R*)- and (*S*)-MTPA acid chloride reagents, respectively.^22^ The measured consistent chemical shift differences (Figure 6, Table S1) exhibited positive Δδ^*SR*^ values for H_2_-13, whereas negative one was recorded for H_3_-15 that determined the absolute configuration of **4** as (14*R*). Based on the obtained results, compound **4** was identified as a new 6,6′-binaphtho-α-pyranone derivative, (14*R*)-OH-talaroderxine C, that was given a trivial name talaroderxine D.

Based on the isolated amounts, compounds **1**, **3**-**5**, **8**, **10**, and **11** were evaluated for their cytotoxic activity against a panel of six different cell lines (Table S2), antimicrobial and nematicidal activities against twelve microorganisms consisting of Gram-positive, Gram-negative bacteria, fungi (Table S3), and *C. elegans* (Table S4), respectively. Additionally, omnipolyphilin A (**1**) and talaroderxines D (**4**) and C (**5**) were evaluated for their inhibitory effects on *S. aureus* biofilm formation.

In the cytotoxicity (MTT) assay, talaroderxines D (**4**) and C (**5**) disclosed potent cytotoxic activities in particular against MCF-7 and A431 (Table 3) with IC_50_ values ranging between 0.068 and 2.38 μM. According to the antimicrobial assay results (Table 4), fusidic acid (**11**) showed weak activity against *Mycobacterium smegmatis* while significant effects were observed against *B. subtilis* (MIC = 1.04 μg mL^–1^) and *S. aureus* (MIC = 8.3 μg mL^–1^). Comparing talaroderxine D (**4**) and talaroderxine C (**5**),^13^ the former revealed less potent antimicrobial activity against *B. subtilis* (MIC = 2.1 μg mL^–1^) but significantly potent antimicrobial effect against *S. aureus* (MIC = 8.3 μg mL^–1^) (Table 4).

**Table 3:** Cytotoxicity (IC_50_) Assay of **4**, **5**, and **11^*a*^**

cytotoxicity assay	IC_50_ (μM)			positive control
tested cell line	**4**	**5**	**11**	epothilone B (nM)
L929 (murine)	n.a	1.19	n.a.	0.65
KB3.1 (cervical adenocarcinoma)	2.01	10.32	n.a.	0.17
PC-3 (prostatic adenocarcinoma)	9.75	8.73	n.d.	0.09
MCF-7 (breast adenocarcinoma)	1.16	0.068	n.d.	0.07
A431 (epidermoid carcinoma)	1.19	2.38	n.d.	0.06
A549 (lung adenocarcinoma)	26.3	2.06	n.d.	0.05

**Table 4:** Antimicrobial (MIC) Assay of **4**, **5**, and **11^a^**

	MIC (μg mL^–1^)			positive control
tested microorganism	**4**	**5**	**11**	antibiotic (μg mL^–1^)
*Staphylococcus aureus*	8.3	66.6	8.3	0.21^G^
*Bacillus subtilis*	2.1	0.52	1.04	16.6°
*Mycobacterium smegmatis*	n.i	n.i	66.6	1.70^K^

a n.a.: No activity. n.i.: No inhibition up to 67 μg mL^–1^. n.d.: Not determined; G: Gentamycin; O: Oxytetracycline; K: Kanamycin.

Furthermore, talaroderxines C (**5**) and D (**4**) significantly inhibited the formation of *S. aureus* biofilms up to 70% by concentrations of 3.9 μg mL^–1^ and 0.25 μg mL^–1^, respectively. However, omnipolyphilin A (**1**) was slightly active against *S. aureus* biofilms with significant inhibitory effects of 30% at a concentration of 62.5 μg mL^–1^ compared to microporenic acid that inhibited biofilm formation by >50% at 7.8 μg mL^–1^. In nematicidal activity assay, only ophiocordylongiiside A (**10**) showed significant effects on *C. elegans* with a mortality rate of 69% at 100 μg mL^–1^. No significant effect against tested cell lines or microorganisms was observed for compounds **3**, **8**, and **10**.

In the course of our ongoing search for anti-infectives, this study introduces a follow-up exploration of secondary metabolites from the fungal genus *Polyphilus*. In conclusion, thirteen metabolites were isolated from *P. sieberi* including two new cyclotetrapeptides, omnipolyphilins A (**1**) and B (**2**), a naphthoquinopyranone, ventiloquinone P (**3**), two naptho-α-pyranone dimers, talaroderxines D (**4**) and C (**5**),^13^ together with other known metabolites, outovirins A (**6**) and C (**7**),^14^ trichodermamide C (**8**),^15^ peniciadametizine B (**9**),^16^ ophiocordylongiiside A (**10**),^17^ two fusidane triterpenes, fusidic acid (**11**),^18,19^ 3-ketofusidic acid (**12**),^18,19^ and lumichromone (**13**).^20^ The isolated talaroderxines C (**5**) and D (**4**) exhibited significant inhibitory effects on the formation of *S. aureus* biofilms far below their MIC values (Table S5, Figures S88-S89). The same properties were also described for the structure-related pigmentosins A and B.^24^ In comparison to the latter, talaroderxines C (**5**) and D (**4**) exhibited not only pronounced effects on *S. aureus* biofilm formation but also stronger cytotoxicity on the tested cell lines. However, talaroderxine D (**4**) showed a 7-fold higher biofilm inhibitory effect compared to its highest cytotoxicity. Future studies may attempt to reduce the cytotoxicity of these natural products while retaining or increasing their potency against biofilms. Ophiocordylongiiside A (**10**) was active against *C. elegans* and should also be tested in the future against plant pathogenic nematodes to establish a possible ecological role. Furthermore, the effects of *P. sieberi* on the actual infestation of *H. filipjevi* in agricultural crops could be evaluated in greenhouse experiments. Within these investigations, studies of the production of additional mycotoxins in the natural environment as well as their ecological impact should be conducted. For this purpose, it might even be feasible to use modern metabolomics techniques as recently employed for a strain of the basidiomycete *Armillaria.*(48)*Polyphilus* spp., however, should perhaps not be further pursued with priority for biocontrol purposes because several of the metabolites we have encountered may be potentially harmful for beneficial organisms, and even the production of the marketed antibiotic drugs of the fusidic acid type, which were finally identified as major antibacterial principles, may pose a risk that can lead to antimicrobial resistance against the antibiotic among soil bacteria.

On the other hand, this study also shows the huge potential of the genus *Polyphilus* to produce a variety of different natural product classes.

Omnipolyphilin A (**1**): pale yellow amorphous solid; [α]_D_^25^ −87.0 (*c* 0.1, MeOH); UV (MeOH) λ_max_ (log ε): 283.5 (0.1), 203.0 (1.4); ECD (*c* = 1.64 × 10^–4^ M; MeCN) λ [nm], (Δε) 285 (−1.18), 260 (1.18), 238 (−21.35), 211 (8.94), 200 (−23.18). ECD (*c* = 1.64 × 10^–4^ M; MeOH) λ [nm], (Δε) 290 (−1.28), 263 (0.30), 237 (−24.05), 210 (7.06), 199 (−27.03). ECD (*c* = 1.64 × 10^–4^ M; MeCN) λ [nm], (Δε) 305sh (−0.36), 285 (−1.21), 260 (1.16), 238 (−21.06), 211 (7.88), 200 (−20.61); NMR data (^1^H NMR: 500 MHz, ^13^C NMR: 125 MHz in methanol-*d*_4_) see Table 1; LR-ESI-MS: *m*/*z* 760.22 [M + H]^+^, *m*/*z* 758.30 [M – H]^−^; HR-ESI-MS: *m*/*z* 760.1930 [M + H]^+^ (calcd. 760.1935 for C_38_H_36_Cl_2_N_5_O_8_^+^), *m*/*z* 782.1749 [M + Na]^+^ (calcd. 782.1755 for C_38_H_35_Cl_2_N_5_NaO_8_^+^); *t*_R_ = 7.85 min (HR-ESI-MS).

Omnipolyphilin B (**2**): colorless amorphous solid; [α]_D_^25^ −135.0 (*c* 0.1, MeOH); UV (MeOH) λ_max_ (log ε): 279.0 (0.1), 199.5 (1.4); NMR data (^1^H NMR: 700 MHz in methanol-*d*_4_) see Table 1; LR-ESI-MS: *m*/*z* 726.28 [M + H]^+^, *m*/*z* 724.32 [M – H]^−^; HR-ESI-MS: *m*/*z* 726.2310 [M + H]^+^ (calcd. 726.2325 for C_38_H_37_ClN_5_O_8_^+^), *m*/*z* 748.2133 [M + Na]^+^ (calcd. 748.2145 for C_38_H_36_ClN_5_NaO_8_^+^); *t*_R_ = 7.35 min (HR-ESI-MS).

Ventiloquinone P (**3**): orange solid powder; [α]_D_^25^ −440.0 (*c* 0.02, MeOH); UV (MeOH) λ_max_ (log ε): 495.0 (0.1), 382.5 (0.2), 275.5 (1.1), 233.0 (0.9), 215.0 (1.3); ECD (*c* = 1.51 × 10^–4^ M; MeOH) λ [nm], (Δε) 443br (−0.81), 369sh (−1.11), 299 (−9.23), 266 (3.45), 221sh (−6.64), 198 (−9.48); NMR data (^1^H NMR: 700 MHz, ^13^C NMR: 175 MHz in DMSO-*d*_6_) see Table 2; LR-ESI-MS: *m*/*z* 331.16 [M + H]^+^, *m*/*z* 683.23 [2M+Na]^+^, *m*/*z* 328.89 [M – H]^−^; HR-ESI-MS: *m*/*z* 331.1173 [M + H]^+^ (calcd. 331.1176 for C_18_H_19_O_6_^+^), *m*/*z* 353.0993 [M + Na]^+^ (calcd. 353.0996 for C_18_H_18_NaO_6_^+^), *m*/*z* 683.2098 [2M+Na]^+^ (calcd. 683.2099 for C_36_H_36_NaO_12_^+^); *t*_R_ = 11.12 min (HR-ESI-MS).

Talaroderxine D (**4**): yellow solid powder; [α]_D_^25^ +181.0 (*c* 0.1, MeOH); UV (MeOH) λ_max_ (log ε): 374.5 (0.7), 261.5 (2.4), 224.5 (1.1), 195.0 (0.9); ECD (*c* = 1.54 × 10^–4^ M; MeOH) λ [nm], (Δε) 386 (2.51), 320 (−4.19), 268 (100.83), 252 (−92.73); NMR data (^1^H NMR: 700 MHz, ^13^C NMR: 175 MHz in DMSO-*d*_6_) see Table 2; LR-ESI-MS: *m*/*z* 647.32 [M + H]^+^, *m*/*z* 645.16 [M – H]^−^; HR-ESI-MS: *m*/*z* 647.2486 [M + H]^+^ (calcd. 647.2487 for C_36_H_39_O_11_^+^), *m*/*z* 669.2308 [M + Na]^+^ (calcd. 669.2306 for C_36_H_38_NaO_11_^+^), *m*/*z* 1293.4897 [2M+H]^+^ (calcd. 1293.4901 for C_72_H_77_O_22_^+^); *t*_R_ = 12.55 min (HR-ESI-MS).

## Supporting Information Available

1D (^1^H/^13^C) and 2D (^1^H–^1^H COSY, HMBC, HSQC, and ROESY) spectra in addition to all HPLC-ESI-MS and HR-ESIMS of **1**-**13** (PDF)
