# Supplementary material for: Omnipolyphilins A and B: Chlorinated Cyclotetrapeptides and Naphtho-α-pyranones from the Plant Nematode-Derived Fungus Polyphilus sieberi
Source: J Agric Food Chem. 2024 Mar 20;72(13):6998–7009. doi: 10.1021/acs.jafc.4c00572 (PMC10995996; doi:10.1021/acs.jafc.4c00572)
Supplement: Supplementary file 1 — jf4c00572_si_001.pdf [file jf4c00572_si_001.pdf]

Supporting Information for

Omnipolyphilins A and B: chlorinated cyclotetrapeptides and naphtho-*α*-pyranones from the plant nematode-derived fungus *Polyphilus sieberi*

Jan-Peer Wennrich,[a,b] Sherif S. Ebada,[a,c*] Ellen Sepanian,[a] Caren Holzenkamp,[a,b] Syeda J. Khalid,[a,b] Hedda Schrey,[a,b] Wolfgang Maier,[d] Attila Mándi,[e] Tibor Kurtán,[e] Samad Ashrafi,[d,f], and Marc Stadler[a,b*]

[a] Department of Microbial Drugs, Helmholtz Centre for Infection Research GmbH (HZI) and German Centre for Infection Research (DZIF), Inhoffenstraße 7, 38124 Braunschweig, Germany

[b] Institute of Microbiology, Technische Universität Braunschweig, Spielmannstraße 7, 38106 Braunschweig, Germany

[c] Department of Pharmacognosy, Faculty of Pharmacy, Ain Shams University, 11566 Cairo, Egypt

[d] Institute for Epidemiology and Pathogen Diagonstics, Julius Kühn Institut (JKI) - Federal Research Center for Cultivated Plants, Messeweg 11-12, 38104 Braunschweig, Germany

[e] Department of Organic Chemistry, University of Debrecen, P. O. Box 400, 4002 Debrecen, Hungary

[f] Institute for Crop and Soil Science, Julius Kühn Institute (JKI) – Federal Research Centre for Cultivated Plants, Bundesallee 58, 38116 Braunschweig, Germany

*Correspondence: sherif.elsayed@helmholtz-hzi.de; sherif_elsayed@pharma.asu.edu.eg (S.S.E.); Marc.Stadler@helmholtz-hzi.de (M.S.); Tel.: +49-531-6181-424

Contents of Supporting Information

#	Contents	Page
**1**	Figure S1. LRESIMS of **1**.	**S4**
**2**	Figure S2. HRESIMS of **1**.	**S5**
**3**	Figure S3. ^{1}H NMR spectrum of **1** in methanol-d_4 at 500 MHz.	**S6**
**4**	Figure S4. ^{13}C NMR spectrum of **1** in methanol-d_4 at 125 MHz.	**S7**
**5**	Figure S5. ^{1}H-^{1}H COSY spectrum of **1** in methanol-d_4 at 500 MHz.	**S8**
**6**	Figure S6. HMBC spectrum of **1** in methanol-d_4 at 500 MHz.	**S9**
**7**	Figure S7. HSQC spectrum of **1** in methanol-d_4 at 500 MHz.	**S10**
**8**	Figure S8. ROESY spectrum of **1** in methanol-d_4 at 500 MHz.	**S11**
**9**	Figure S8a. ROESY spectrum of **1** in DMSO-d_4 at 500 MHz.	**S12**
**10**	Figure S9. Experimental ECD spectra of **1** in acetonitrile (blue) and methanol (green).	**S13**
**11**	Figure S10. LRESIMS of **2**.	**S14**
**12**	Figure S11. HRESIMS of **2**.	**S15**
**13**	Figure S12. ^{1}H NMR spectrum of **2** in methanol-d_4 at 700 MHz.	**S16**
**14**	Figure S13. ^{1}H-^{1}H COSY spectrum of **2** in methanol-d_4 at 700 MHz.	**S17**
**15**	Figure S14. HMBC spectrum of **2** in methanol-d_4 at 700 MHz.	**S18**
**16**	Figure S15. HSQC spectrum of **2** in methanol-d_4 at 700 MHz.	**S19**
**17**	Figure S16. ROESY spectrum of **2** in methanol-d_4 at 700 MHz.	**S20**
**18**	Figure S17. LRESIMS of **3**.	**S21**
**19**	Figure S18. HRESIMS of **3**.	**S22**
**20**	Figure S19. ^{1}H NMR spectrum of **3** in DMSO-d_6 at 700 MHz.	**S23**
**21**	Figure S20. ^{13}C NMR spectrum of **3** in DMSO-d_6 at 170 MHz.	**S24**
**22**	Figure S21. ^{1}H-^{1}H COSY spectrum of **3** in DMSO-d_6 at 700 MHz.	**S25**
**23**	Figure S22. HMBC spectrum of **3** in DMSO-d_6 at 700 MHz.	**S26**
**24**	Figure S23. HSQC spectrum of **3** in DMSO-d_6 at 700 MHz.	**S27**
**25**	Figure S24. LRESIMS of **4**.	**S28**
**26**	Figure S25. HRESIMS of **4**.	**S29**
**27**	Figure S26. ^{1}H NMR spectrum of **4** in DMSO-d_6 at 700 MHz.	**S30**
**28**	Figure S27. ^{13}C NMR spectrum of **4** in DMSO-d_6 at 175 MHz.	**S31**
**29**	Figure S28. ^{1}H-^{1}H COSY spectrum of **4** DMSO-d_6 at 700 MHz.	**S32**
**30**	Figure S29. HMBC spectrum of **4** in DMSO-d_6 at 700 MHz.	**S33**
**31**	Figure S30. HSQC spectrum of **4** in DMSO-d_6 at 700 MHz.	**S34**
**32**	Figure S31. ROESY spectrum of **4** in DMSO-d_6 at 700 MHz.	**S35**
**33**	Figure S32. LRESIMS of **5**.	**S36**
**34**	Figure S33. HRESIMS of **5**.	**S37**
**35**	Figure S34. ^{1}H NMR spectrum of **5** in DMSO-d_6 at 500 MHz.	**S38**
**36**	Figure S35. ^{13}C NMR spectrum of **5** in DMSO-d_6 at 125 MHz.	**S39**
**37**	Figure S36. ^{1}H-^{1}H COSY spectrum of **5** in DMSO-d_6 at 500 MHz.	**S40**
**38**	Figure S37. HMBC spectrum of **5** in DMSO-d_6 at 500 MHz.	**S41**
**39**	Figure S38. HSQC spectrum of **5** in DMSO-d_6 at 500 MHz.	**S42**
**40**	Figure S39. ROESY spectrum of **5** in DMSO-d_6 at 500 MHz.	**S43**
**41**	Figure S40. LRESIMS of **6**.	**S44**
**42**	Figure S41. HRESIMS of **6**.	**S45**
**43**	Figure S42. ^{1}H NMR spectrum of **6** in methanol-d_4 at 500 MHz.	**S46**
**44**	Figure S43. ^{1}H-^{1}H COSY spectrum of **6** in methanol-d_4 at 500 MHz.	**S47**
**45**	Figure S44. HMBC spectrum of **6** in methanol-d_4 at 500 MHz.	**S48**
**46**	Figure S45. HSQC spectrum of **6** in methanol-d_4 at 500 MHz.	**S49**
**47**	Figure S46. LRESIMS of **7**.	**S50**
**48**	Figure S47. HRESIMS of **7**.	**S51**
**49**	Figure S48. ^{1}H NMR spectrum of **7** in methanol-d_4 at 500 MHz.	**S52**
**50**	Figure S49. ^{1}H-^{1}H COSY spectrum of **7** in methanol-d_4 at 500 MHz.	**S53**
**51**	Figure S50. HMBC spectrum of **7** in methanol-d_4 at 500 MHz.	**S54**
**52**	Figure S51. HSQC spectrum of **7** in methanol-d_4 at 500 MHz.	**S55**
**53**	Figure S52. LRESIMS of **8**.	**S56**
**54**	Figure S53. HRESIMS of **8**.	**S57**
**55**	Figure S54. ^{1}H NMR spectrum of **8** in methanol-d_4 at 500 MHz.	**S58**
**56**	Figure S55. ^{13}C NMR spectrum of **8** in methanol-d_4 at 125 MHz.	**S59**
**57**	Figure S56. HMBC spectrum of **8** in methanol-d_4 at 500 MHz.	**S60**
**58**	Figure S57. HSQC spectrum of **8** in methanol-d_4 at 500 MHz.	**S61**
**59**	Figure S58. ROESY spectrum of **8** in methanol-d_4 at 500 MHz.	**S62**
**60**	Figure S59. HRESIMS of **9**.	**S63**
**61**	Figure S60. ^{1}H NMR spectrum of **9** in methanol-d_4 at 700 MHz.	**S64**
**62**	Figure S61. ^{13}C NMR spectrum of **9** in methanol-d_4 at 175 MHz.	**S65**

**63**	Figure S62. ^{1}H-^{1}H COSY spectrum of **9** methanol-*d$_4$* at 700 MHz.	**S66**
**64**	Figure S63. HMBC spectrum of **9** methanol-*d$_4$* at 700 MHz.	**S67**
**65**	Figure S64. HSQC spectrum of **9** methanol-*d$_4$* at 700 MHz.	**S68**
**66**	Figure S65. ROESY spectrum of **9** methanol-*d$_4$* at 700 MHz.	**S69**
**67**	Figure S66. LRESIMS of **10**.	**S70**
**68**	Figure S67. HRESIMS of **10**.	**S71**
**69**	Figure S68. ^{1}H NMR spectrum of **10** in methanol-*d$_4$* at 500 MHz.	**S72**
**70**	Figure S69. ^{1}H-^{1}H COSY spectrum of **10** in methanol-*d$_4$* at 500 MHz.	**S73**
**71**	Figure S70. HMBC spectrum of **10** in methanol-*d$_4$* at 500 MHz.	**S74**
**72**	Figure S71. HSQC spectrum of **10** in methanol-*d$_4$* at 500 MHz.	**S75**
**73**	Figure S72. HRESIMS of **11**.	**S76**
**74**	Figure S73. ^{1}H NMR spectrum of **11** in acetone-*d$_6$* at 500 MHz.	**S77**
**75**	Figure S74. ^{13}C NMR spectrum of **11** in acetone-*d$_6$* at 125 MHz.	**S78**
**76**	Figure S75. ^{1}H-^{1}H COSY spectrum of **11** in acetone-*d$_6$* at 500 MHz.	**S79**
**77**	Figure S76. HMBC spectrum of **11** in acetone-*d$_6$* at 500 MHz.	**S80**
**78**	Figure S77. HSQC spectrum of **11** in acetone-*d$_6$* at 500 MHz.	**S81**
**79**	Figure S78. HRESIMS of **12**.	**S82**
**80**	Figure S79. ^{1}H NMR spectrum of **12** in acetone-*d$_6$* at 500 MHz.	**S83**
**81**	Figure S80. HRESIMS spectra of **13**.	**S84**
**82**	Figure S81. ^{1}H NMR spectrum of **13** in DMSO-*d$_6$* at 500 MHz.	**S85**
**83**	Figure S82. ^{1}H-^{1}H COSY spectrum of **13** in DMSO-*d$_6$* at 500 MHz.	**S86**
**84**	Figure S83. Marfey's results of omnipolyphilin A and tyrosine.	**S87**
**85**	Figure S84. Marfey's results of omnipolyphilin A and 3,5-dichloro-tyrosine.	**S87**
**86**	Figure S85. Experimental ECD spectrum of compound **3** in MeOH.	**S88**
**87**	Figure S86. Experimental ECD spectrum of compound **4** in MeOH.	**S88**
**88**	Table S1. Chemical shift differences between the (*S*)-MTPA and (*R*)-MTPA esters of **4**.	**S88**
**89**	Antimicrobial activity assay	**S89**
**90**	Cytotoxicity (MTT) assay	**S89**
**91**	Table S2. Cytotoxic activity (IC_{50}) of tested compounds.	**S90**
**92**	Table S3. Antimicrobial activity (MIC) of tested compounds.	**S90**
**93**	Table S4. Nematicidal activity of tested compounds against *Caenorhabditis elegans*.	**S91**
**94**	Figure S87. Effects on the biofilm formation of *S. aureus* after 24 h treatment with omnipolyphilin A (**1**).	**S91**
**95**	Figure S88. Effects on the biofilm formation of *S. aureus* after 24 h treatment with talaroderxine D (**4**).	**S91**
**96**	Figure S89. Effects on the biofilm formation of *S. aureus* after 24 h treatment with talaroderxine C (**5**).	**S91**
**97**	Table S5. Inhibition of biofilm formation of S. *aureus* by omnipolyphilin A (**1**), talaroderxines D (**4**) and C (**5**) at different concentrations.	**S92**
**98**	Figure S90. Separation scheme of the isolated compounds **1**-**6**, **10** and **13**.	**S92**
**99**	Table S6. Separation parameters of *P. sieberi* 17C BRFT + WOFT methanol extract.	**S93**
**100**	Table S7. Separation parameters of fraction F7.	**S93**
**101**	Table S8. Separation parameters of fraction F8.	**S93**
**102**	Table S9. Separation parameters of fraction F10.	**S93**
**103**	Table S10. Separation parameters of fraction F9.	**S94**
**104**	Figure S91. Separation scheme of the isolated compounds **7**-**9**.	**S94**
**105**	Table S11. Separation parameters of *P. sieberi* 17A BRFT + WOFT methanol extract.	**S95**
**106**	Table S12. Separation parameters of fraction F5.	**S95**
**107**	Table S13. Separation parameters of fraction F6.	**S96**
**108**	Figure S92. ^{1}H NMR spectrum of **4** in pyridine-*d$_5$* at 700 MHz.	**S97**
**109**	Figure S93. ^{1}H-^{1}H COSY spectrum of **4** in pyridine-*d$_5$* at 700 MHz.	**S98**
**110**	Figure S94. ^{1}H NMR spectrum of 14-*O*-(*R*)-MTPA ester of **4** in pyridine-*d$_5$* at 700 MHz.	**S99**
**111**	Figure S95. ^{1}H-^{1}H COSY spectrum of 14-*O*-(*R*)-MTPA ester of **4** in pyridine-*d$_5$* at 700 MHz.	**S100**
**112**	Figure S96. ^{1}H NMR spectrum of 14-*O*-(*S*)-MTPA ester of **4** in pyridine-*d$_5$* at 700 MHz.	**S101**
**113**	Figure S97. ^{1}H-^{1}H COSY spectrum of 14-*O*-(*S*)-MTPA ester of **4** in pyridine-*d$_5$* at 700 MHz.	**S102**
**114**	Figure S98. Lowest-energy MMFF conformers of a) (L-Tyr^{1},D-Tyr^{2})-**1** and b) (D-Tyr^{1},L-Tyr^{2})-**1** with the characteristic H-8a–Tyr2-H-α protons [d = 2.20 Å for a) and 4.12 Å for b)].	**S103**
**115**	Figure S99. Thirteen low-energy overlapped solution conformers of (L-Tyr^{1},D-Tyr^{2})-**1** (≥ 1% Boltzmann population; level of optimization: ωB97X/TZVP PCM/MeCN).	**S103**
**116**	Figure S100. Experimental ECD spectrum of **1** (black) compared with the CAM-B3LYP sTDA spectrum of (L-Tyr^{1},D-Tyr^{2})-**1** (dark yellow). Level of DFT optimization: ωB97X/TZVP PCM/MeCN.	**S104**
**117**	Figure S101. Thirty low-energy overlapped solution conformers of (*R*)-**3** (≥ 1% Boltzmann population) belonging to two groups based on the conformation of the heteroring [28 conformers with a sum Boltzmann population of 69.9% for group A and two conformers with 2.4% for group B; level of optimization: ωB97X/TZVP PCM/MeCN].	**S104**
**118**	Table S14. Cartesian coordinates and energies of the low-energy conformers calculated at the ωB97X/TZVP PCM/MeCN level.	**S105**
**119**	Table S15. Cartesian coordinates and energies of the low-energy conformers calculated at the ωB97X/TZVP PCM/MeOH level.	**S116**

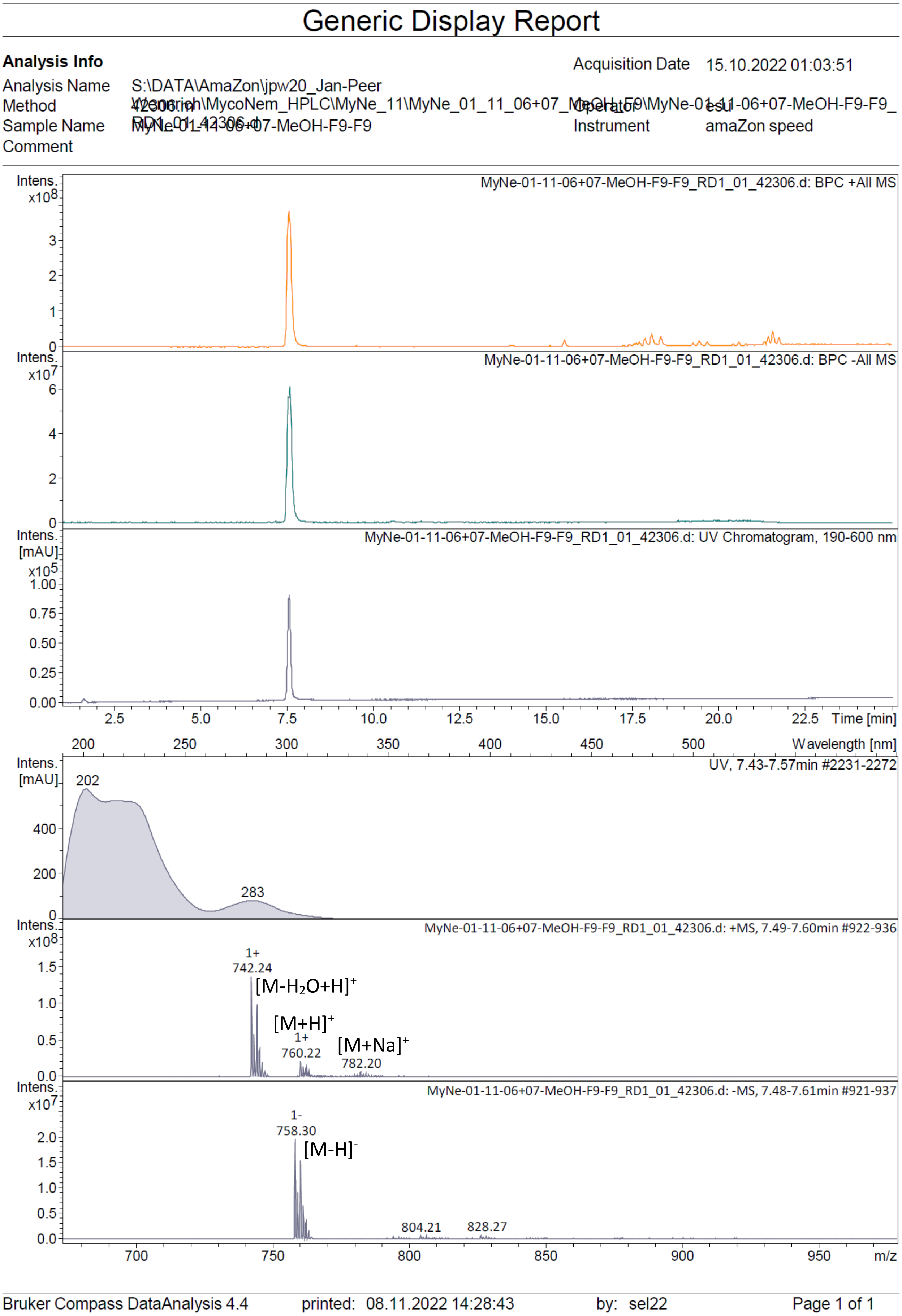

Figure S1. LRESIMS of **1**.

Generic Display Report

Analysis Info

Analysis Name	S:\DATA\MaXis\ESE22_EllenSepanian\22_11\MyNe-01-11-06+07-MeOH-F9-F9_22_01_10978.d	Acquisition Date	01.11.2022 11:07:34
Method	pos_säure_10000_screening_ms_100_2500_line.m	Operator	ate06
Sample Name	MyNe-01-11-06+07-MeOH-F9-F9	Instrument	maXis
Comment	Screening01<br>Waters Acquity UPLC BEH C_18 1,7um 2.1x50mm		

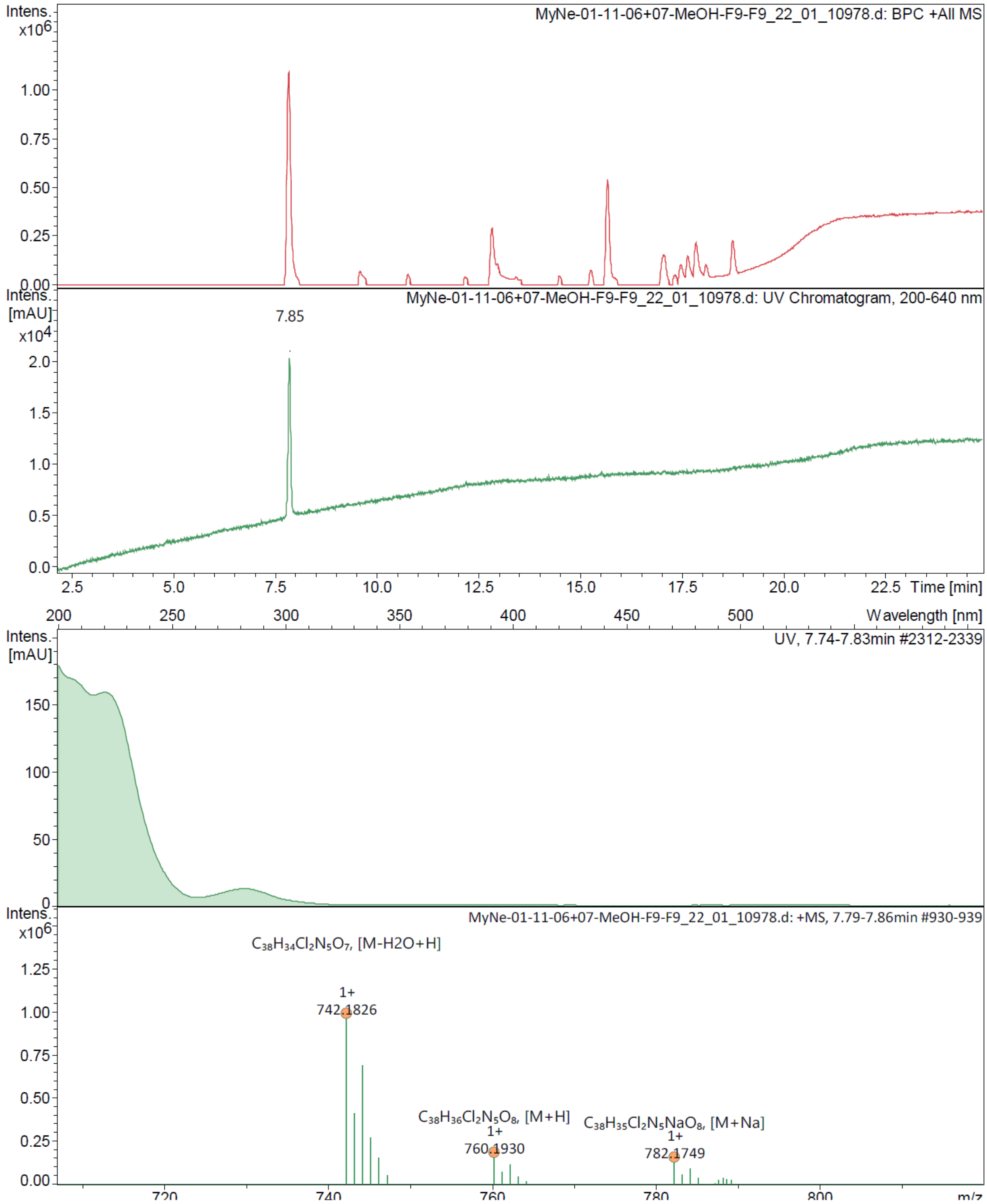

Bruker Compass DataAnalysis 4.4 printed: 01.06.2023 19:58:56 by: sel22 Page 1 of 1

Figure S2. HRESIMS of **1**.

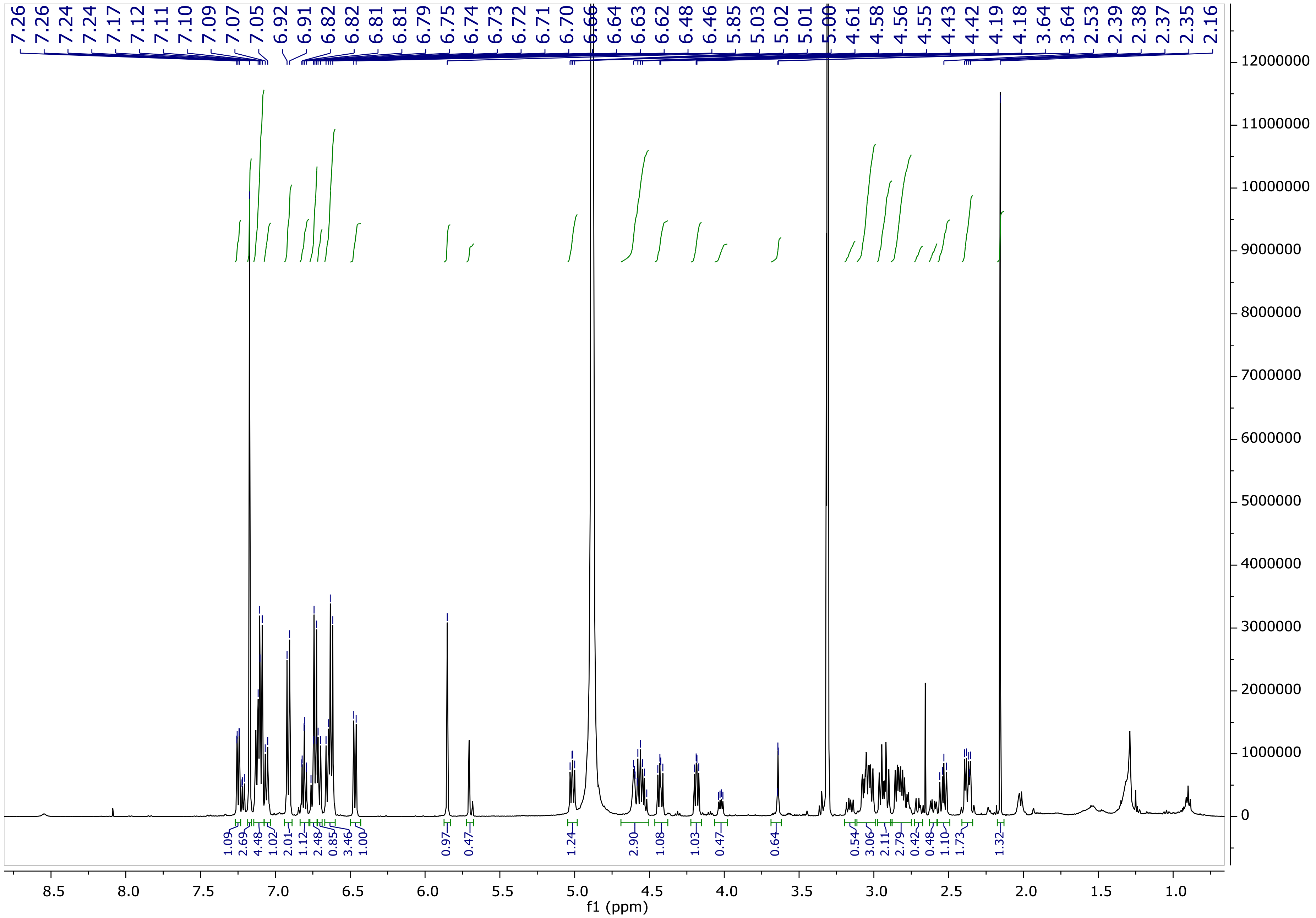

Figure S3. ^{1}H NMR spectrum of **1** in methanol-d_4 at 500 MHz.

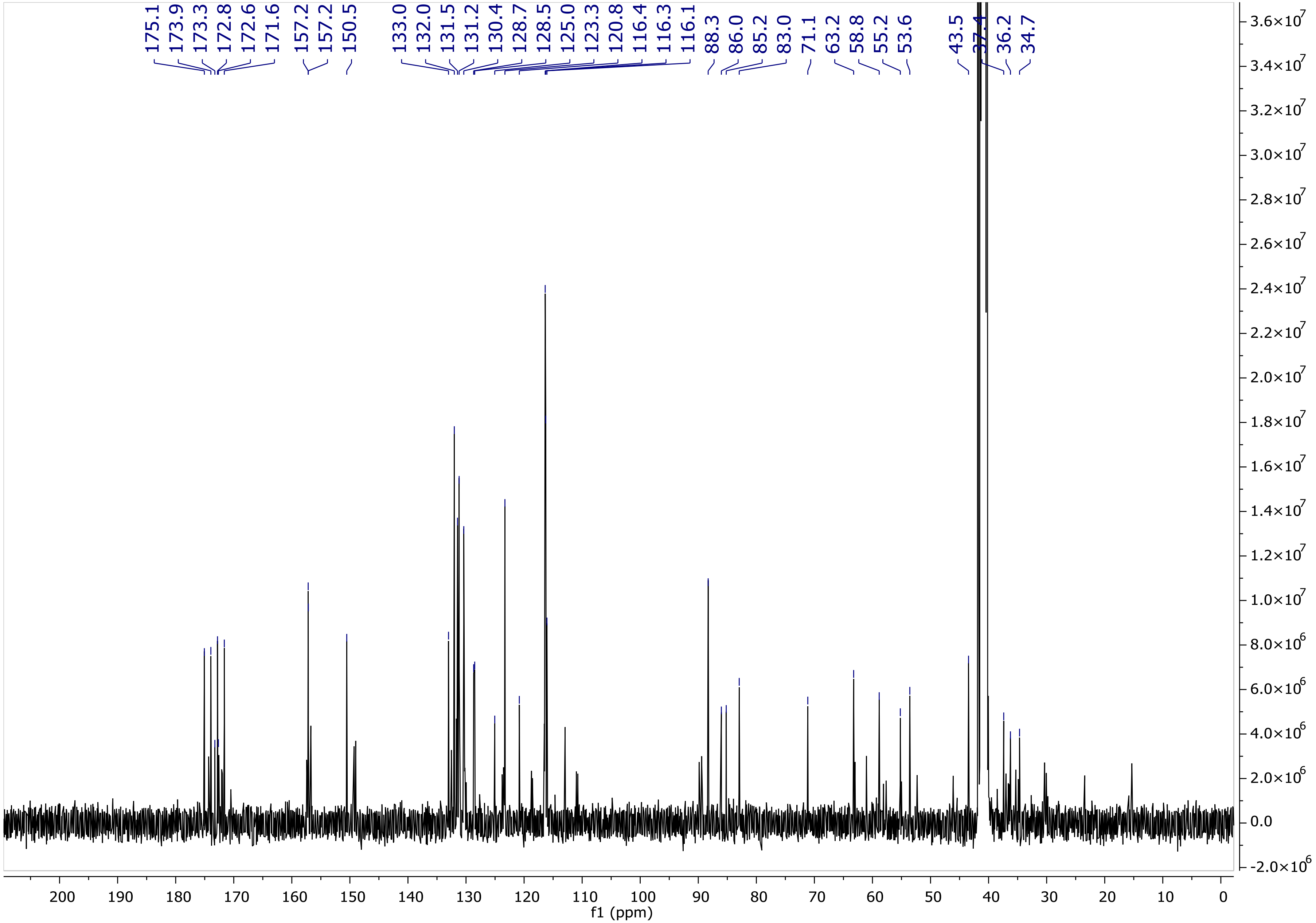

Figure S4. ^{13}C NMR spectrum of **1** in methanol-d_4 at 125 MHz.

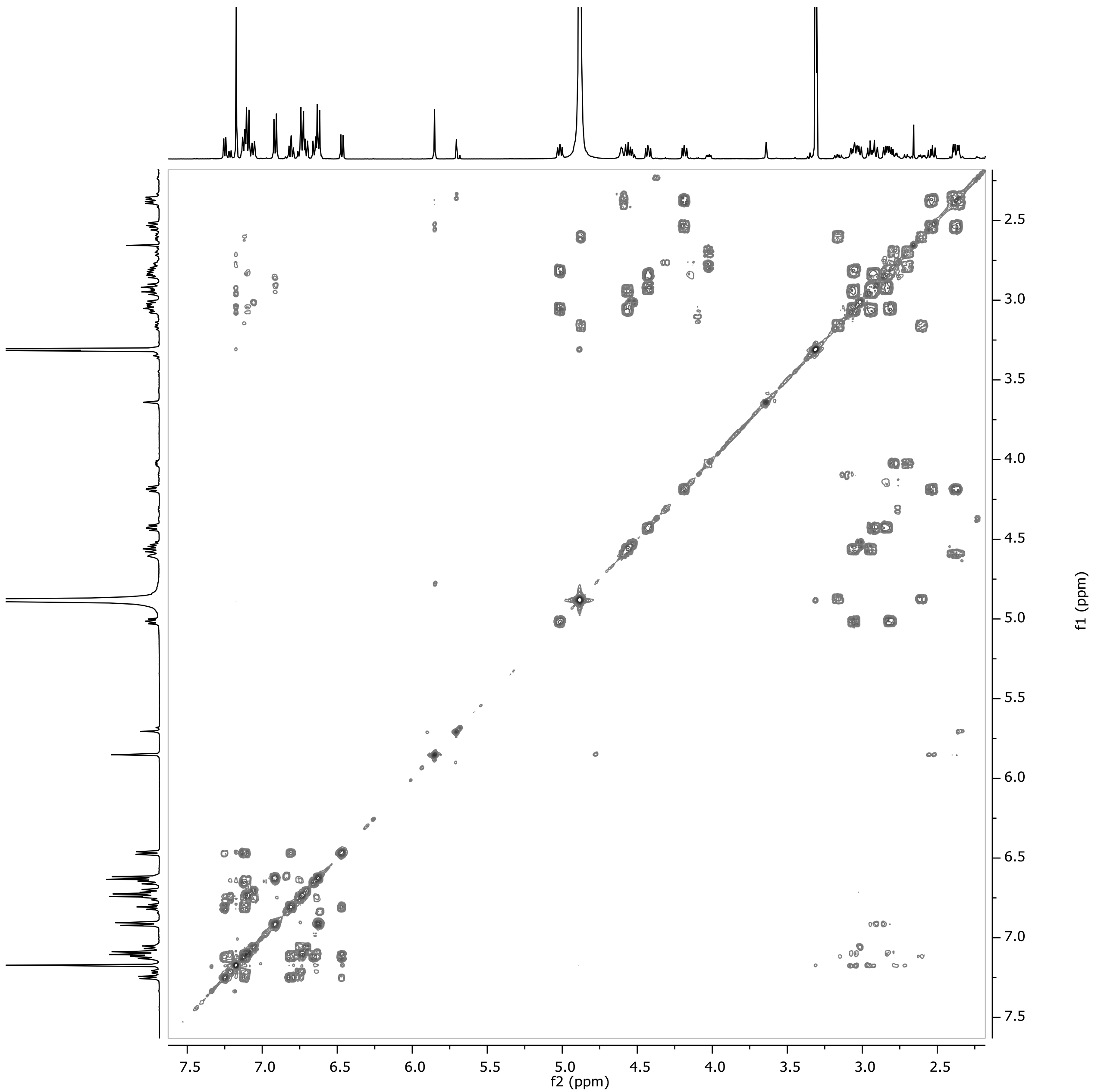

Figure S5. ^{1}H-^{1}H COSY spectrum of **1** in methanol-d_4 at 500 MHz.

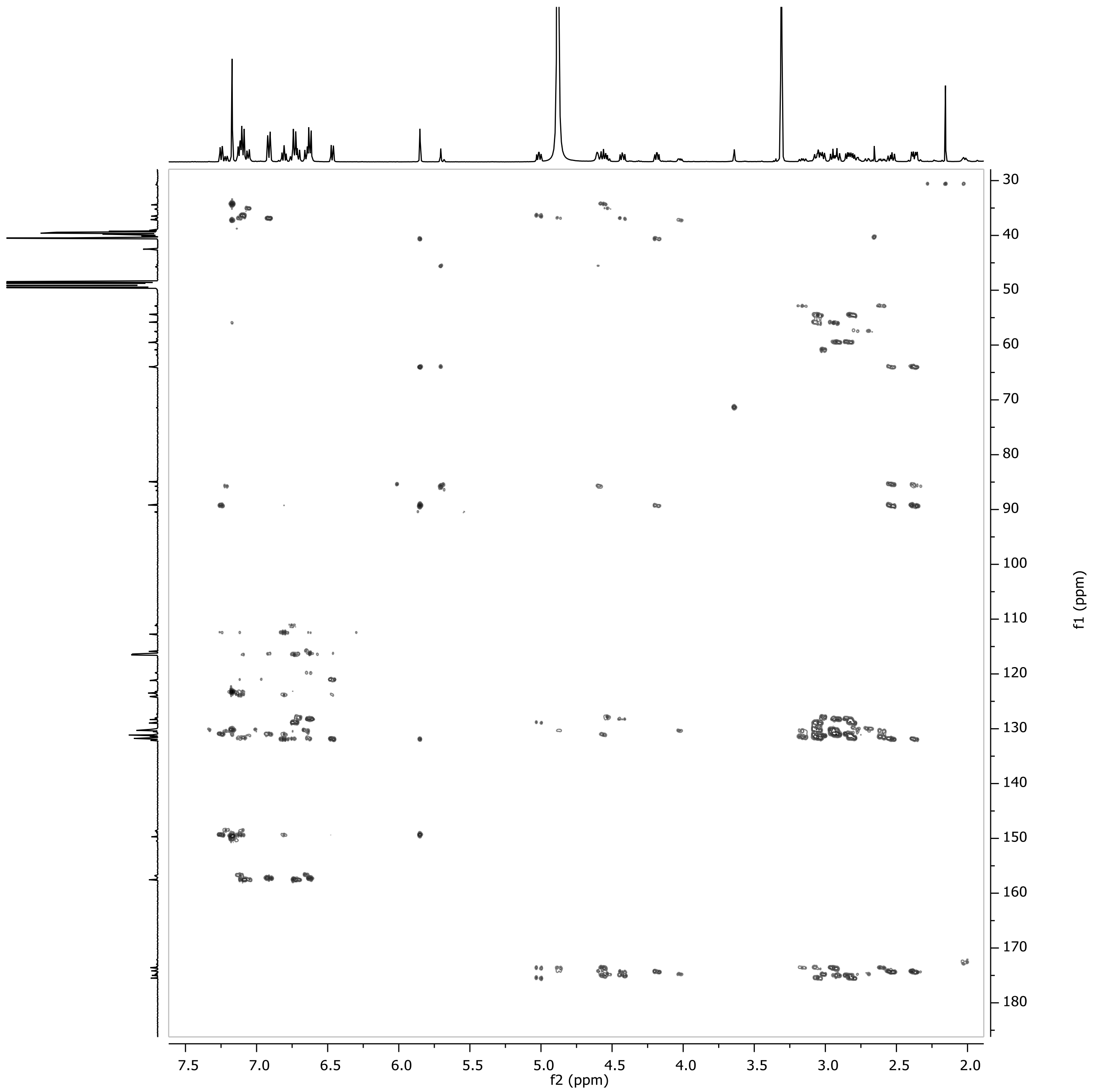

Figure S6. HMBC spectrum of **1** in methanol-d_4 at 500 MHz.

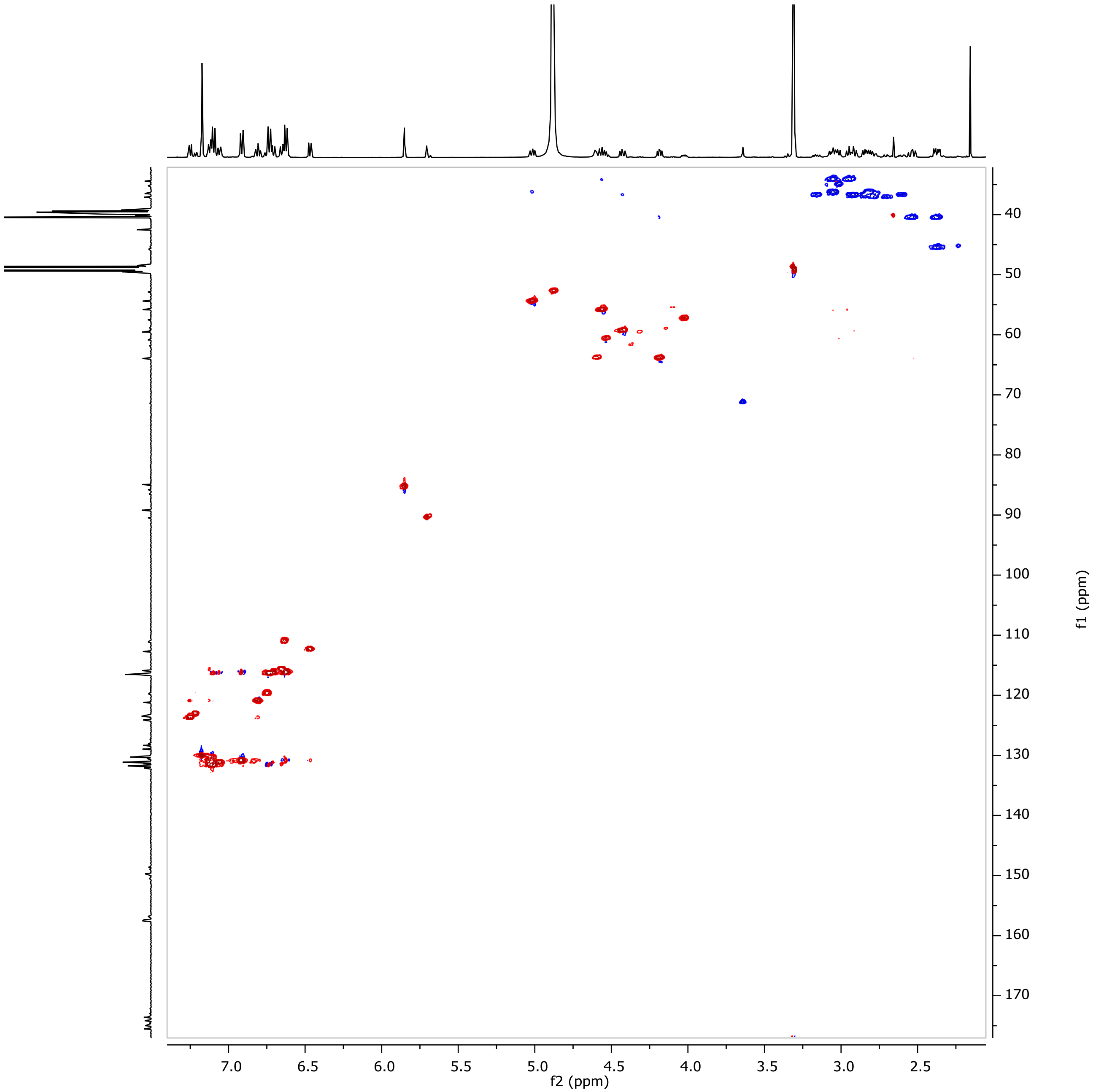

Figure S7. HSQC spectrum of **1** in methanol-d_4 at 500 MHz.

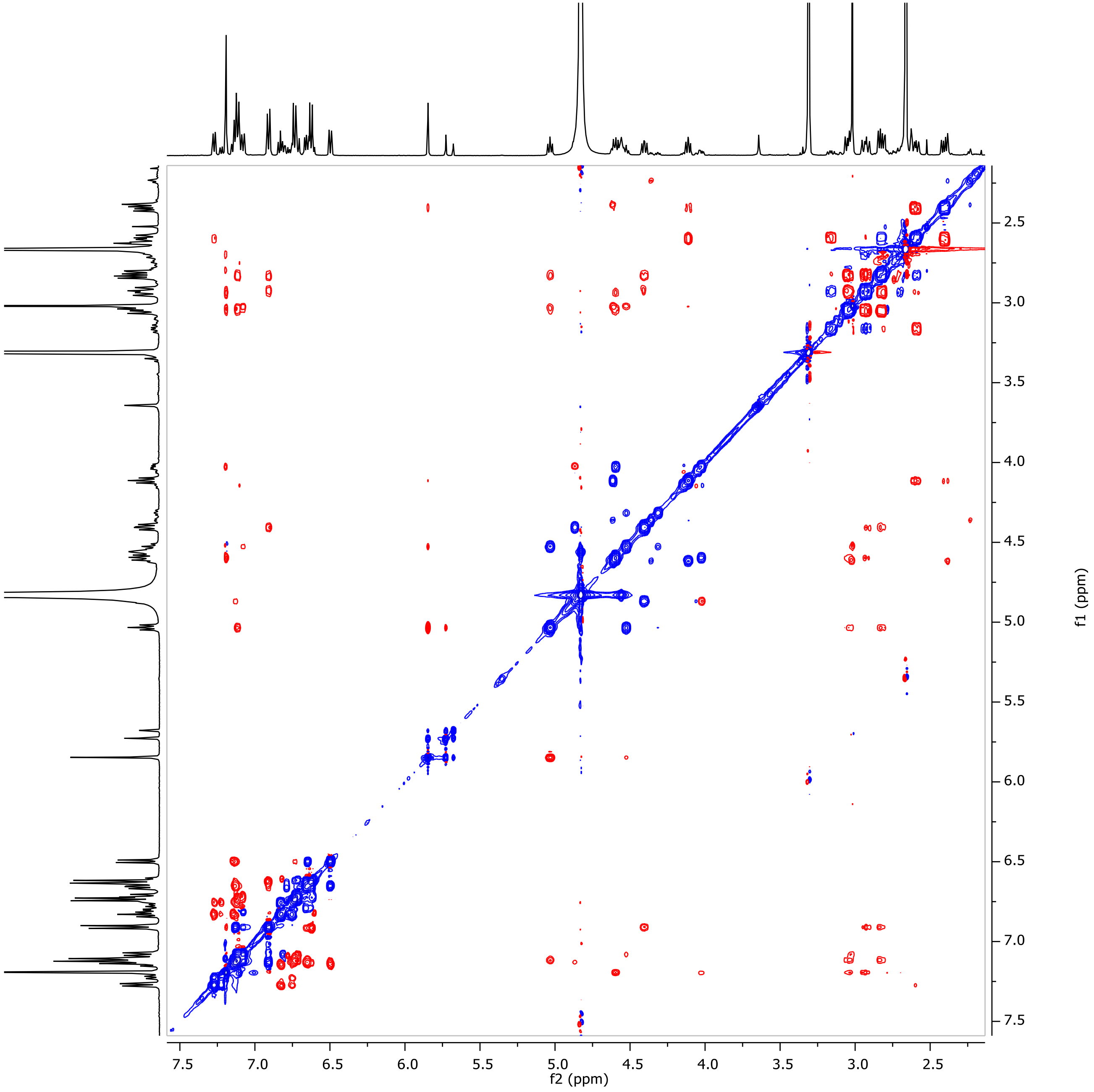

Figure S8. ROESY spectrum of **1** in methanol-d_4 at 500 MHz.

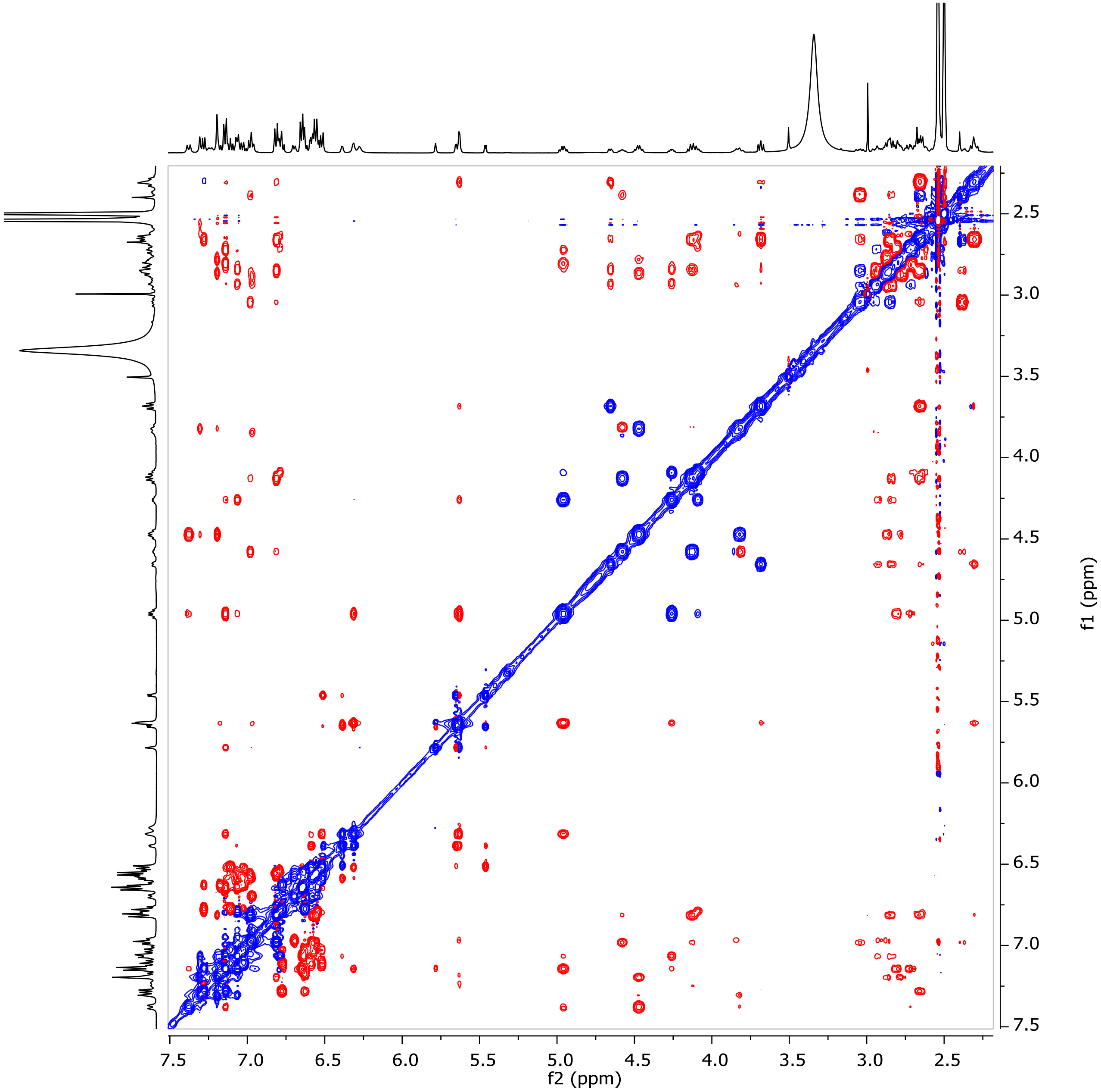

Figure S8a. ROESY spectrum of **1** in DMSO-d_6 at 500 MHz.

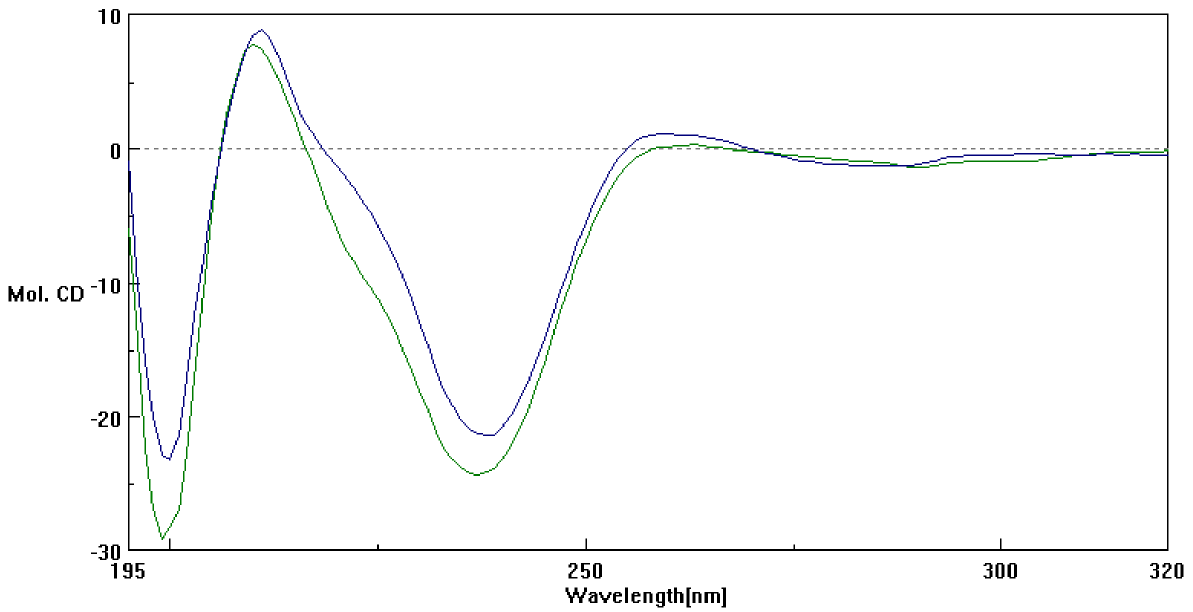

Figure S9. Experimental ECD spectra of **1** in acetonitrile (blue) and methanol (green).

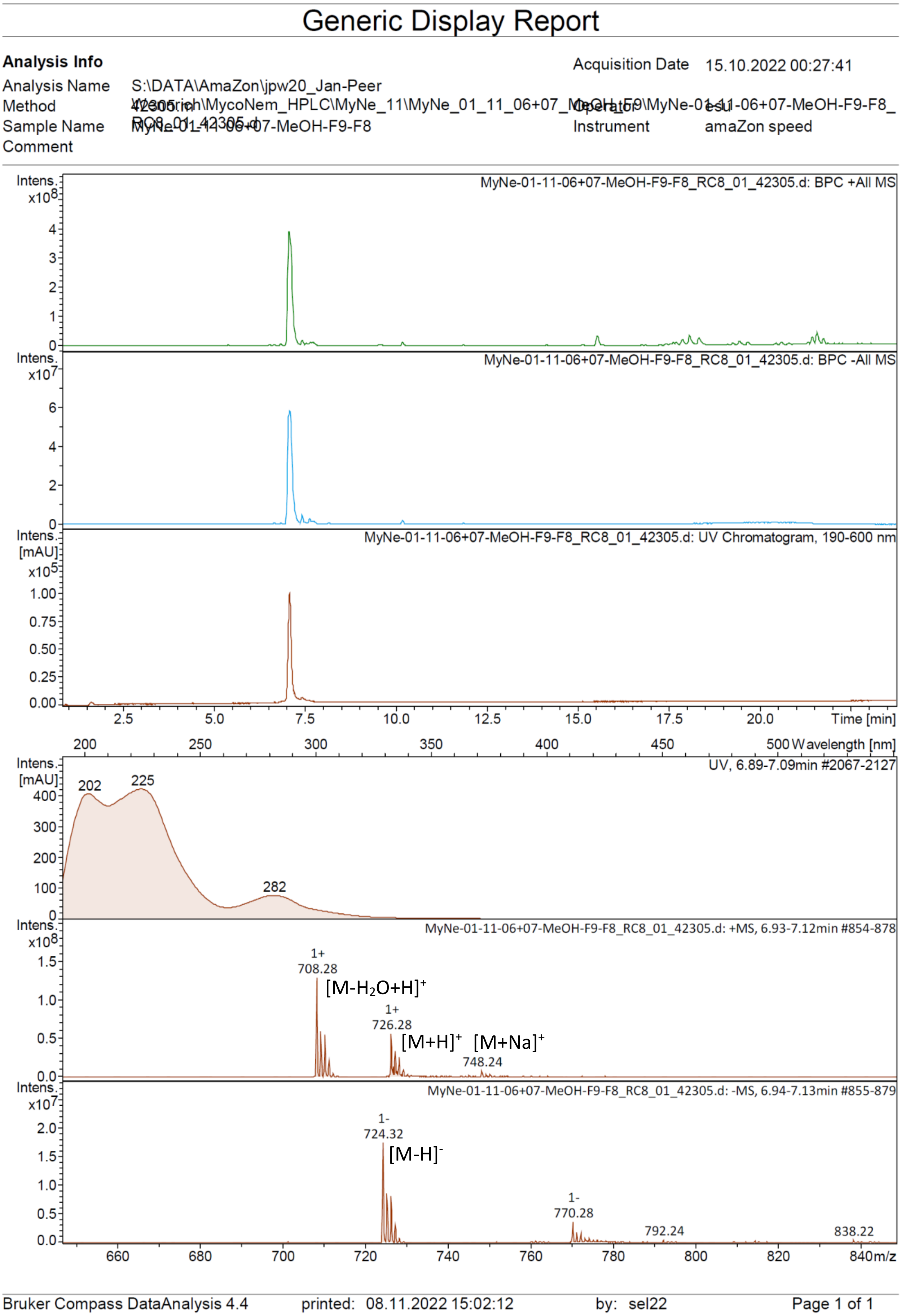

Figure S10. LRESIMS of **2**.

Generic Display Report

Analysis Info

		Acquisition Date	13.01.2023 13:12:10
Analysis Name	S:\DATA\MaXis\ESE22_Ellen Sepanian\23_01\MyNe-01-11-06+07-MeOH-F9-F8_24_01_11194.d		
Method	pos_säure_10000_screening_ms_100_2500_line.m	Operator	ate06
Sample Name	MyNe-01-11-06+07-MeOH-F9-F8	Instrument	maXis
Comment	Screening01<br>Waters Acquity UPLC BEH C_18 1,7um 2.1x50mm		

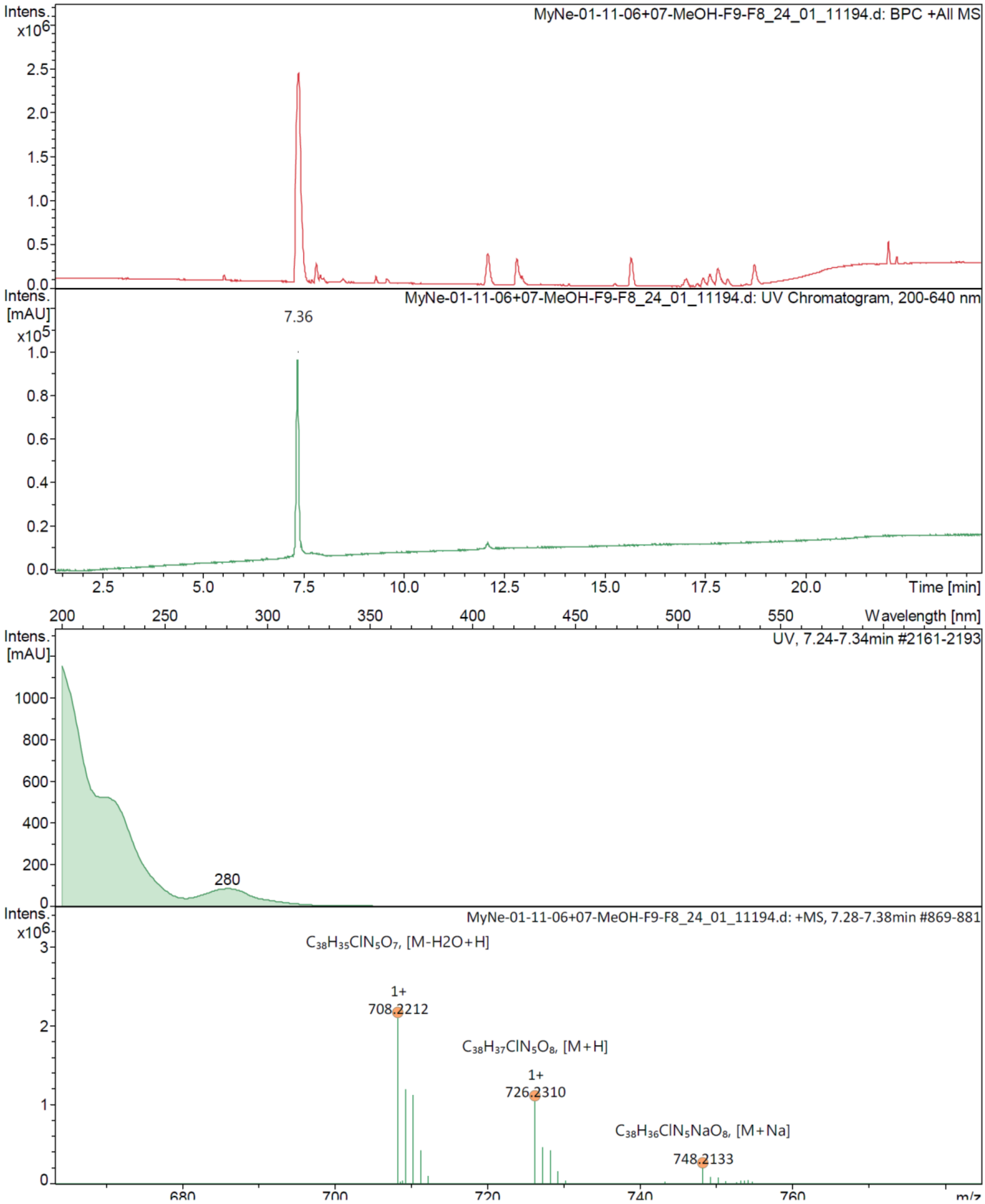

Figure S11. HRESIMS of **2**.

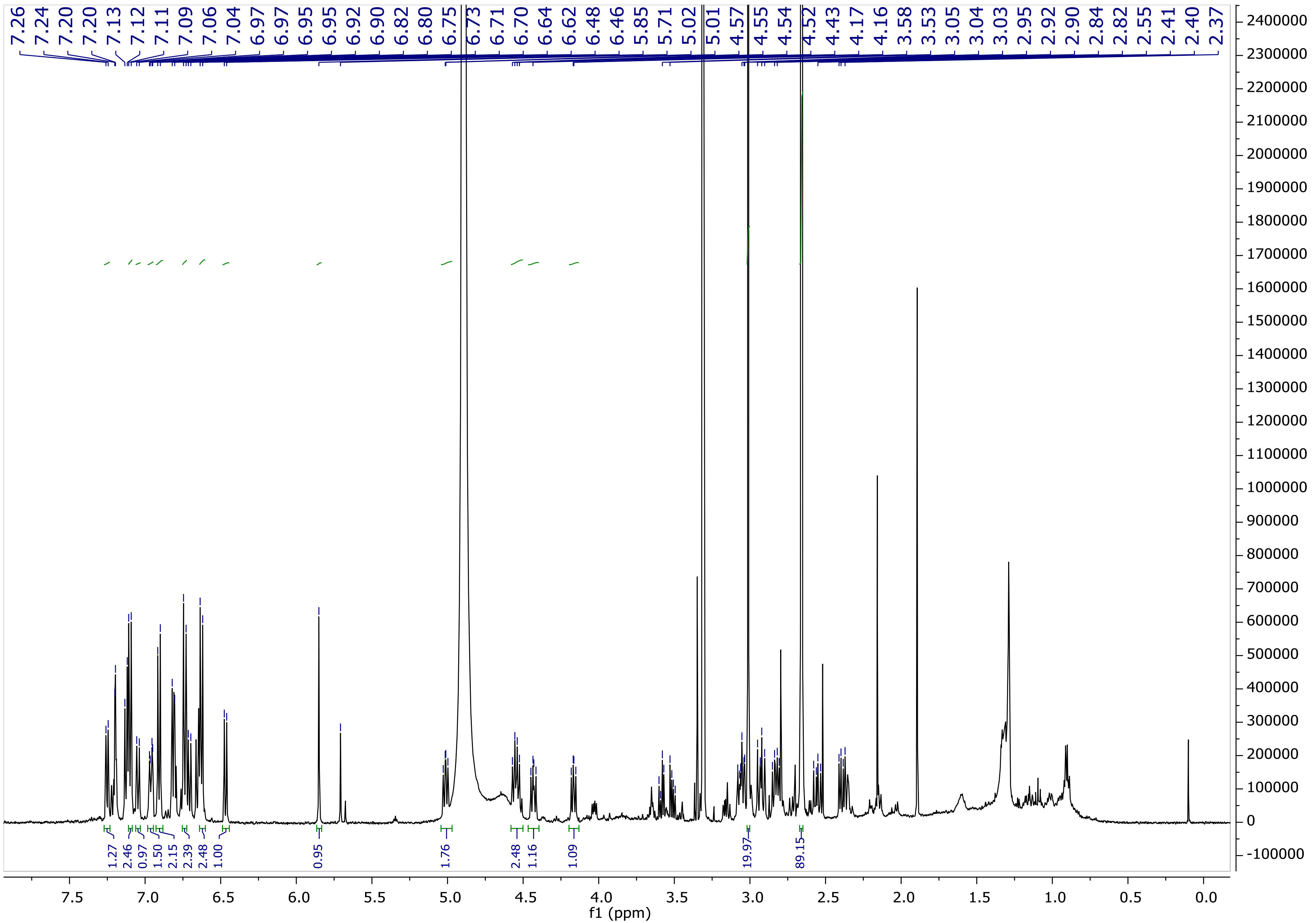

Figure S12. ^{1}H NMR spectrum of **2** in methanol-d_4 at 700 MHz.

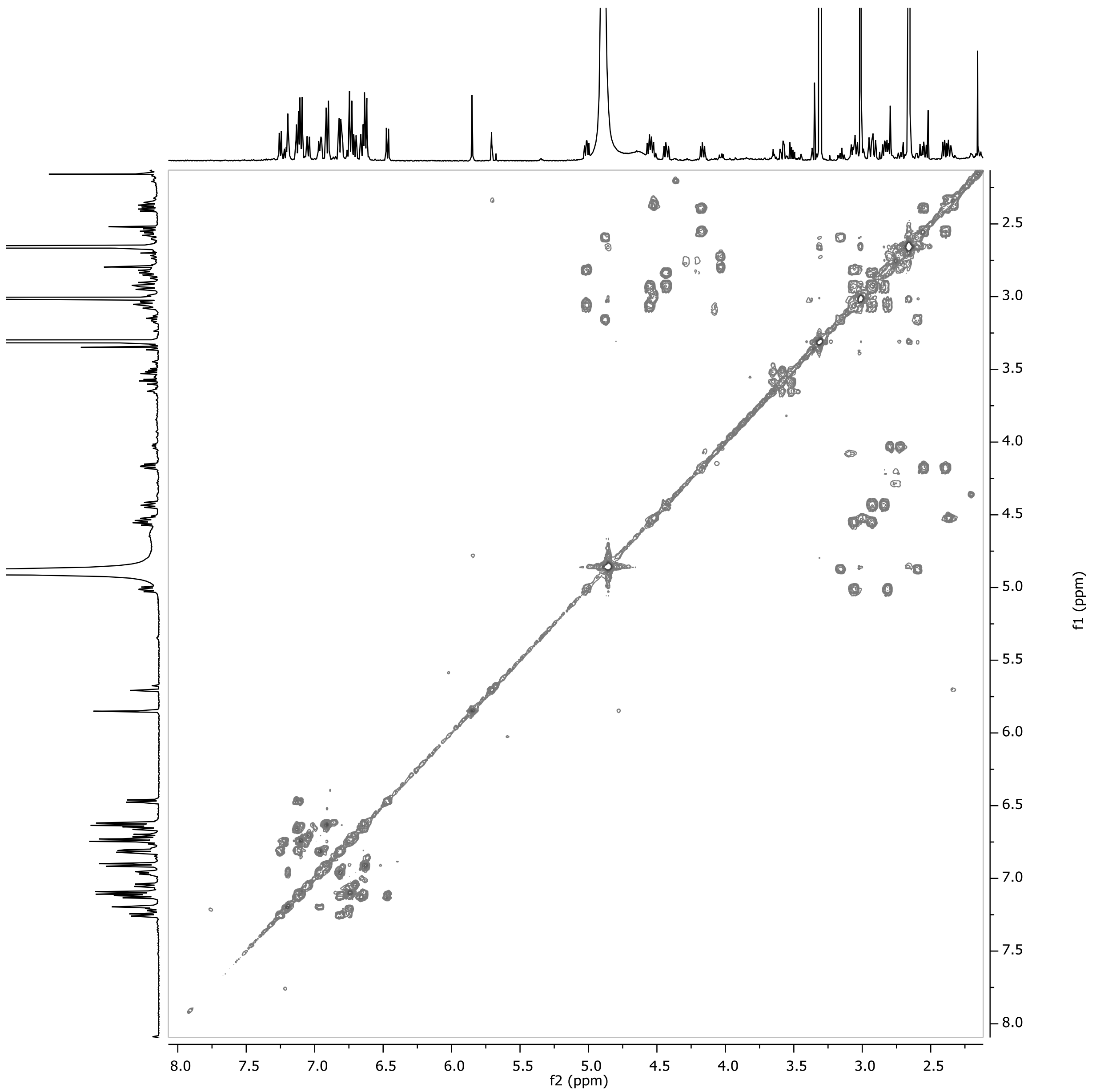

Figure S13. ^{1}H-^{1}H COSY spectrum of **2** in methanol-d_4 at 700 MHz.

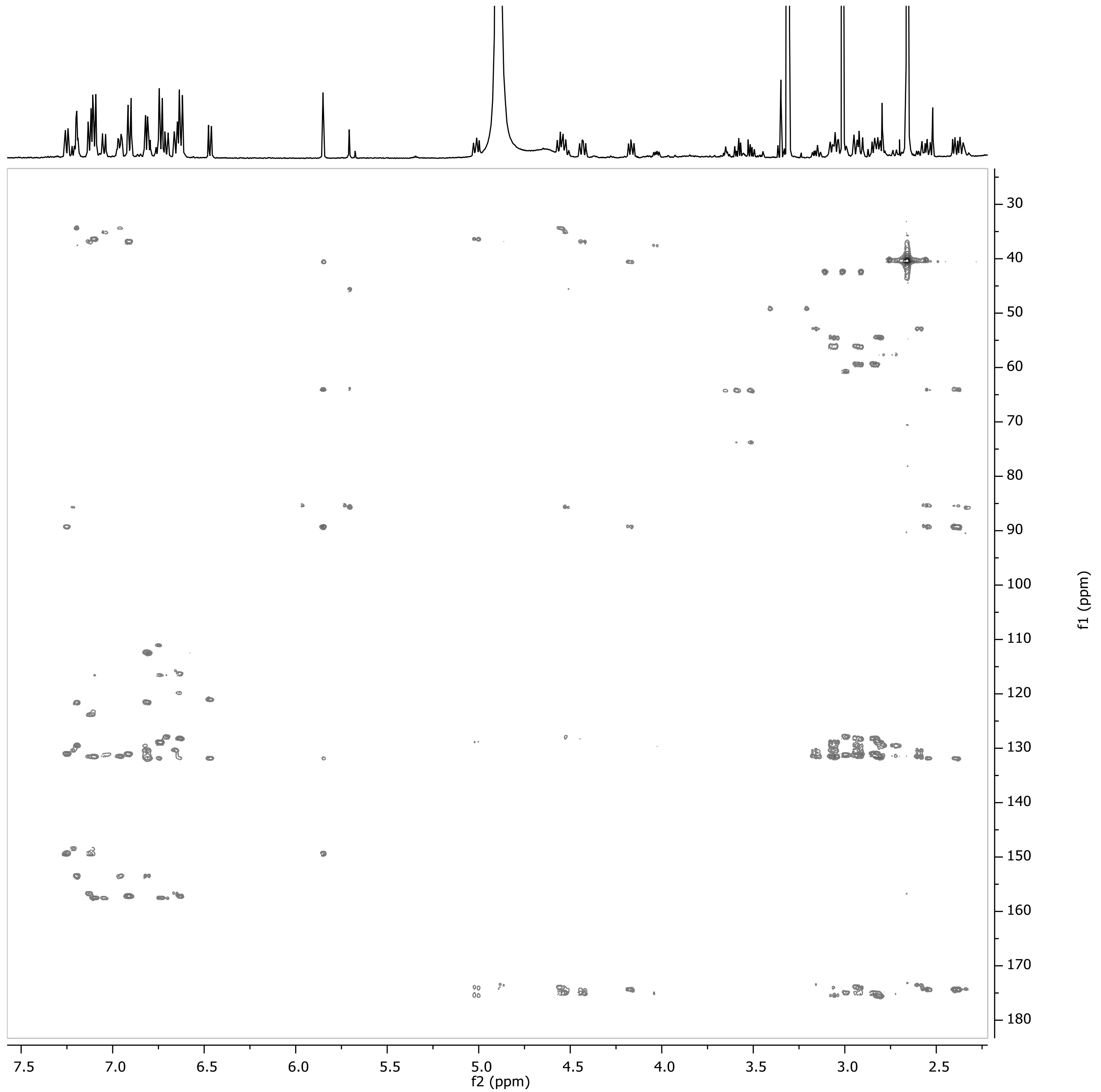

Figure S14. HMBC spectrum of **2** in methanol-d_4 at 700 MHz.

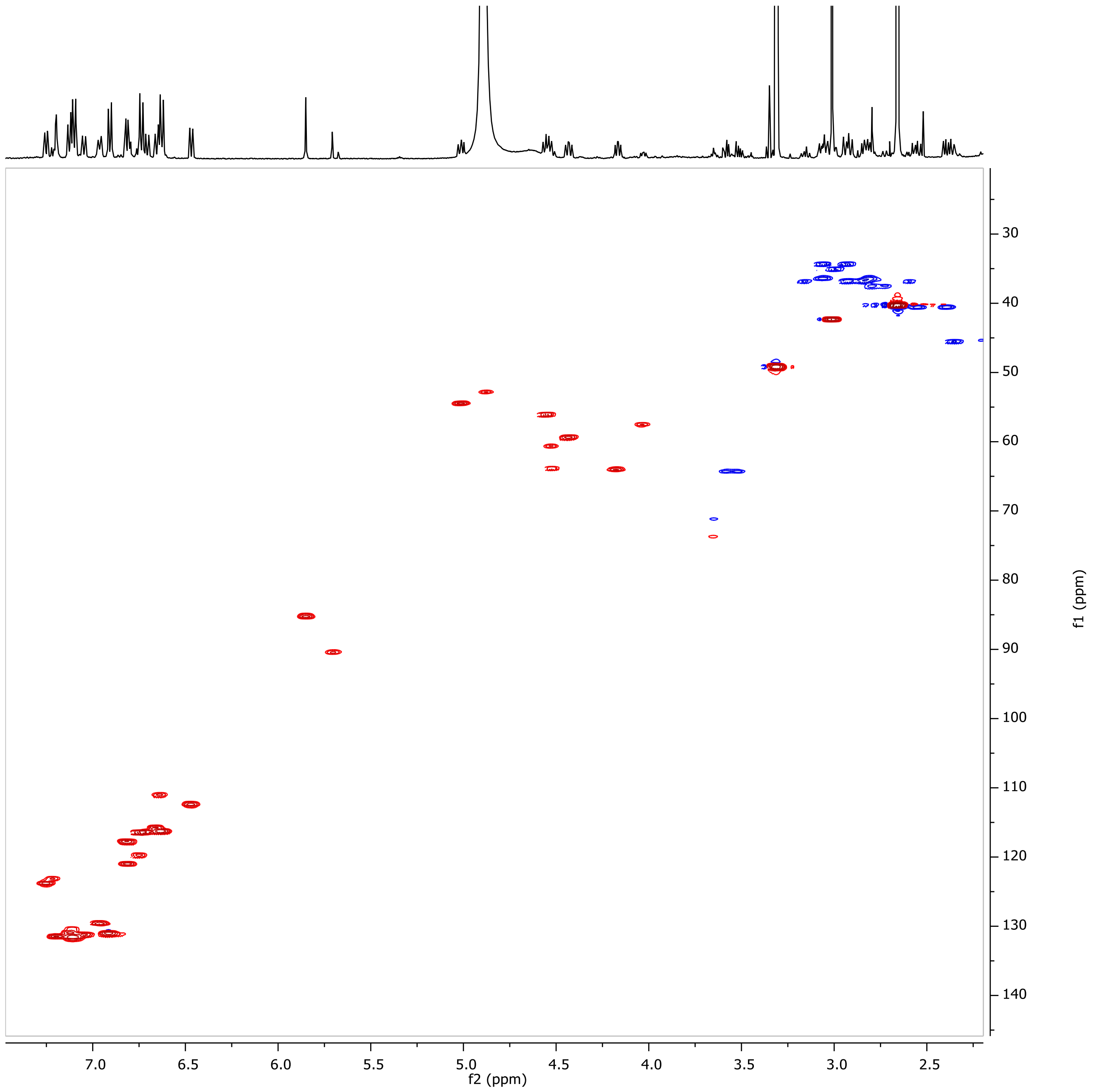

Figure S15. HSQC spectrum of **2** in methanol-d_4 at 700 MHz.

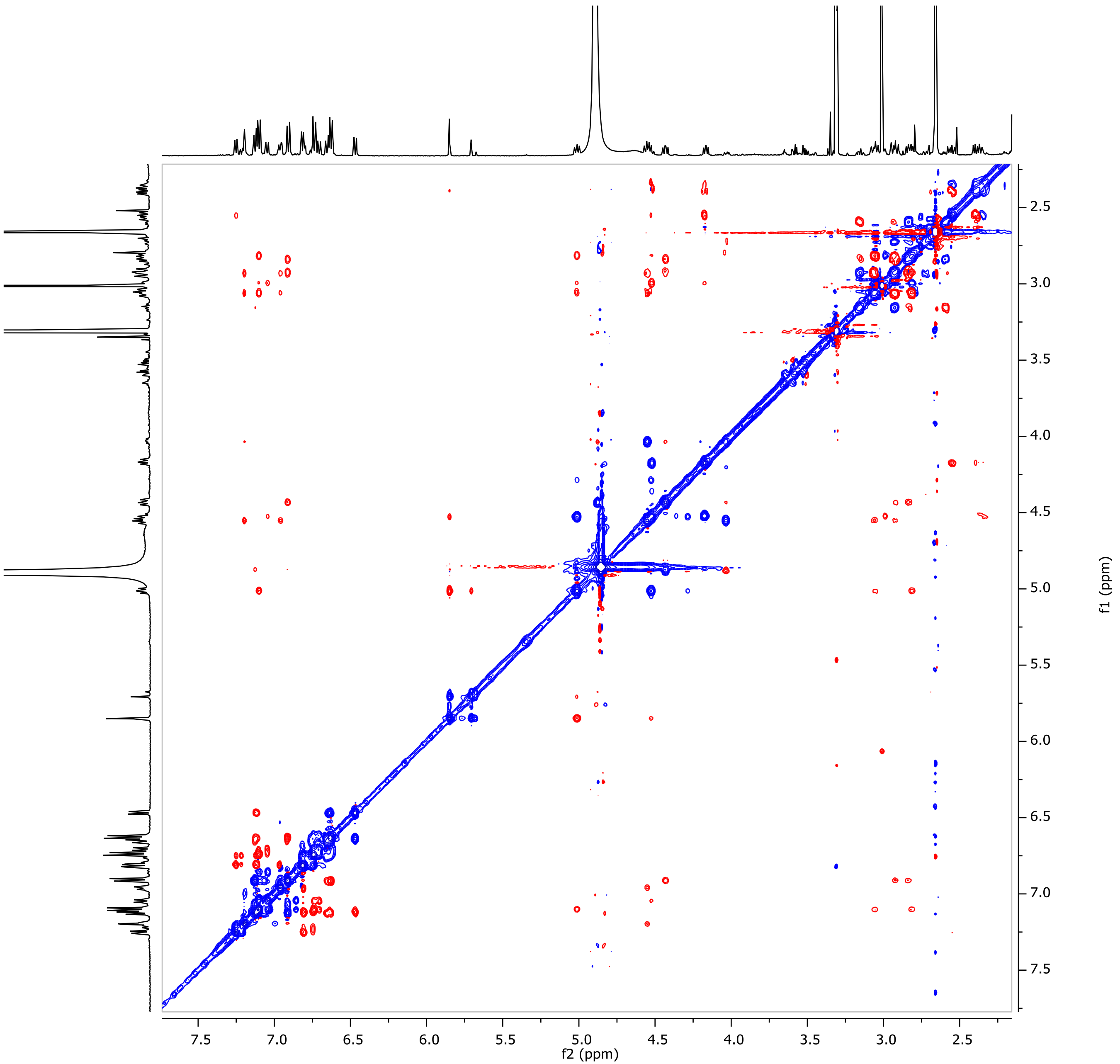

Figure S16. ROESY spectrum of **2** in methanol-d_4 at 700 MHz.

Generic Display Report

Analysis Info

Analysis Name	S:\DATA\AmaZon\jpw20_Jan-Peer	Acquisition Date	13.01.2023 05:44:07
Method	[illegible]\MycoNem_HPLC\MyNe_11\01-11-06+07-MeOH-F7-F7+F8\01-11-06+07-MeOH-F7-F7+F8-F19_	Operator	esu
Sample Name	01-11-06+07-MeOH-F7-F7+F8-F19	Instrument	amaZon speed
Comment			

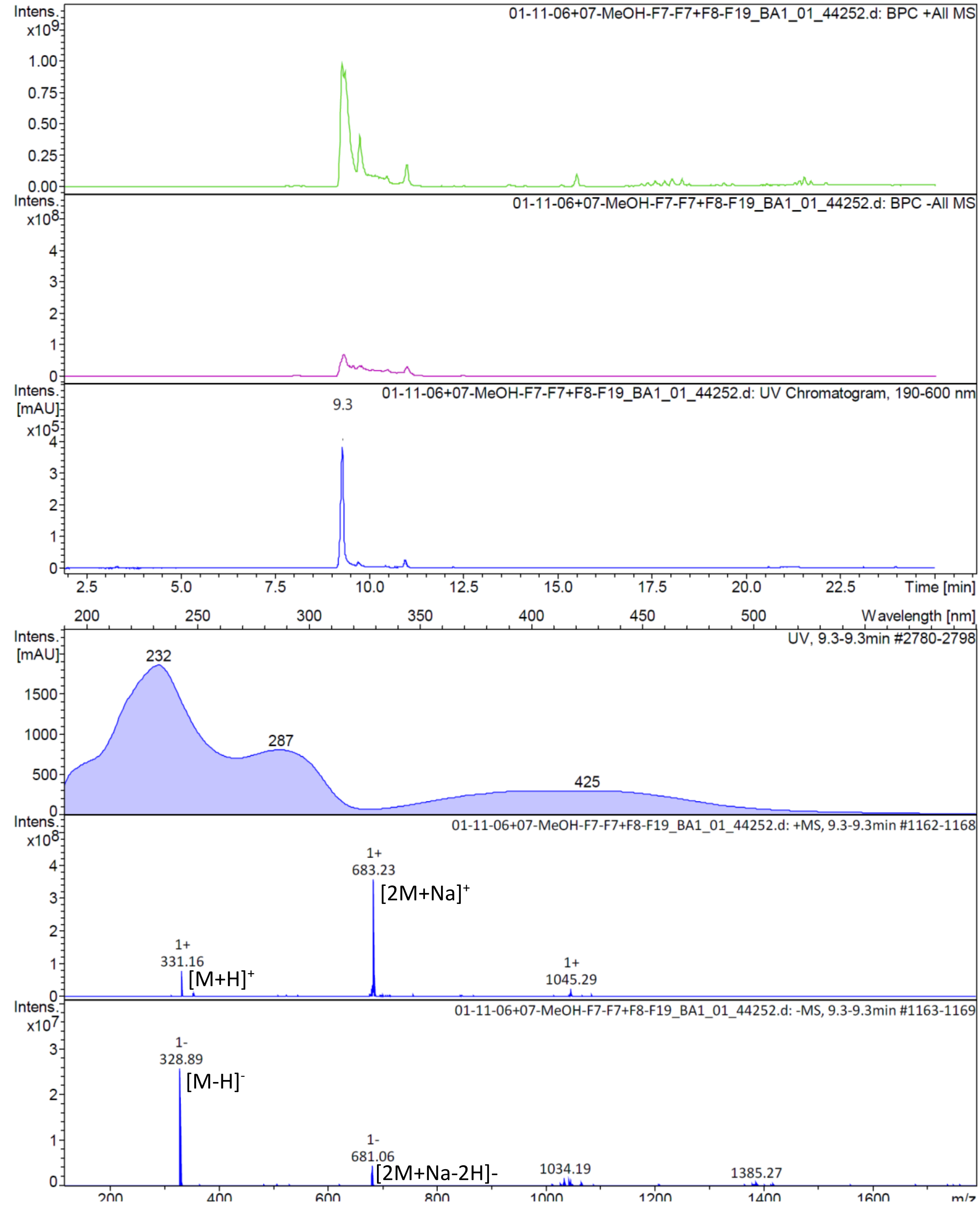

Bruker Compass DataAnalysis 4.4 printed: 01.06.2023 19:33:54 by: sel22 Page 1 of 1

Figure S17. LRESIMS of **3**.

Generic Display Report

Analysis Info

		Acquisition Date	24.01.2023 15:15:30
Analysis Name	S:\DATA\MaXis\ESE22_EllenSepanian\23_01\01-11-06+07-MeOH-F7-F7+F8-F20_12_01_11217.d		
Method	pos_säure_10000_screening_ms_100_2500_line.m	Operator	ate06
Sample Name	F20	Instrument	maXis
Comment	Screening01<br>Waters Acquity UPLC BEH C_18 1,7um 2.1x50mm		

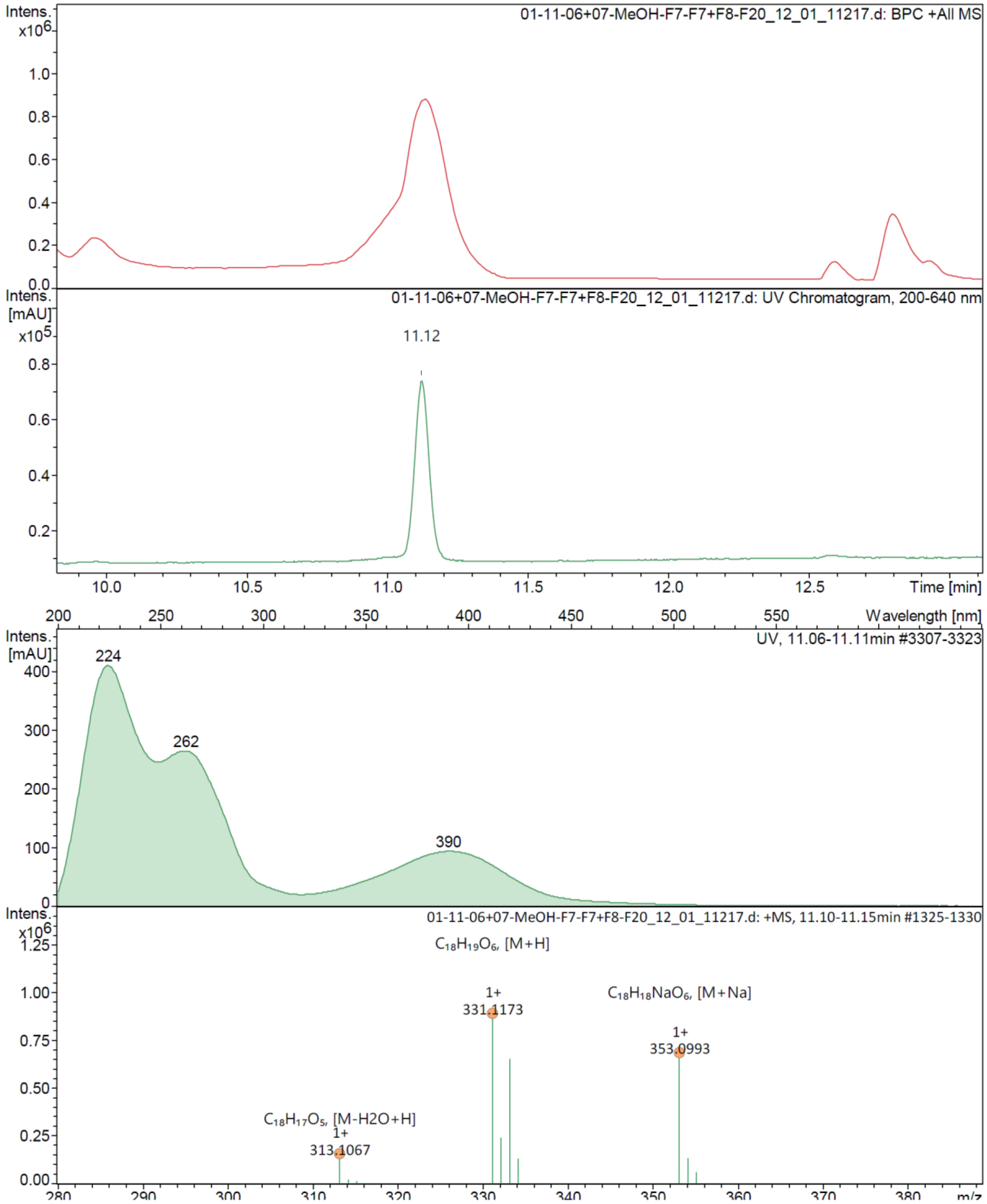

Bruker Compass DataAnalysis 4.4 printed: 01.06.2023 19:47:24 by: sel22 Page 1 of 1

Figure S18. HRESIMS of **3**.

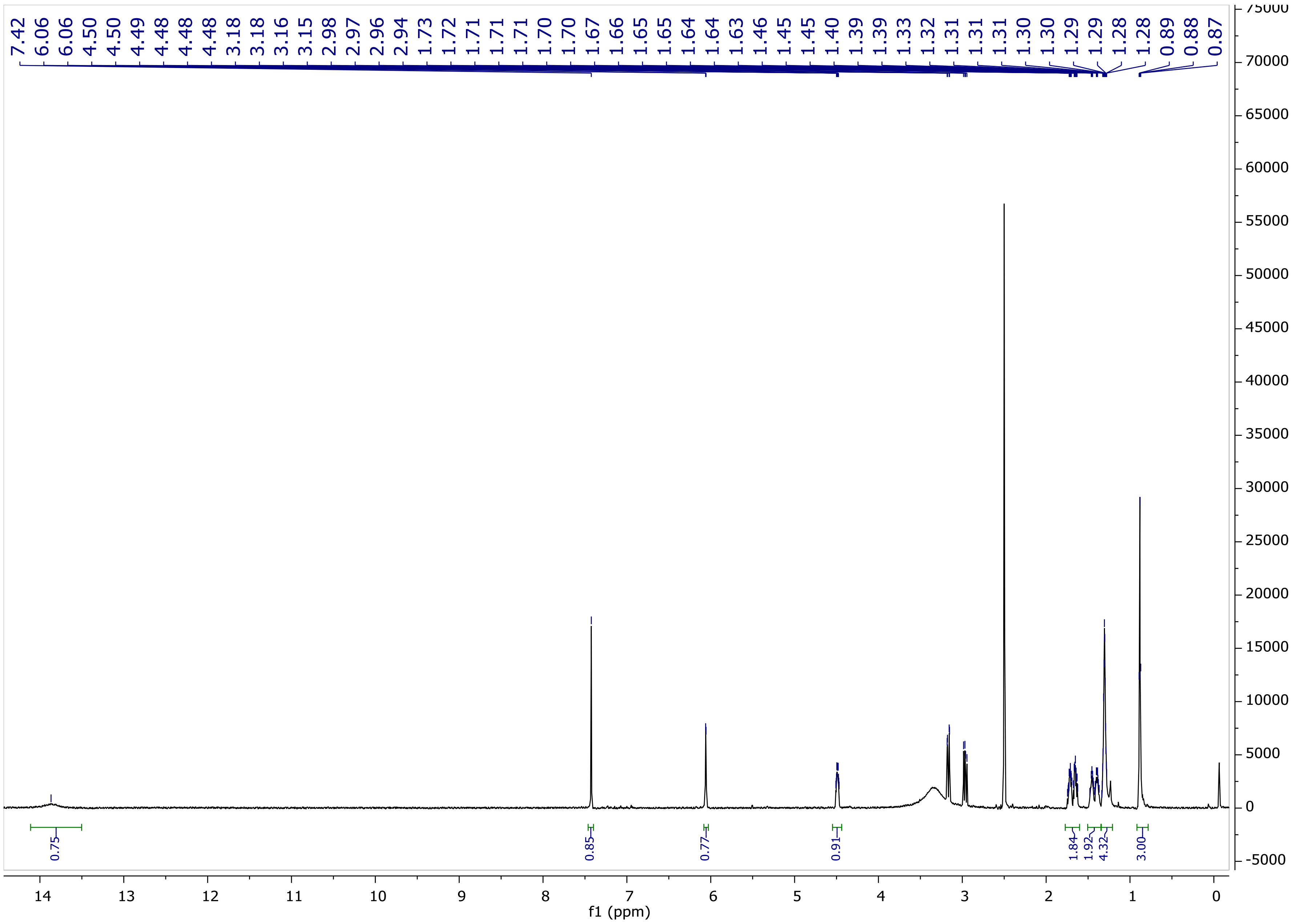

Figure S19. ^{1}H NMR spectrum of **3** in DMSO-d_6 at 700 MHz.

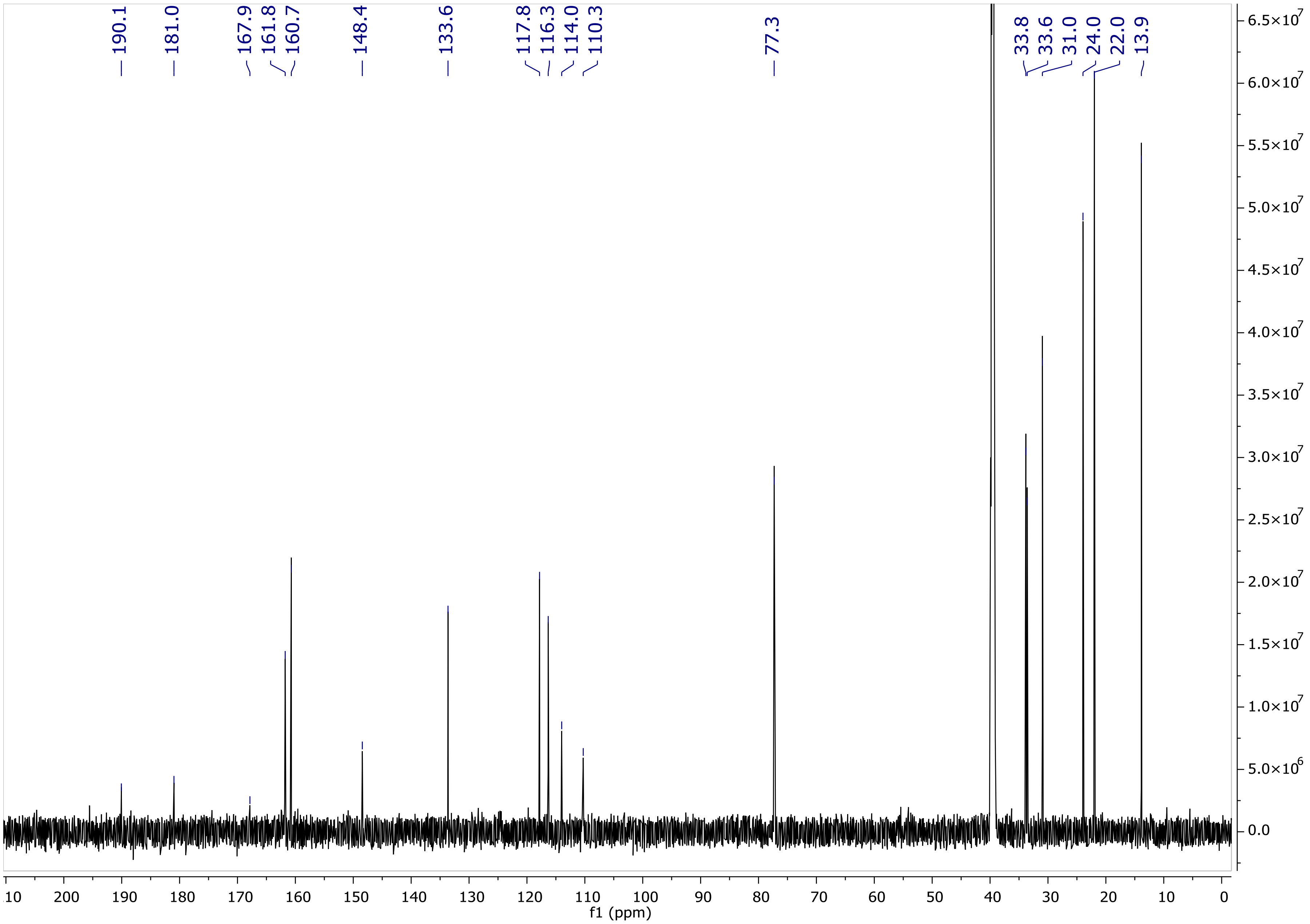

Figure S20. ^{1}H NMR spectrum of **3** in DMSO-d_6 at 175 MHz.

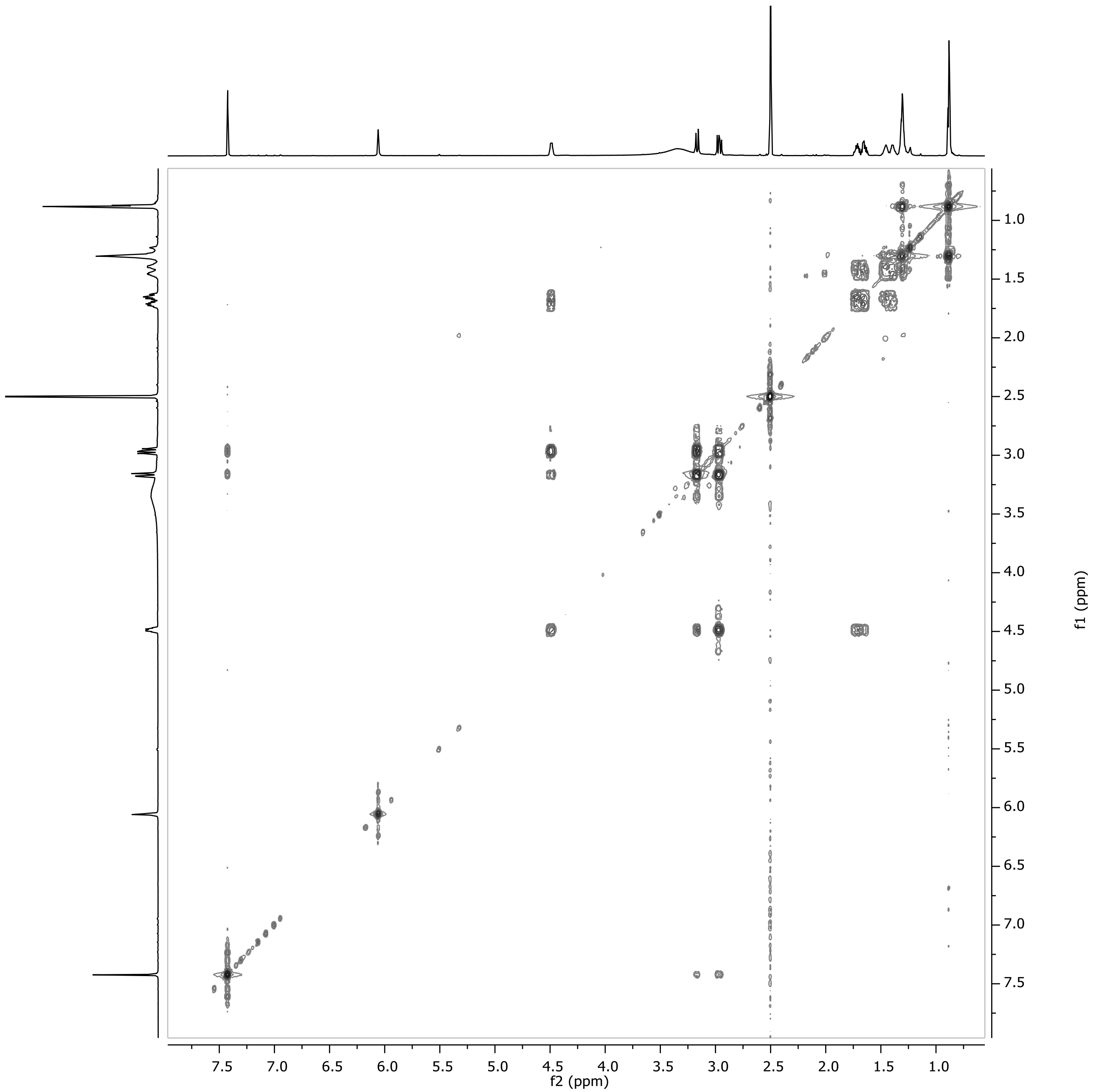

Figure S21. ^{1}H-^{1}H COSY spectrum of **3** in DMSO-d_6 at 700 MHz.

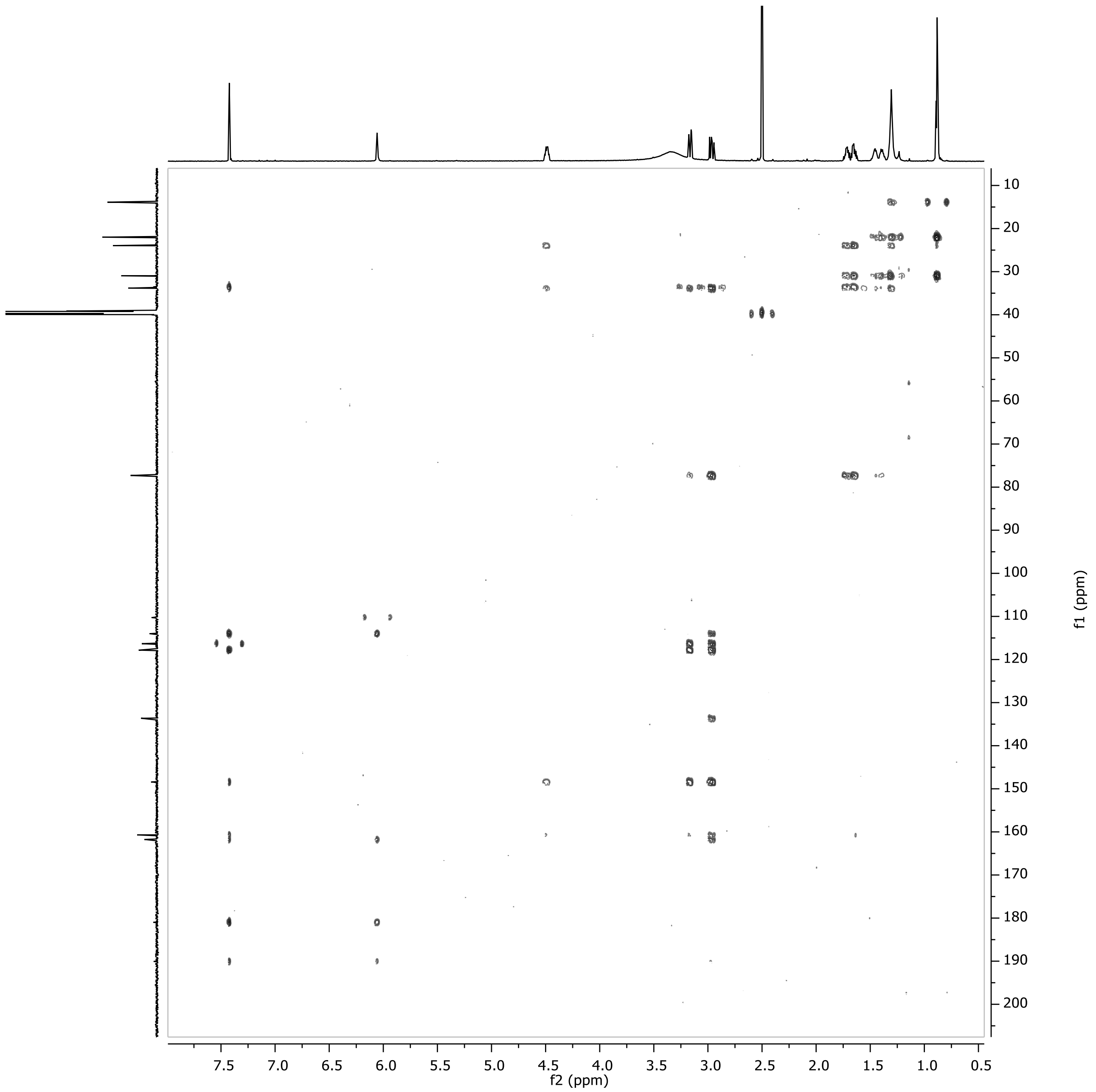

Figure S22. HMBC spectrum of **3** in DMSO-d_6 at 700 MHz.

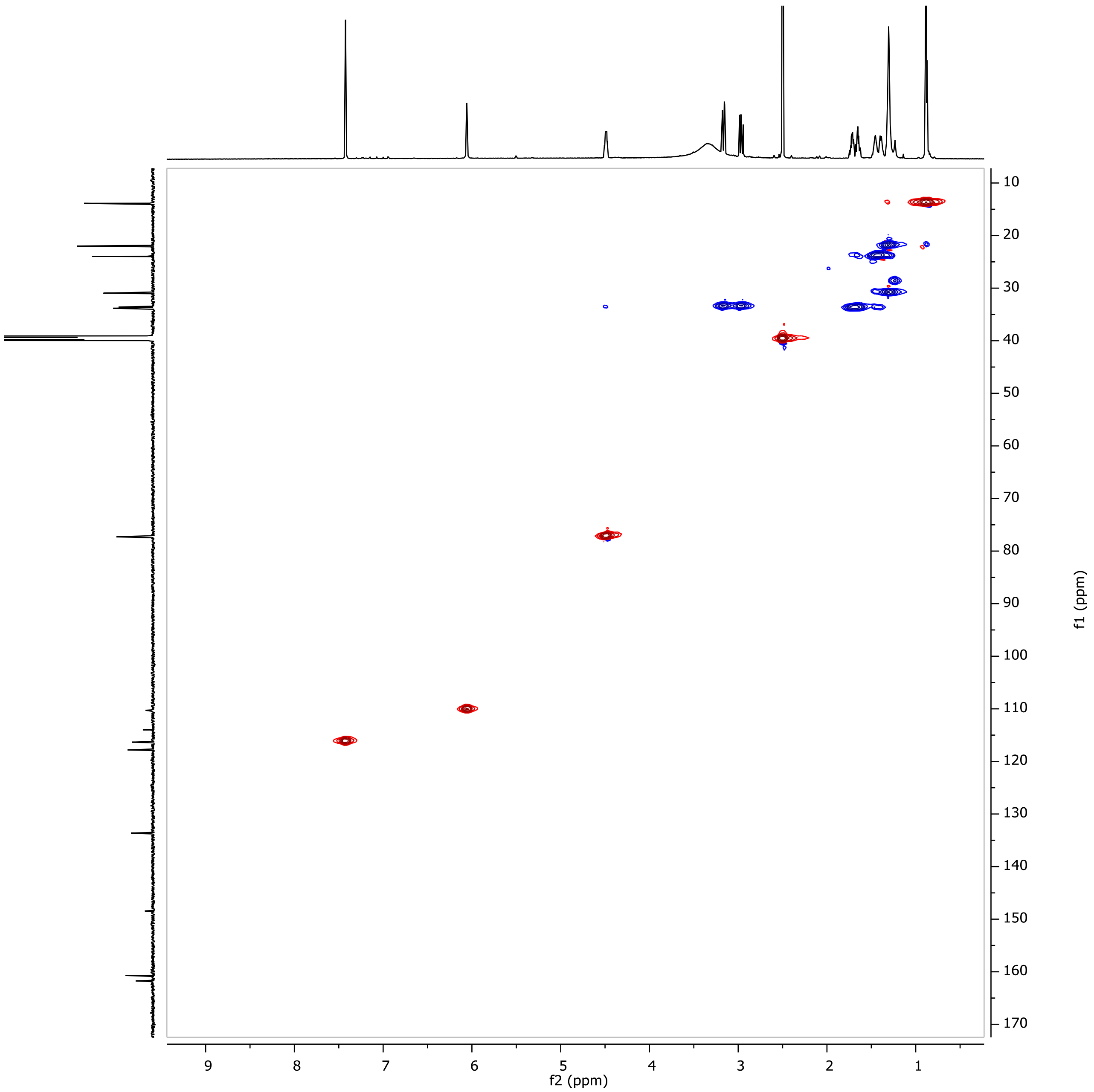

Figure S23. HSQC spectrum of **3** in DMSO-d_6 at 700 MHz.

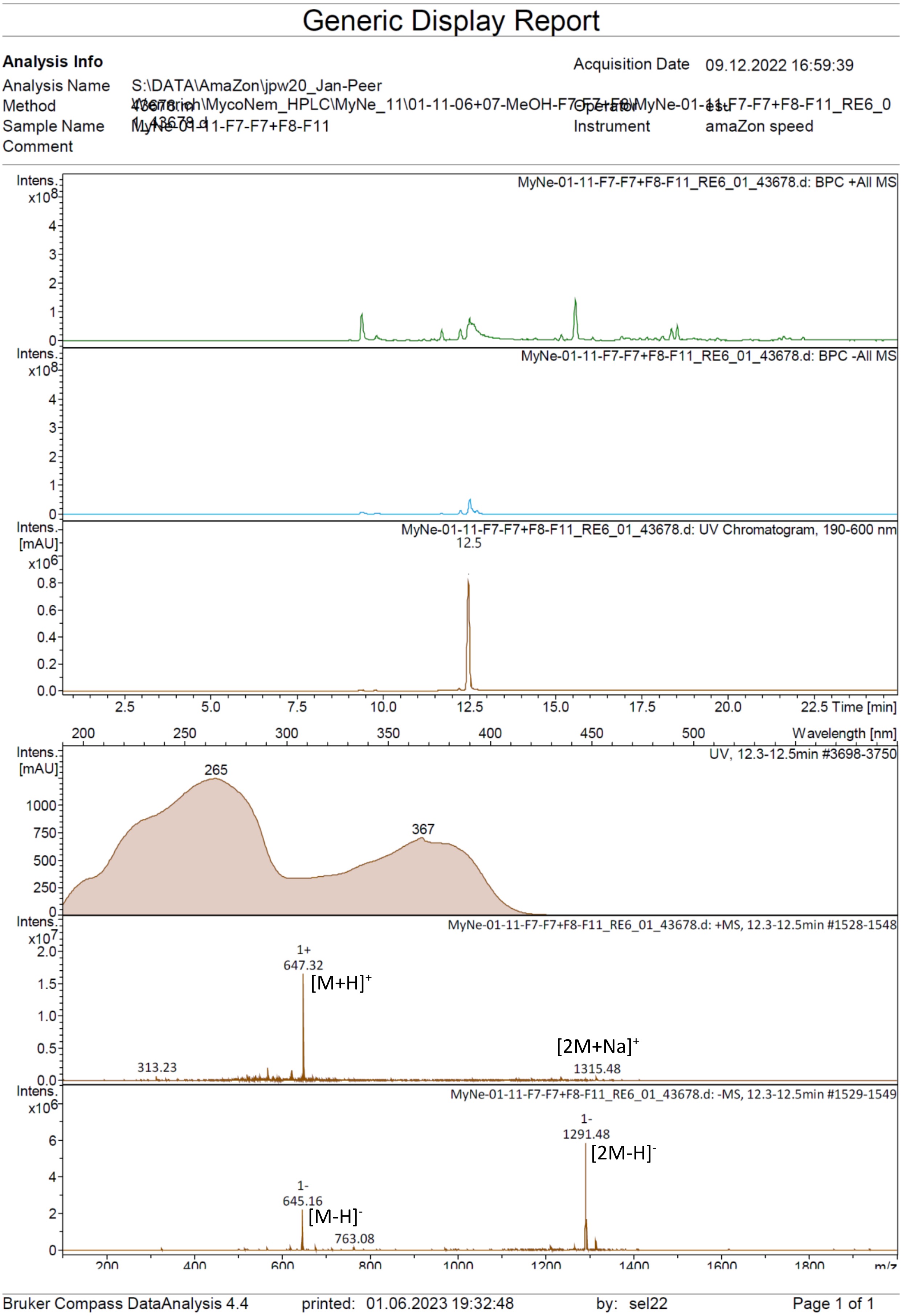

Figure S24. LRESIMS of **4**.

Generic Display Report

Analysis Info

		Acquisition Date	24.01.2023 14:44:33
Analysis Name	S:\DATA\MaXis\ESE22_Ellen Sepanian\23_01\03-11-03-M-MeOH-F7_11_01_11216.d		
Method	pos_säure_10000_screening_ms_100_2500_line.m	Operator	ate06
Sample Name	F7	Instrument	maXis
Comment	Screening01<br>Waters Acquity UPLC BEH C_18 1,7um 2.1x50mm		

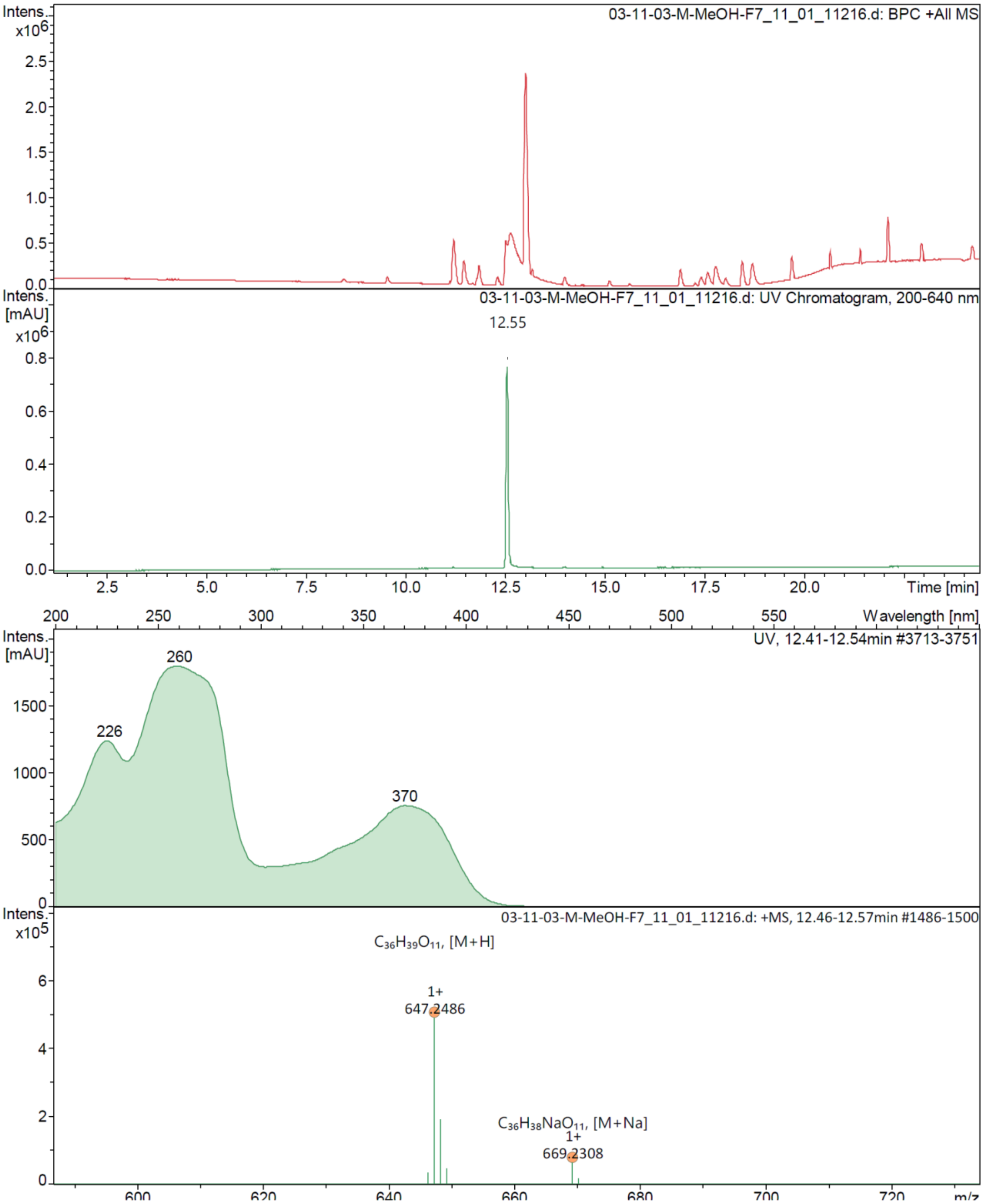

Bruker Compass DataAnalysis 4.4 printed: 01.06.2023 20:10:43 by: sel22 Page 1 of 1

Figure S25. HRESIMS of **4**.

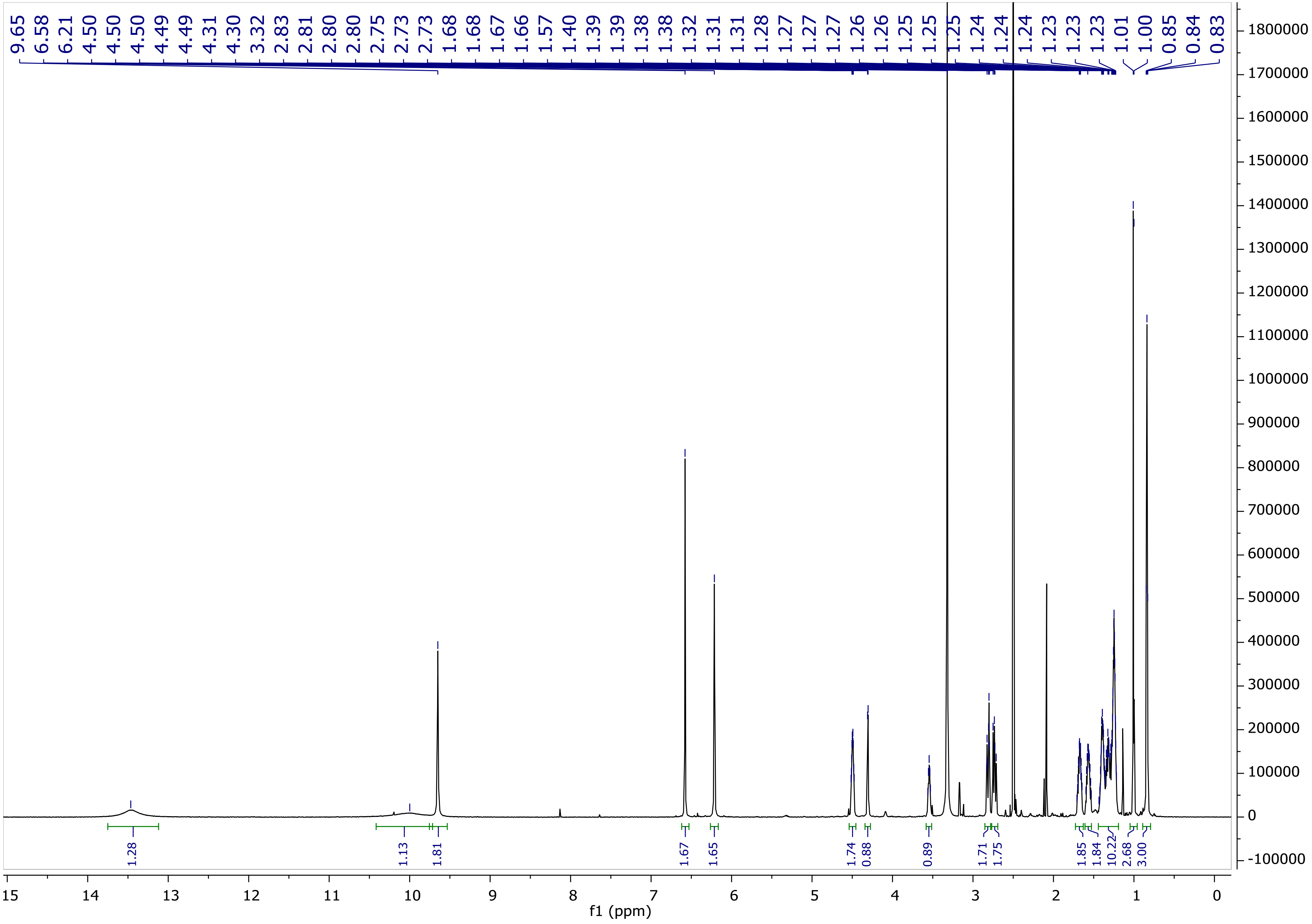

Figure S26. ^{1}H NMR spectrum of **4** in DMSO-d_6 at 700 MHz.

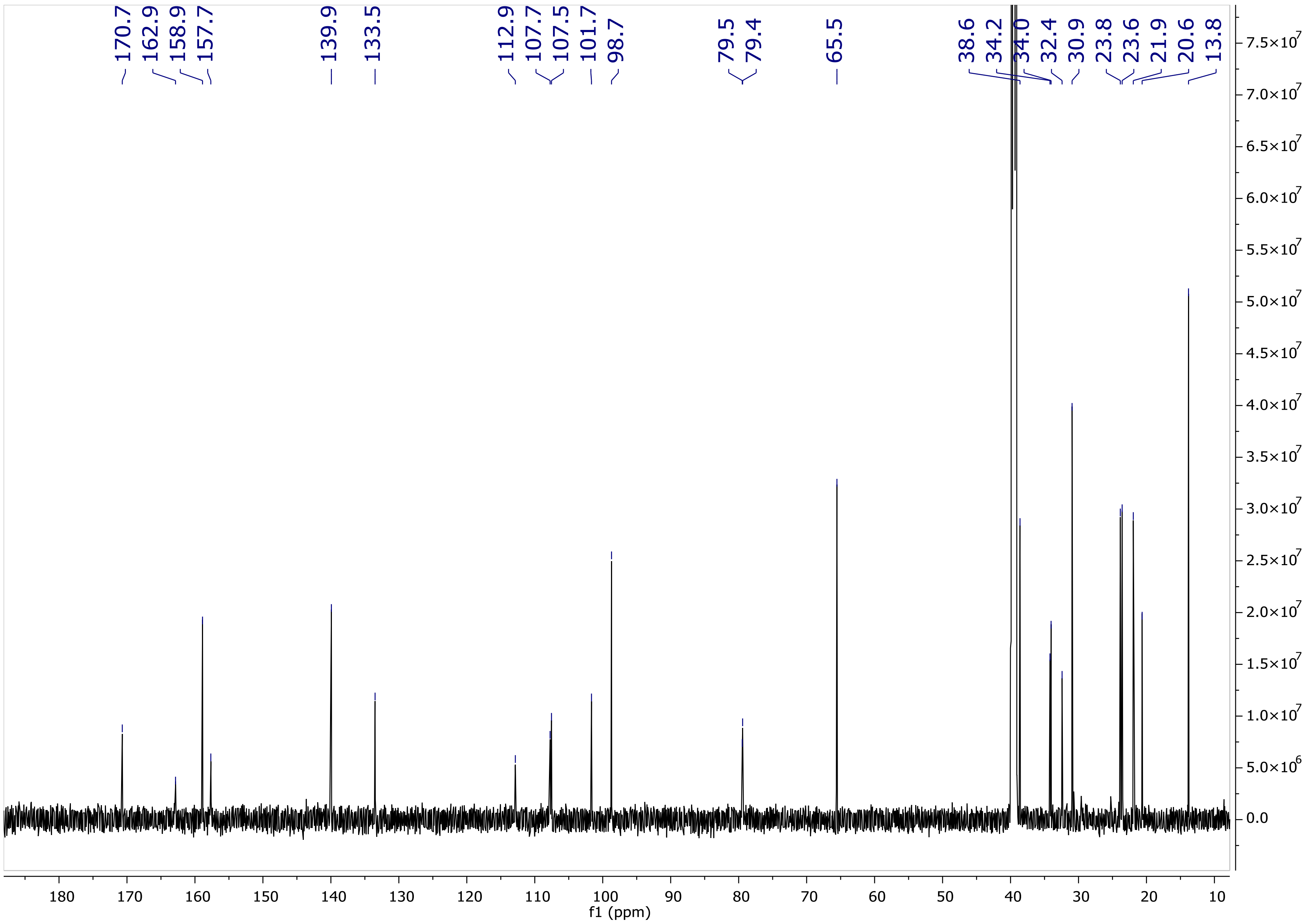

Figure S27. ^{1}H NMR spectrum of **4** in DMSO-d_6 at 175 MHz.

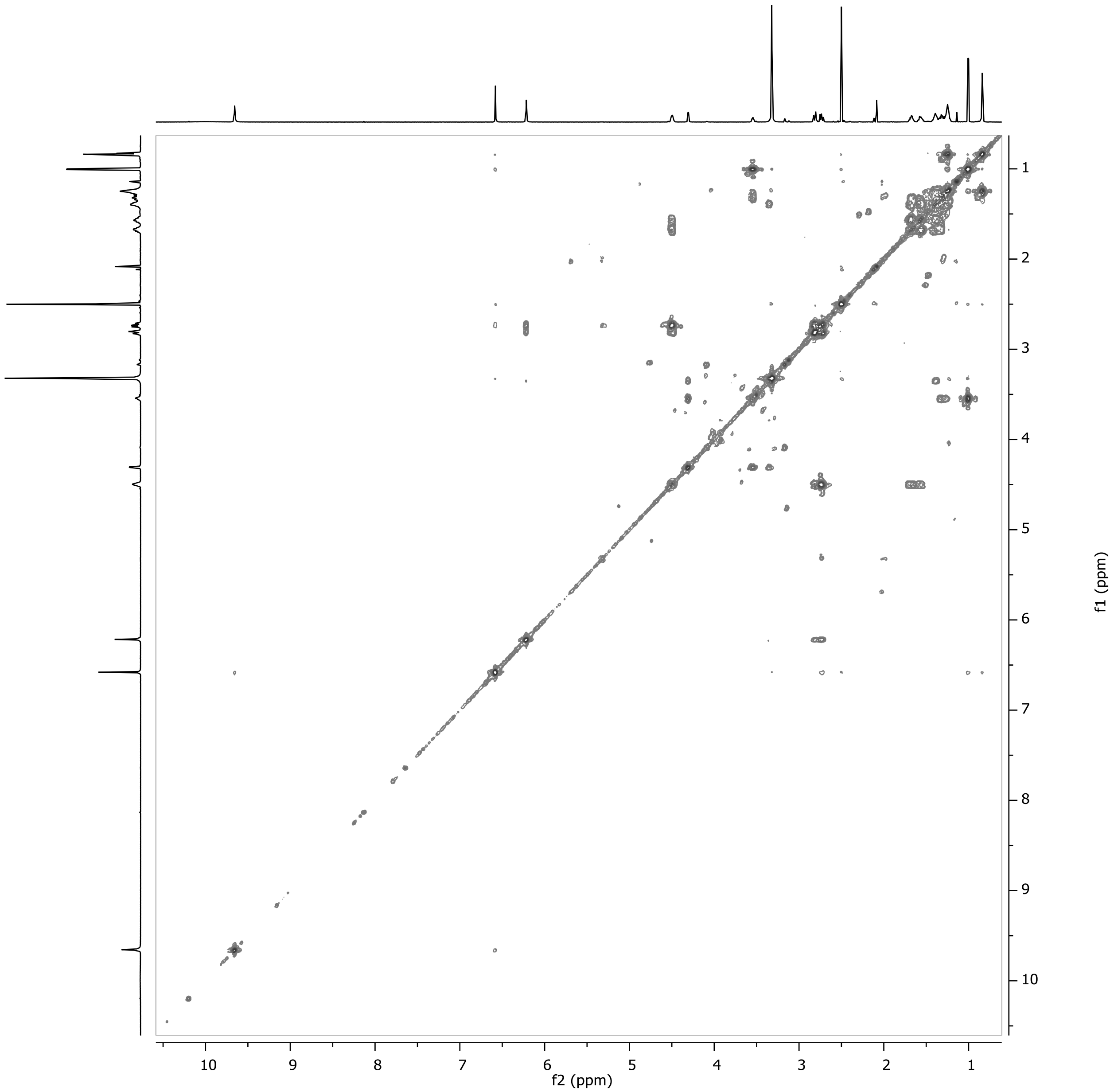

Figure S28. ^{1}H-^{1}H COSY spectrum of **4** in DMSO-d_6 at 700 MHz.

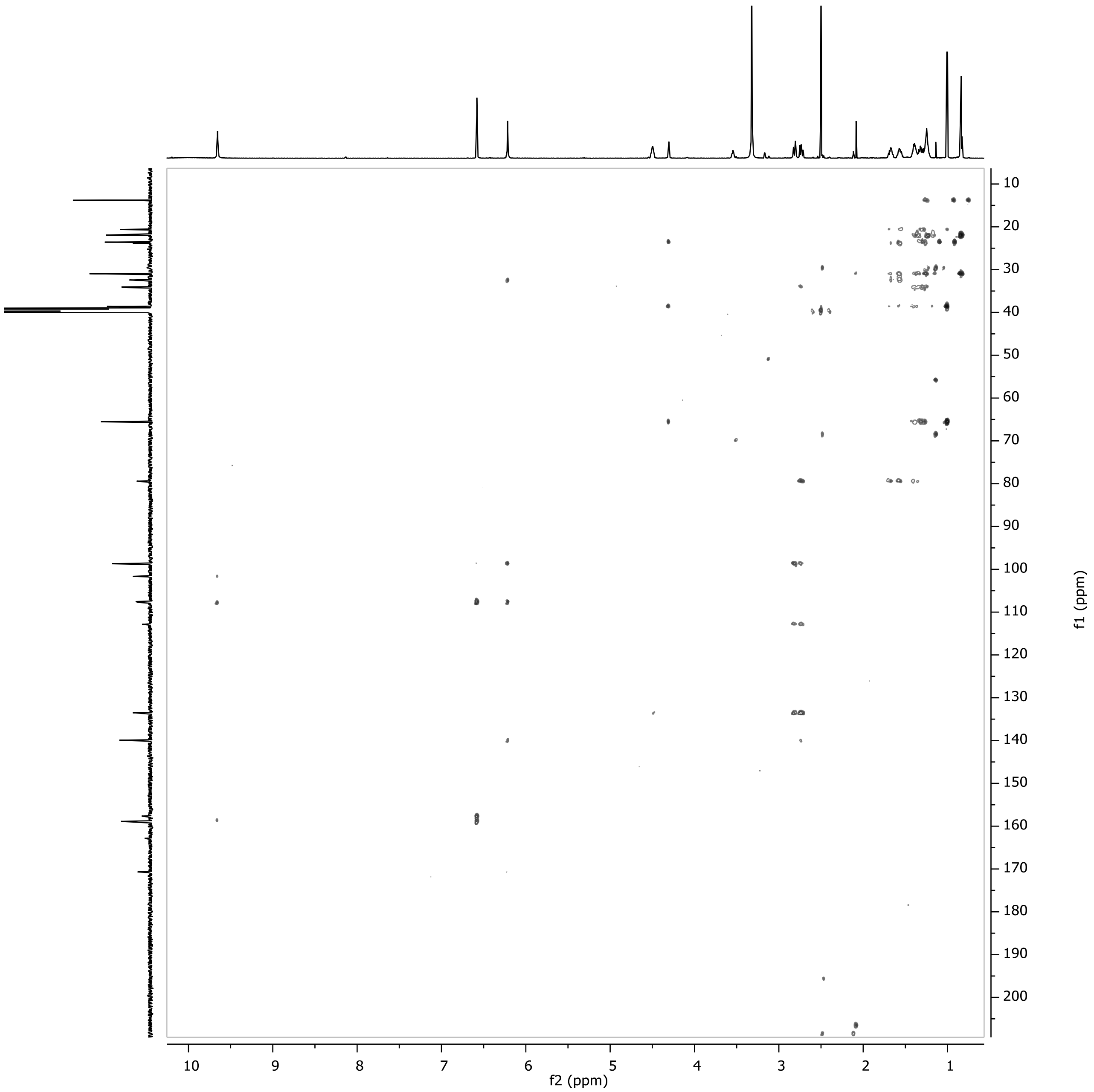

Figure S29. HMBC spectrum of **4** in DMSO-d_6 at 700 MHz.

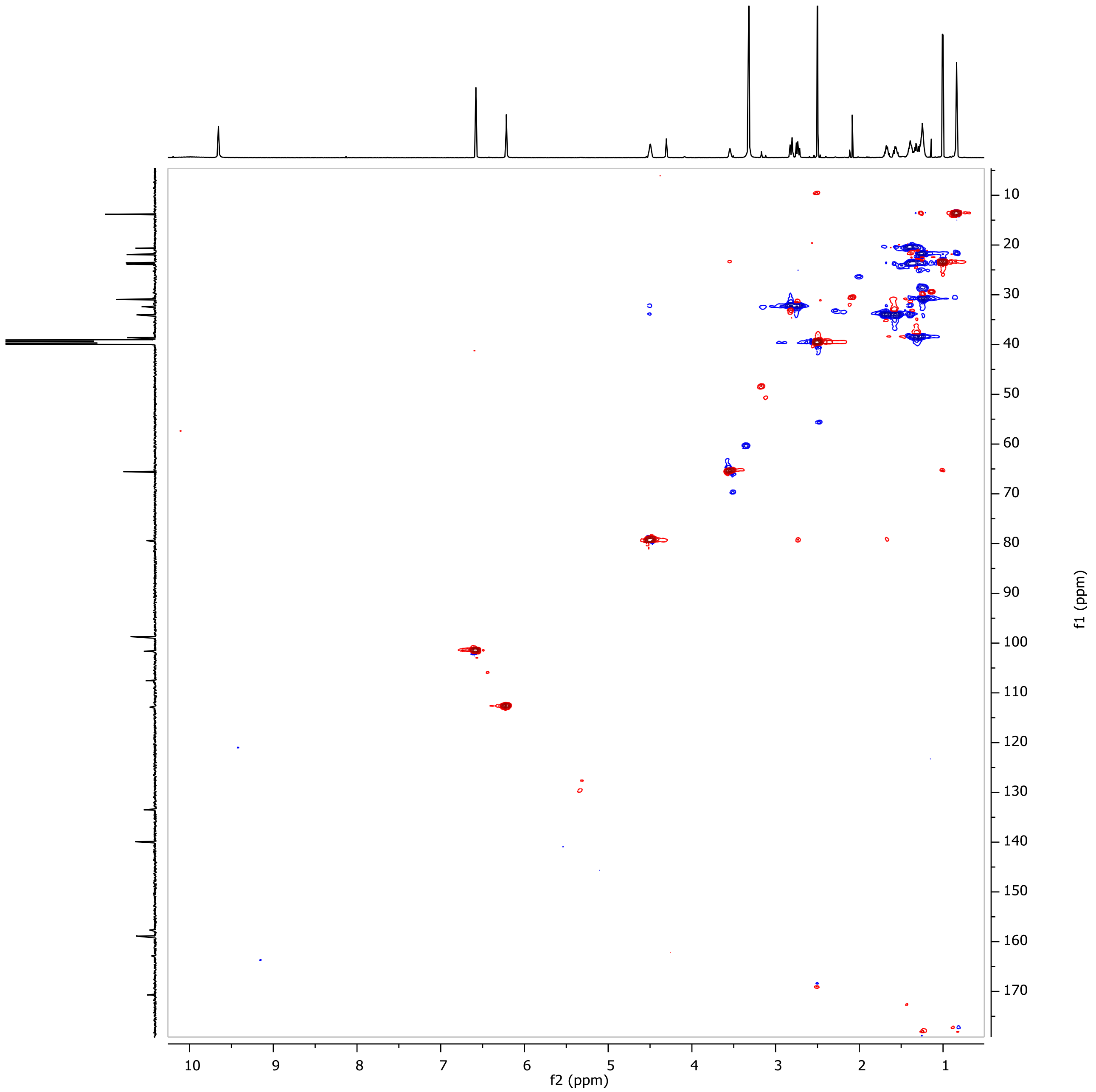

Figure S30. HSQC spectrum of **4** in DMSO-d_6 at 700 MHz.

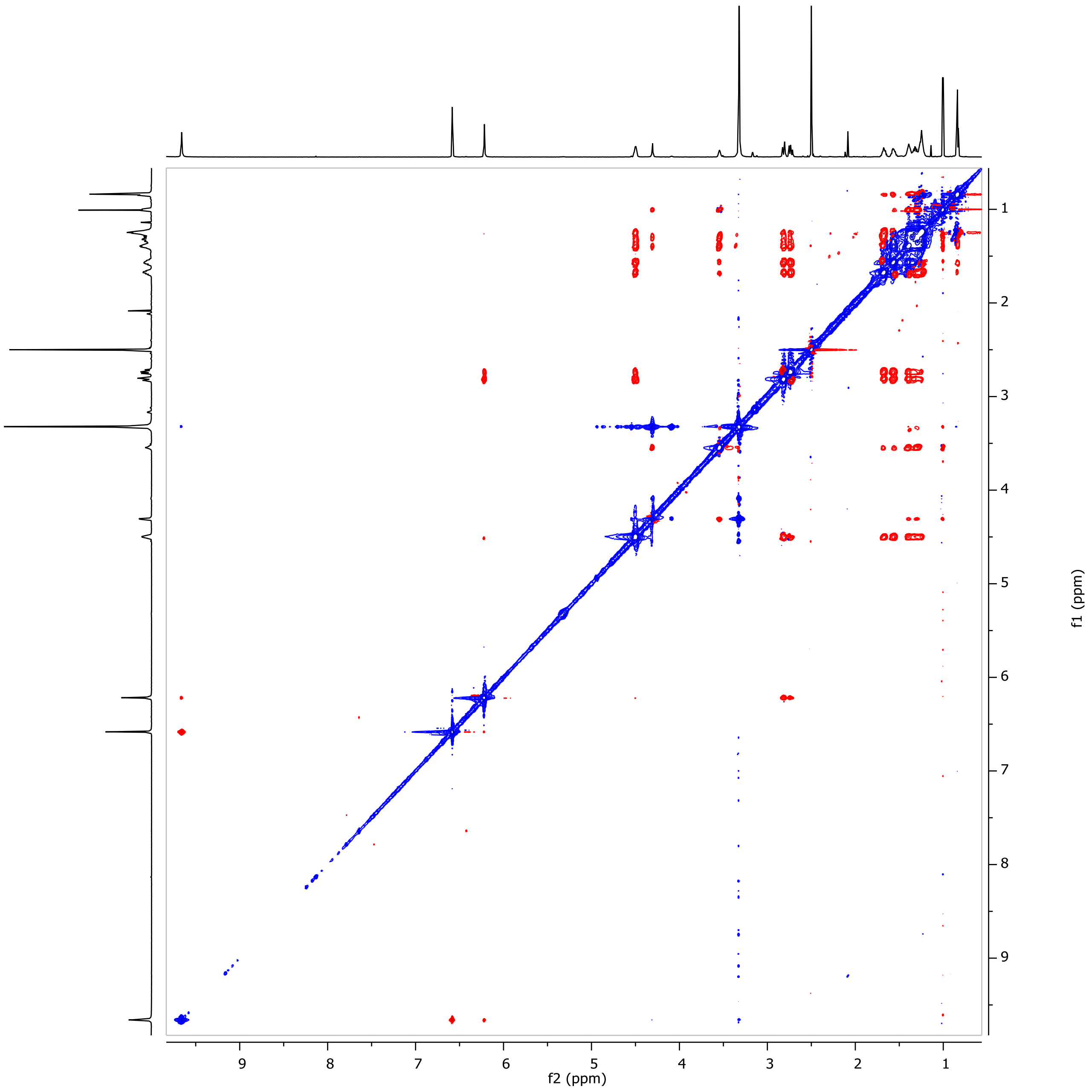

Figure S31. ROESY spectrum of **4** in DMSO-d_6 at 700 MHz.

Generic Display Report

Analysis Info

Analysis Name: S:\DATA\AmaZon\jpw20_Jan-Peer Wenrich\MycoNem_HPLC\MyNe_35\MyNe-01-35-06+07-MeOH-F5\MyNe-01-35-06+07-MeOH-F5-F15_GD4_01_43601.d

Method: 43601.m

Sample Name: MyNe-01-35-06+07-MeOH-F5-F15

Comment:

Acquisition Date: 12/7/2022 6:33:48 PM

Operator: esu

Instrument: amaZon speed

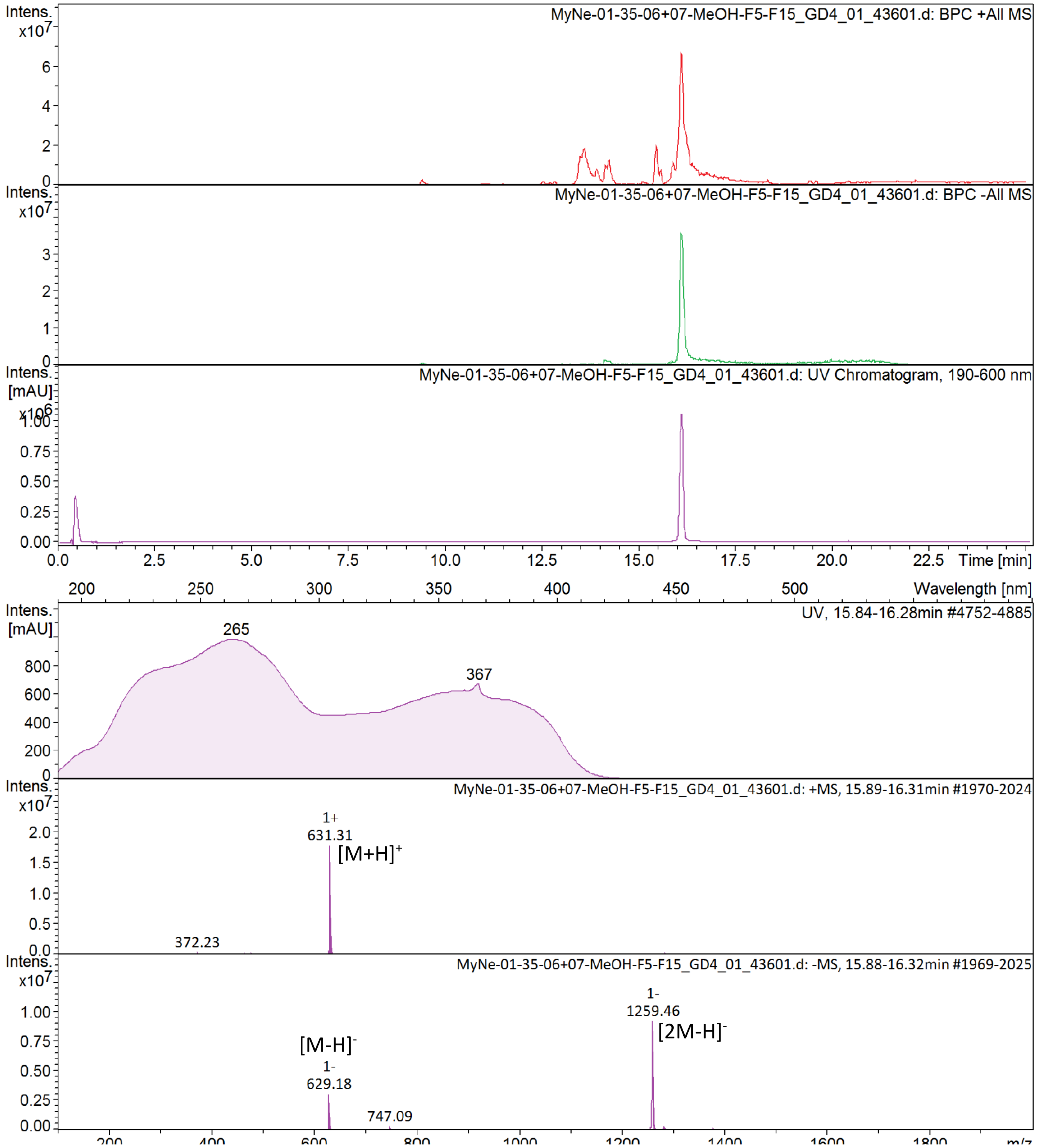

Bruker Compass DataAnalysis 4.4 printed: 1/10/2023 10:36:55 AM by: esu Page 1 of 1

Figure S32. LRESIMS of **5**.

Generic Display Report

Analysis Info | Acquisition Date 13.01.2023 11:39:14

Analysis Name	S:\DATA\MaXis\ESE22_Ellen Sepanian\23_01\F5-F15_21_01_11191.d
Method	pos_säure_10000_screening_ms_100_2500_line.m
Operator	ate06
Sample Name	F5-F15
Instrument	maXis
Comment	Screening01
	Waters Acquity UPLC BEH C_18 1,7um 2.1x50mm

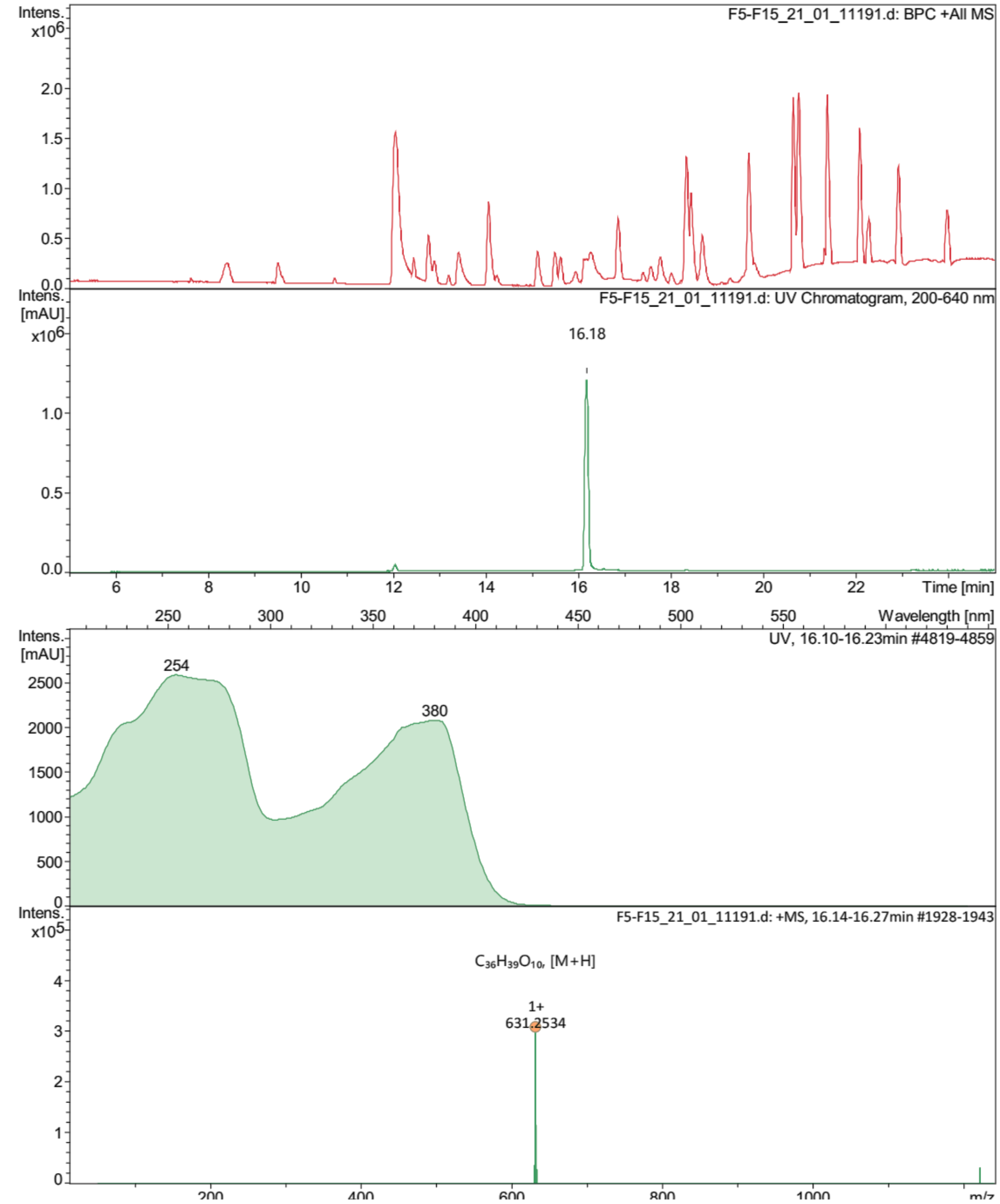

Bruker Compass DataAnalysis 4.4 printed: 11.06.2023 16:48:53 by: sel22 Page 1 of 1

Figure S33. HRESIMS of **5**.

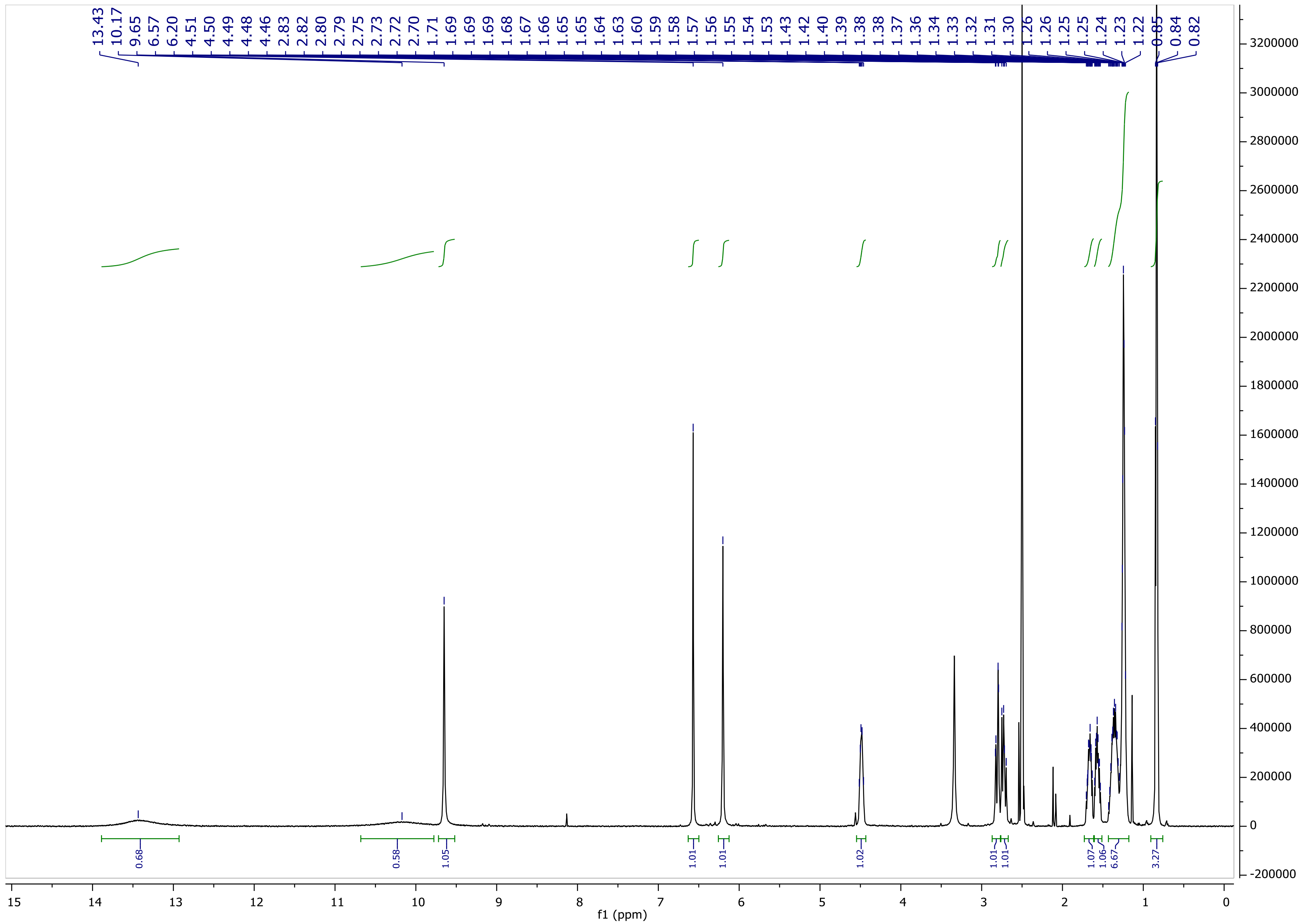

Figure S34. ^{1}H NMR spectrum of **5** in DMSO-d_6 at 500 MHz.

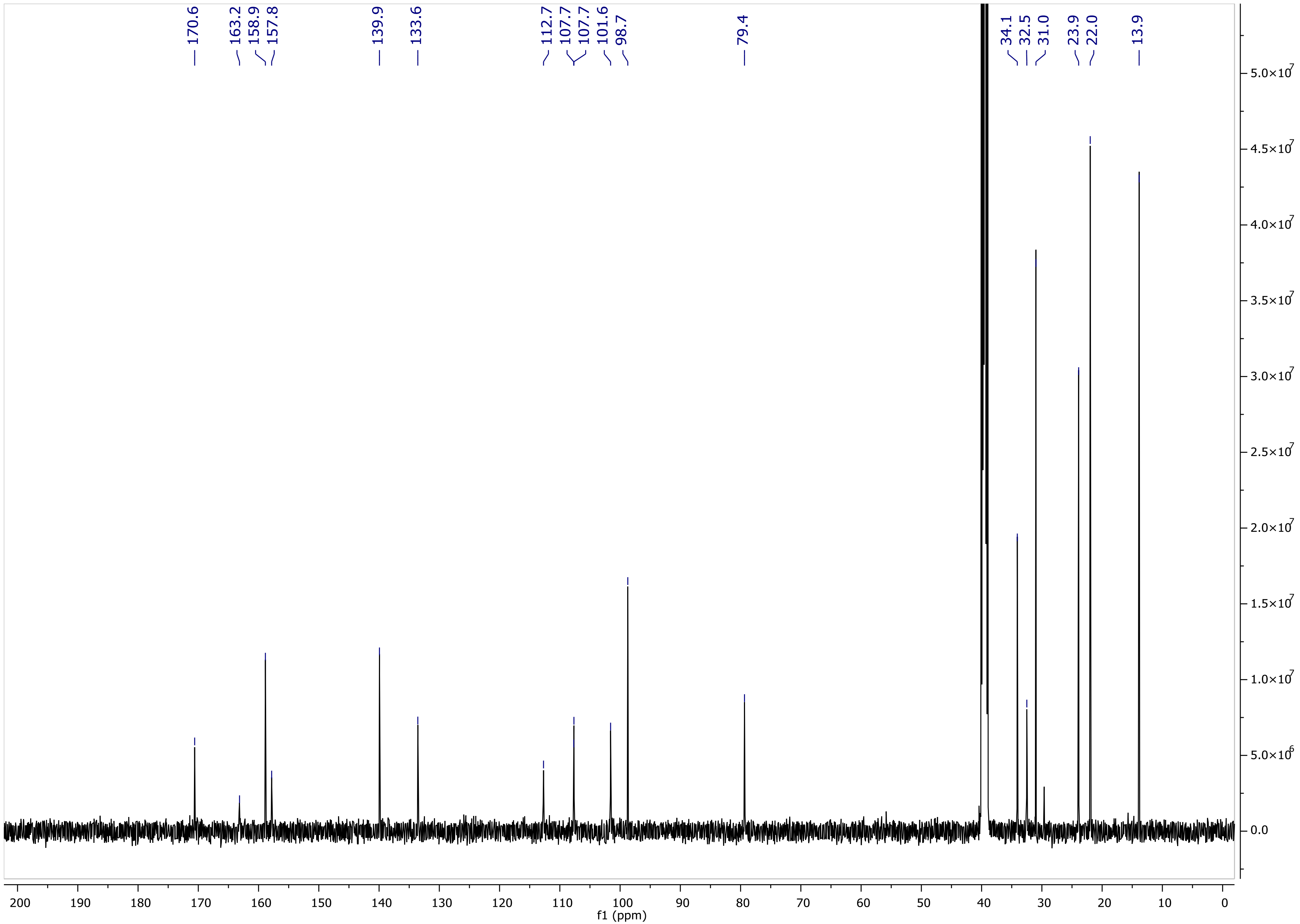

Figure S35. ^{13}C NMR spectrum of **5** in DMSO-d_6 at 125 MHz.

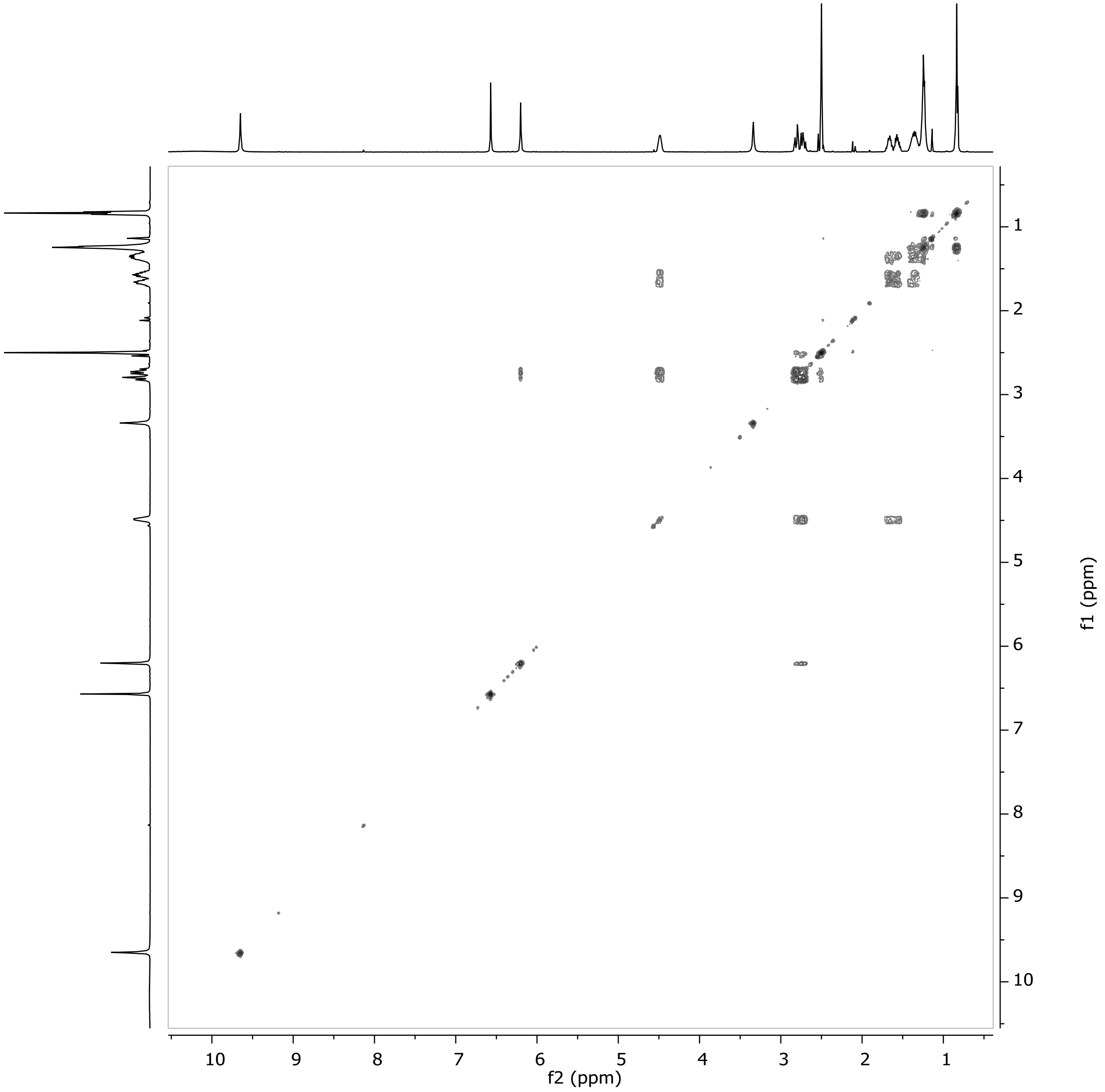

Figure S36. ^1H-^1H COSY spectrum of **5** in DMSO-d_6 at 500 MHz.

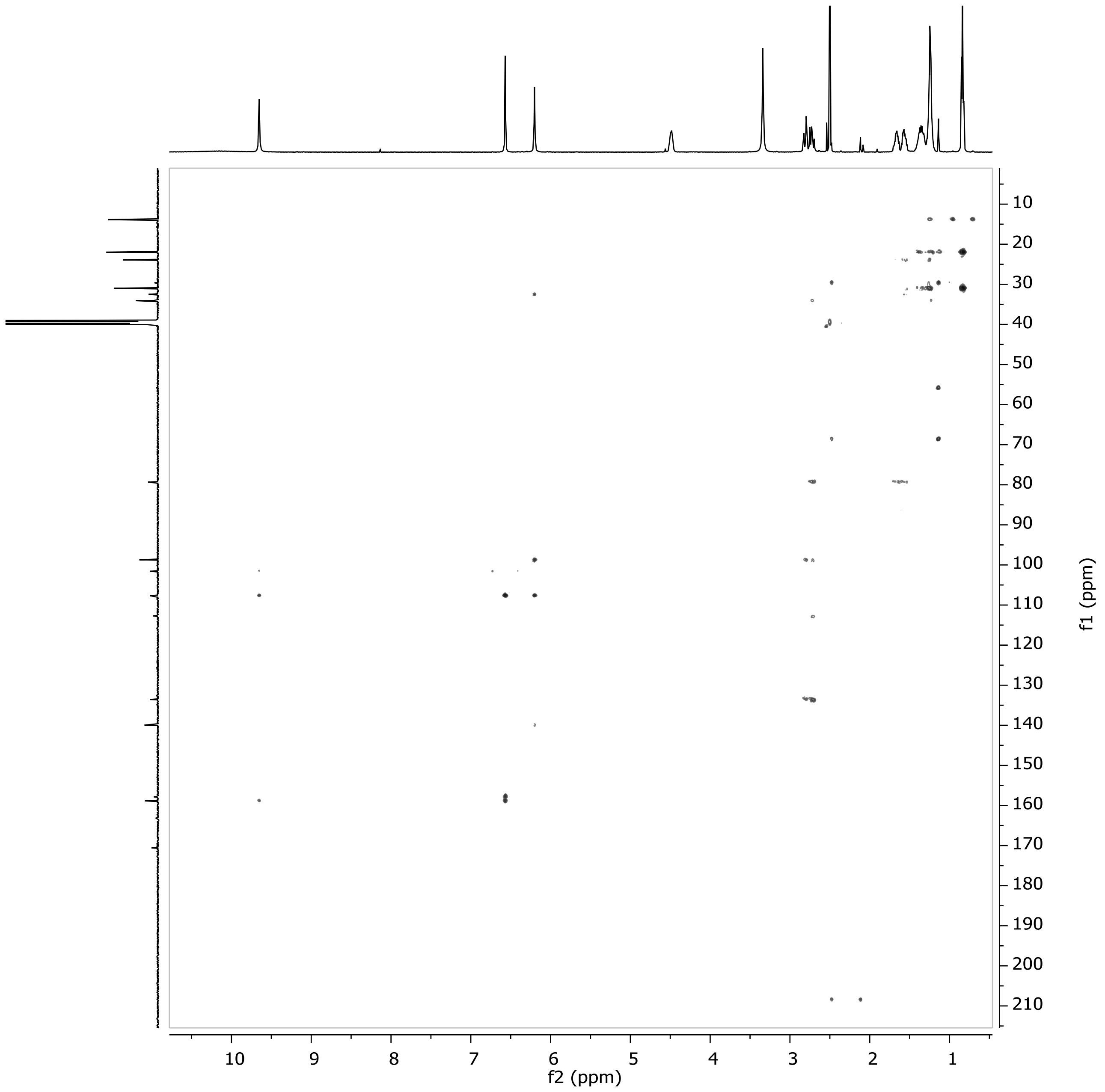

Figure S37. HMBC spectrum of **5** in DMSO-d_6 at 500 MHz.

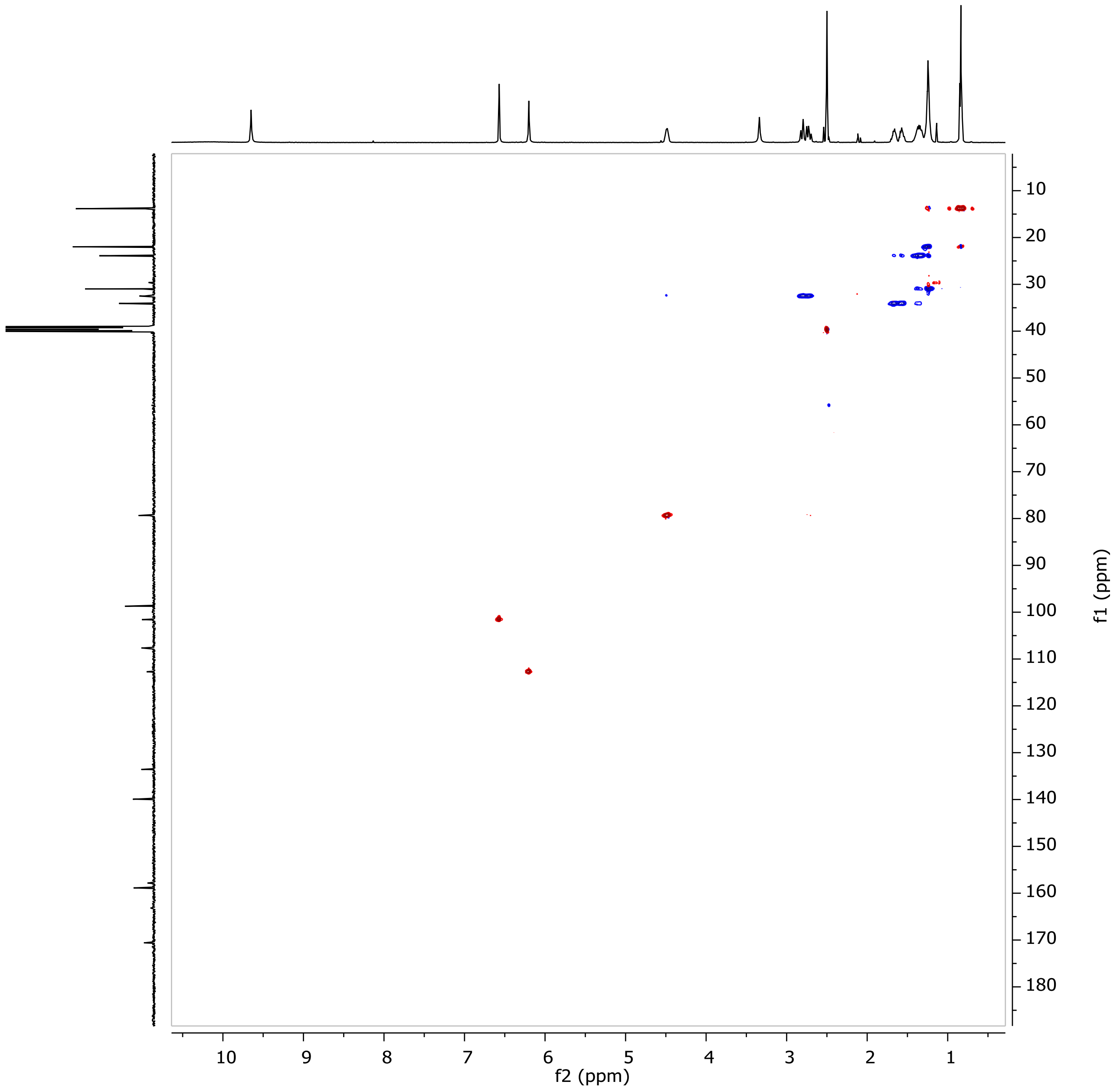

Figure S38. HSQC spectrum of **5** in DMSO-d_6 at 500 MHz.

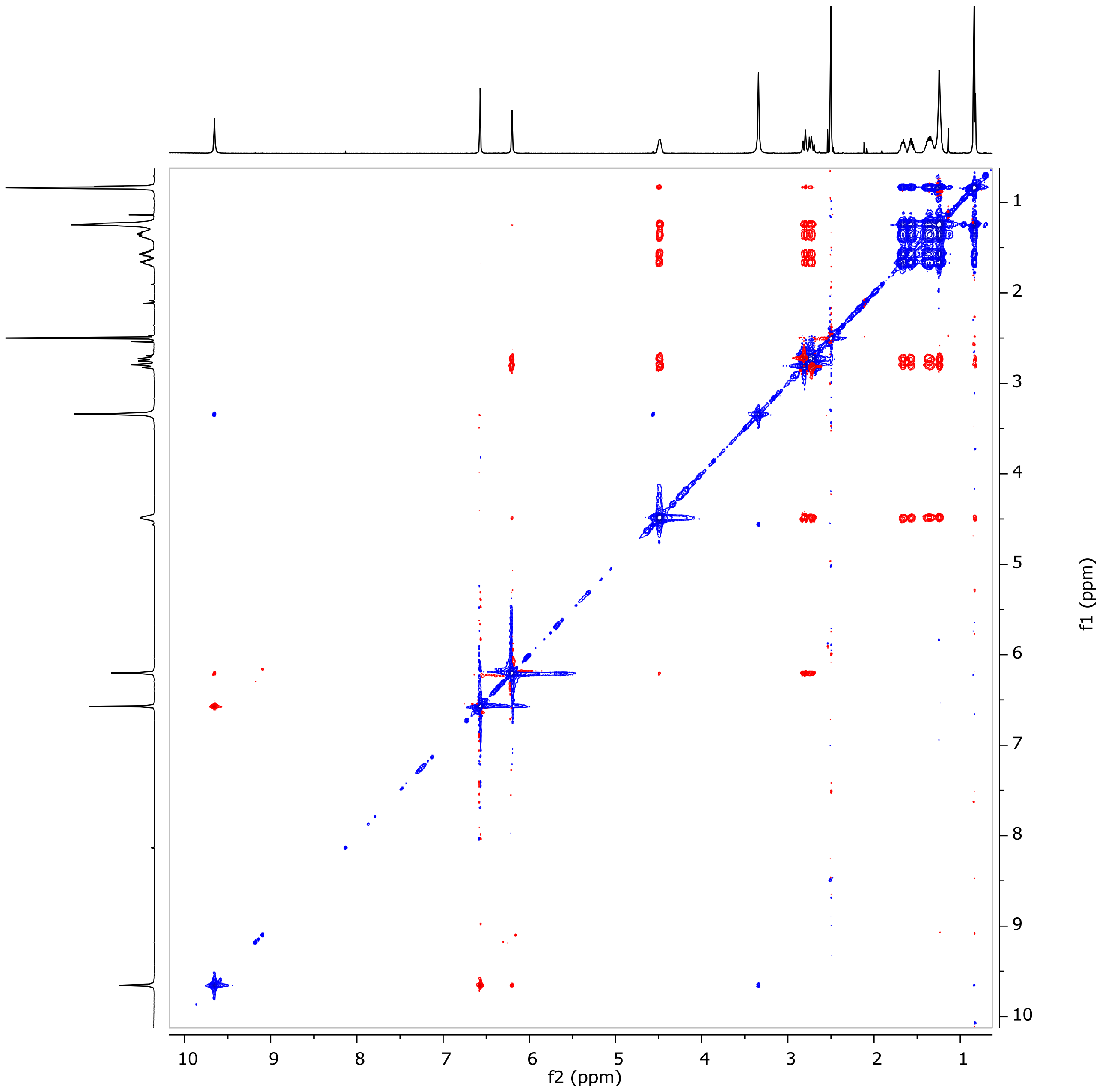

Figure S39. ROESY spectrum of **5** in DMSO-d_6 at 500 MHz.

Generic Display Report

Analysis Info

Analysis Name	S:\DATA\AmaZon\jpw20_Jan-Peer Wennrich\MycoNem_HPLC\MyNe_11\MyNe_01_11_06+07_MeOH_F9\MyNe-01-11-06+07-MeOH-F9-F2_RC2_01_42297.d	Acquisition Date	14.10.2022 19:38:17
Method	42297.m	Operator	esu
Sample Name	MyNe-01-11-06+07-MeOH-F9-F2	Instrument	amaZon speed
Comment			

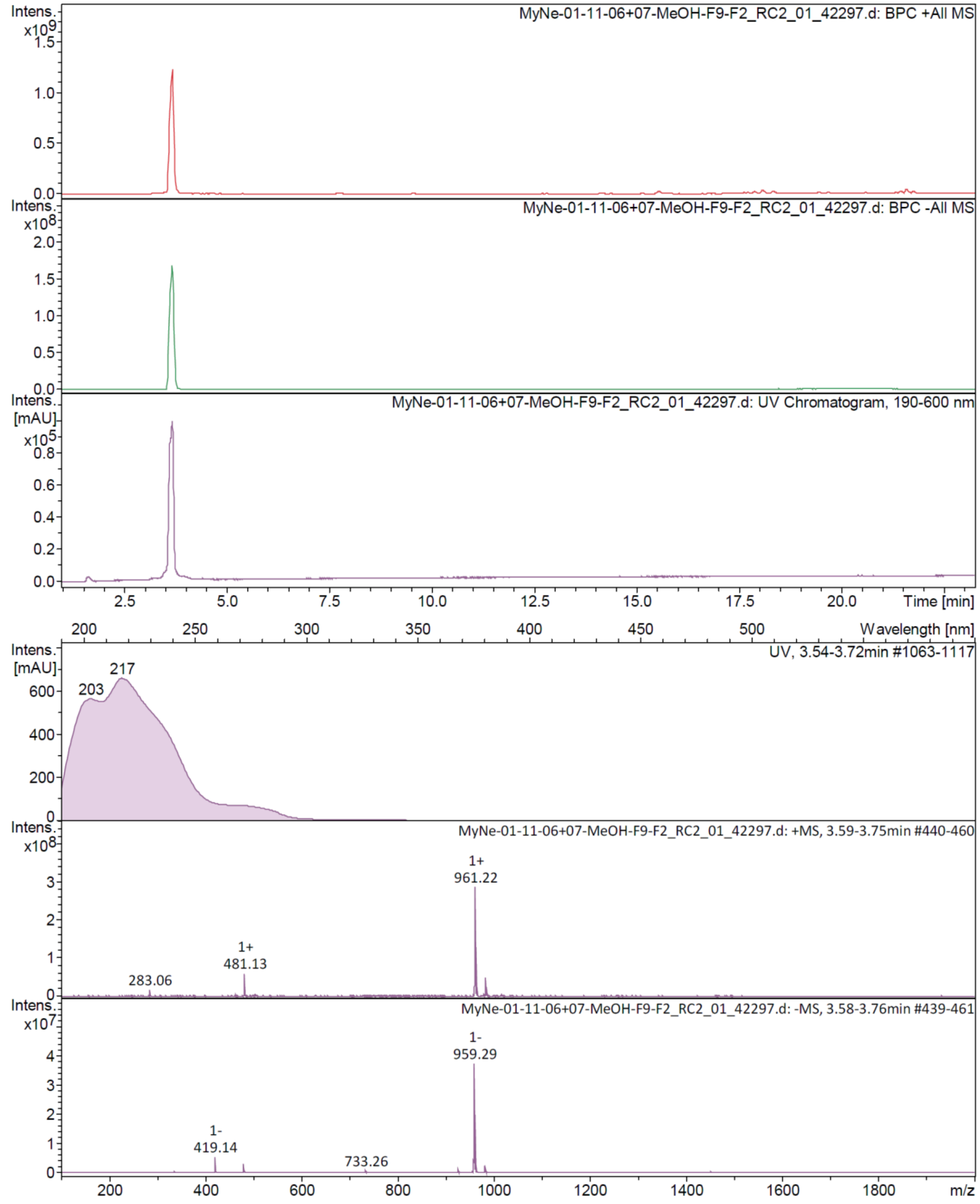

Bruker Compass DataAnalysis 4.4 printed: 08.11.2022 14:43:14 by: sel22 Page 1 of 1

Figure S40. LRESIMS of **6**.

Generic Display Report

Analysis Info

Analysis Name	S:\DATA\MaXis\ESE22_Ellen Sepanian\22_12\MyNe-01-11-06+07-MEOH-F9-F2_21_01_11172.d	Acquisition Date	13.12.2022 09:26:55
Method	pos_säure_10000_screening_ms_100_2500_line.m	Operator	ate06
Sample Name	MyNe-01-11-06+07-MEOH-F9-F2	Instrument	maXis
Comment	Screening01<br>Waters Acquity UPLC BEH C_18 1,7um 2.1x50mm		

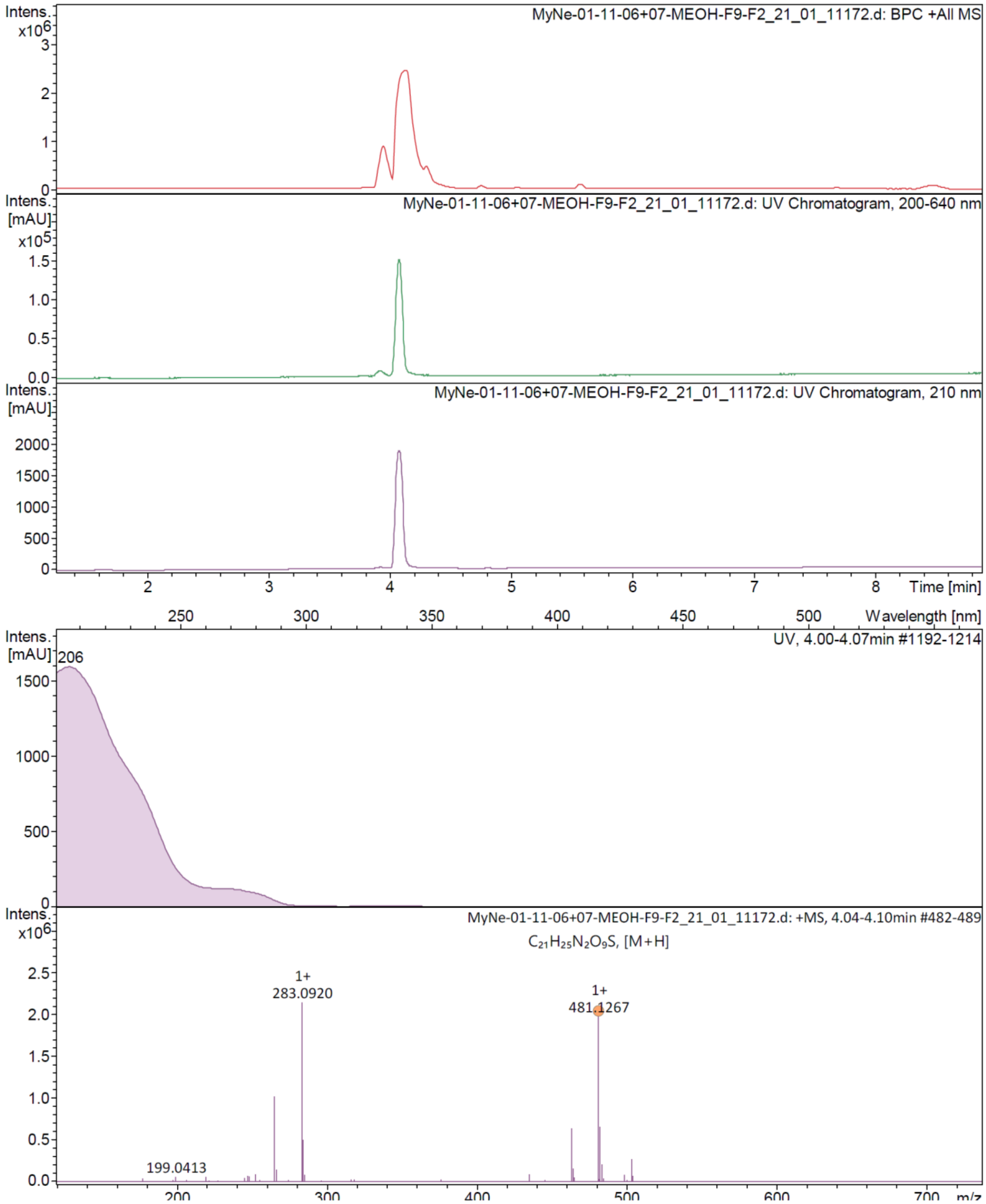

Bruker Compass DataAnalysis 4.4 printed: 16.12.2022 16:30:02 by: sel22 Page 1 of 1

Figure S41. HRESIMS of **6**.

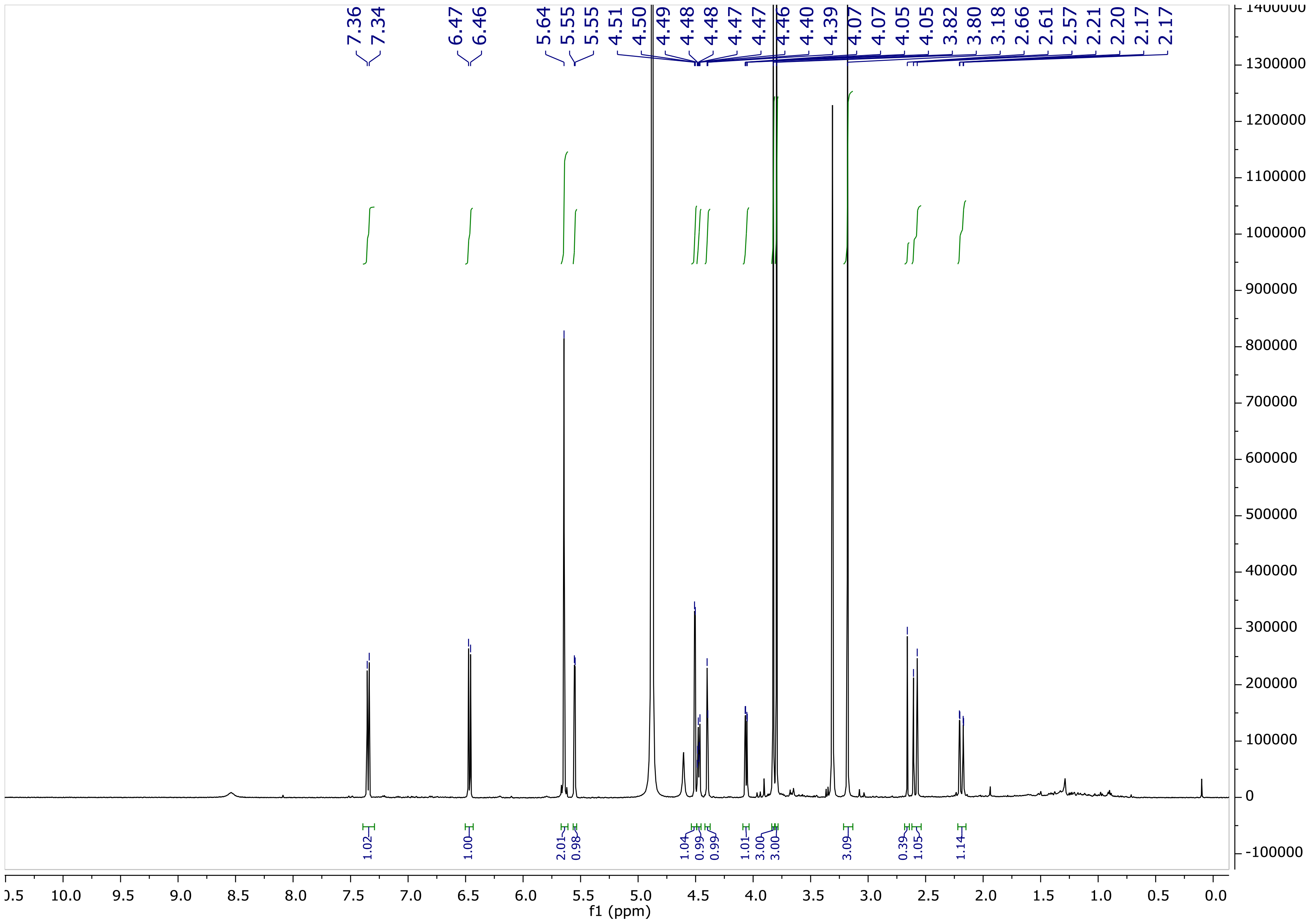

Figure S42. ^{1}H NMR spectrum of **6** in methanol-d_4 at 500 MHz.

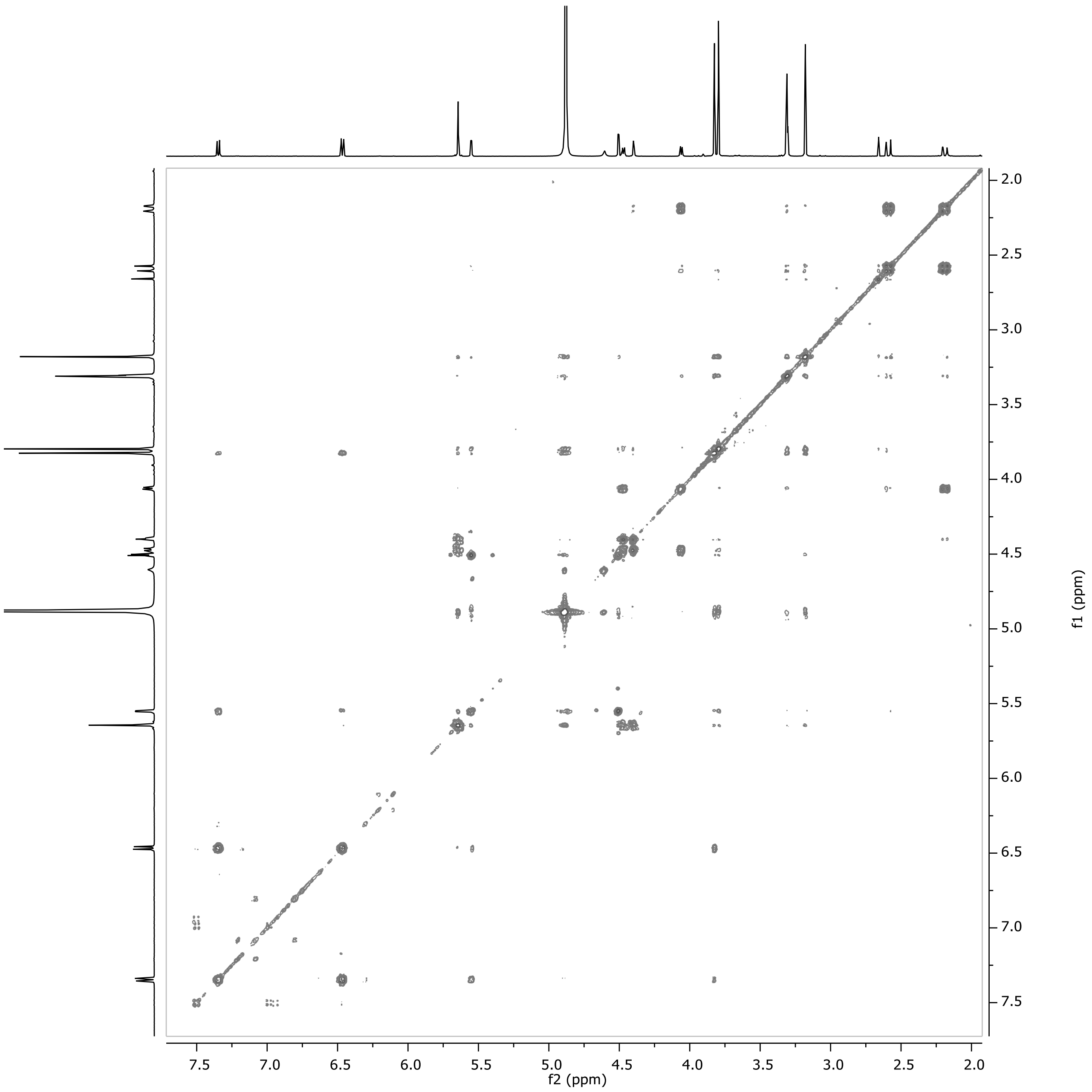

Figure S43. ^{1}H-^{1}H COSY spectrum of **6** in methanol-d_4 at 500 MHz.

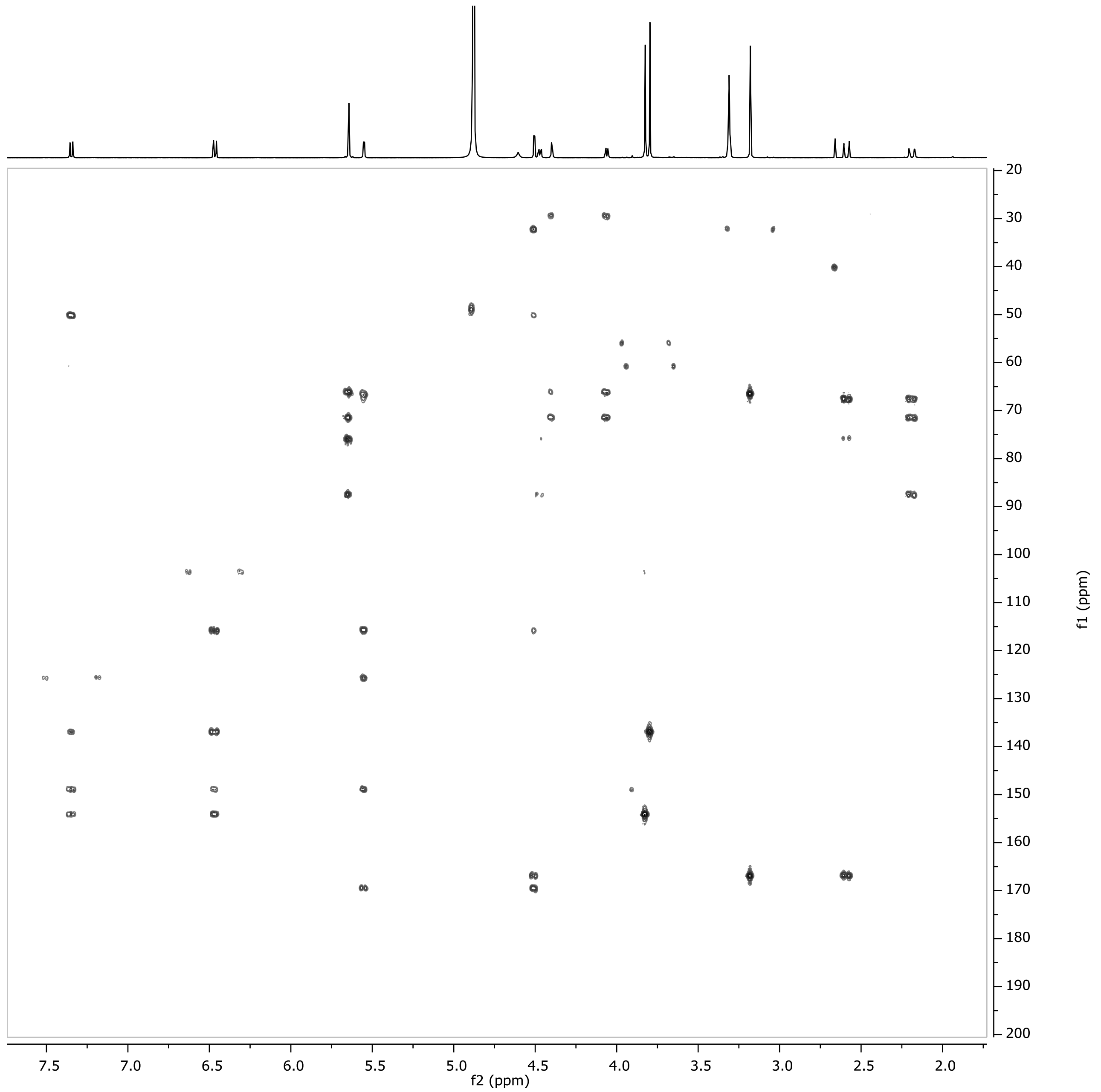

Figure S44. HMBC spectrum of **6** in methanol-d_4 at 500 MHz.

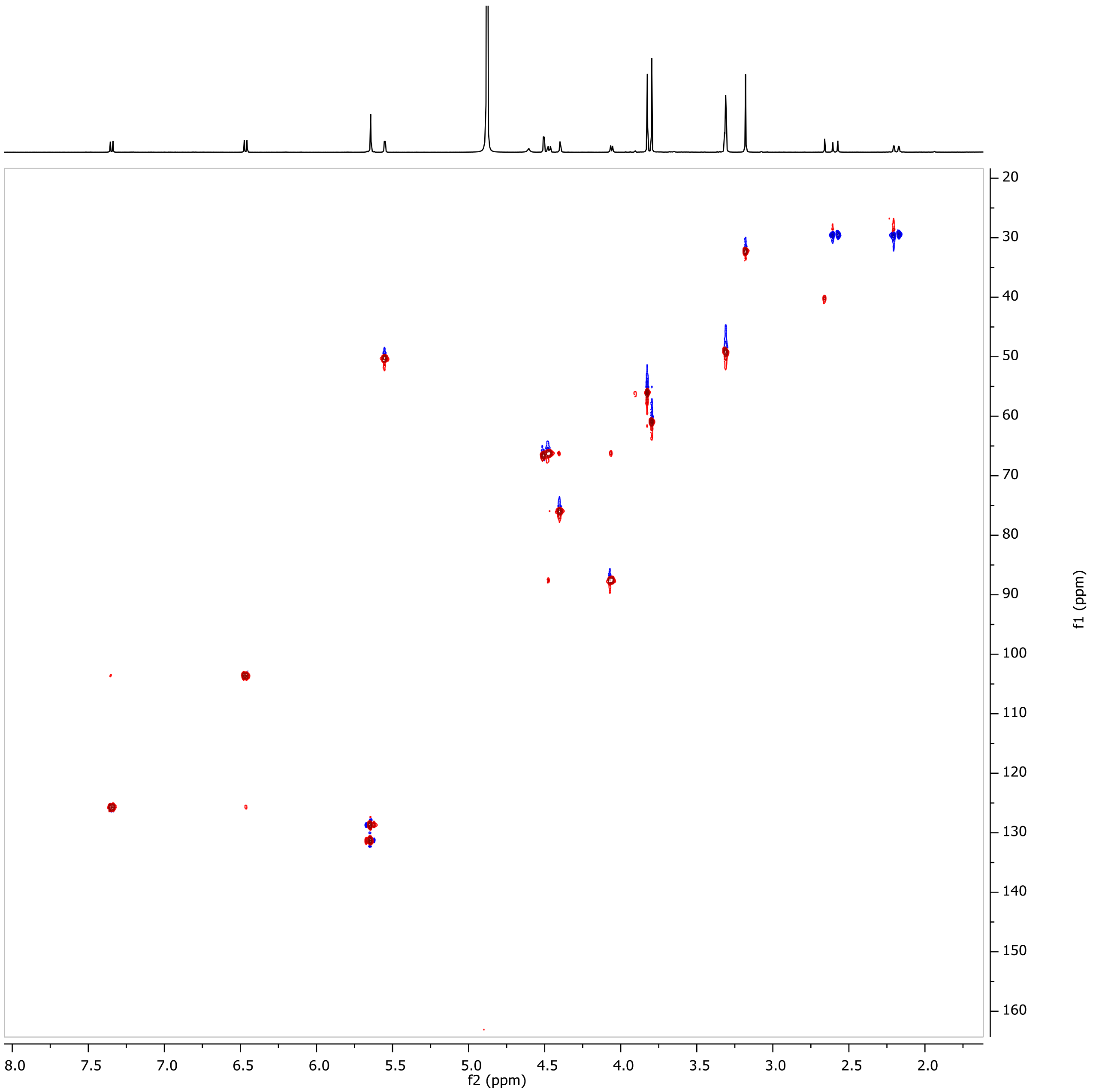

Figure S45. HSQC spectrum of **6** in methanol-d_4 at 500 MHz.

Generic Display Report

Analysis Info

Analysis Name	S:\DATA\AmaZon\jpw20_Jan-Peer Wennrich\MycoNem_HPLC\MyNe_09\F6\MyNe-01-09-06+07-MeOH-F6-F3_RB3_01_42271.d	Acquisition Date	14.10.2022 02:15:22
Method	42271.m	Operator	esu
Sample Name	MyNe-01-09-06+07-MeOH-F6-F3	Instrument	amaZon speed
Comment			

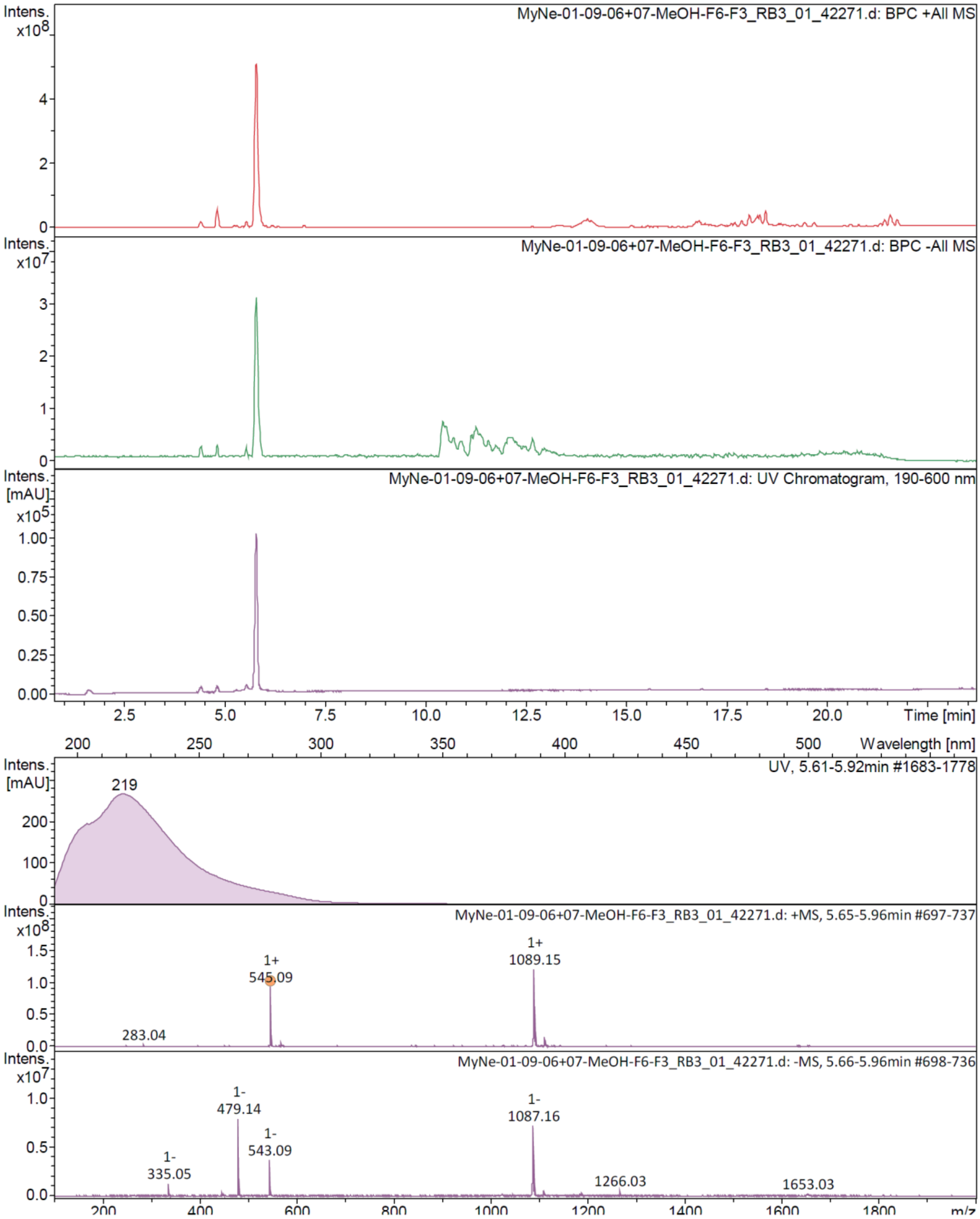

Bruker Compass DataAnalysis 4.4 printed: 15.11.2022 13:44:32 by: sel22 Page 1 of 1

Figure S46. LRESIMS of **7**.

Generic Display Report

Analysis Info

		Acquisition Date	24.11.2022 12:30:42
Analysis Name	S:\DATA\MaXis\ESE22_Ellen Sepanian\22_11\MyNe-01-09-06+07-MeOH-F6-F3_71_01_11102.d		
Method	pos_säure_10000_screening_ms_100_2500_line.m	Operator	ate06
Sample Name	MyNe-01-09-06+07-MeOH-F6-F3	Instrument	maXis
Comment	Screening01<br>Waters Acquity UPLC BEH C_18 1,7um 2.1x50mm		

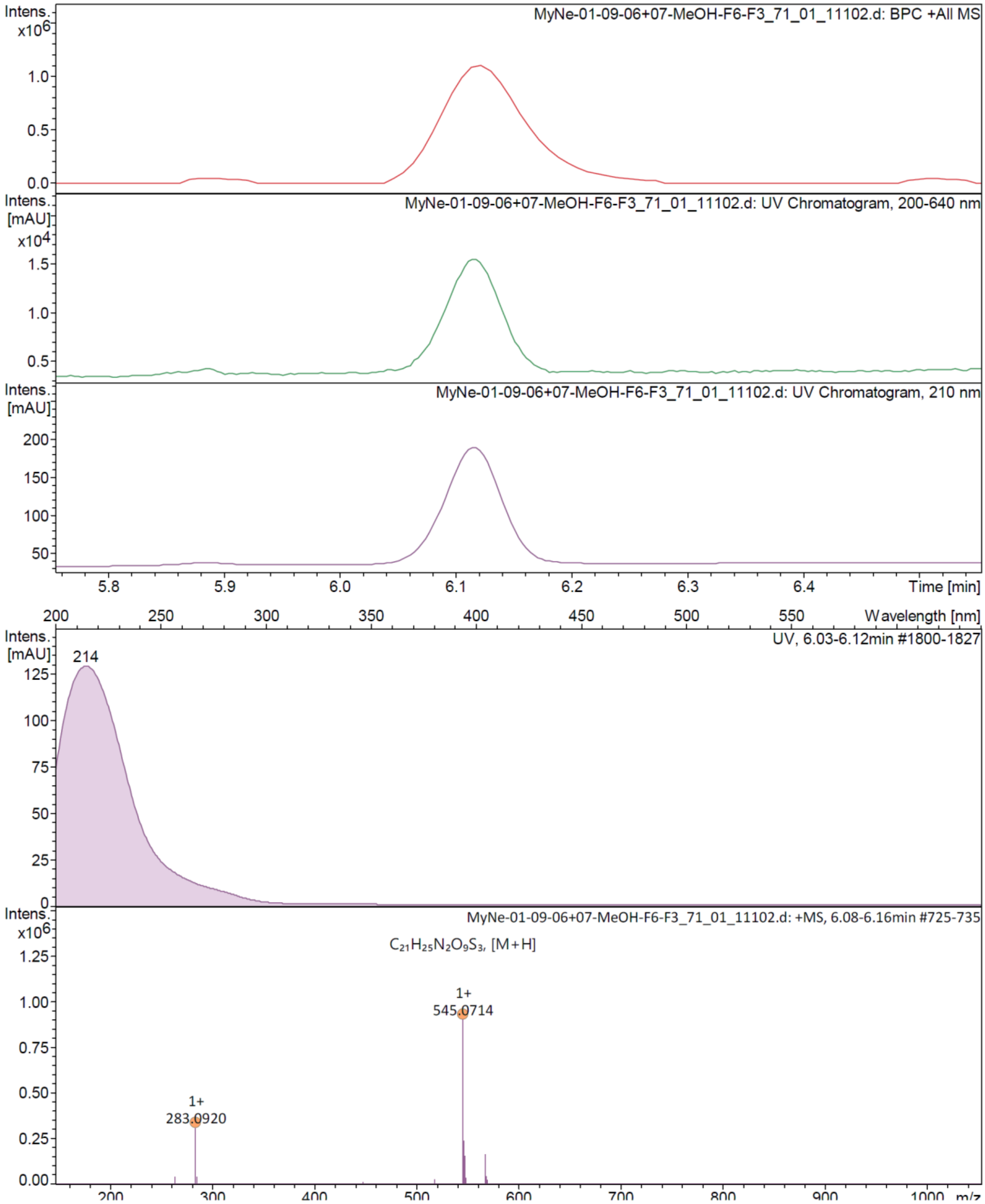

Figure S47. HRESIMS of **7**.

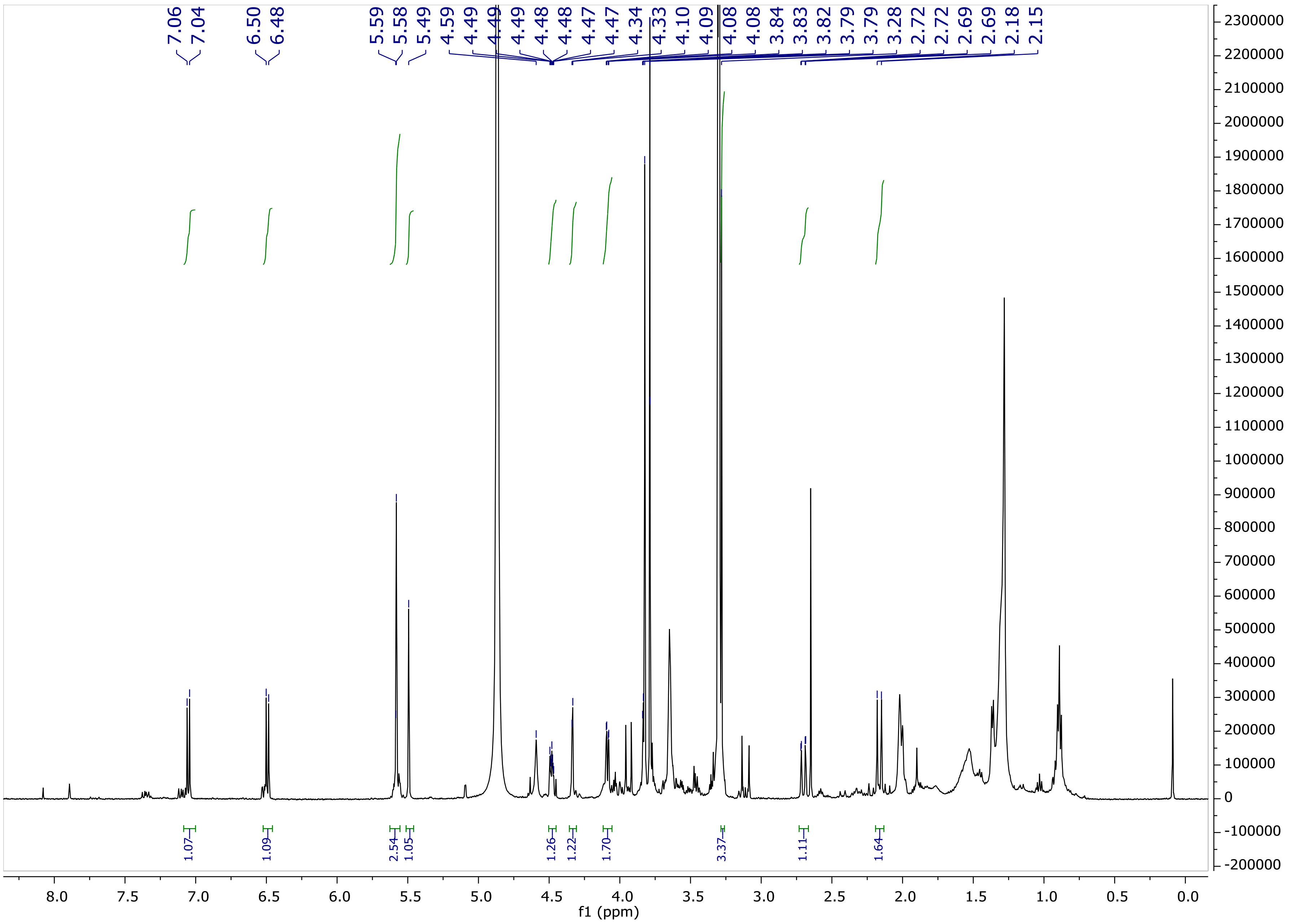

Figure S48. ^{1}H NMR spectrum of **7** in methanol-d_4 at 500 MHz.

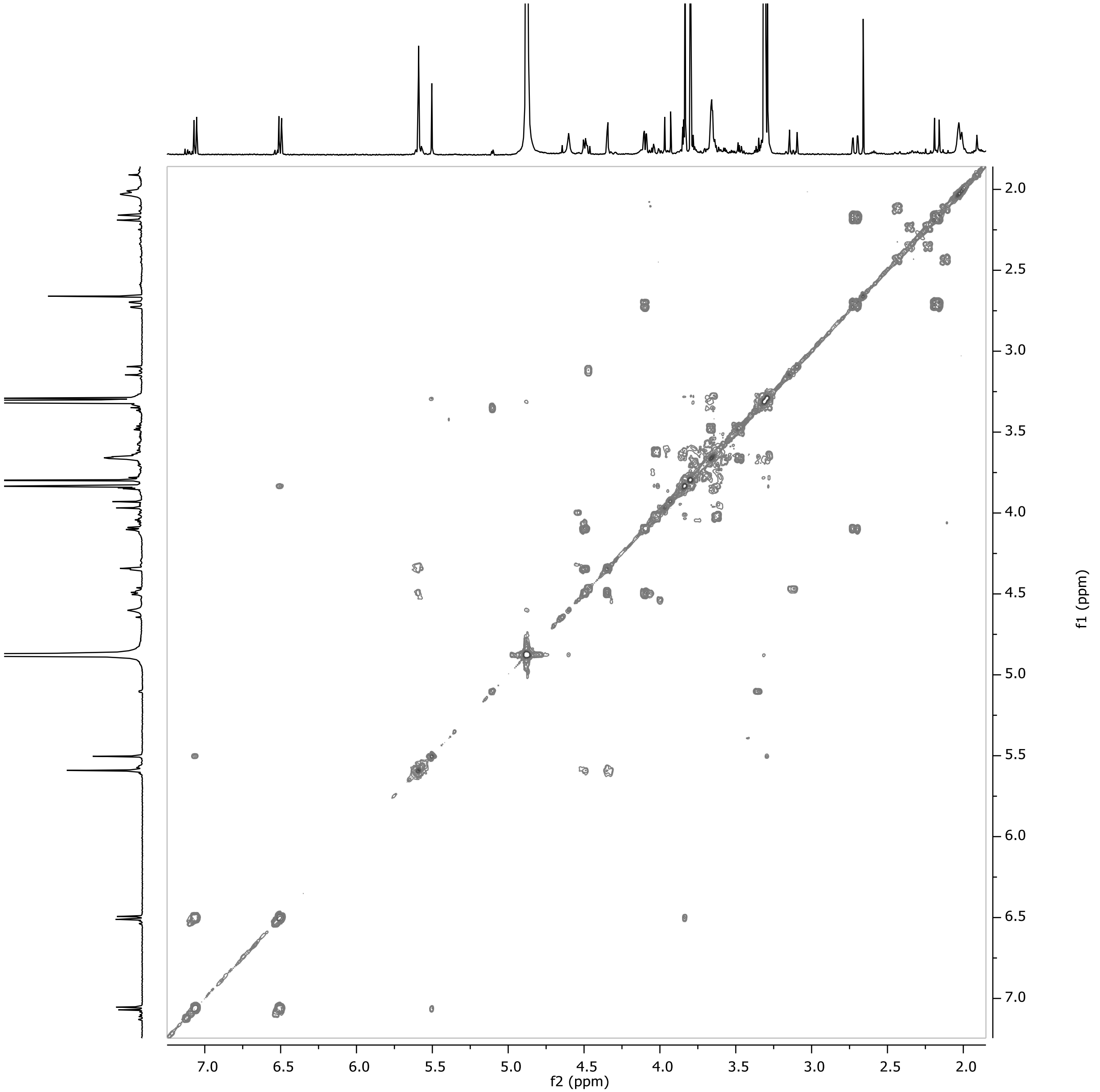

Figure S49. ^{1}H-^{1}H COSY spectrum of **7** in methanol-d_4 at 500 MHz.

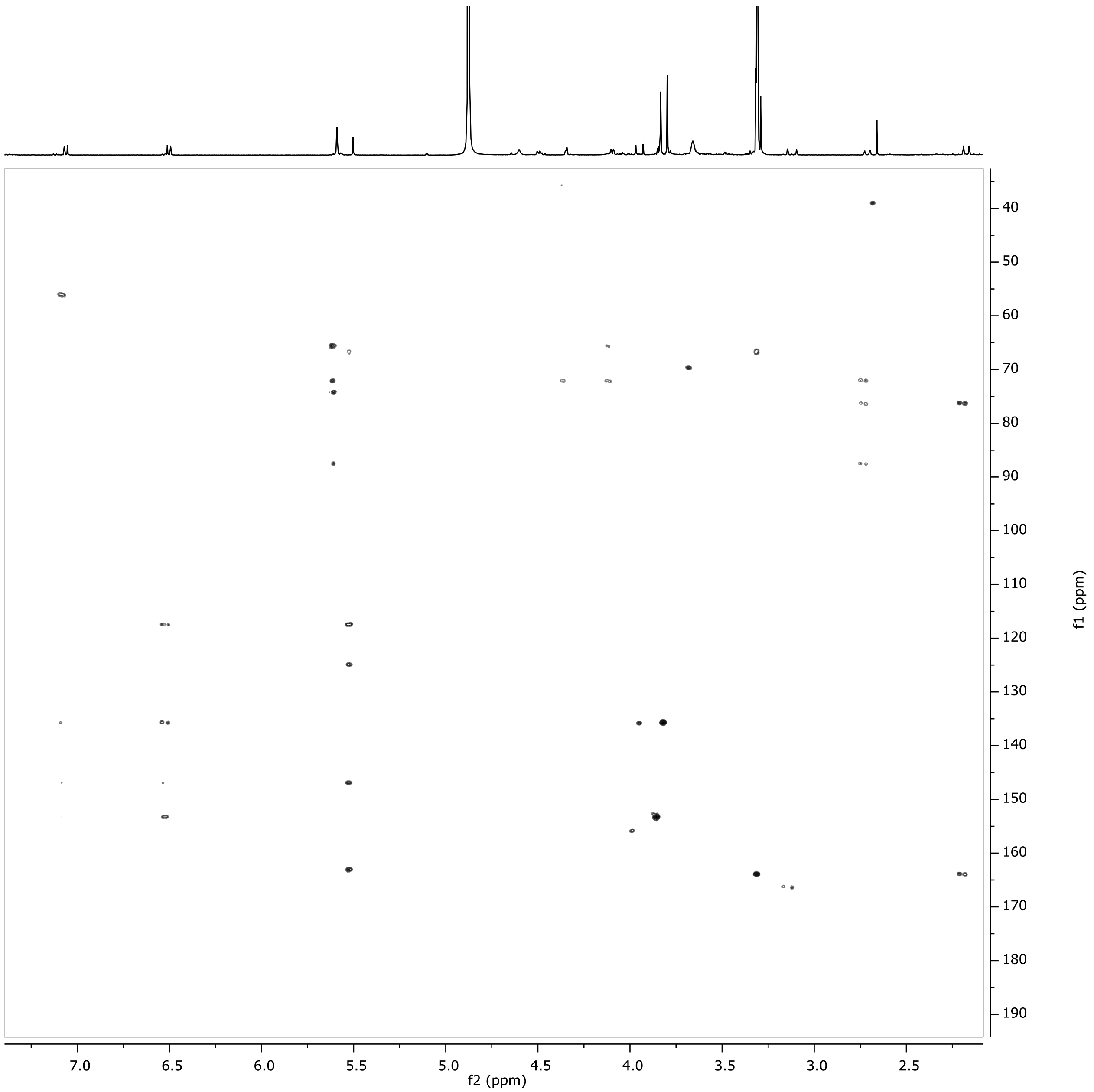

Figure S50. HMBC spectrum of **7** in methanol-d_4 at 500 MHz.

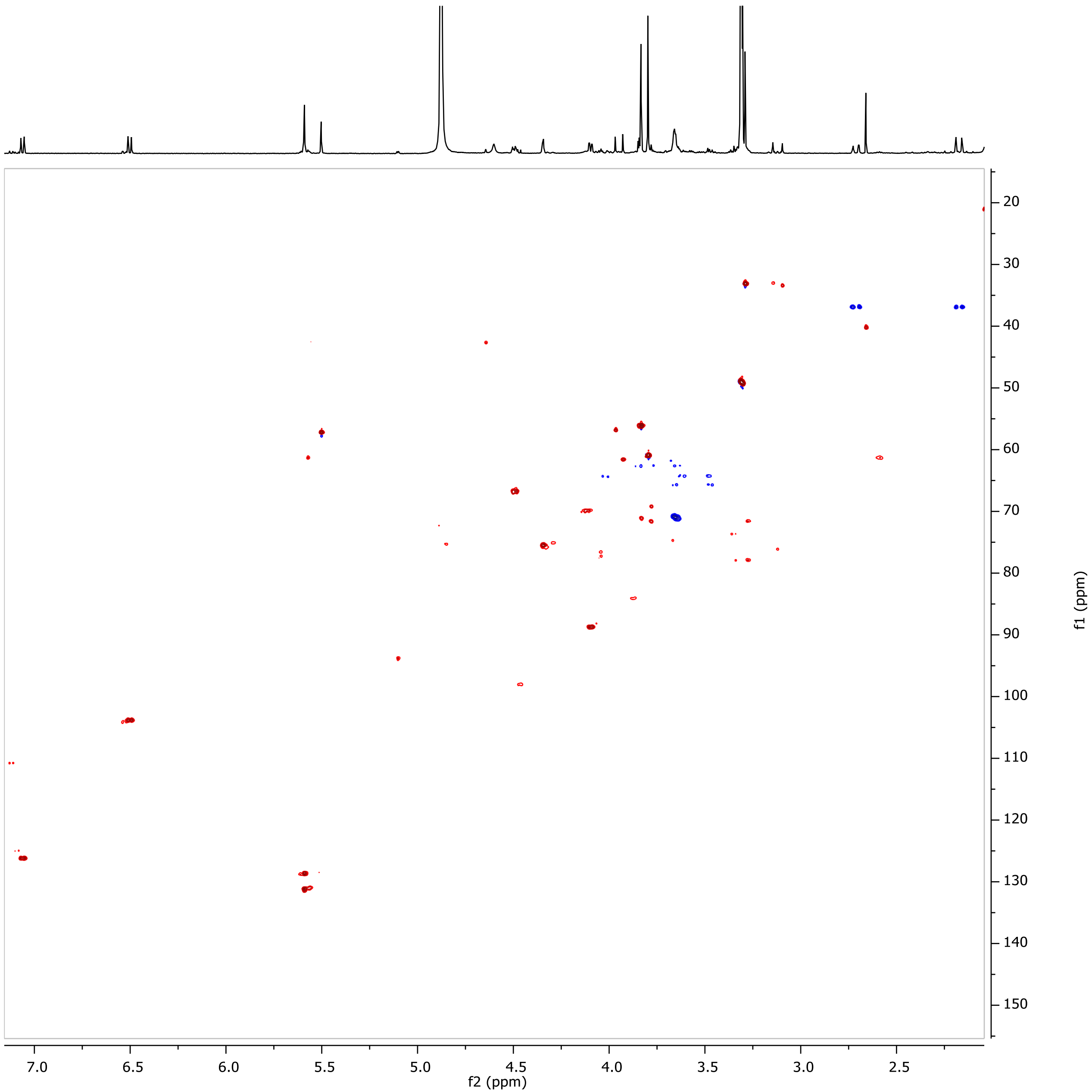

Figure S51. HSQC spectrum of **7** in methanol-d_4 at 500 MHz.

Generic Display Report

Analysis Info

Analysis Name S:\DATA\AmaZon\jpw20_Jan-Peer Wennrich\MycoNem_HPLC\MyNe_09\F6\MyNe-01-09-06+07-MeOH-F6-F2_RB2_01_42270.d
Method 42270.m
Sample Name MyNe-01-09-06+07-MeOH-F6-F2
Comment

Acquisition Date 14.10.2022 01:39:13
Operator esu
Instrument amaZon speed

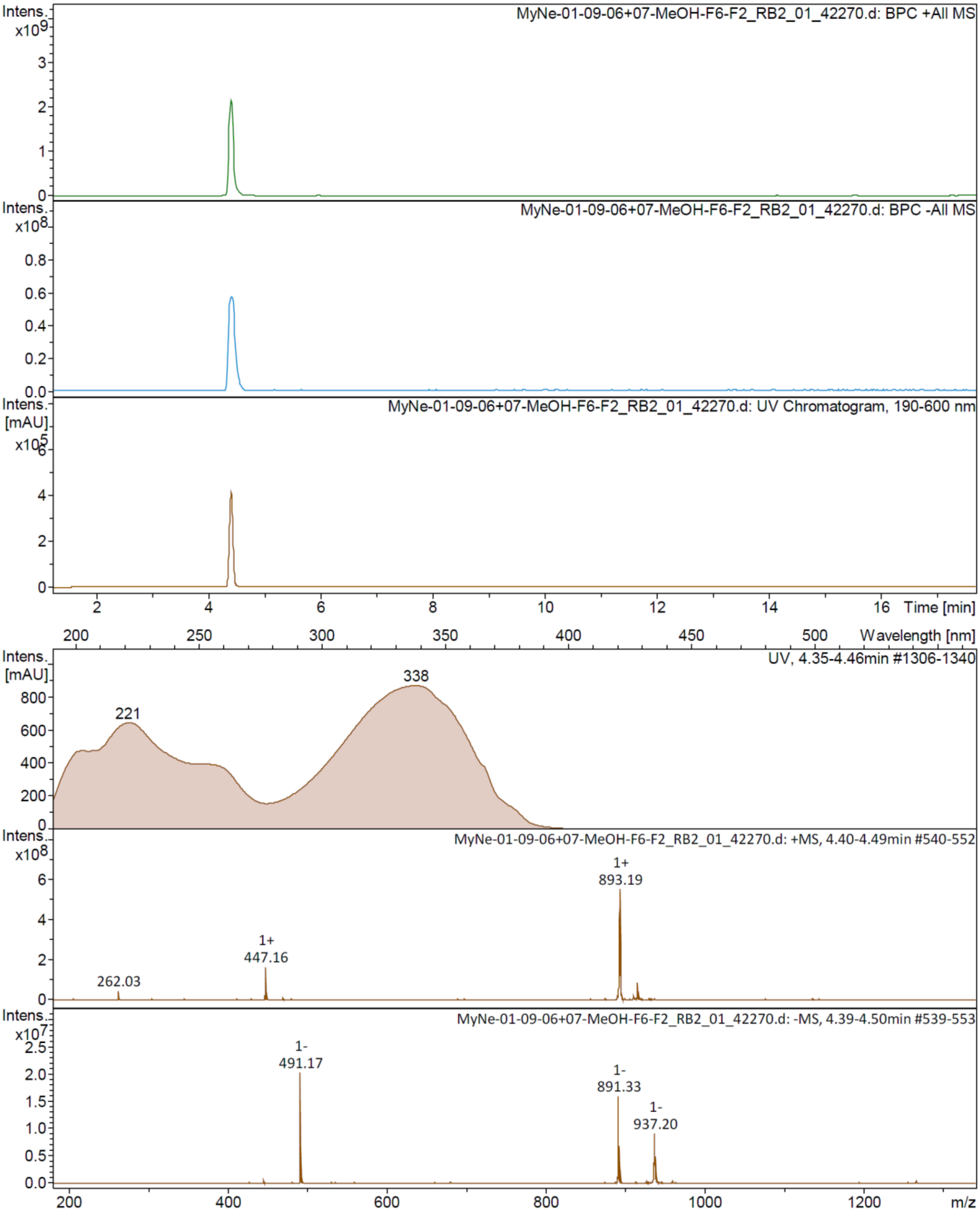

Bruker Compass DataAnalysis 4.4 printed: 08.11.2022 14:29:59 by: sel22 Page 1 of 1

Figure S52. LRESIMS of **8**.

Generic Display Report

Analysis Info

Analysis Name	C:\SEL22\Polyphellus_Ellen\MyNe-01-09-06+07-MeOH-F6-F2_21_01_10977.d	Acquisition Date	01.11.2022 10:36:33
Method	pos_säure_10000_screening_ms_100_2500_line.m	Operator	ate06
Sample Name	MyNe-01-09-06+07-MeOH-F6-F2	Instrument	maXis
Comment	Screening01<br>Waters Acquity UPLC BEH C_18 1,7um 2.1x50mm		

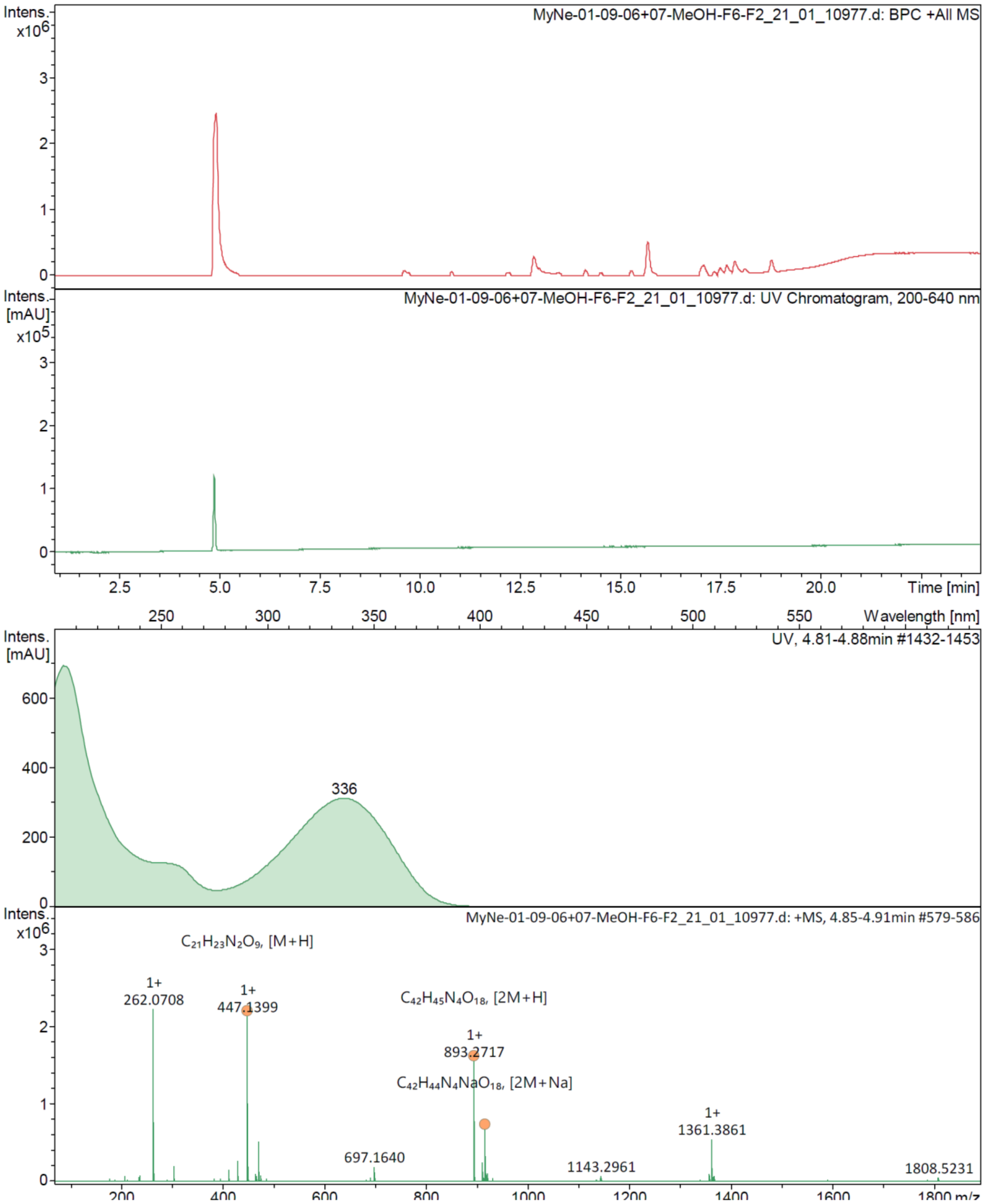

Bruker Compass DataAnalysis 4.4 printed: 06.11.2022 16:14:38 by: sel22 Page 1 of 1

Figure S53. HRESIMS of **8**.

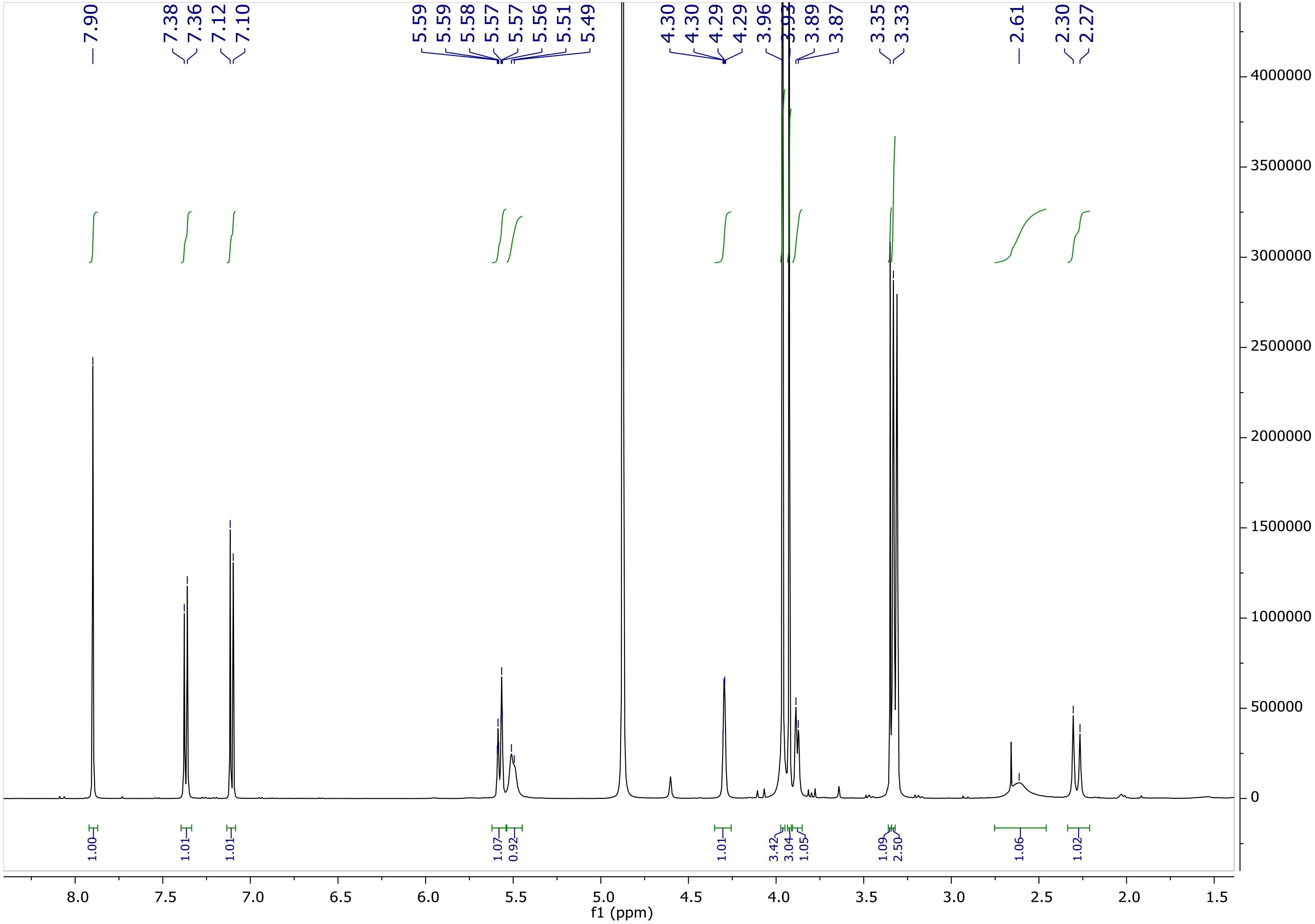

Figure S54. ^{1}H NMR spectrum of **8** in methanol-d_4 at 500 MHz.

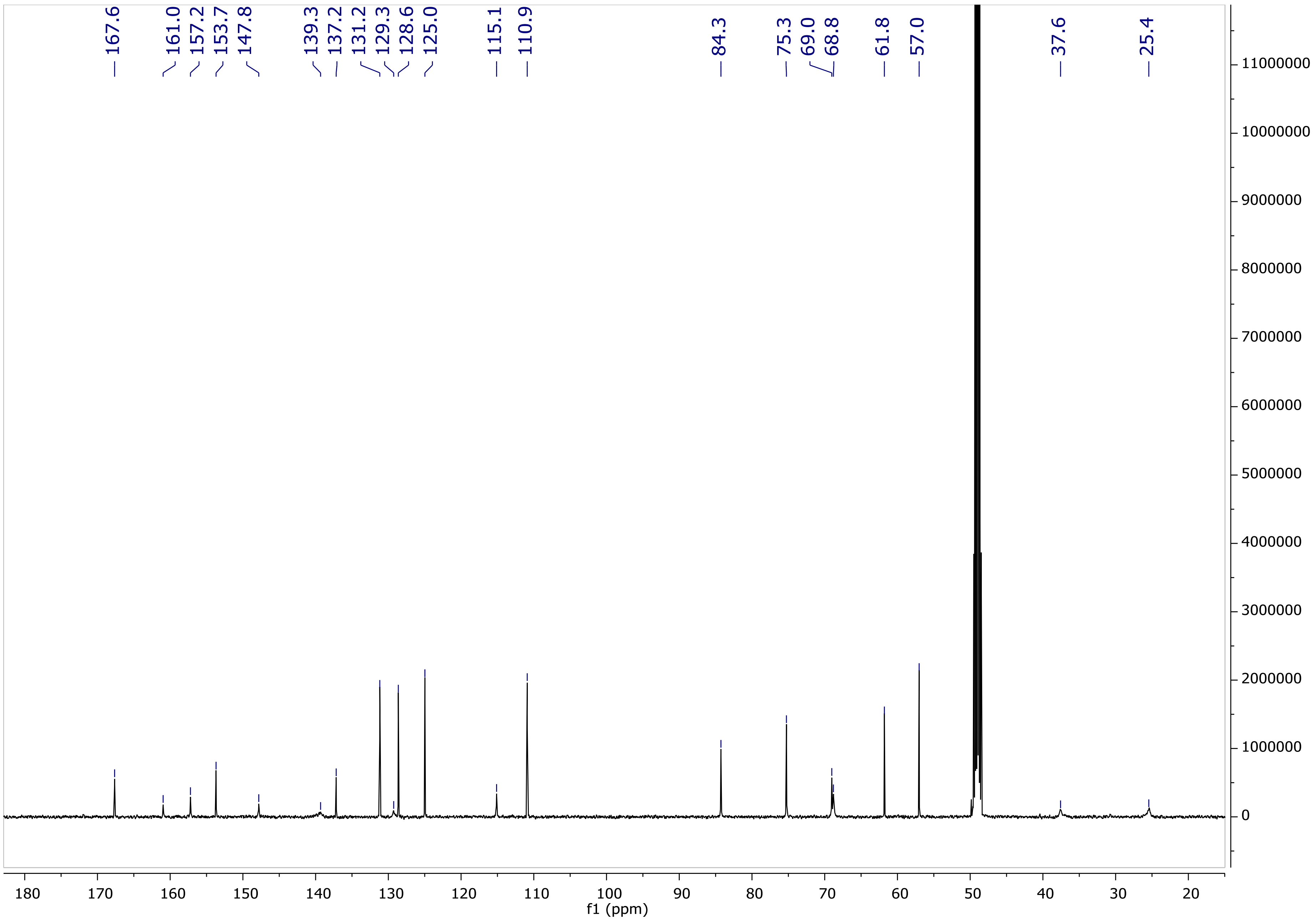

Figure S55. ^{13}C NMR spectrum of **8** in methanol-d_4 at 125 MHz.

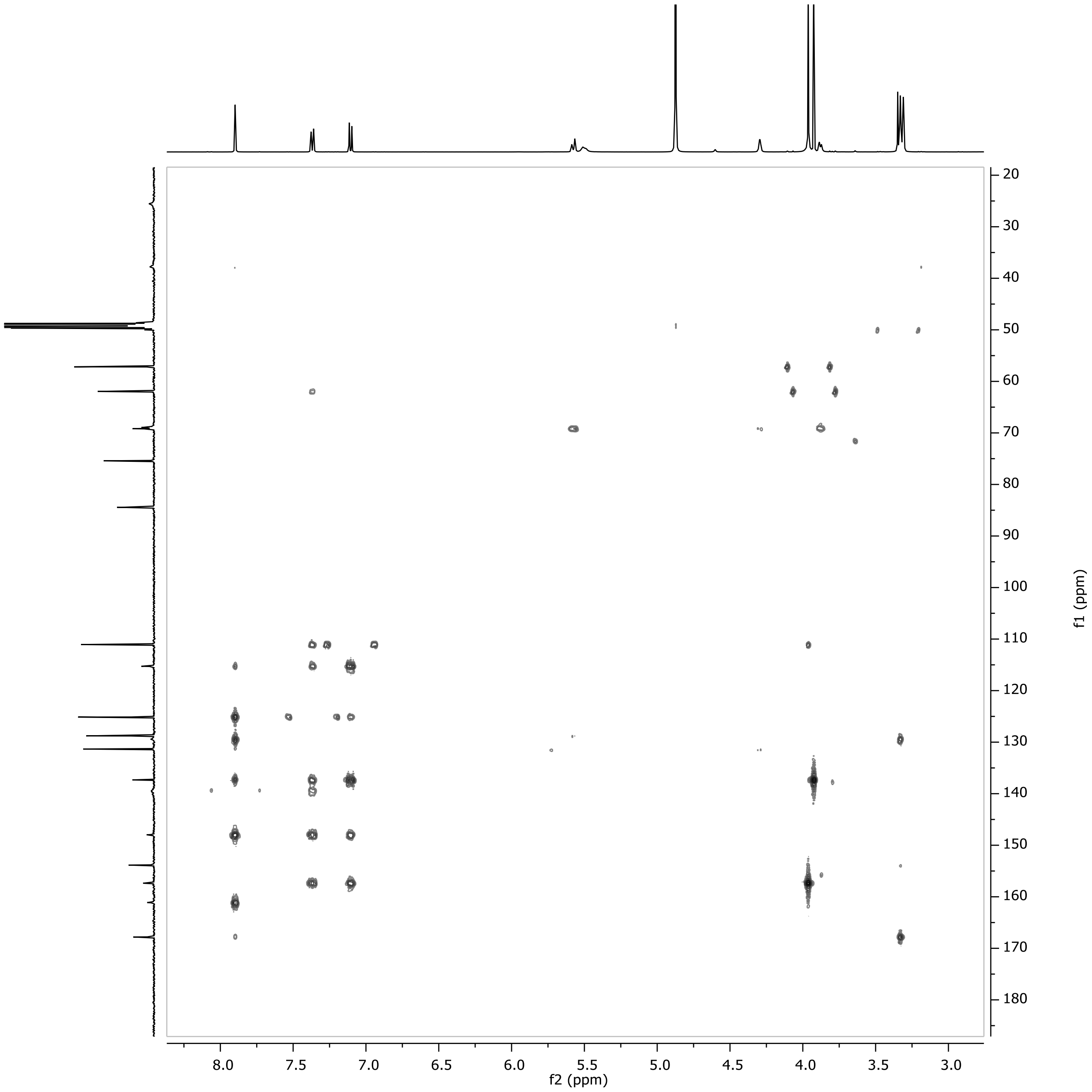

Figure S56. HMBC spectrum of **8** in methanol-d_4 at 500 MHz.

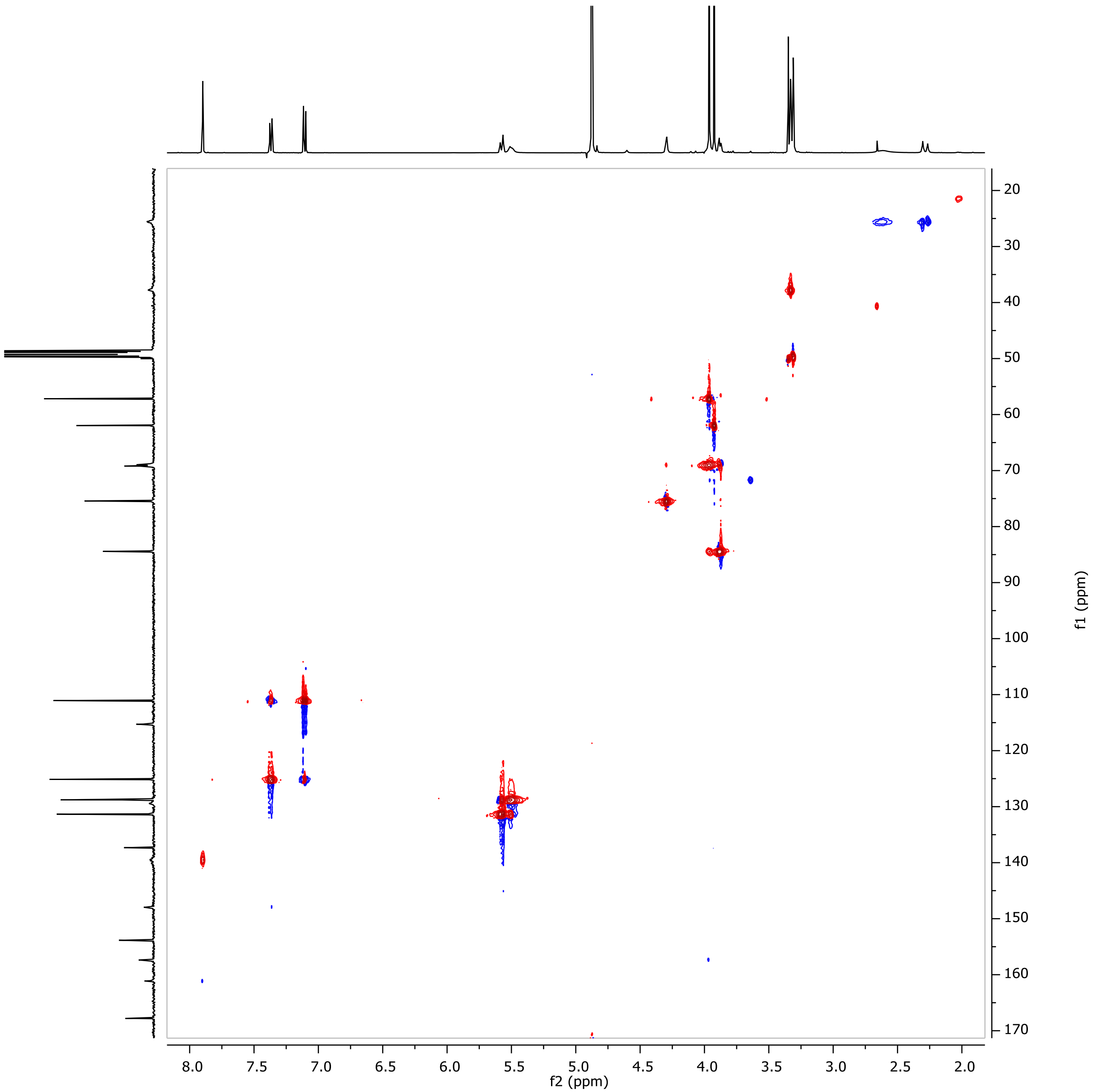

Figure S57. HSQC spectrum of **8** in methanol-d_4 at 500 MHz.

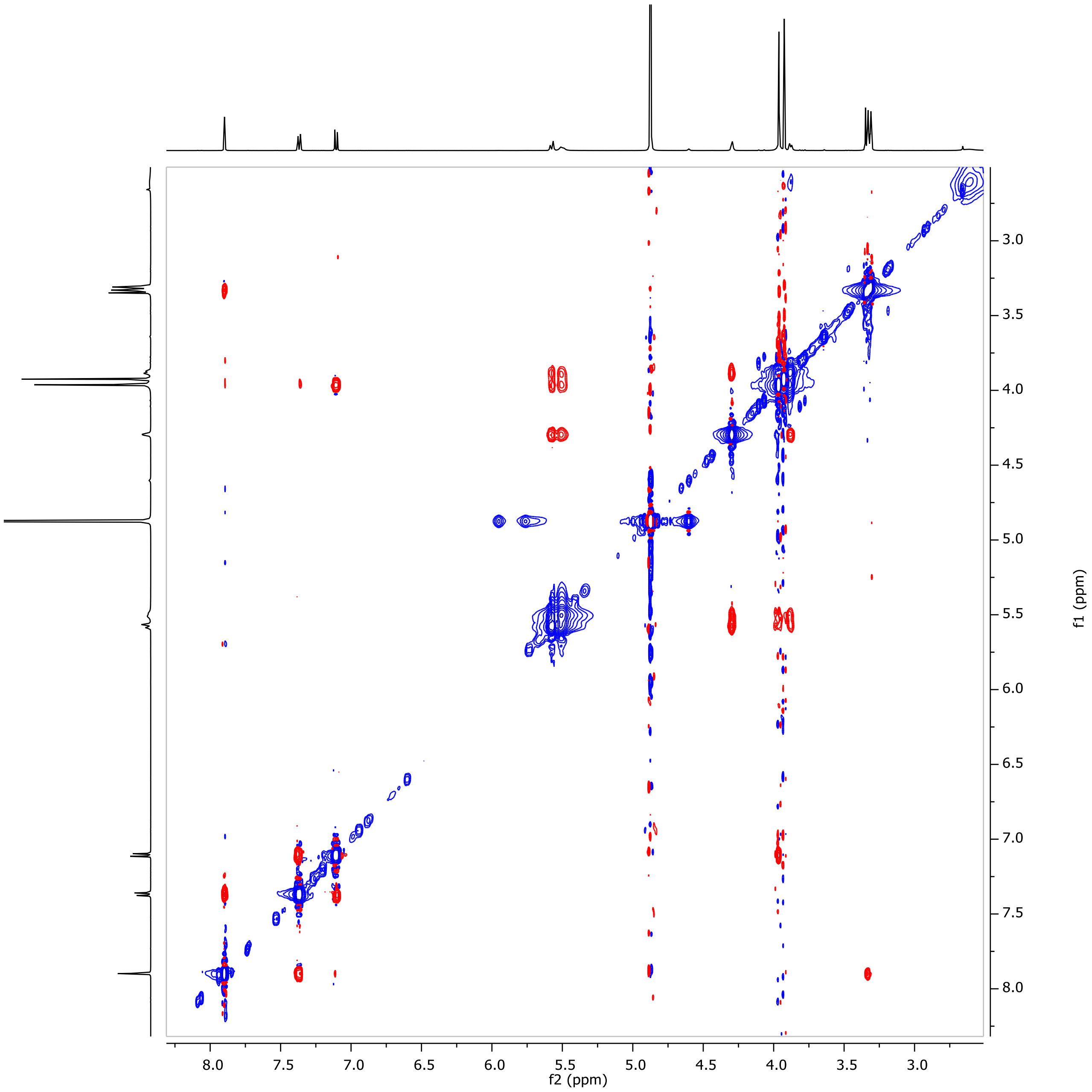

Figure S58. ROESY spectrum of **8** in methanol-d_4 at 500 MHz.

Generic Display Report

Analysis Info

Analysis Name	S:\DATA\MaXis\ESE22_Ellen Sepanian\23_02\MyNe-01-09-06+07-MeOH-F5-F7_21_01_11239.d	Acquisition Date	15.02.2023 11:55:55
Method	pos_säure_10000_screening_ms_100_2500_line.m	Operator	ate06
Sample Name	MyNe-01-09-06+07-MeOH-F5-F7	Instrument	maXis
Comment	Screening01<br>Waters Acquity UPLC BEH C_18 1,7um 2.1x50mm		

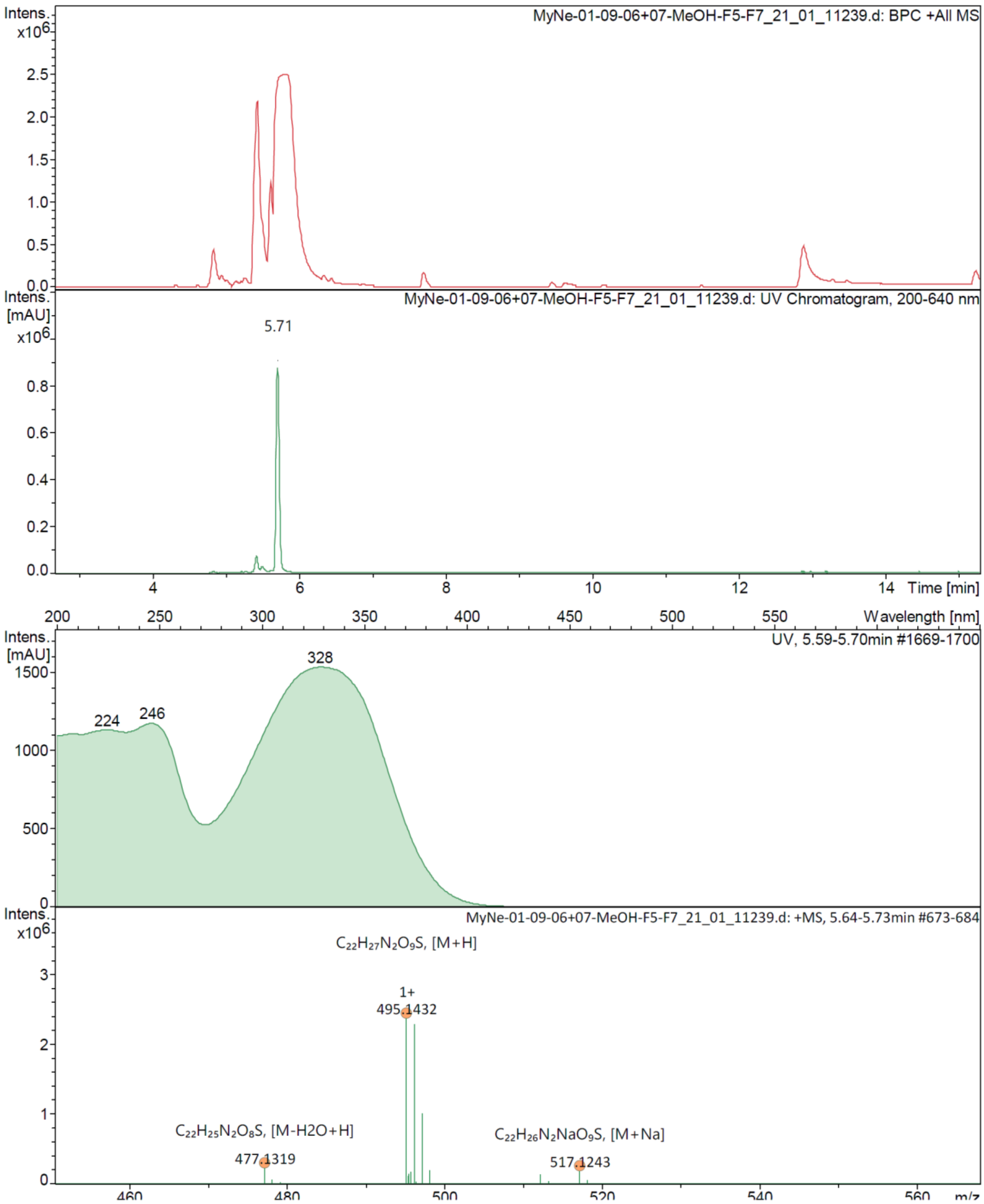

Bruker Compass DataAnalysis 4.4 printed: 09.06.2023 19:58:48 by: sel22 Page 1 of 1

Figure S59. HRESIMS of **9**.

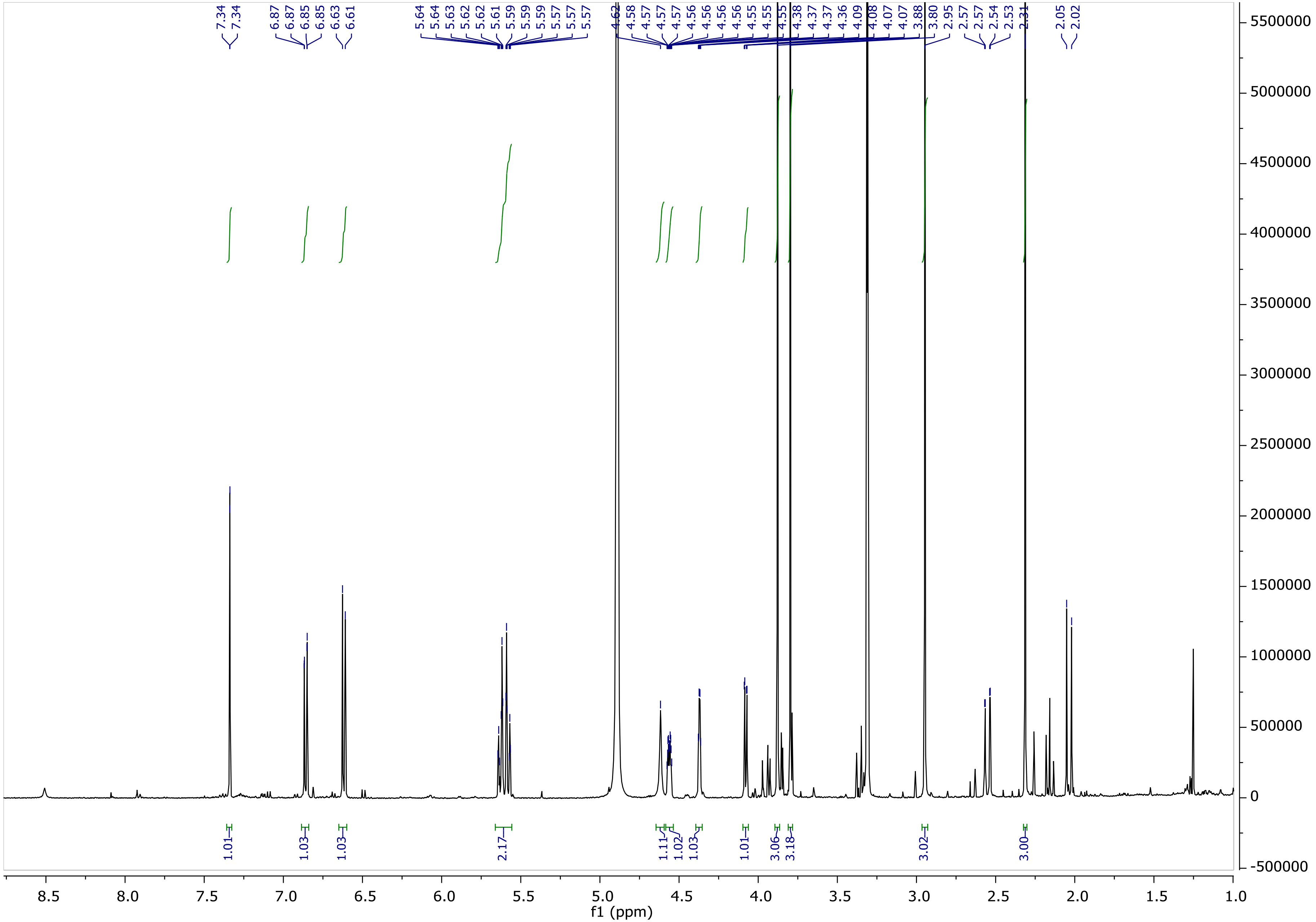

Figure S60. ^{1}H NMR spectrum of **9** in methanol-d_4 at 700 MHz.

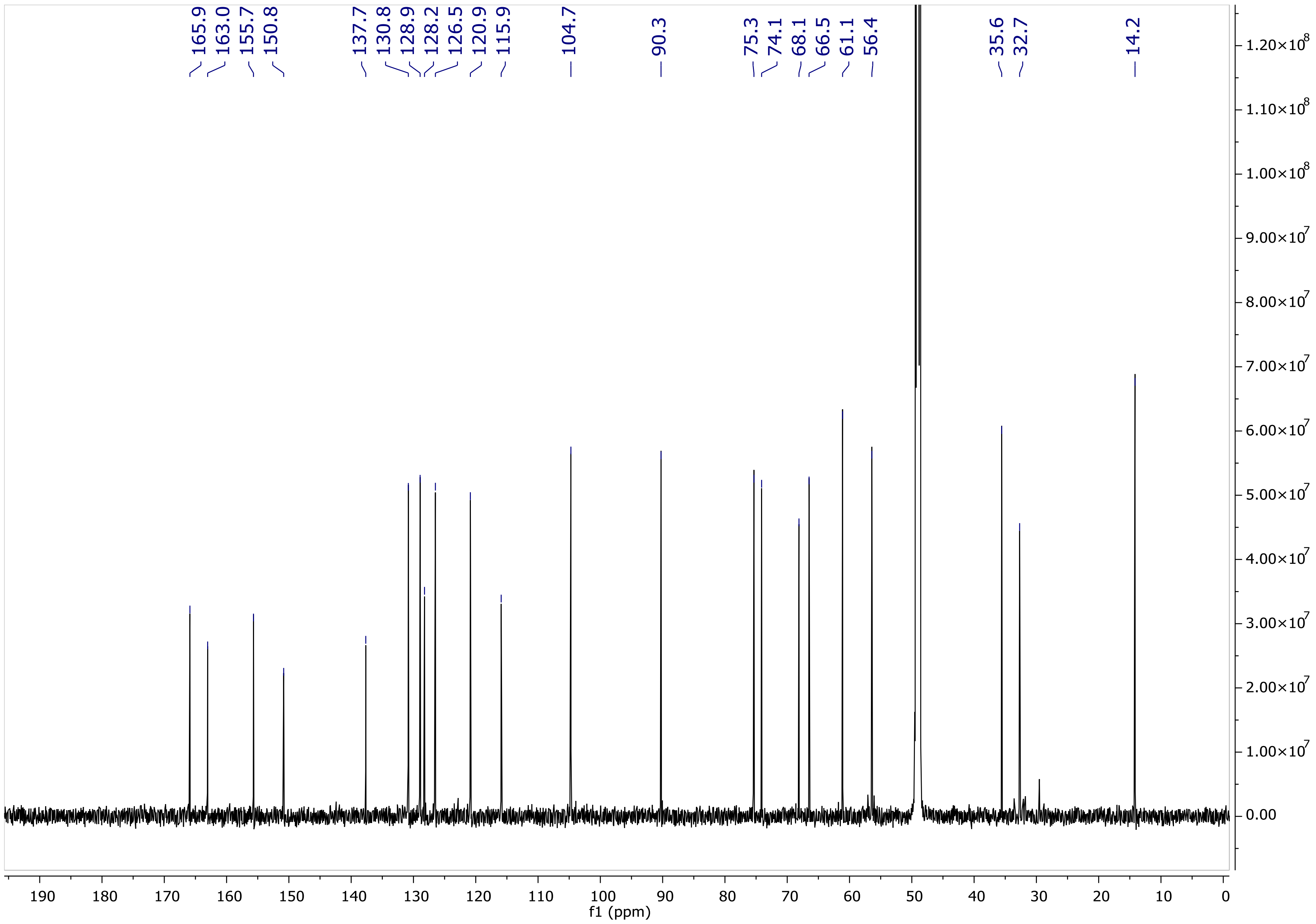

Figure S61. ^{13}C NMR spectrum of **9** in methanol-d_4 at 175 MHz.

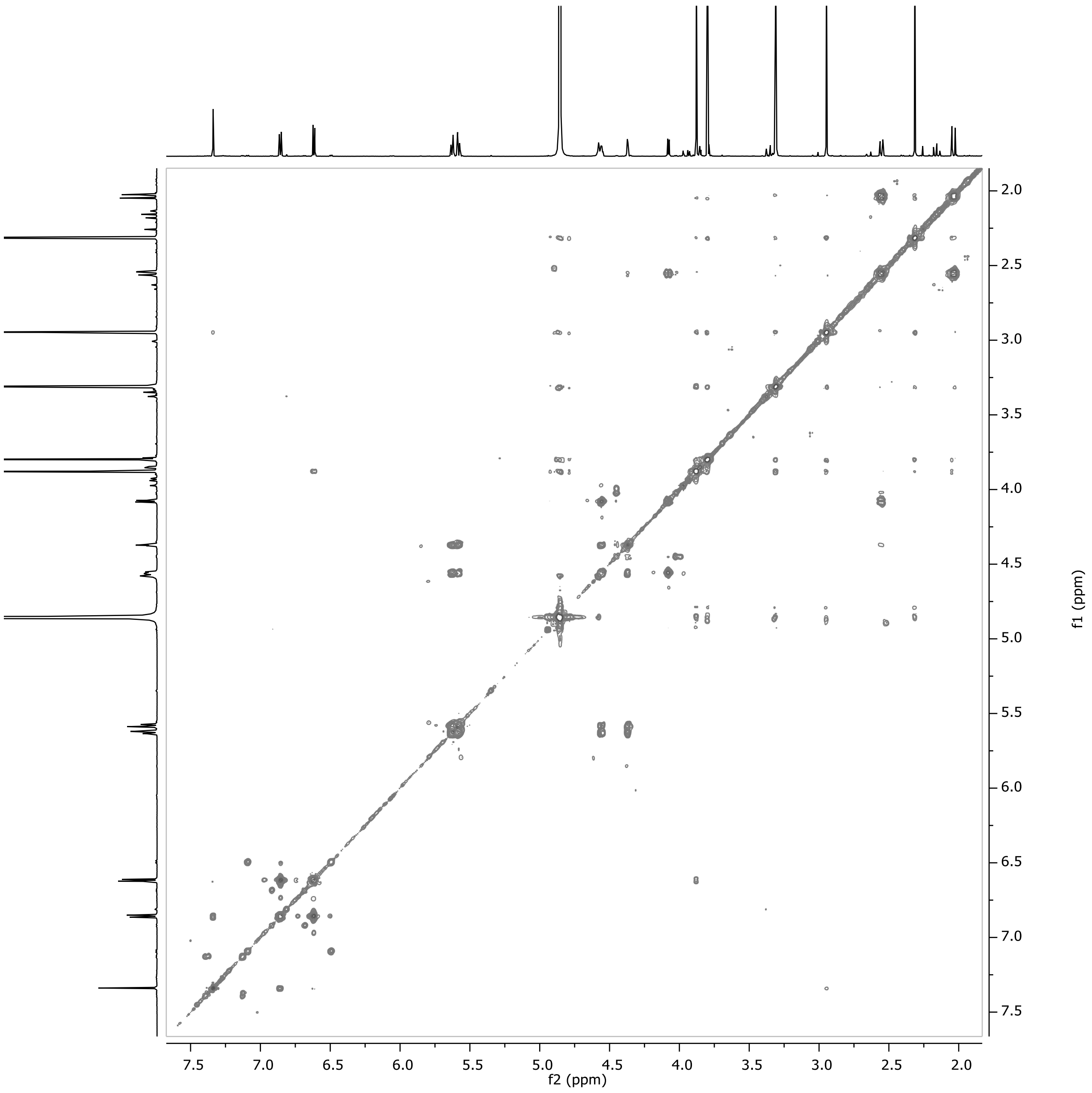

Figure S62. ^{1}H-^{1}H COSY spectrum of **9** methanol-d_4 at 700 MHz.

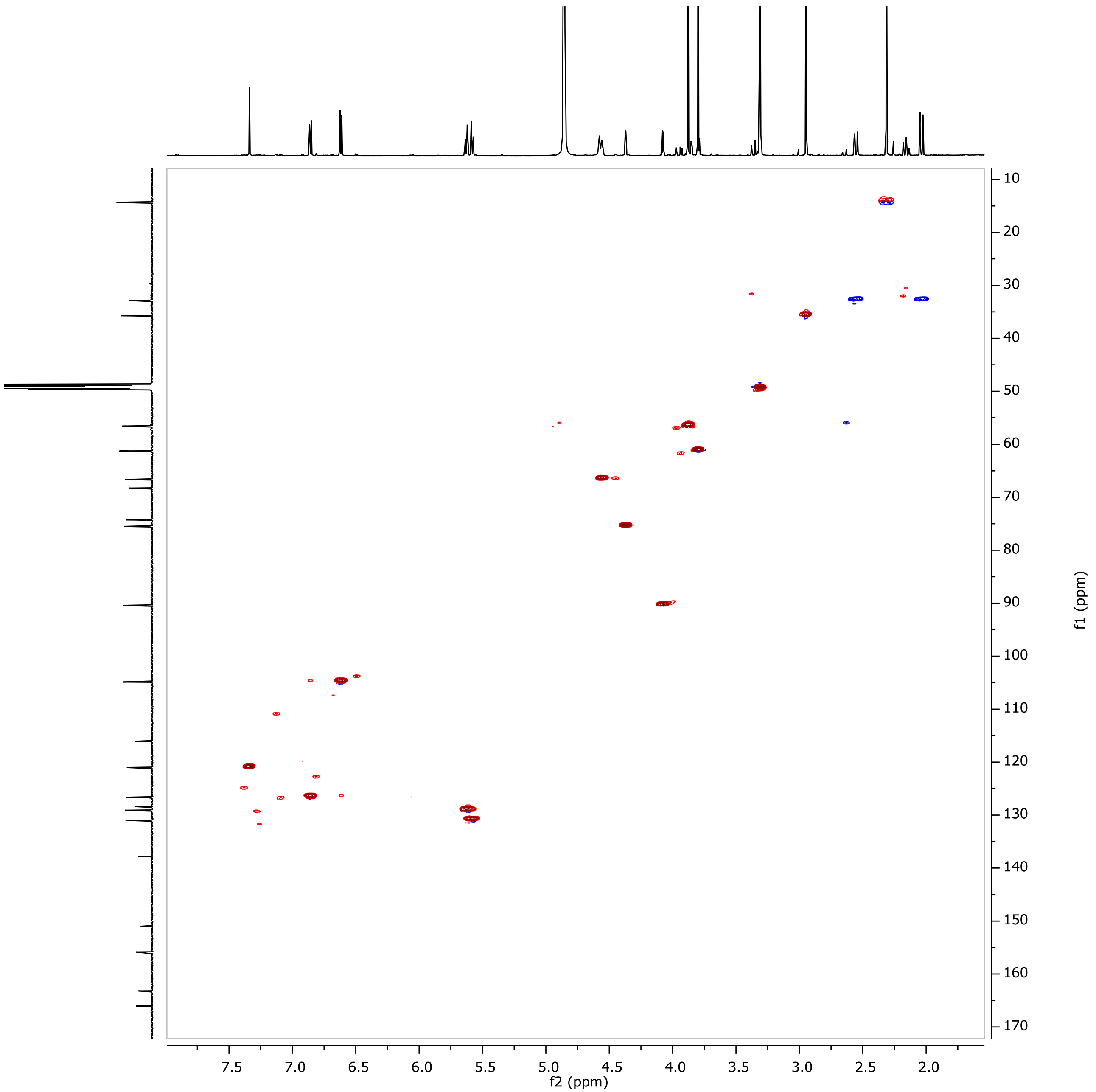

Figure S63. HMBC spectrum of **9** methanol-d_4 at 700 MHz.

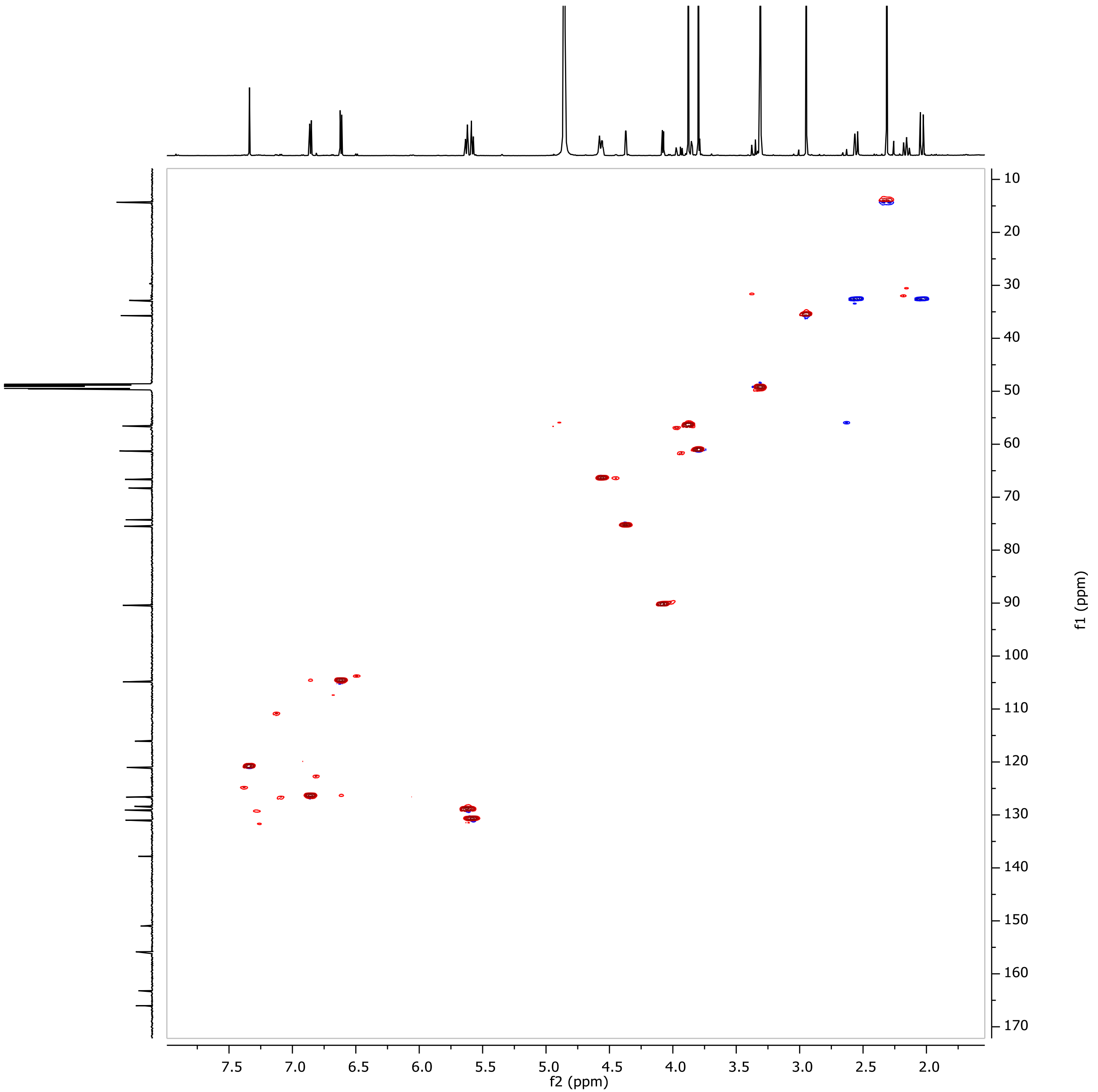

Figure S64. HSQC spectrum of **9** methanol-d_4 at 700 MHz.

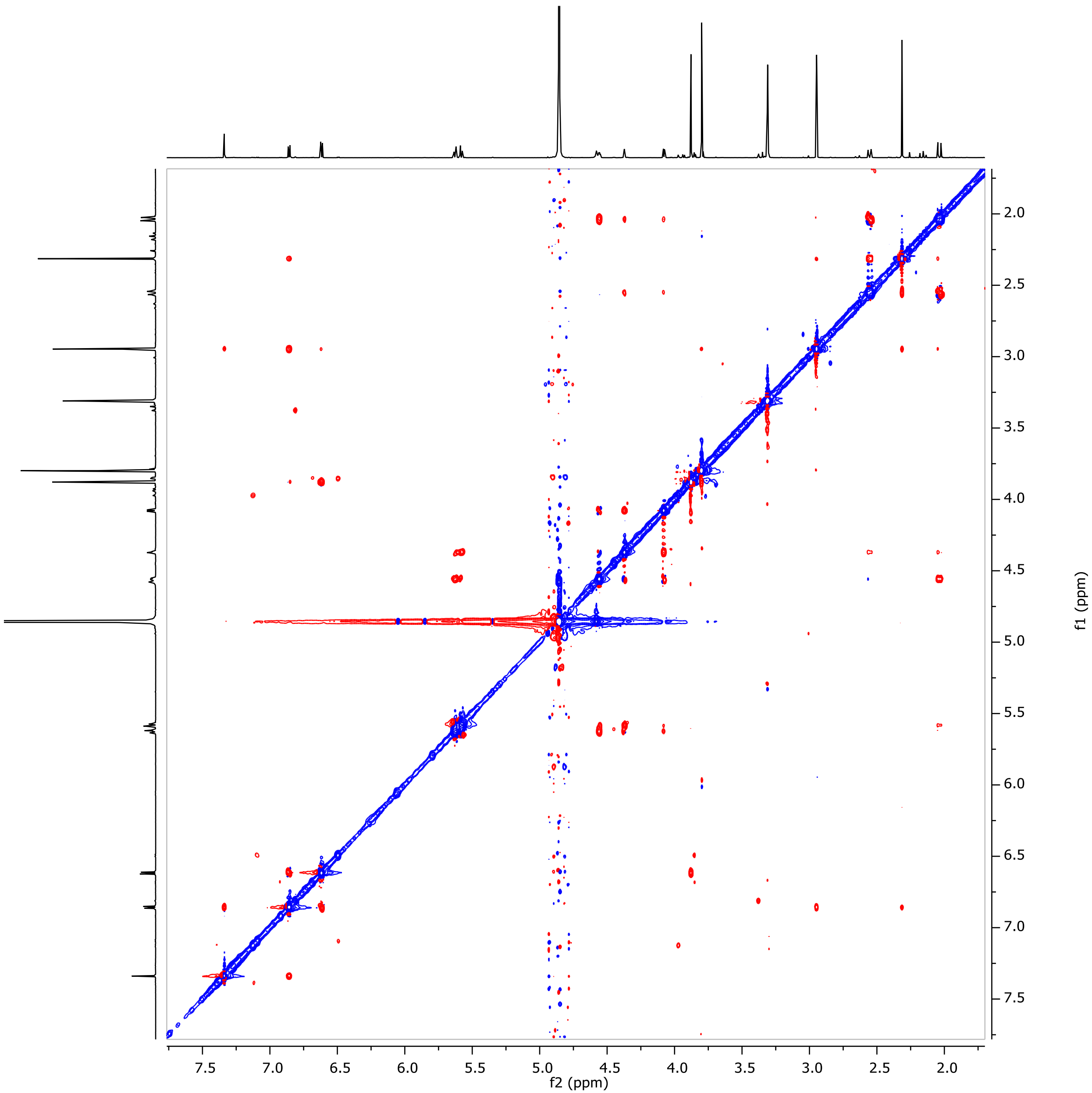

Figure S65. ROESY spectrum of **9** methanol-d_4 at 700 MHz.

Generic Display Report

Analysis Info

Analysis Name	S:\DATA\AmaZon\jpw20_Jan-Peer Wennrich\MycoNem_HPLC\MyNe_11\MyNe_01_11_06+07_MeOH_F9\MyNe-01-11-06+07-MeOH-F9-F12_RD4_01_42309.d	Acquisition Date	15.10.2022 02:52:20
Method	42309.m	Operator	esu
Sample Name	MyNe-01-11-06+07-MeOH-F9-F12	Instrument	amaZon speed
Comment			

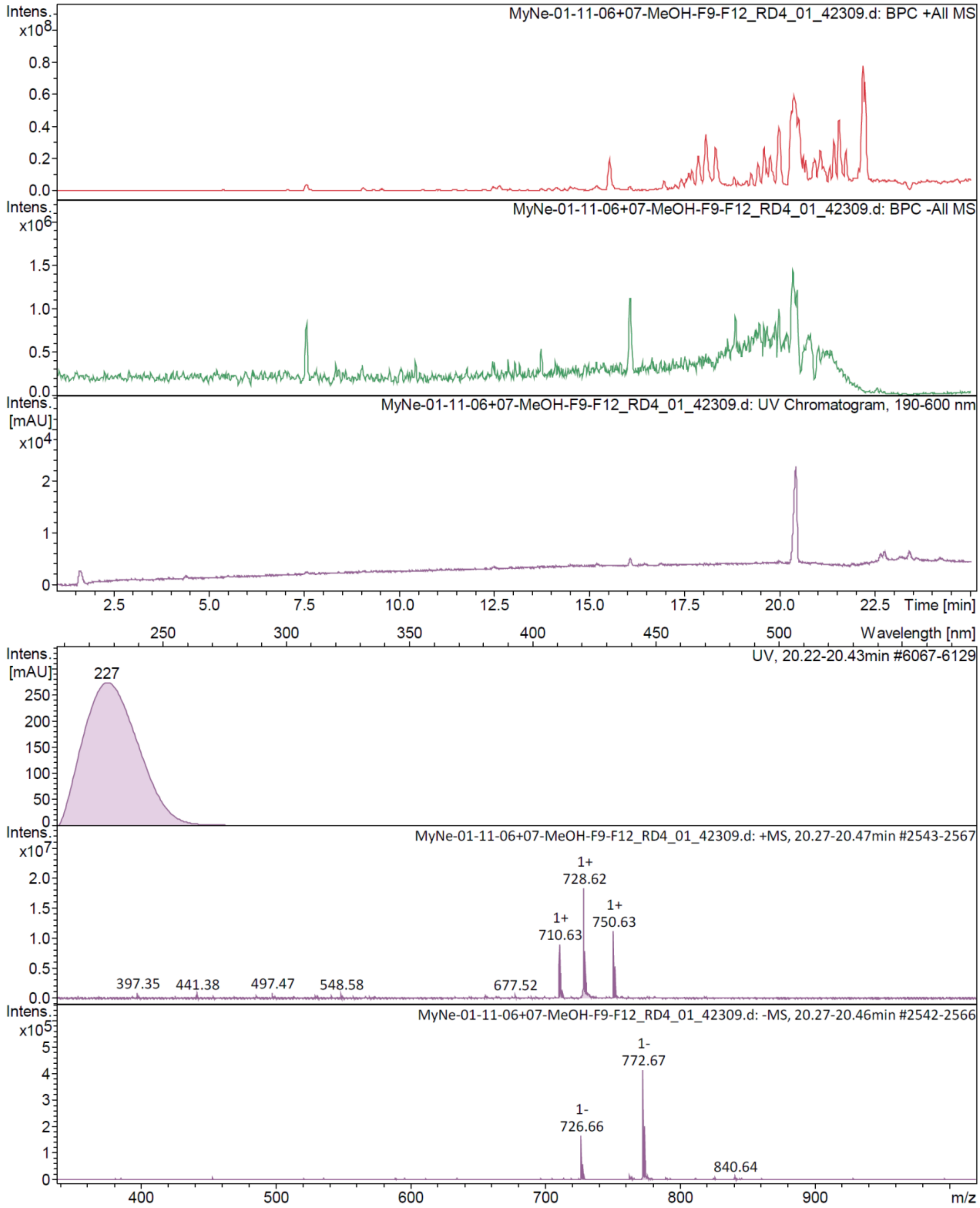

Bruker Compass DataAnalysis 4.4 printed: 08.11.2022 14:27:16 by: sel22 Page 1 of 1

Figure S66. LRESIMS of **10**.

Generic Display Report

Analysis Info

Analysis Name	S:\DATA\MaXis\ESE22_Ellen Sepanian\22_11\MyNe-01-11-06+07-MeOH-F9-F12_23_01_10979.d	Acquisition Date	01.11.2022 11:38:30
Method	pos_säure_10000_screening_ms_100_2500_line.m	Operator	ate06
Sample Name	MyNe-01-11-06+07-MeOH-F9-F12	Instrument	maXis
Comment	Screening01<br>Waters Acquity UPLC BEH C_18 1,7um 2.1x50mm		

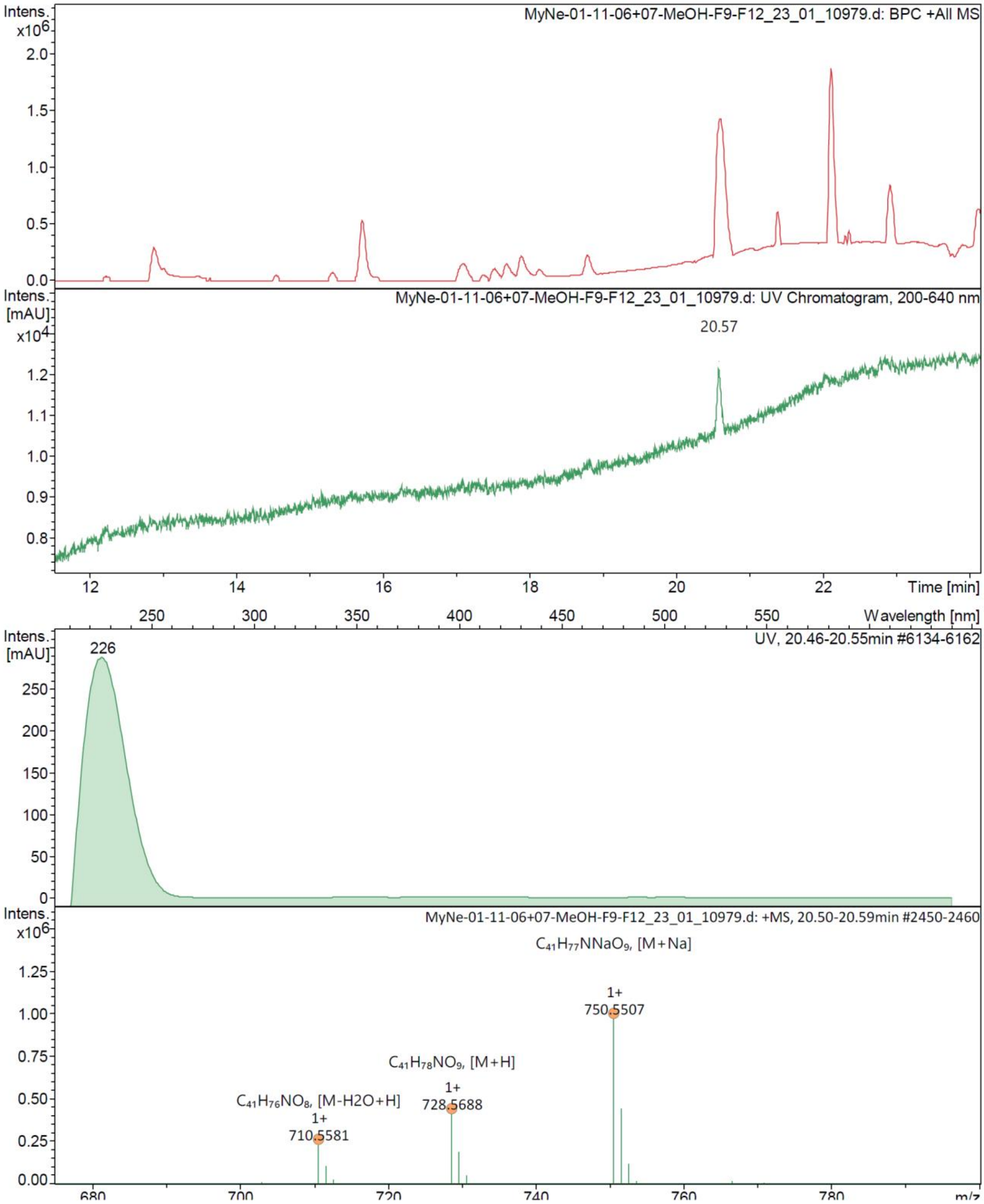

Bruker Compass DataAnalysis 4.4 printed: 09.06.2023 20:35:36 by: sel22 Page 1 of 1

Figure S67. HRESIMS of **10**.

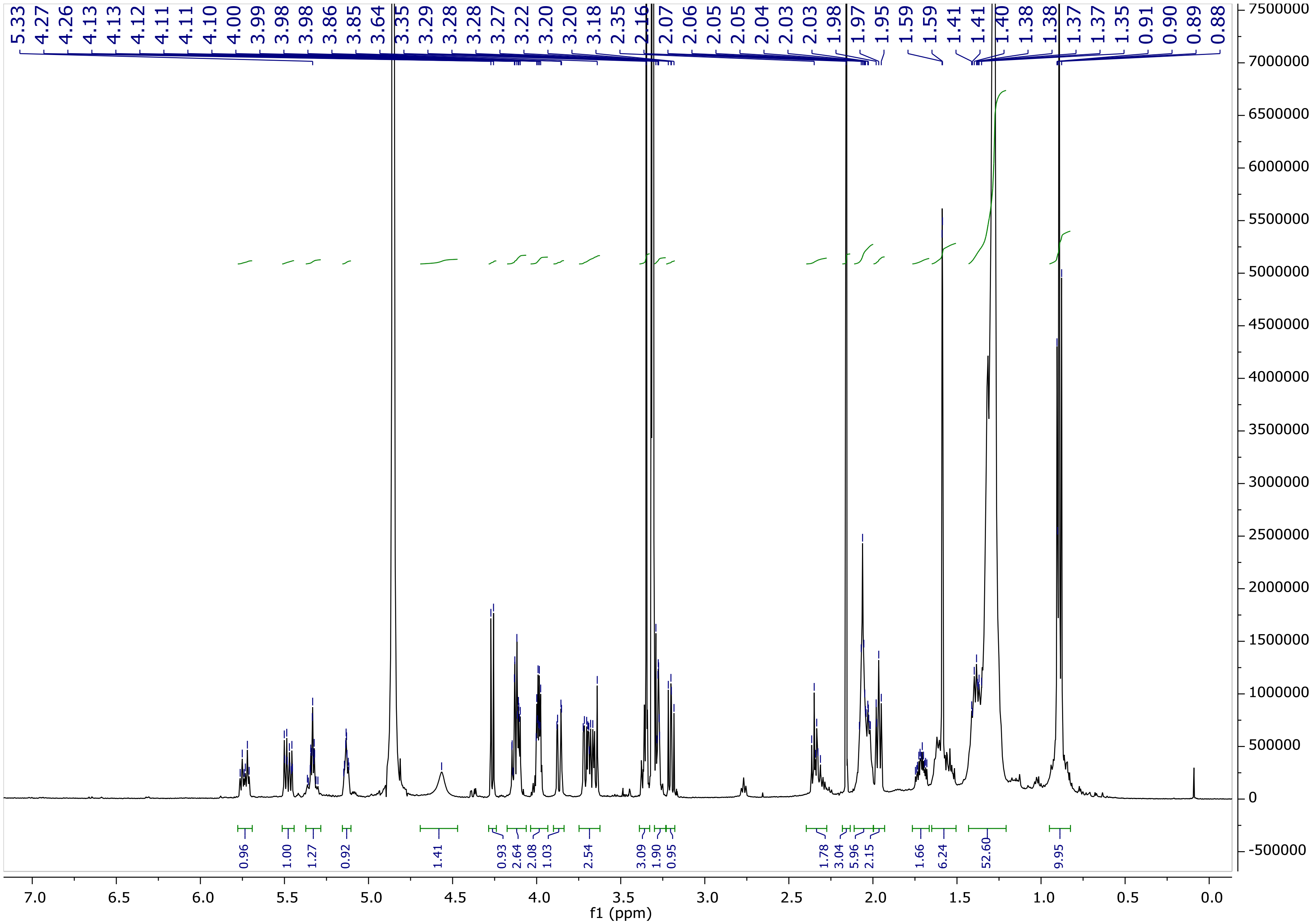

Figure S68. ^1H NMR spectrum of **10** in methanol-d_4 at 500 MHz.

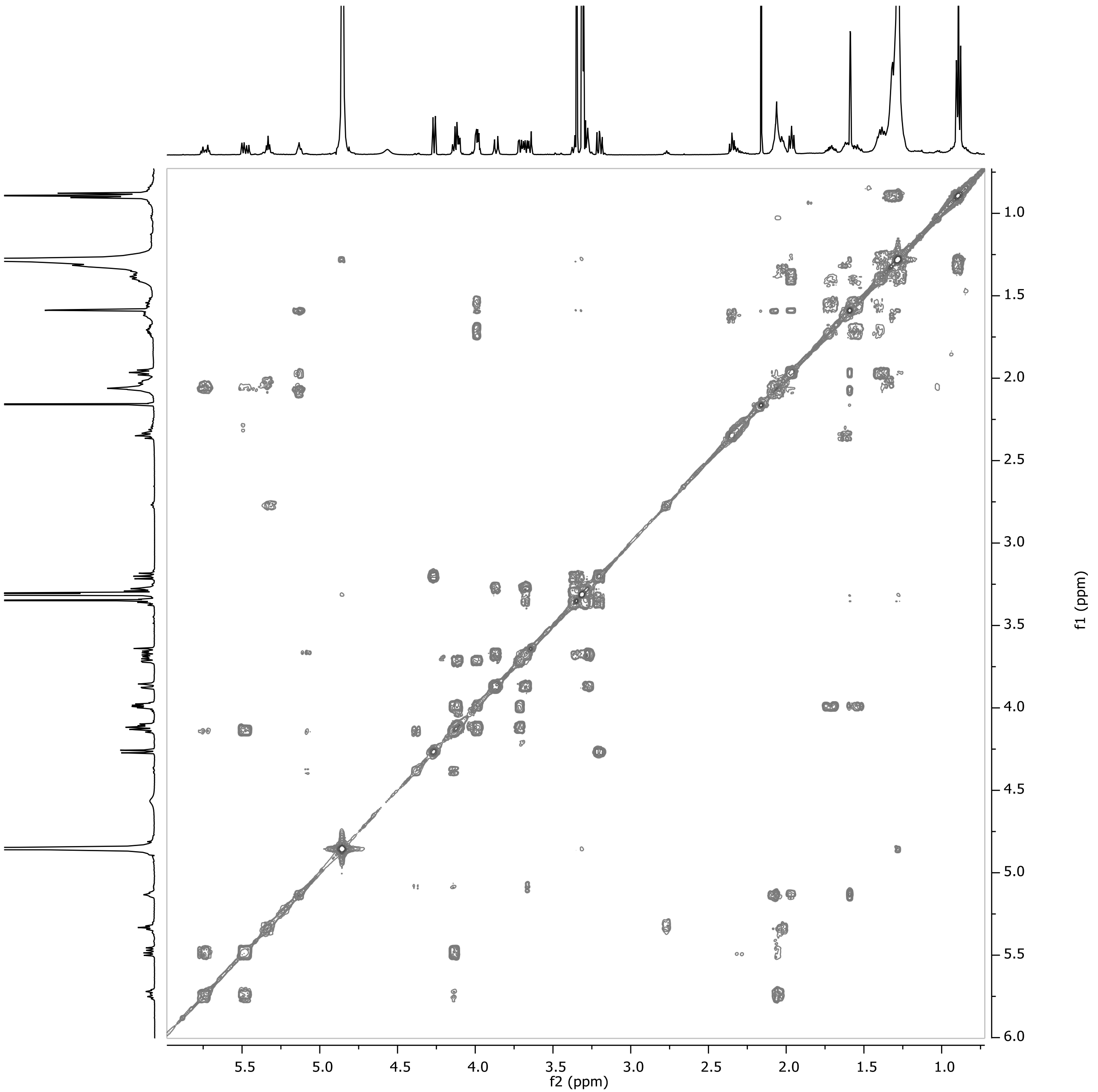

Figure S69. ^{1}H-^{1}H COSY spectrum of **10** in methanol-d_4 at 500 MHz.

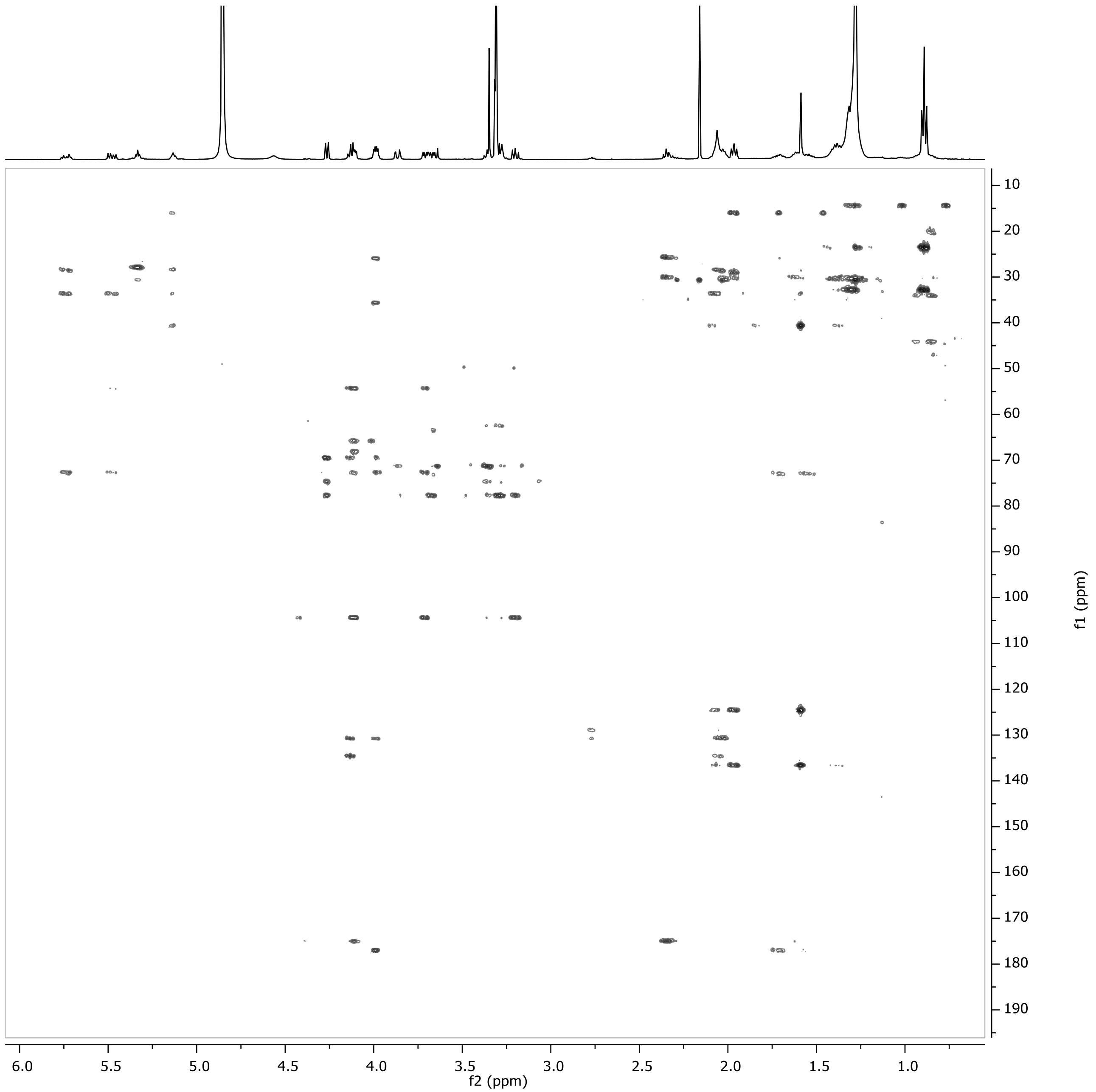

Figure S70. HMBC spectrum of **10** in methanol-d_4 at 500 MHz.

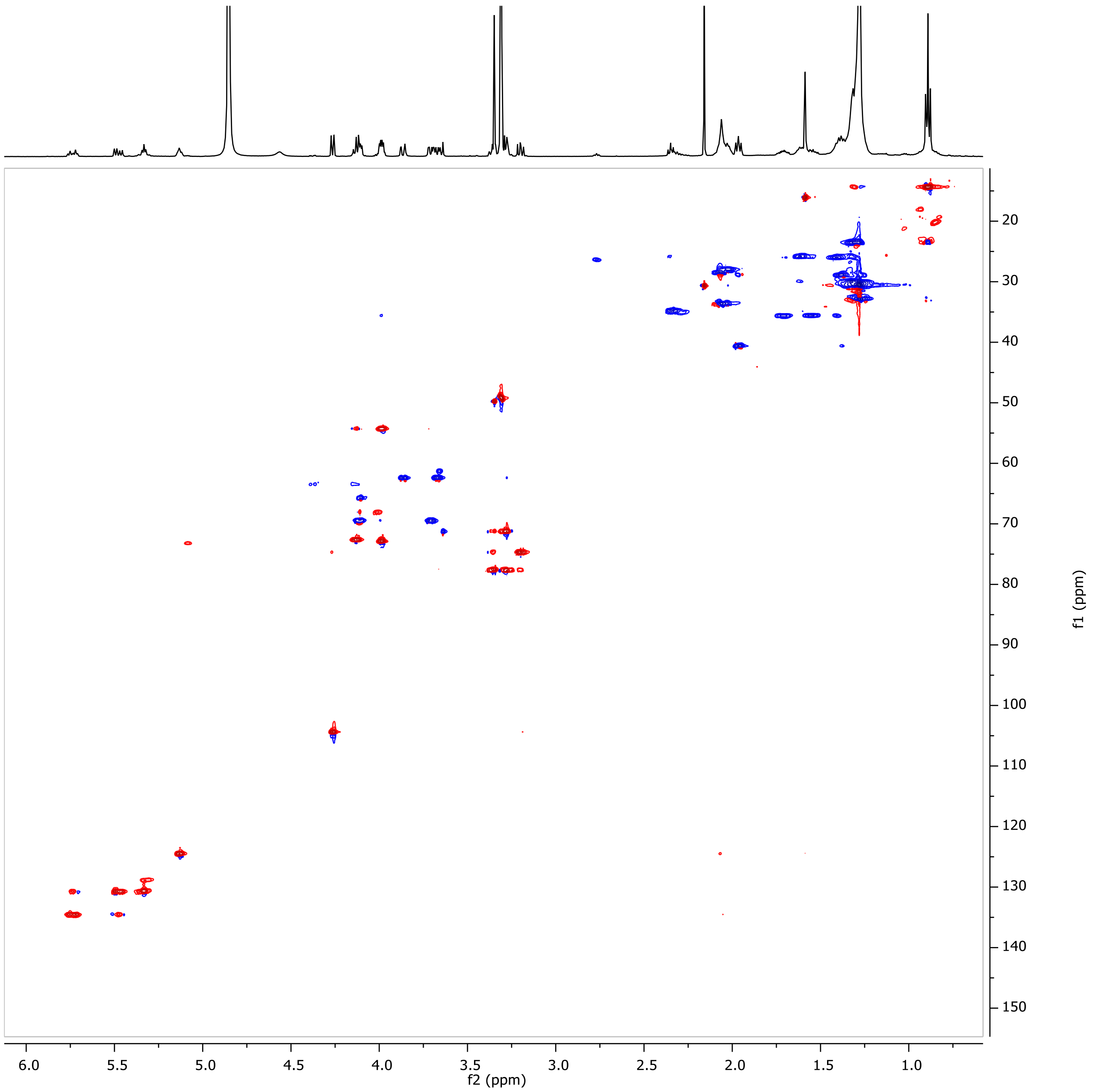

Figure S71. HSQC spectrum of **10** in methanol-d_4 at 500 MHz.

Generic Display Report

Analysis Info

Analysis Name	S:\DATA\MaXis\ESE22_Ellen Sepanian\23_01\MyNe-02-11-02-X-MeOH-F7_15_01_11211.d	Acquisition Date	18.01.2023 14:26:13
Method	pos_säure_10000_screening_ms_100_2500_line.m	Operator	ate06
Sample Name	MyNe-02-11-02-X-MeOH-F7	Instrument	maXis
Comment	Screening01<br>Waters Acquity UPLC BEH C_18 1,7um 2.1x50mm		

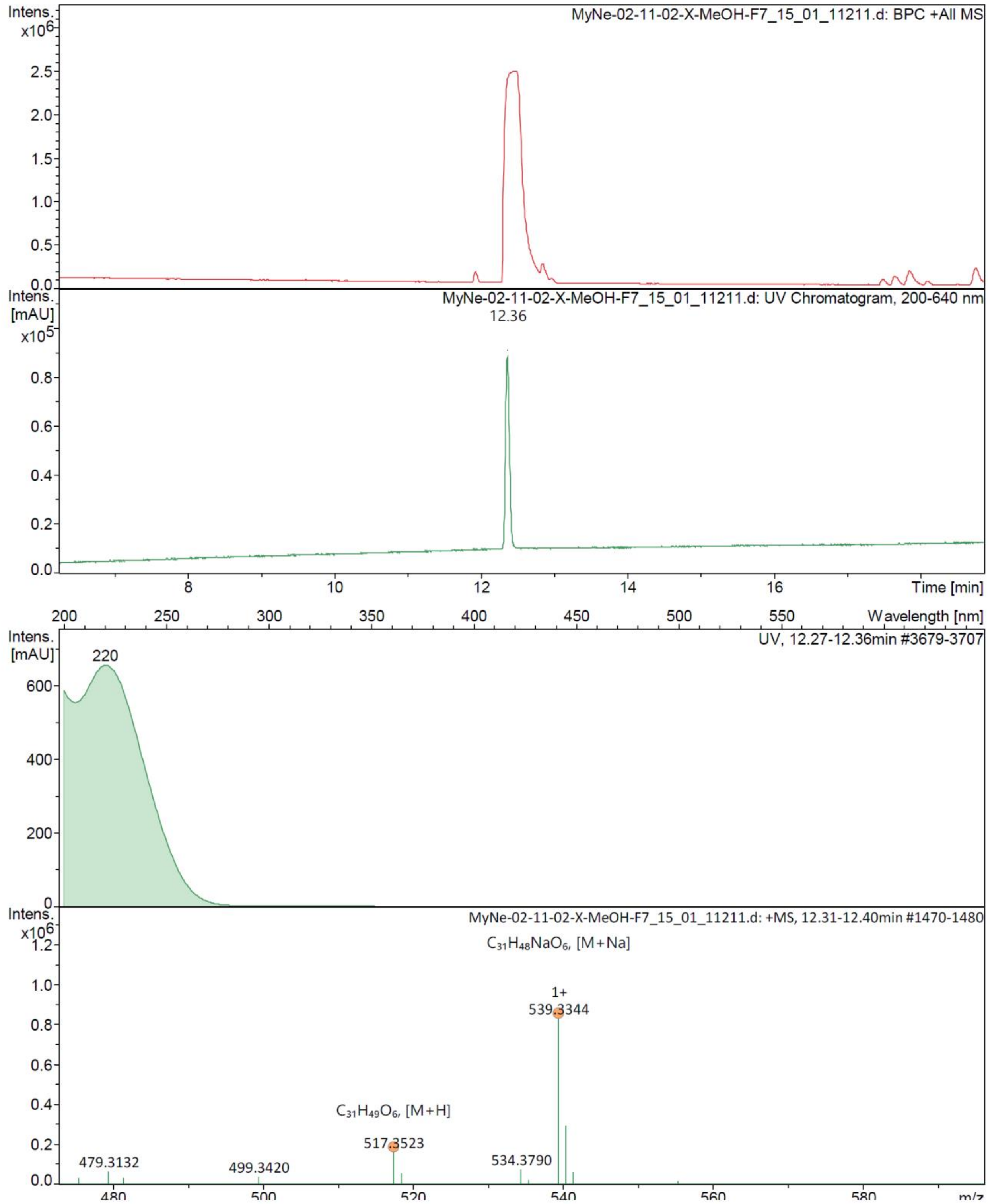

Bruker Compass DataAnalysis 4.4 printed: 09.06.2023 20:52:33 by: sel22 Page 1 of 1

Figure S72. HRESIMS of **11**.

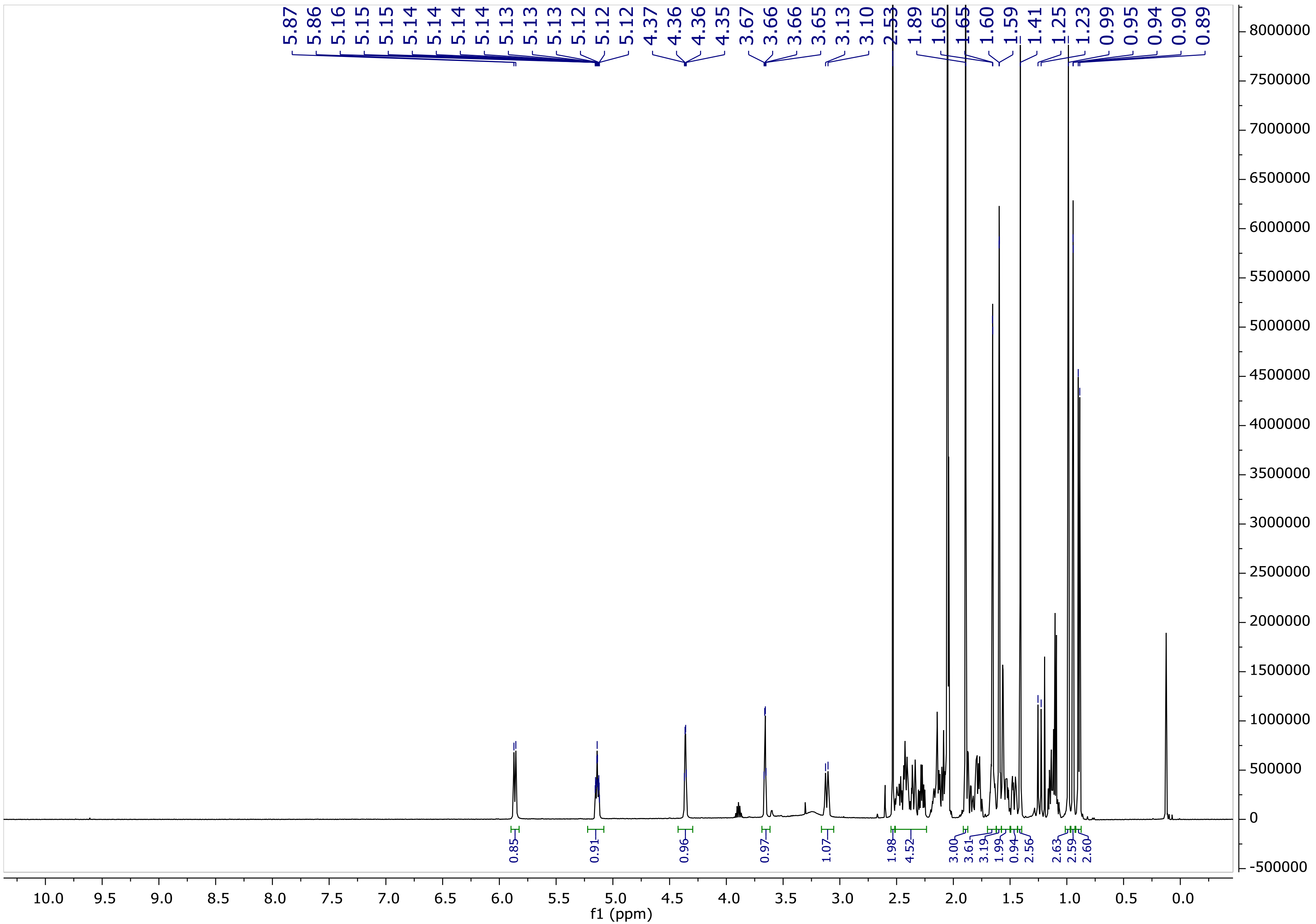

Figure S73. ^{1}H NMR spectrum of **11** in acetone-d_6 at 500 MHz.

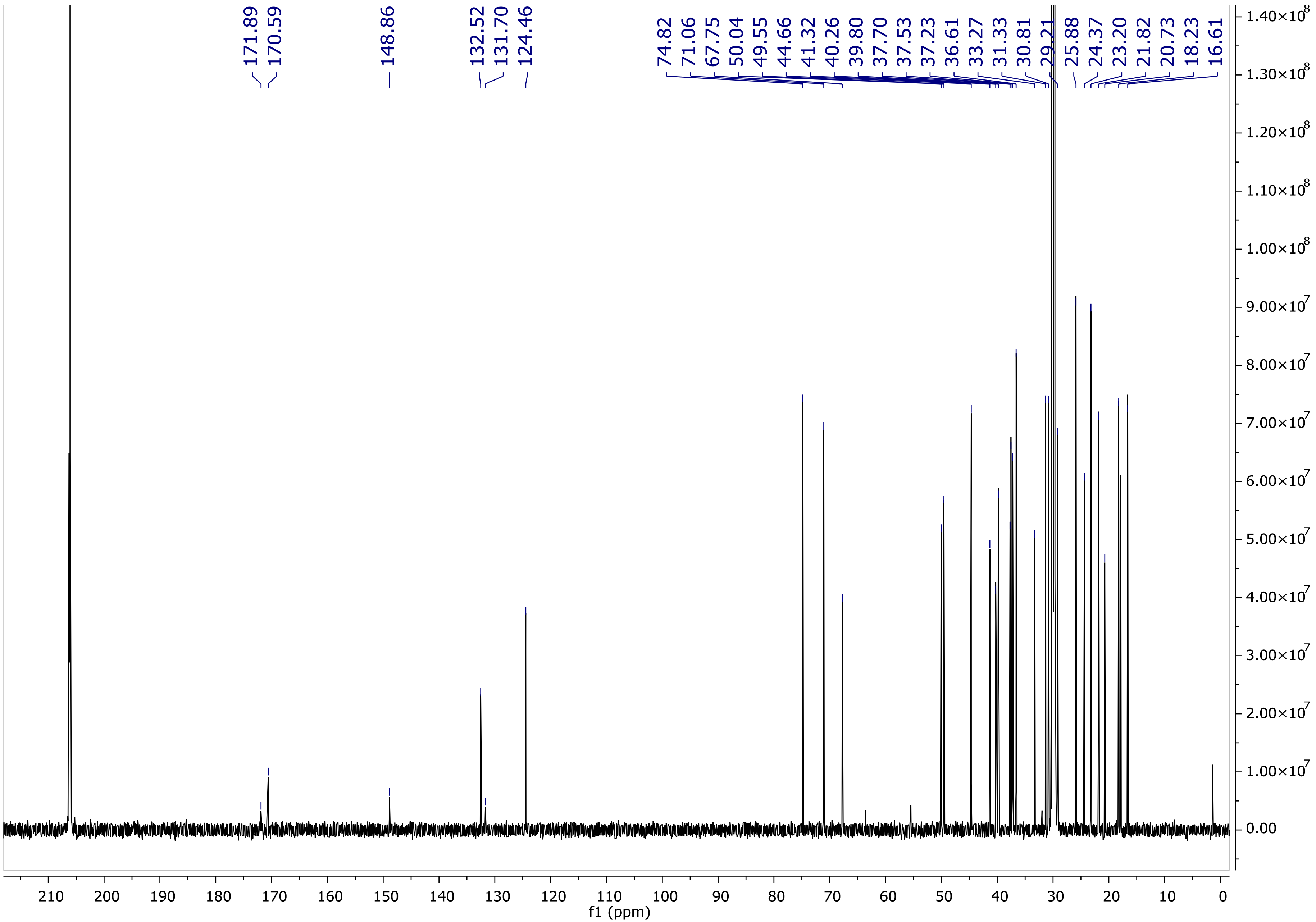

Figure S74. ^{13}C NMR spectrum of **11** in acetone-d_6 at 125 MHz.

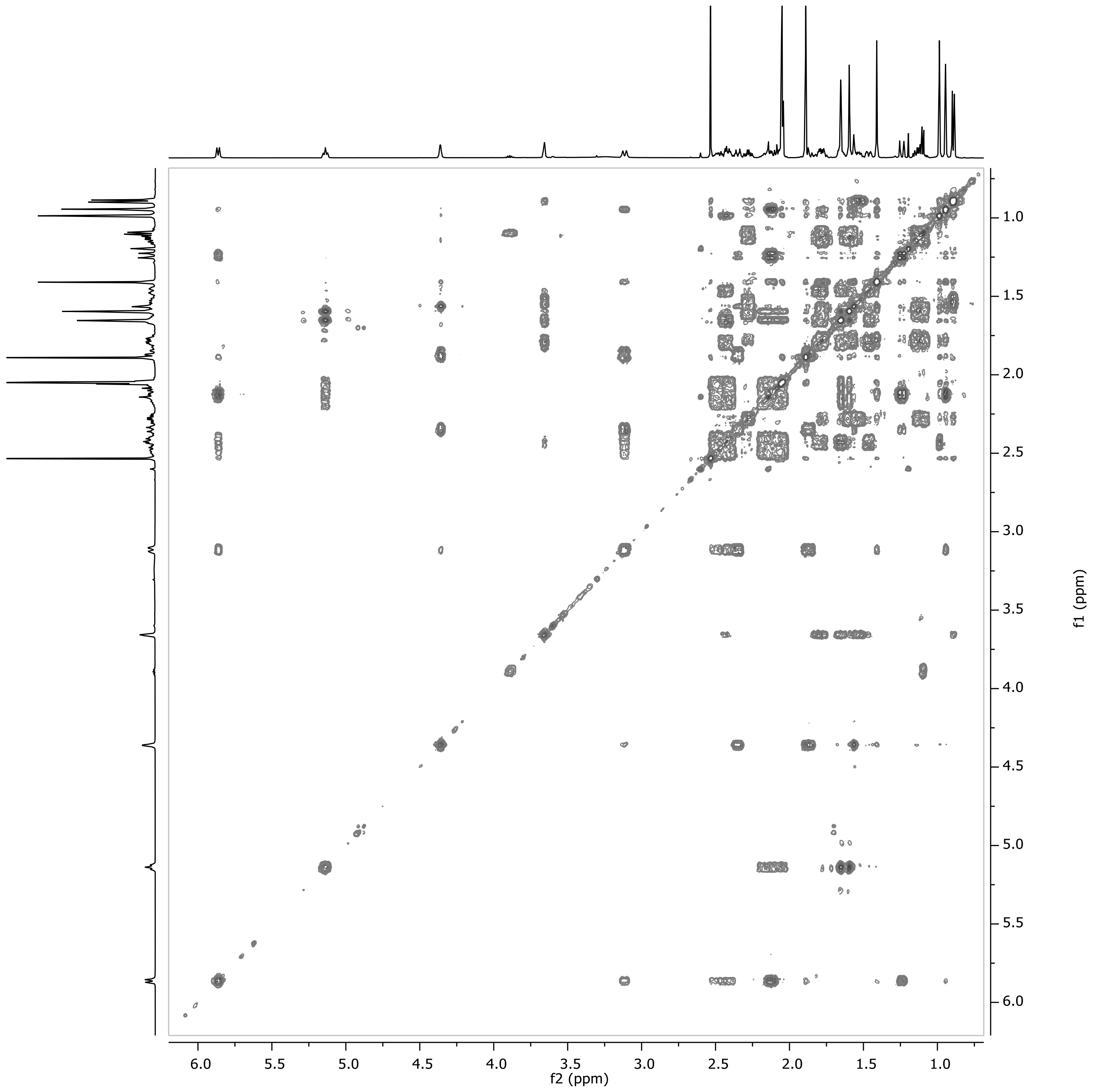

Figure S75. ^{1}H-^{1}H COSY spectrum of **11** in acetone-d_6 at 500 MHz.

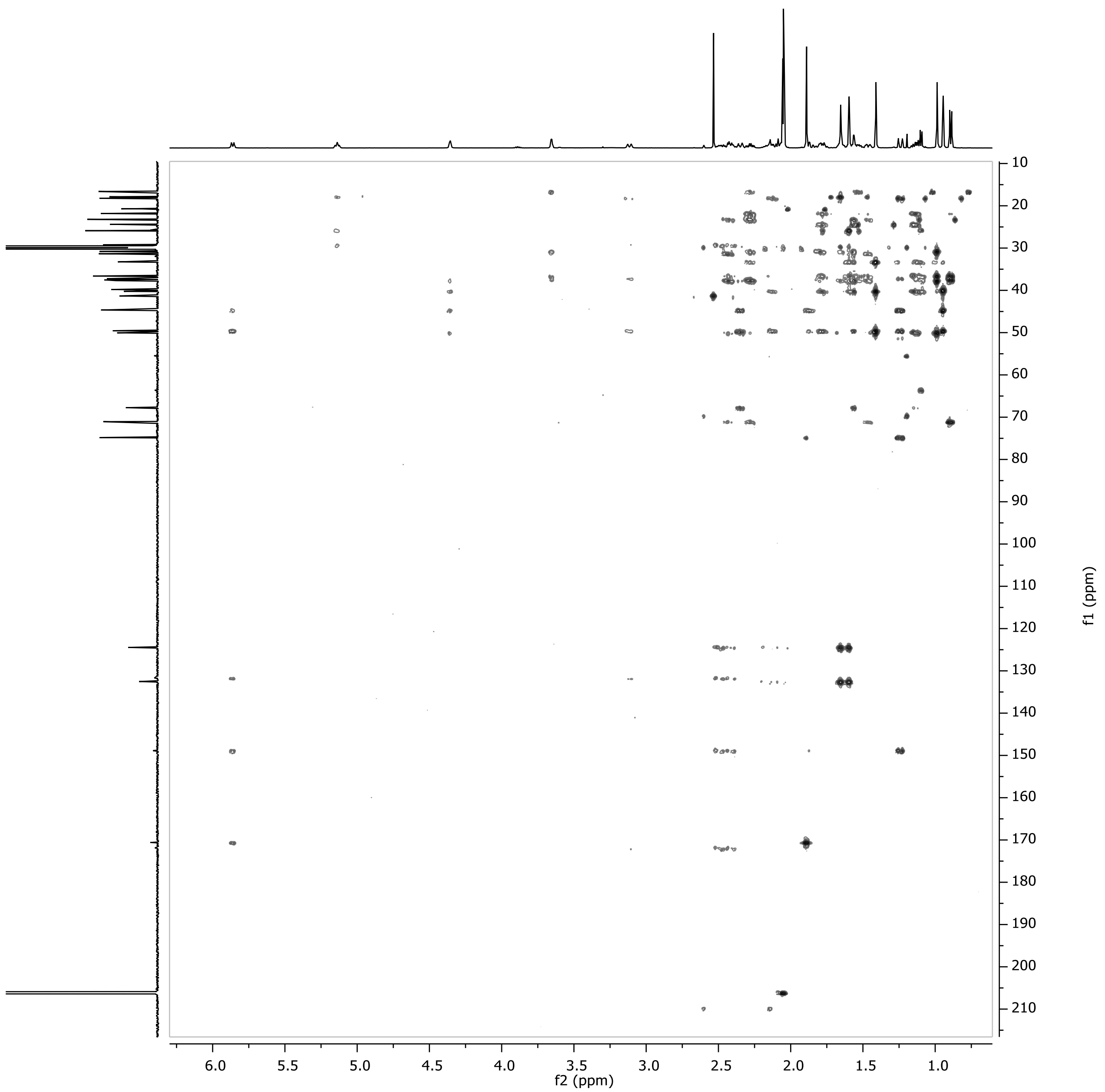

Figure S76. HMBC spectrum of **11** in acetone-d_6 at 500 MHz.

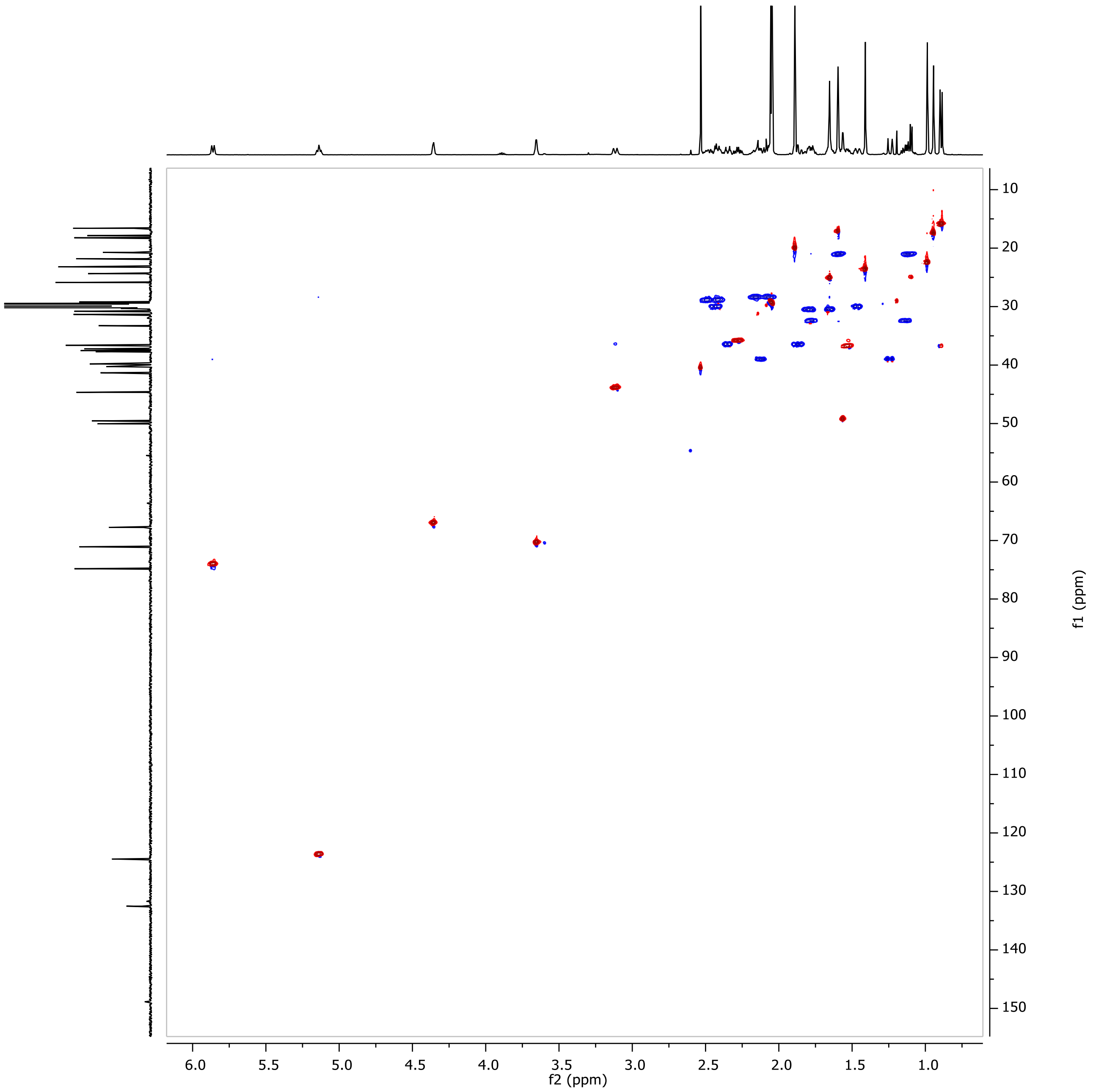

Figure S77. HSQC spectrum of **11** in acetone-d_6 at 500 MHz.

Generic Display Report

Analysis Info

Analysis Name	S:\DATA\MaXis\ESE22_Ellen Sepanian\23_01\MyNe-02-11-02-X-MeOH-F6_14_01_11210.d	Acquisition Date	18.01.2023 13:55:15
Method	pos_säure_10000_screening_ms_100_2500_line.m	Operator	ate06
Sample Name	MyNe-02-11-02-X-MeOH-F6	Instrument	maXis
Comment	Screening01<br>Waters Acquity UPLC BEH C_18 1,7um 2.1x50mm		

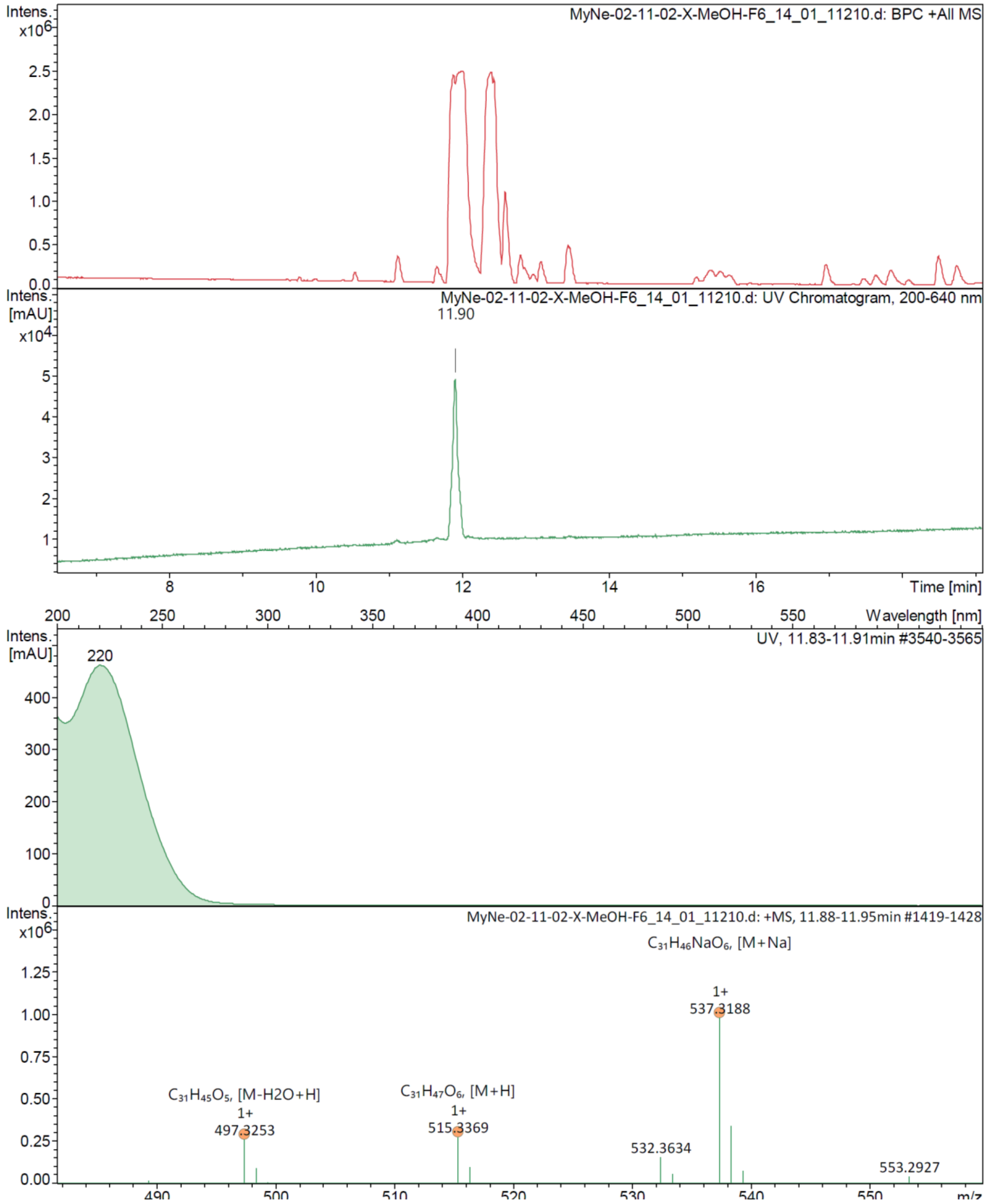

Bruker Compass DataAnalysis 4.4 printed: 09.06.2023 21:05:19 by: sel22 Page 1 of 1

Figure S78. HRESIMS of **12**.

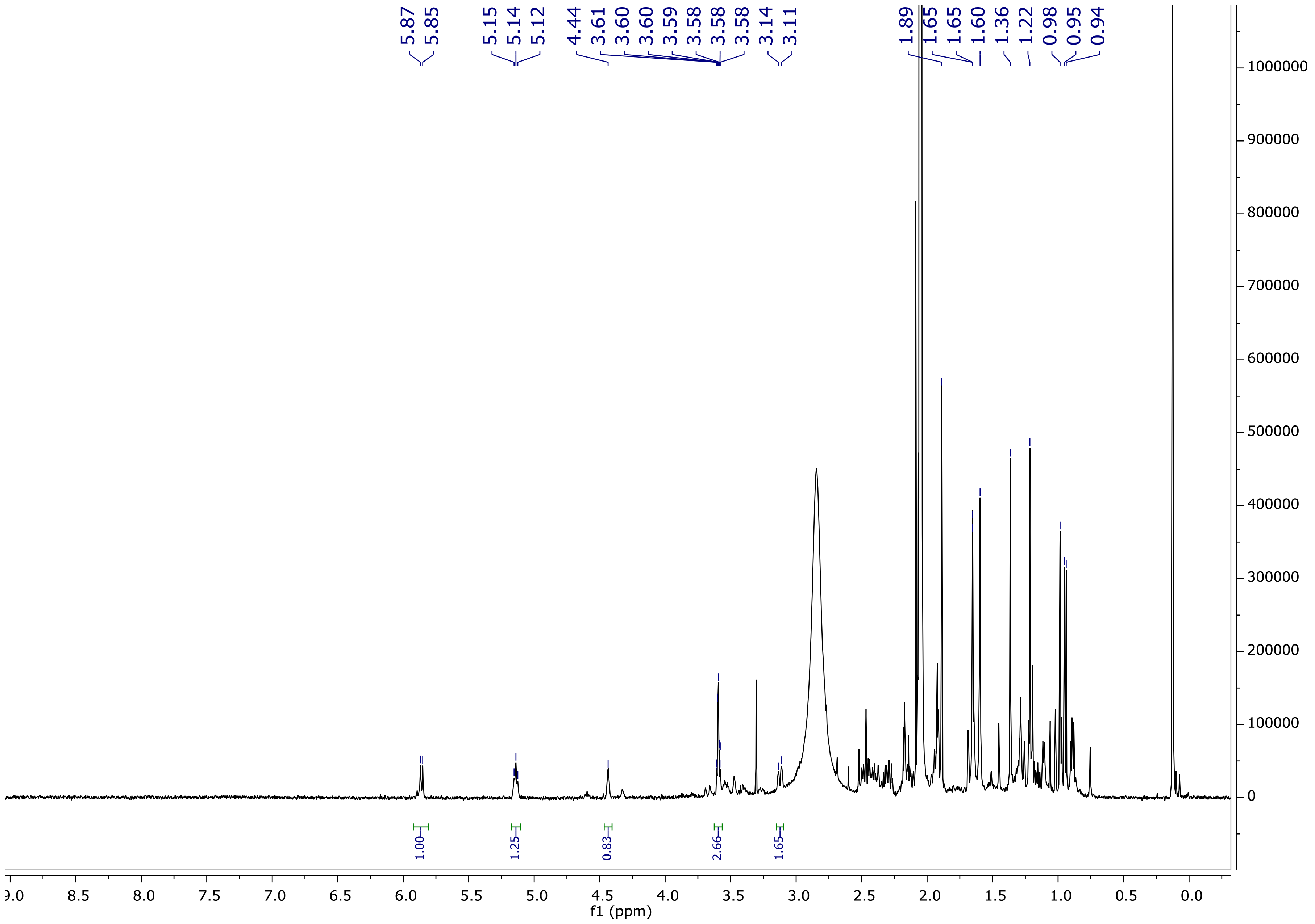

Figure S79. ^{1}H NMR spectrum of **12** in acetone-d_6 at 500 MHz.

Generic Display Report

**Analysis Info**		Acquisition Date	24.11.2022 13:32:39
Analysis Name	S:\DATA\MaXis\ESE22_Ellen Sepanian\22_11\MyNe-01-11-06+07-MeOH-F7-F2_73_01_11104.d		
Method	pos_säure_10000_screening_ms_100_2500_line.m	Operator	ate06
Sample Name	MyNe-01-11-06+07-MeOH-F7-F2	Instrument	maXis
Comment	Screening01<br>Waters Acquity UPLC BEH C_18 1,7um 2.1x50mm		

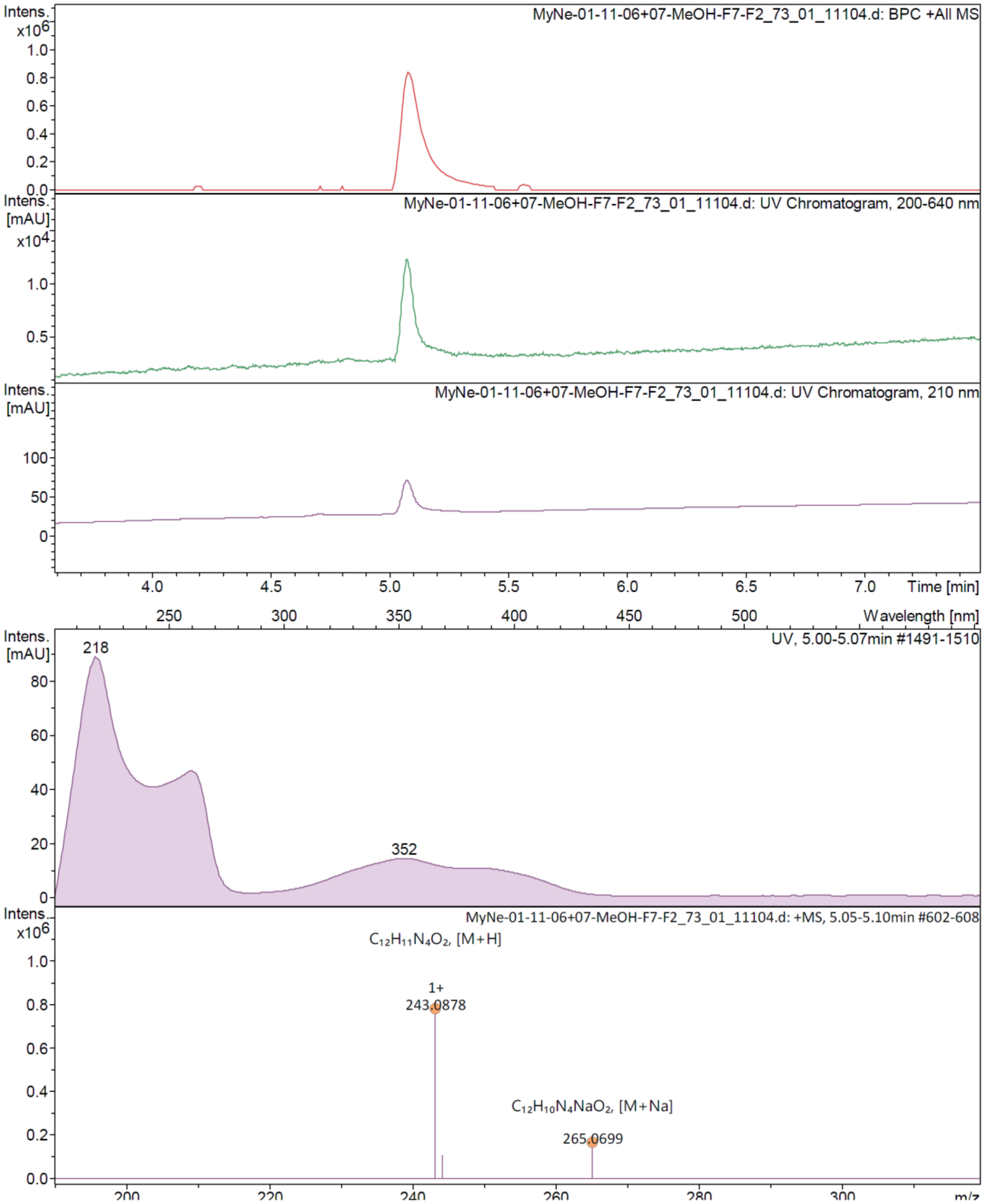

Figure S80. HRESIMS of **13**.

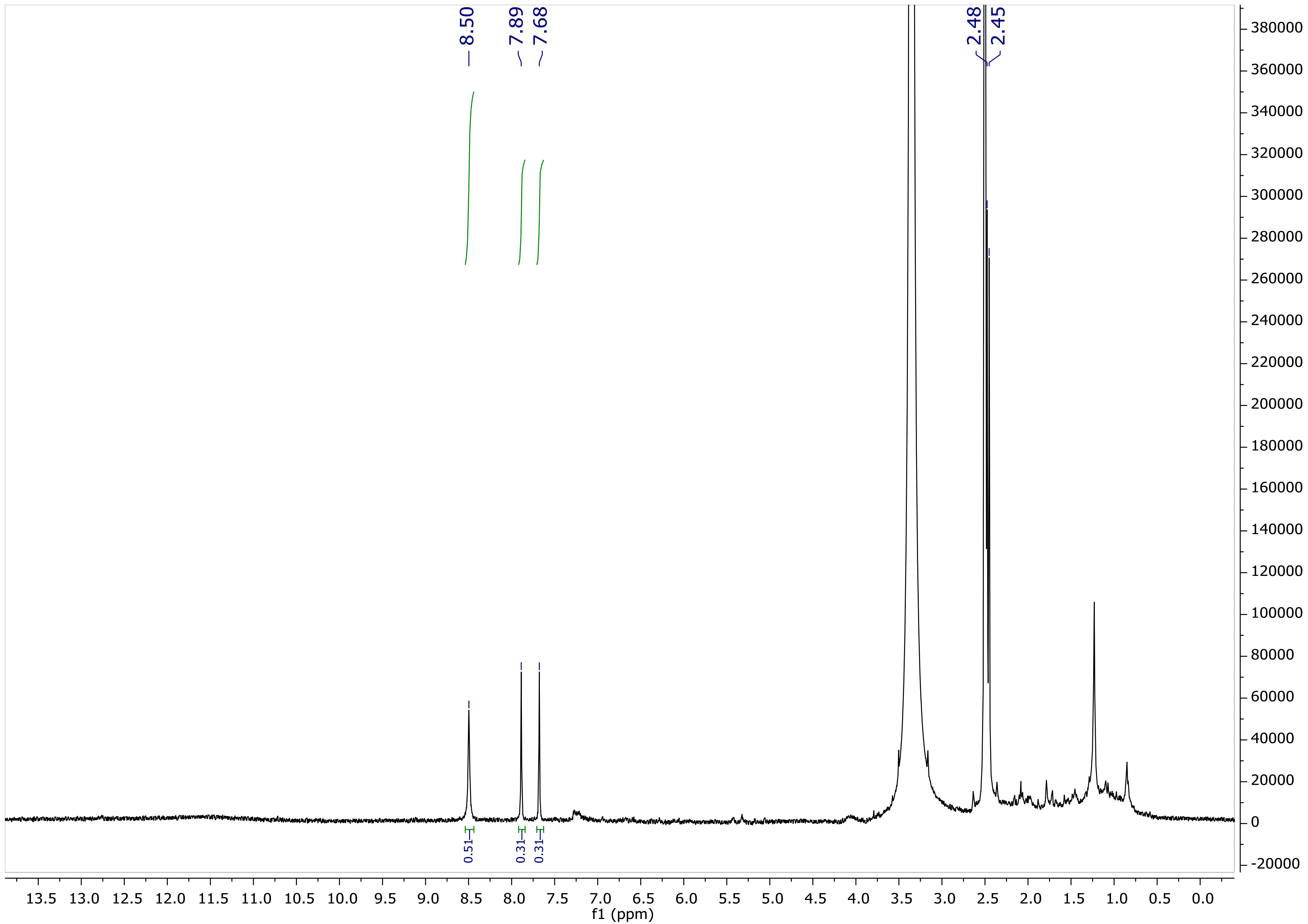

Figure S81. ^{1}H NMR spectrum of **13** in DMSO-d_6 at 500 MHz.

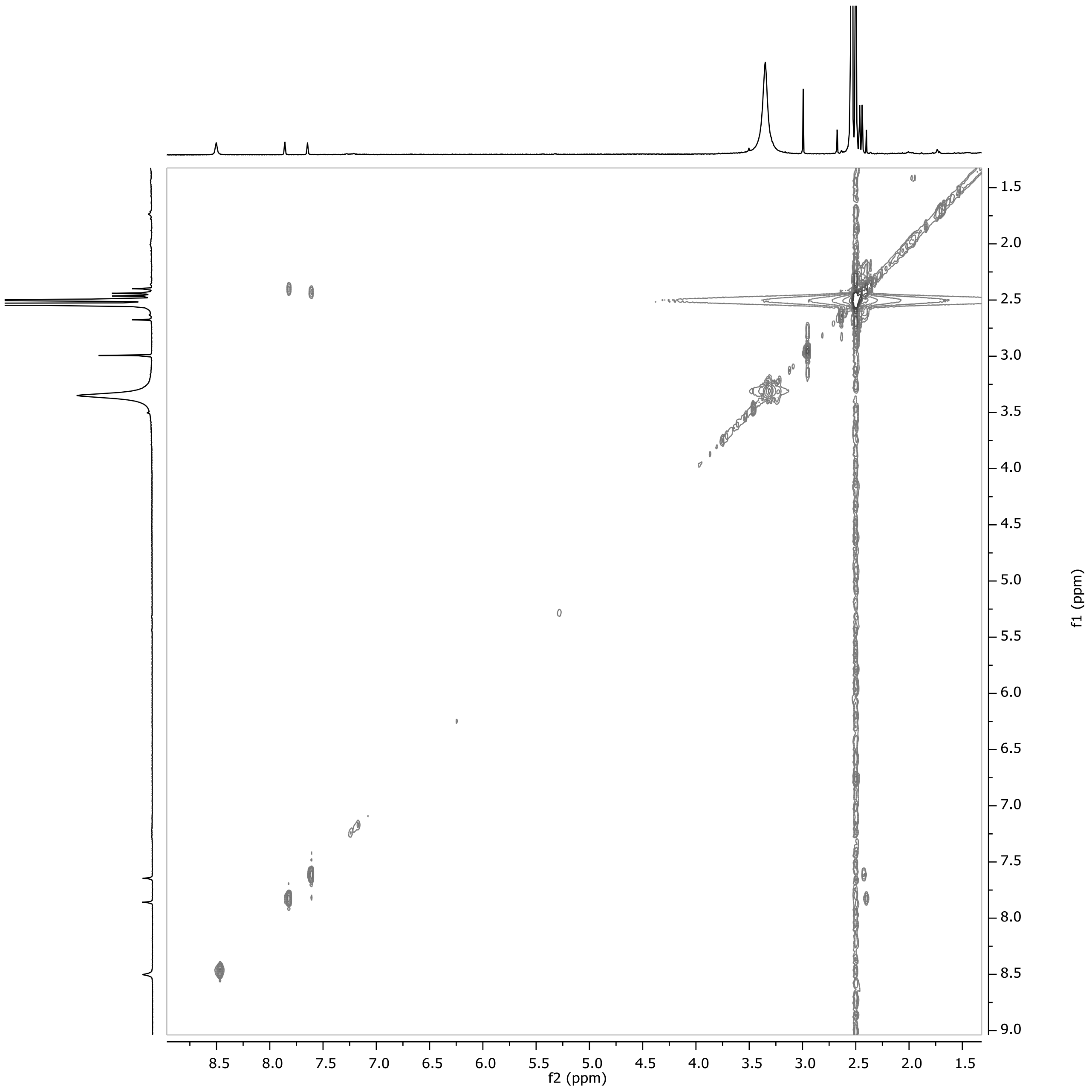

Figure S82. ^{1}H-^{1}H COSY spectrum of **13** in DMSO-d_6 at 500 MHz.

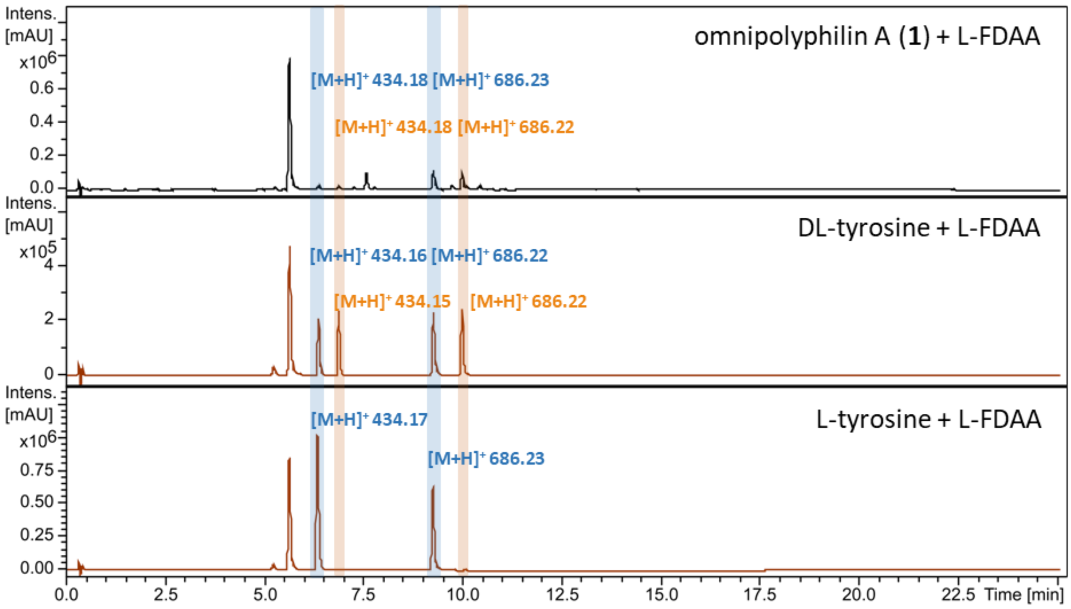

Figure S83. LC-ESI-MS spectra omnipolyphilin A, DL-tyrosine and L-tyrosine + FDAA. Top-bottom: omnipolyphilin A + FDAA, DL-tyrosine + FDAA, L-tyrosine + FDAA. $[M+H]^+$ of the mono and bis adducts of FDAA with L-tyrosine in blue and D-tyrosine in orange are displayed. UV-chromatogram at 190-600 nm is shown. Bars are indicating identical MS-Peaks (blue L-tyrosine and orange D-tyrosine).

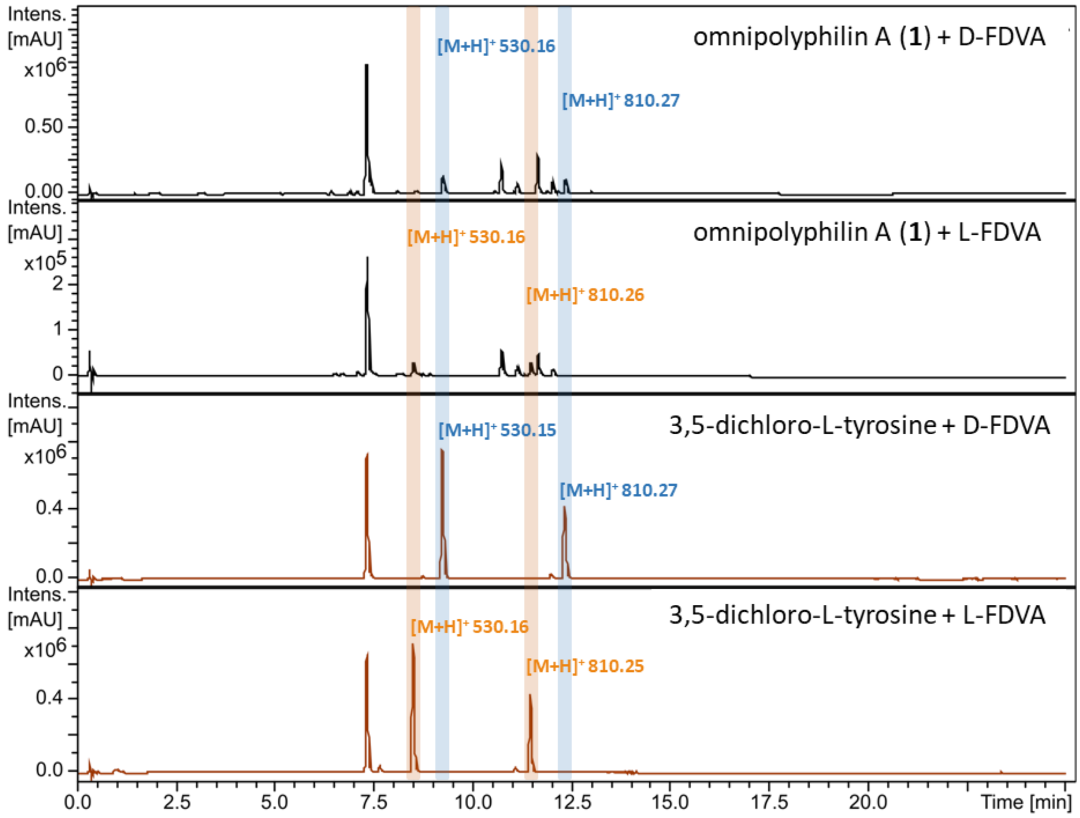

Figure S84. LC-ESI-MS spectra omnipolyphilin A, DL-tyrosine and L-tyrosine with L-FDVA and D-FDVA. Top-bottom: omnipolyphilin A + D-FDVA, omnipolyphilin A + L-FDVA, 3,5-dichloro-L-tyrosine D-FDVA, 3,5-dichloro-L-tyrosine L-FDVA. $[M+H]^+$ of the mono and bis adducts of D-FDVA in blue and L-FDVA in orange with 3,5-dichloro-L-tyrosine are displayed. UV-chromatogram at 190-600 nm is shown. Bars are indicating identical MS-Peaks (blue D-FDVA and orange L-FDVA).

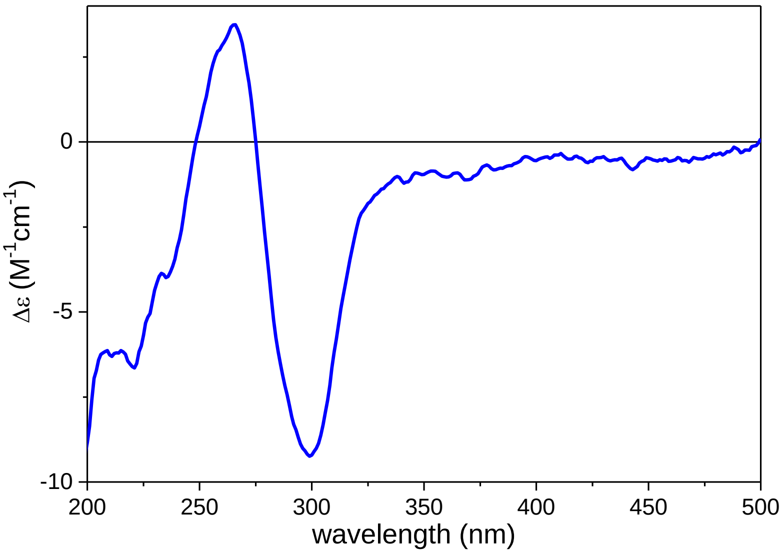

Figure S85. Experimental ECD spectrum of compound **3** in MeOH.

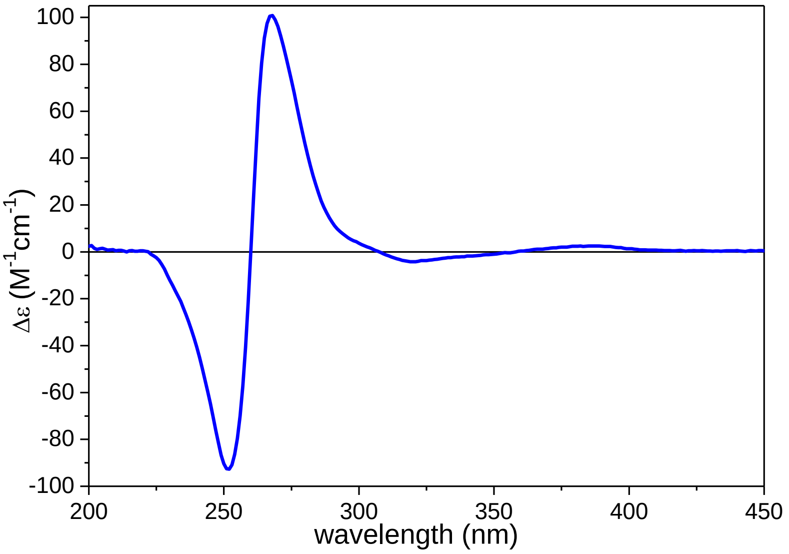

Figure S86. Experimental ECD spectrum of compound **4** in MeOH.

Table S1. Chemical shift differences between the (*S*)-MTPA and (*R*)-MTPA esters of **4**.

pos.	Chemical shift (δ_H, in pyridine-d_5, 700 MHz)			**δ(*S*) – δ(*R*)**
	Talaroderxine D (**4**)	(*S*)-MTPA ester	(*R*)-MTPA ester	
H_2-13	*α* 1.4438	*α* 1.4512	*α* 1.3719	+0.0793
	β 1.5829	*β* 1.5693	*β* 1.4516	+0.1177
H-14	3.9160	5.1481	5.1345	+0.0136
H_3-15	1.2900	1.1511	1.2154	-0.0643

Antimicrobial activity assay

The antimicrobial effects were assessed by determining the minimum inhibitory concentration (MIC) using 96-well round-bottom plates. Compounds **1**, **3**, **4**, **5**, **8**, **10** and **11** were subjected to testing against a panel of five fungi, namely *Candida albicans* [DSM 1665], *Mucor hiemalis* [DSM 2656], *Rhodotorula glutinis* [DSM 10134], *Schizosaccharomyces pombe* [DSM 70572], and *Wickerhamomyces anomalus* [DSM 6766], as well as bacteria including *Bacillus subtilis* [DSM 10], *Mycobacterium smegmatis* [ATCC 700084], and *Staphylococcus aureus* [DSM 346] (Gram-positive), as well as *Acinetobacter baumannii* [DSM 30008], *Chromobacterium violaceum* [DSM 30191], *Escherichia coli* [DSM 1116], and *Pseudomonas aeruginosa* [PA14] (Gram-negative). A cell suspension of most bacteria was prepared in Mueller-Hinton Broth (SN X927.1, Carl Roth GmbH, Karlsruhe, Germany) and adjusted to an optical density (OD) of 0.01 at 600 nm. The suspension of *Mycobacterium smegmatis* was cultured in 27H9 + ADC (Middlebrook 7H9 Broth Base + Middlebrook ADC Growth Supplement, SN M0678 + M0553, Merck, Darmstadt, Germany) and adjusted to an OD of 0.1 at 548 nm. All the fungal strains were cultivated in MYC medium (containing 1% bacto peptone, 1% yeast extract, 2% glycerol, pH 6.3) and adjusted to an OD of 0.1 at 548 nm. Subsequently, 150 µL of cell suspension was filled into all wells of a 96-well round-bottom plate. In the first row A, an additionally 130 µL of cell suspensions plus 20 µL of the test compounds (1 mg mL^{-1}) in methanol were added. 20 µL of methanol served as the negative control. Different positive controls were employed depending on the test organisms: nystatin (1 mg mL^{-1}) against fungi; oxytetracycline (0.1 mg mL^{-1}) against *B. subtilis*; gentamycin (0.1 mg mL^{-1}) against *S. aureus*, *E. coli*, *C. violaceum*, *P. aeruginosa*; ciprofloxacin (0.25 mg mL^{-1}) against *A. baumannii*; kanamycin (0.1 mg mL^{-1}) against *M. smegmatis* were used.

Subsequently, starting from row A, 150 µL of the cell suspension + compound were transferred to the next row, mixed and 150 µL were transferred to the following row, creating a serial dilution from row A to H. The remaining 150 µL after the last row were discarded. This resulted in a concentration range from 66.7 µg mL^{-1} in row A to 0.52 µg mL^{-1} in row H. The plates were incubated at 800 rpm on a microplate shaker overnight with the temperature selected based on the specific requirements of the microorganisms: 30°C for most of the tested organisms, and 37°C for *M. smegmatis*, *E. coli*, and *P. aeruginosa*. The recorded MIC is the lowest concentration of the compounds that prevented visible growth.

Cytotoxicity (MTT) assay

The cytotoxicity of compounds **1**, **3**, **4**, **5**, **8**, **10** and **11** was assessed against KB3.1 (human endocervical adenocarcinoma), L929 (mouse fibroblasts), A431 (human squamous carcinoma), A549 (human lung carcinoma), PC-3 (human prostate adenocarcinoma) and MCF-7 (human breast adenocarcinoma) cell lines, using a 96-well plate. The compounds were prepared as described above, and epothilone B served as the positive control. The compounds were tested in a concentration range of 37 to 0.6×10^{-3} µg mL^{-1} and incubated at 37°C with 10% CO_2 in Gibco™ Dulbecco’s Modified Eagle Medium (SN 61965026, Thermo Fisher Scientific, Waltham, MA, USA) supplemented with 10% Fetal Bovine Serum (SN 10500064, Thermo Fisher Scientific). 3-(4,5-Dimethyl-2-thiazolyl)-2,5-diphenyl-2*H*-tetrazolium bromide (MTT, M2128, Sigma-Aldrich, Deisenhofen, Germany) was sued for staining after 5 days of incubation. Living cells are still able to convert this dye to its purple derivative. A microplate reader at 595 nm was used to measure the intensity of the purple derivative, relative to cells without additives (considered 100% viability), for each concentrations. The results were used to determine the percentage of cell viability. Finally, the half-maximum inhibitory concentration IC_{50} in µM was determined.

Table S2. Cytotoxicity (IC_{50}) of tested compounds.

	IC_{50} (µM)							Positive Control
Test Cell Line	**1**	**3**	**4**	**5**	**8**	**10**	**11**	Epothilone B (nM)
L929 (murine)	n.a	n.a	n.a	1.19	n.a	n.a	n.a	0.65
KB3.1 (cervix)	n.a	n.a	2.01	10.32	n.a	n.a	n.a	0.17
PC-3 (prostate)	n.d	n.d	9.75	8.73	n.d	n.d	n.d	0.09
MCF-7 (breast)	n.d	n.d	1.16	0.068	n.d	n.d	n.d	0.07
A431 (skin)	n.d	n.d	1.19	2.38	n.d	n.d	n.d	0.06
A549 (lung)	n.d	n.d	26.3	2.06	n.d	n.d	n.d	0.05

n.a.: No activity, n.d.: Not determined.

Table S3. Antimicrobial activity (MIC) of tested compounds.

Test Microorganism	MIC (µg mL^{-1})							Antibiotic (µg mL^{-1})
	**1**	**3**	**4**	**5**	**8**	**10**	**11**	
Staphylococcus aureus	n.i	n.i	8.3	66.6	n.i	n.i	8.3	0.21^{G}
Escherichia coli	n.i	n.i	n.i	n.i	n.i	n.i	n.i	0.42^{G}
Bacillus subtilis	n.i	n.i	2.1	0.52	n.i	n.i	1.04	16.6^{O}
Pseudomonas aeruginosa	n.i	n.i	n.i	n.i	n.i	n.i	n.i	0.21^{G}
Wickerhamomyces anomalus	n.i	n.i	n.i	n.i	n.i	n.i	n.i	16.6^{N}
Candida albicans	n.i	n.i	n.i	n.i	n.i	n.i	n.i	8.3^{N}
Acinetobacter baumanii	n.i	n.i	n.i	n.i	n.i	n.i	n.i	0.52^{C}
Chromobacterium violaceum	n.i	n.i	n.i	n.i	n.i	n.i	n.i	1.70^{G}
Schizosaccharomyces pombe	n.i	n.i	n.i	n.i	n.i	n.i	n.i	8.30^{N}
Mucor hiemails	n.i	n.i	n.i	n.i	n.i	n.i	n.i	8.30^{N}
Rhodotorula glutinis	n.i	n.i	n.i	n.i	n.i	n.i	n.i	4.20^{N}
Mycobacterium smegmatis	n.i	n.i	n.i	n.i	n.i	n.i	66.6	1.70^{K}

n.a.: No activity. n.i.: No inhibition up to 67 µg/mL. n.d.: Not determined.
G: Gentamycin; O: Oxytetracycline; N: Nystatin; C: Ciprofloxacin; K: Kanymycin.

Table S4. Nematicidal activity of tested compounds against *Caenorhabditis elegans*.

Tested Organism	Conc. [μg mL⁻¹]	Mortality rate (%)							
		**1**	**4**	**5**	**8**	**10**	**11**	Negative Control	Ivermectin
Caenorhabditis elegans N21	100	11	14	16	12	69	1	8.5±2.5	n.d.
	50	10	11	15	10	20	1		n.d.
	10	9	11	10	12	13	9		n.d.
	1	n.d.	n.d.	n.d.	n.d.	n.d.	n.d.		98

n.a.: No activity. n.d.: Not determined.

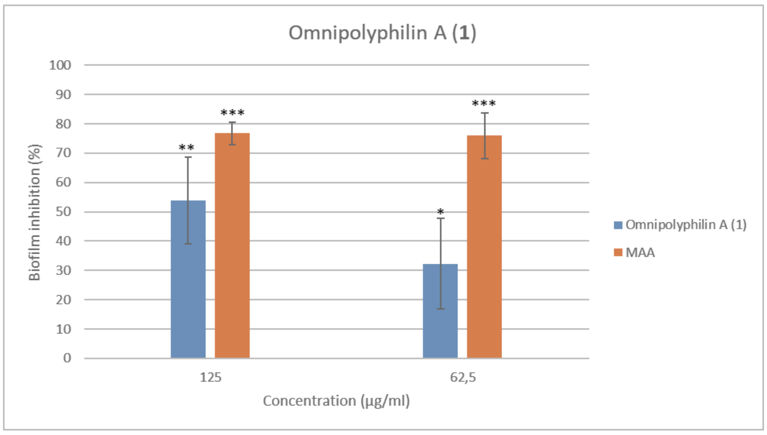

Figure S87. Effects on the biofilm formation of *S. aureus* after 24 h treatment with omnipolyphilin A (**1**).

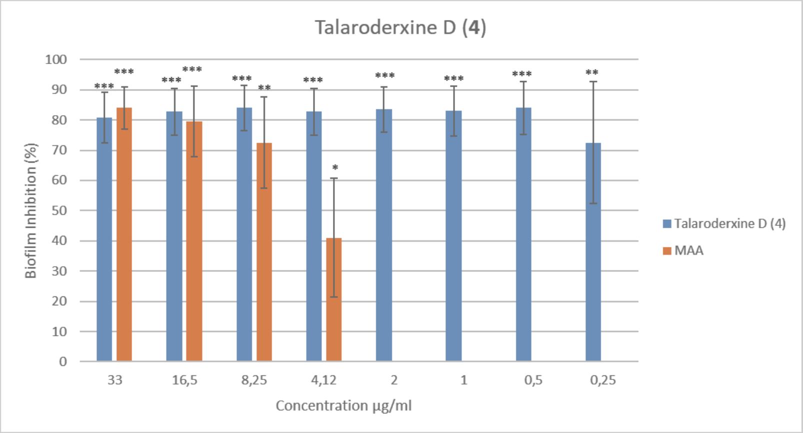

Figure S88. Effects on the biofilm formation of *S. aureus* after 24 h treatment with talaroderxine D (**4**).

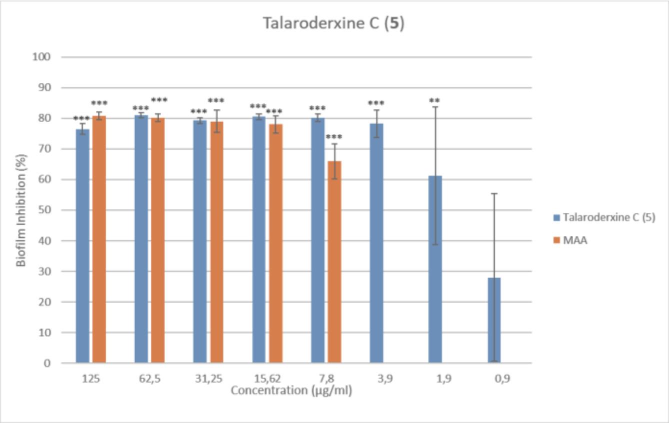

Figure S89. Effects on the biofilm formation of *S. aureus* after 24 h treatment with talaroderxine C (**5**).

Microporenic acid A (MAA) was used as positive control. Methanol was used as solvent control and taken as 100%. Error bars indicate standard deviation of duplicates in two biological repeats; p values: * $p < 0.05$, ** $p < 0.01$, *** $p < 0.001$.

Table S5. Inhibition of biofilm formation of S. *aureus* by omnipolyphilin A (**1**), talaroderxines D (**4**) and C (**5**) at different concentrations.

**Compound**	**Tested Strain**	**Biofilm Inhibition [% ± SD]**
Omnipolyphilin A (**1**)	*Staphylococcus aureus* (DSM 1104)	53 ± 14 (125 µg/ml)<br>32 ± 15 (62.5 µg/ml)
Talaroderxine D (**4**)	*Staphylococcus aureus* (DSM 1104)	83 ± 7 (2 µg/ml)<br>83 ± 8 (1 µg/ml)<br>84 ± 8 (0.5 µg/ml)<br>72 ± 20 (0.25 µg/ml)
Talaroderxine C (**5**)	*Staphylococcus aureus* (DSM 1104)	80 ± 1 (15.62 µg/ml)<br>80 ± 1 (7.8 µg/ml)<br>78 ± 4 (3.9 µg/ml)<br>61 ± 22 (1.9 µg/ml)<br>28 ± 27 (0.9 µg/ml)

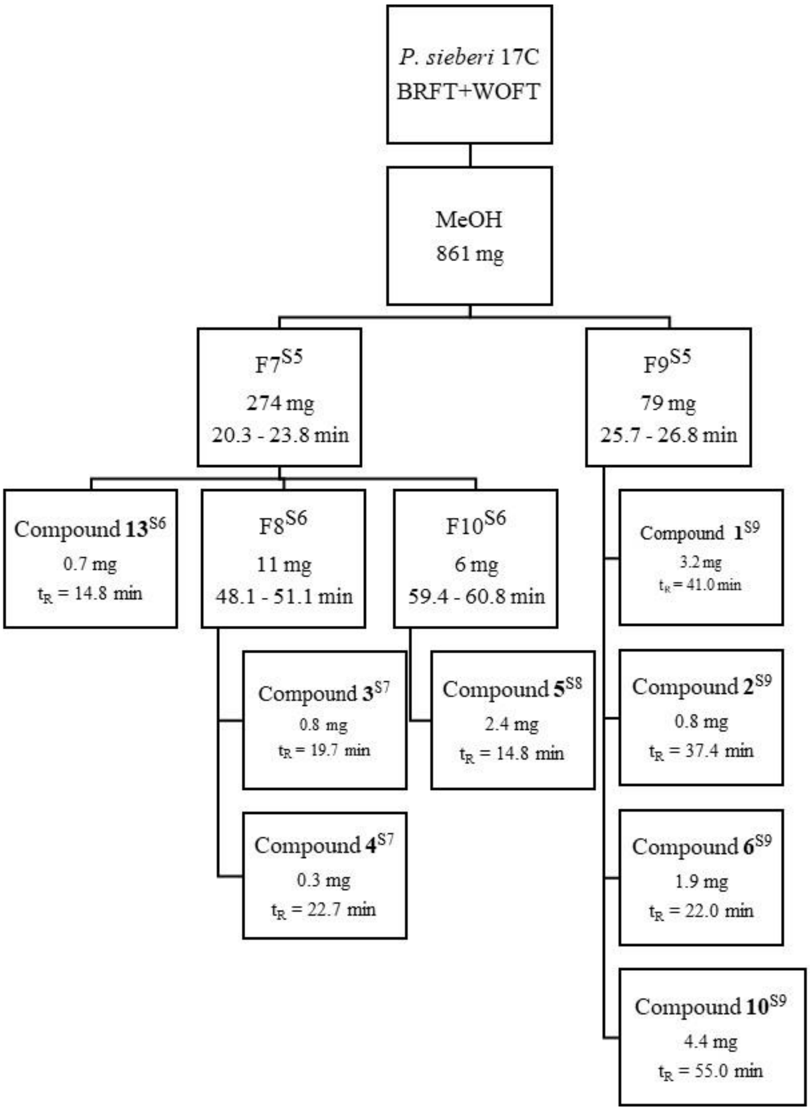

[S5-S9]: Tables S5-S9 with the separation parameters of indicated fractions.

Figure S90. Separation scheme of the isolated compounds **1-6**, **10** and **13**.

Table S6. Separation parameters of *P. sieberi* 17C BRFT + WOFT methanol extract.

Parameter	Settings
**System**	Grace Reveleris® X2 flash chromatography system
**Cartridge**	FlashPure ID Silica 24 g cartridge
**Solvent A**	heptane + 0.1% FA
**Solvent B**	58% heptane, 40% TBME, 2% MeOH + 0.1% FA
**Solvent C**	37.5% acetone, 37.5% DCM, 25% MeOH + 0.1% FA
**Flow rate [mL min^{-1}]**	32
**Fraction volume [mL]**	11
**Sample amount [mg]**	861
**Repetitions**	1
**Gradient [t_{min}]**	t_0 = 0% A**B**, t_3 = 0% A**B**, t_{13} = 100% A**B**, t_{18} = 100% A**B**, t_{33} = 100% B**C**, t_{43} = 100% B**C**

Table S7. Separation parameters of fraction F7.

Parameter	Settings
**System**	Büchi Pure C-850 FlashPrep
**Column**	Gemini C18 column (250 × 50 mm, 10 µm, Phenomenex)
**Solvent A**	H_2O + 0.1% FA
**Solvent B**	MeCN + 0.1% FA
**Flow rate [mL min^{-1}]**	50
**Fraction volume [mL]**	17
**Sample amount [mg]**	274
**Repetitions**	1
**Gradient [t_{min}]**	t_0 = 30% B, t_5 = 30% B, t_{15} = 60% B, t_{55} = 90% B, t_{60} = 100% B, t_{70} = 100% B

Table S8. Separation parameters of fraction F8.

Parameter	Settings
**System**	Agilent Technologies 1200 Infinity Series semi-preparative HPLC
**Column**	XBridge BEH C18 column (250 × 10 mm, 5 µm; Waters™)
**Solvent A**	H_2O + 0.1 % FA
**Solvent B**	MeCN + 0.1 % FA
**Flow rate [mL min^{-1}]**	5
**Fraction volume [mL]**	0.5
**Sample amount [mg]**	11
**Repetitions**	3
**Gradient [t_{min}]**	t_0 = 70% B, t_3 = 70% B, t_{26} = 100% B, t_{26} = 100% B, t_{29}= 100% B

Table S9. Separation parameters of fraction F10.

Parameter	Settings
**System**	Agilent Technologies 1200 Infinity Series semi-preparative HPLC
**Column**	XBridge BEH C18 column (250 × 10 mm, 5 µm; Waters™)
**Solvent A**	H_2O + 0.1 % FA
**Solvent B**	MeCN + 0.1 % FA
**Flow rate [mL min^{-1}]**	5
**Fraction volume [mL]**	0.5
**Sample amount [mg]**	6
**Repetitions**	2
**Gradient [t_{min}]**	t_0 = 80% B, t_3 = 80% B, t_{18} = 100% B, t_{35} = 100% B

Table S10. Separation parameters of fraction F9.

Parameter	Settings
**System**	Büchi Pure C-850 FlashPrep
**Column**	Gemini C18 column (250 × 21.2 mm, 10 µm, Phenomenex)
**Solvent A**	H_2O + 0.1% FA
**Solvent B**	MeCN + 0.1% FA
**Flow rate [mL min^{-1}]**	20
**Fraction volume [mL]**	7
**Sample amount [mg]**	79
**Repetitions**	2
**Gradient [t_{min}]**	t_0 = 15% B, t_5 = 15% B, t_{45} = 45% B, t_{50} = 100% B, t_{60} = 100% B

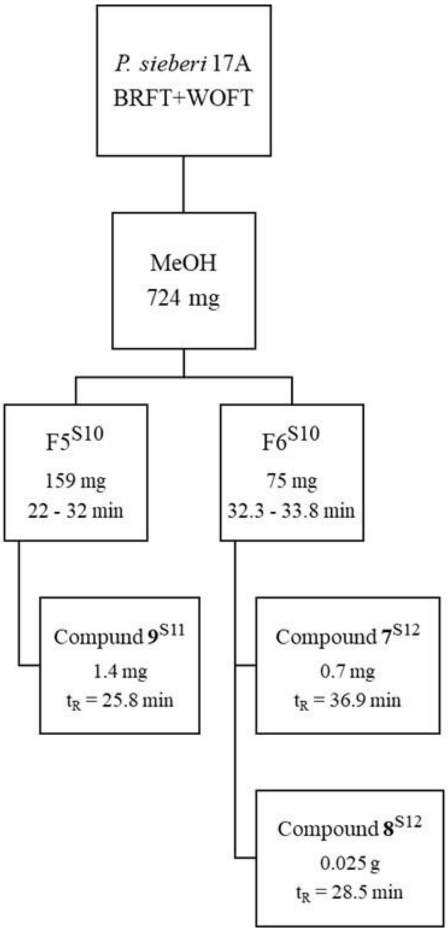

S10-S12: Tables S10-S12 with the separation parameters of indicated fractions.

Figure S91. Separation scheme of the isolated compounds **7-9**.

Table S11. Separation parameters of *P. sieberi* 17A BRFT + WOFT methanol extract.

Parameter	Settings
**System**	Grace Reveleris® X2 flash chromatography system
**Cartridge**	FlashPure ID Silica 40 g cartridge
**Solvent A**	heptane + 0.1% FA
**Solvent B**	58% heptane, 40% TBME, 2% MeOH + 0.1% FA
**Solvent C**	37.5% acetone, 37.5% DCM, 25% MeOH + 0.1% FA
**Flow rate [mL min^{-1}]**	40
**Fraction volume [mL]**	15
**Sample amount [mg]**	724
**Repetitions**	1
**Gradient [t_{min}]**	t_0 = 0% A**B**, t_3 = 0% A**B**, t_{13} = 100% A**B**, t_{18} = 100% A**B**, t_{21} = 0% B**C**, t_{41} = 100% B**C,** t_{46} = 100% B**C**

Table S12. Separation parameters of fraction F5.

Parameter	Settings
**System**	Büchi Pure C-850 FlashPrep
**Column**	Gemini C18 column (250 × 21.2 mm, 10 µm, Phenomenex)
**Solvent A**	H_2O + 0.1% FA
**Solvent B**	MeCN + 0.1% FA
**Flow rate [mL min^{-1}]**	20
**Fraction volume [mL]**	7
**Sample amount [mg]**	159
**Repetitions**	4
**Gradient [t_{min}]**	t_0 = 15% B, t_5 = 15% B, t_{50} = 45% B, t_{55} = 100% B, t_{65} = 100% B

Table S13. Separation parameters of fraction F6.

Parameter	Settings
**System**	Büchi Pure C-850 FlashPrep
**Column**	Gemini C18 column (250 × 21.2 mm, 10 µm, Phenomenex)
**Solvent A**	H_2O + 0.1% FA
**Solvent B**	MeCN + 0.1% FA
**Flow rate [mL min^{-1}]**	20
**Fraction volume [mL]**	7
**Sample amount [mg]**	75
**Repetitions**	1
**Gradient [t_{min}]**	t_0 = 15% B, t_5 = 15% B, t_{35} = 35% B, t_{45} = 100% B, t_{55} = 100% B

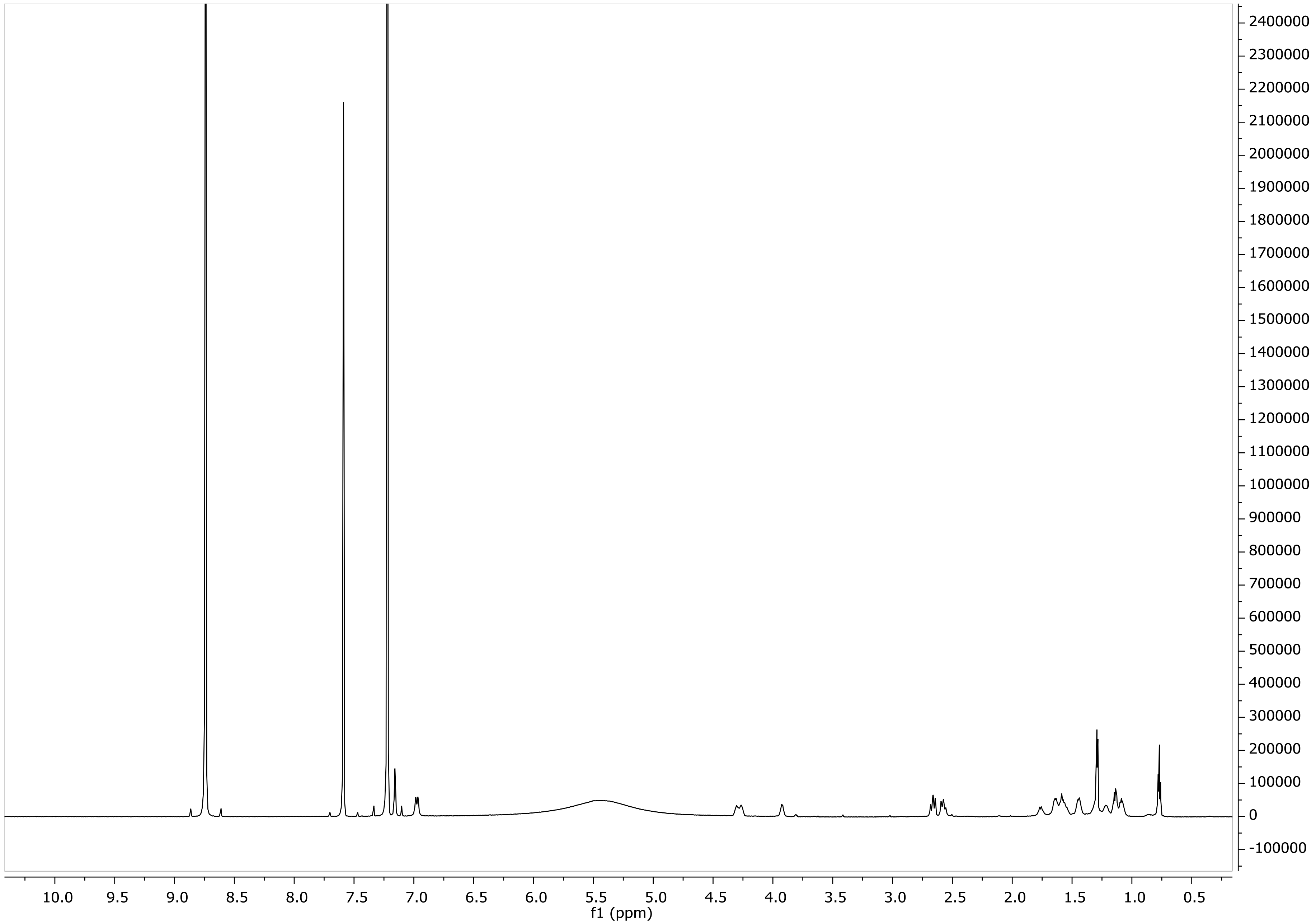

Figure S92. ^{1}H NMR spectrum of **4** in pyridine-d_5 at 700 MHz.

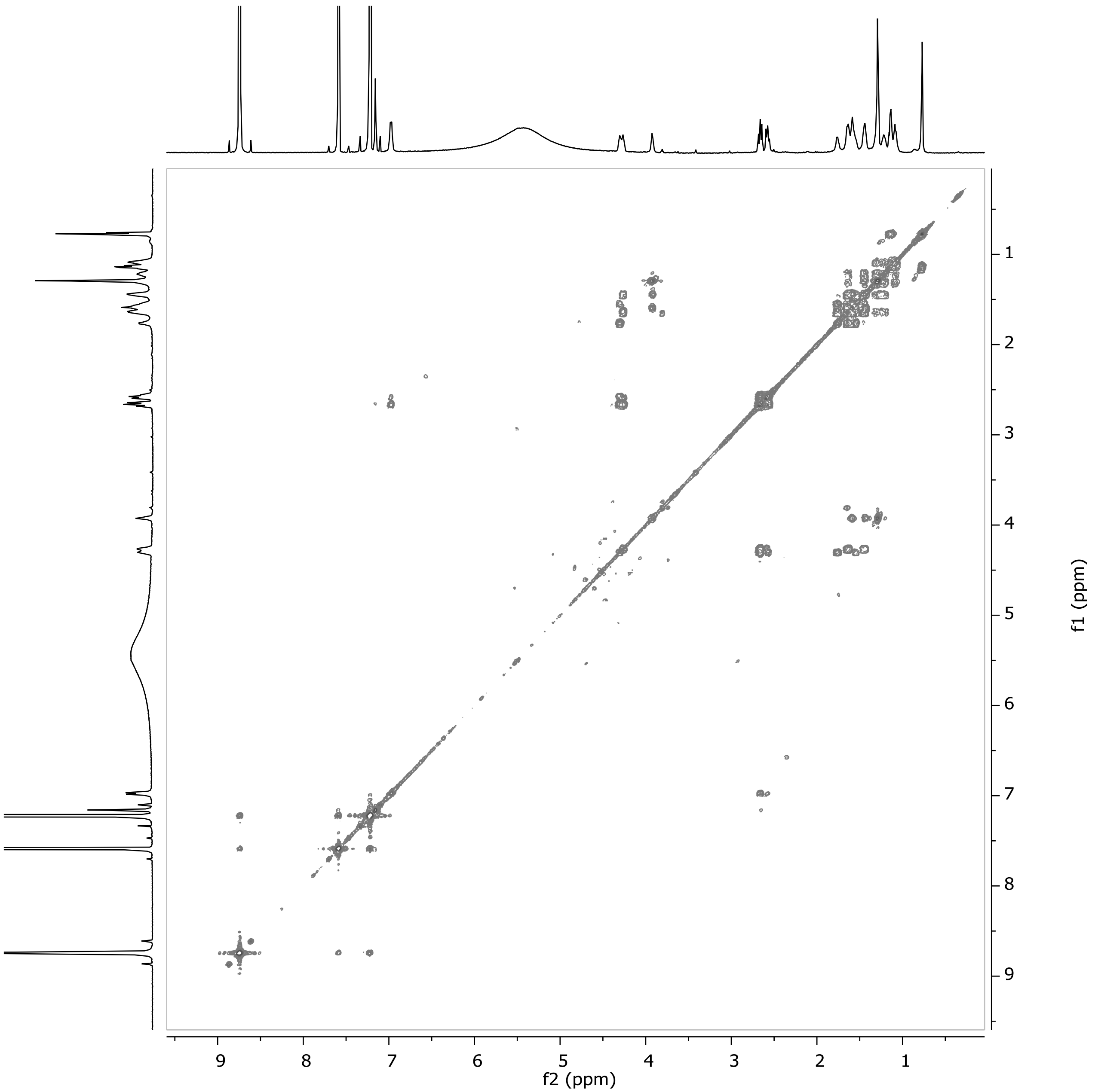

Figure S93. ^{1}H-^{1}H COSY spectrum of **4** in pyridine-d_5 at 700 MHz.

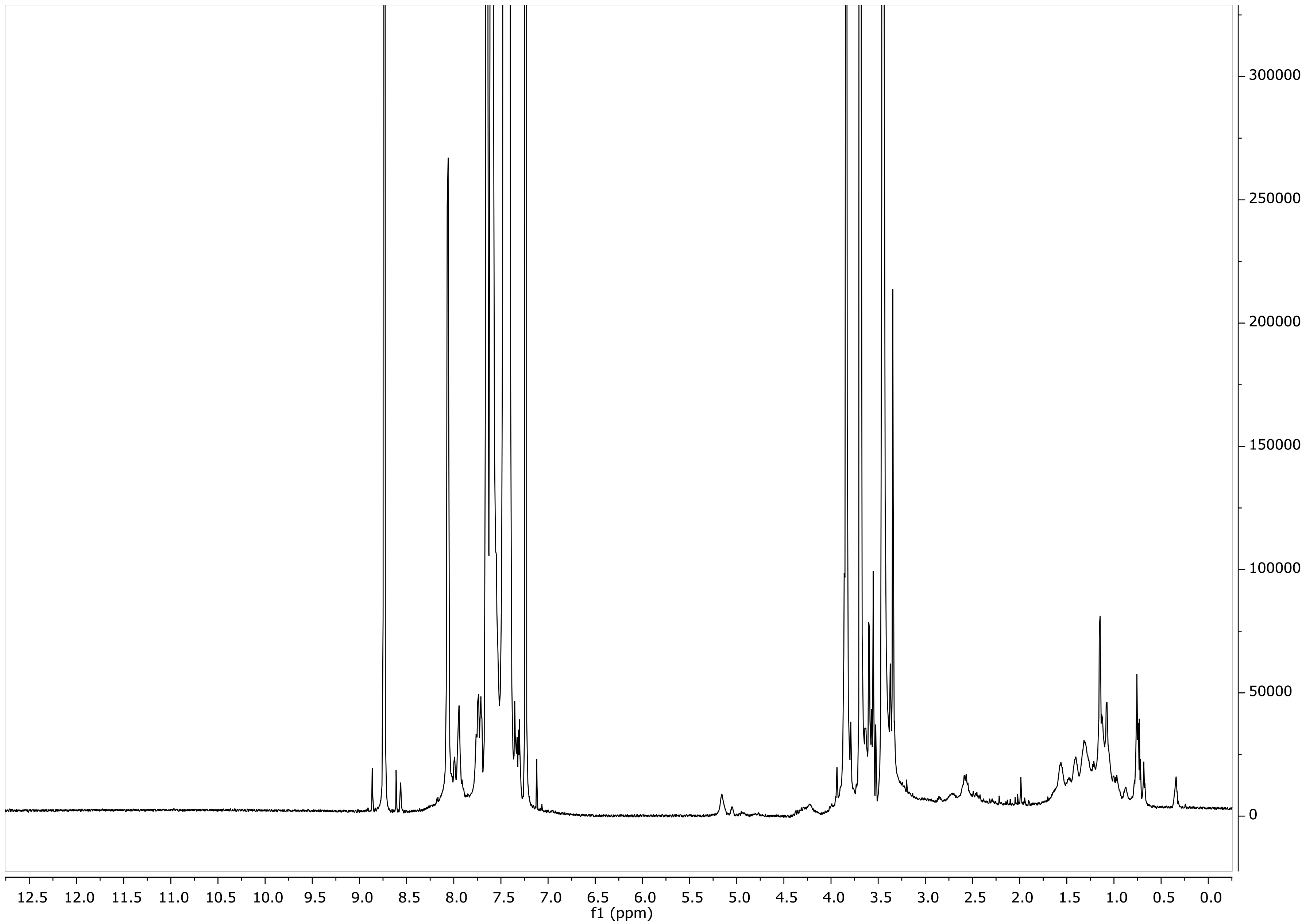

Figure S94. ^{1}H NMR spectrum of 14-*O*-(*R*)-MTPA ester of **4** in pyridine-d_5 at 700 MHz.

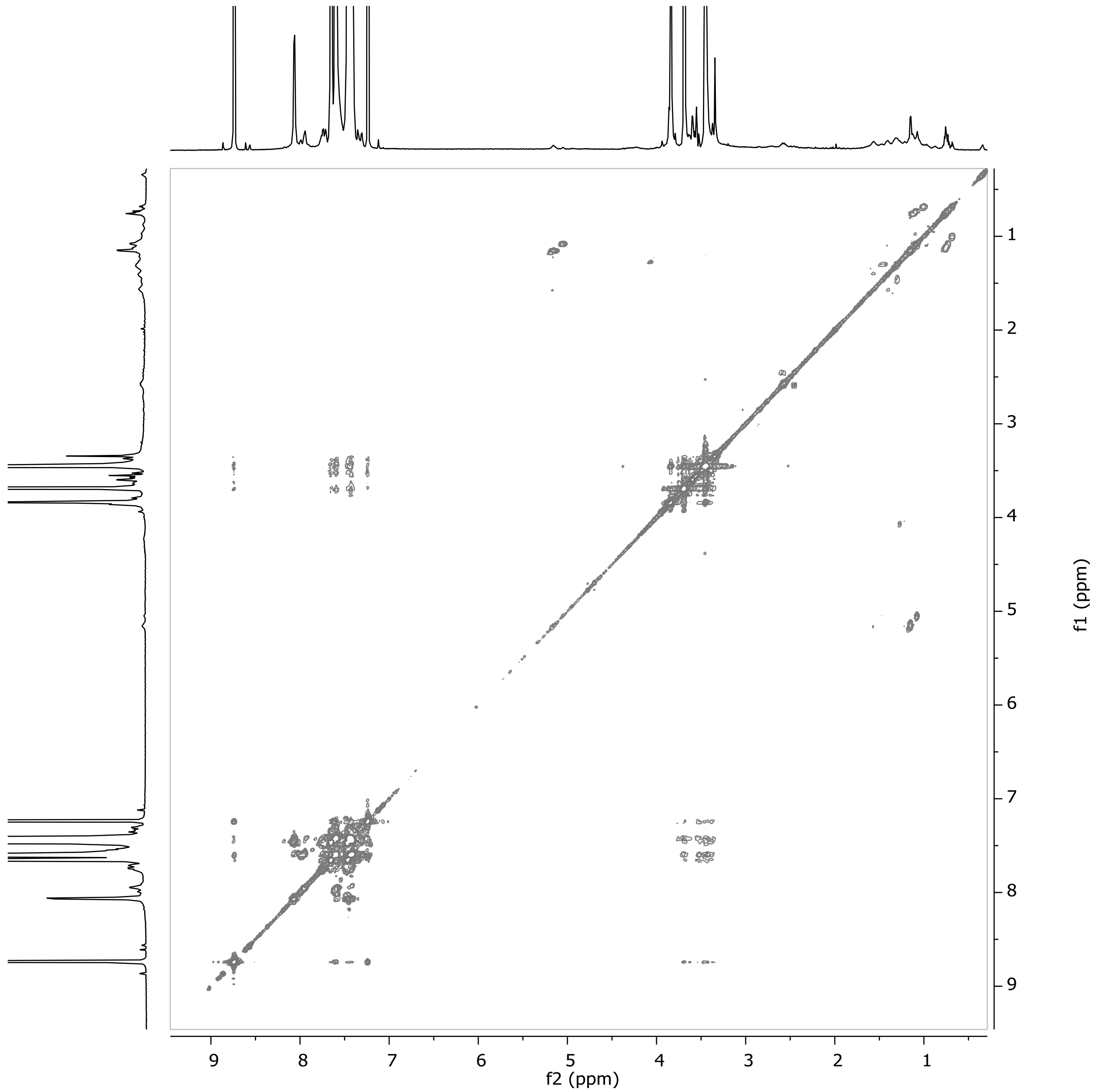

Figure S95. ^{1}H-^{1}H COSY spectrum of 14-*O*-(*R*)-MTPA ester of **4** in pyridine-d_5 at 700 MHz.

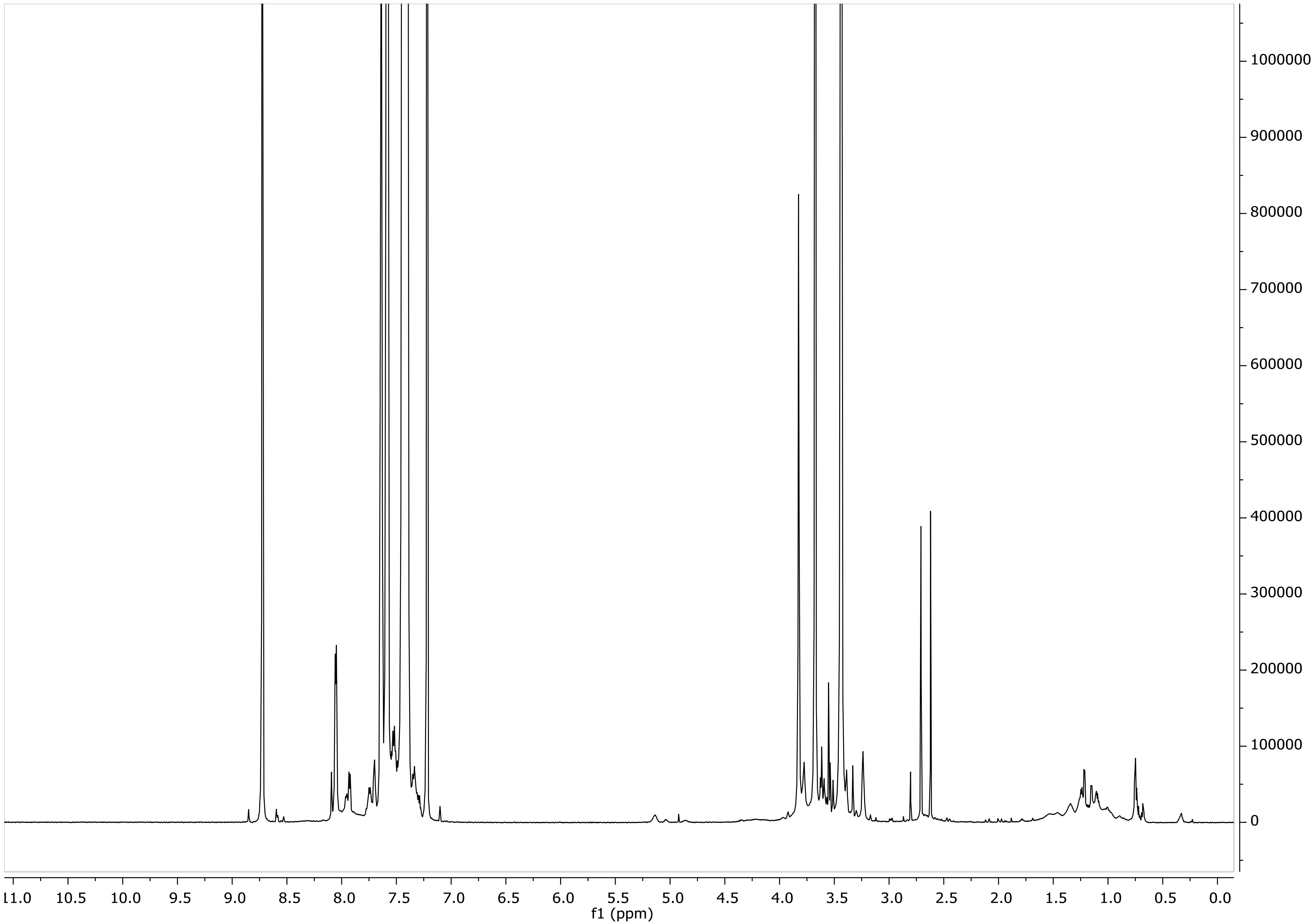

Figure S96. ^{1}H NMR spectrum of 14-*O*-(*S*)-MTPA ester of **4** in pyridine-d_5 at 700 MHz.

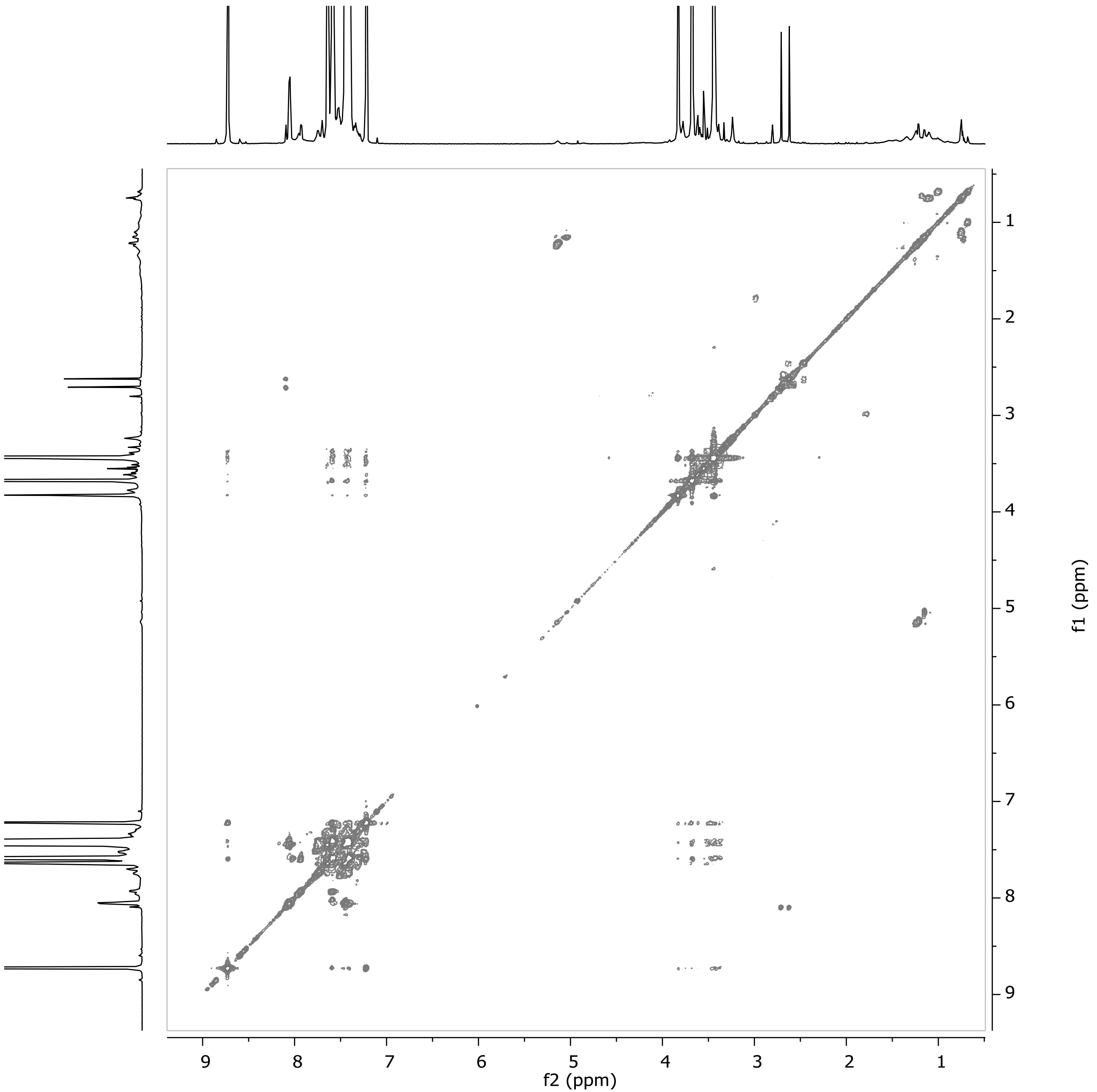

Figure S97. ^{1}H-^{1}H COSY spectrum of 14-*O*-(*S*)-MTPA ester of **4** in pyridine-d_5 at 700 MHz.

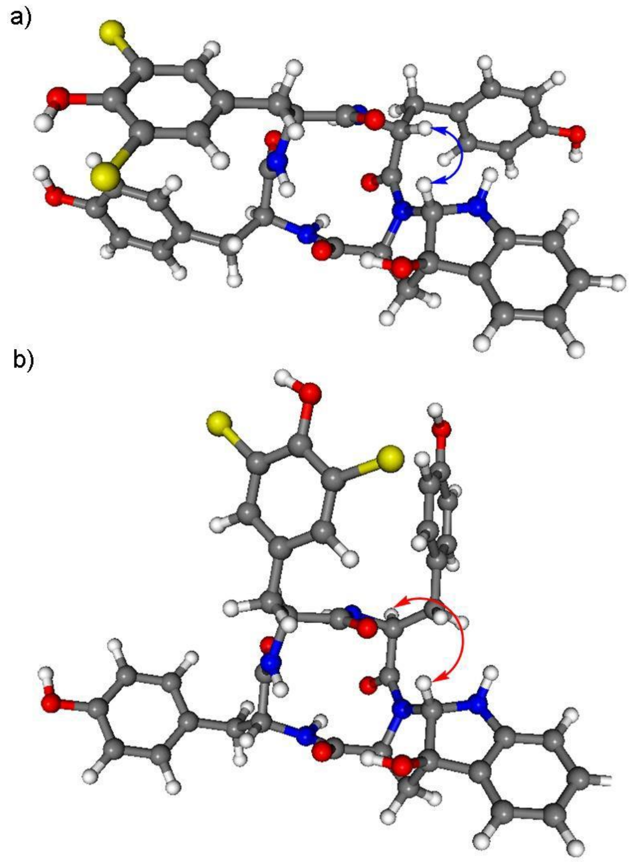

Figure S98. Lowest-energy MMFF conformers of a) (L-Tyr^1,D-Tyr^2)-**1** and b) (D-Tyr^1,L-Tyr^2)-**1** with the characteristic H-8a–Tyr2-H-α protons [d = 2.20 Å for a) and 4.12 Å for b)].

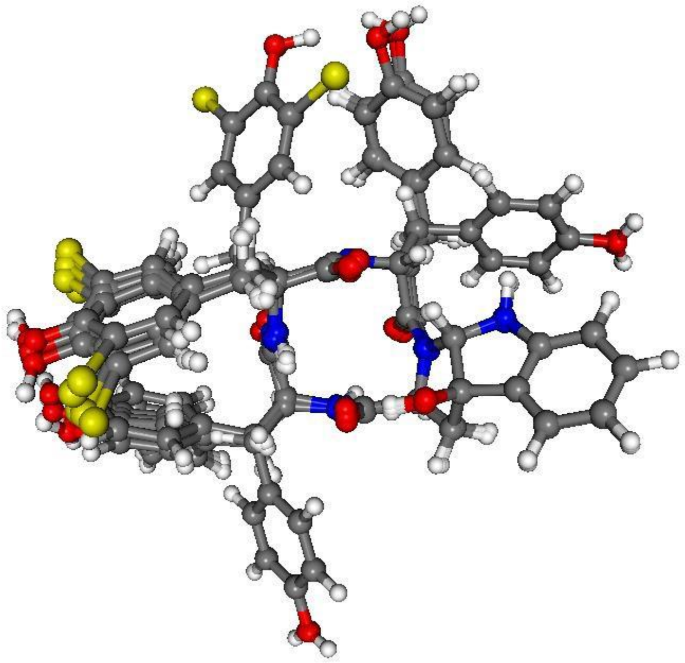

Figure S99. Thirteen low-energy overlapped solution conformers of (L-Tyr^1,D-Tyr^2)-**1** (≥ 1% Boltzmann population; level of optimization: ωB97X/TZVP PCM/MeCN).

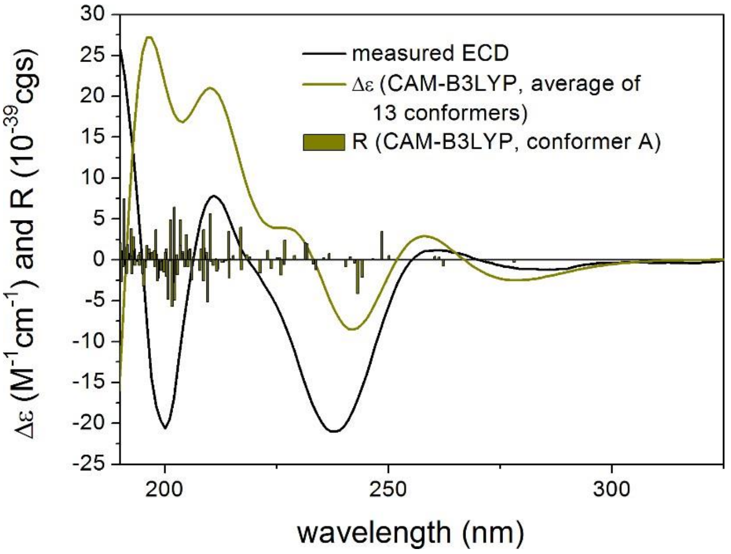

Figure S100. Experimental ECD spectrum of **1** (black) compared with the CAM-B3LYP sTDA spectrum of (L-Tyr^1,D-Tyr^2)-**1** (dark yellow). Level of DFT optimization: ωB97X/TZVP PCM/MeCN.

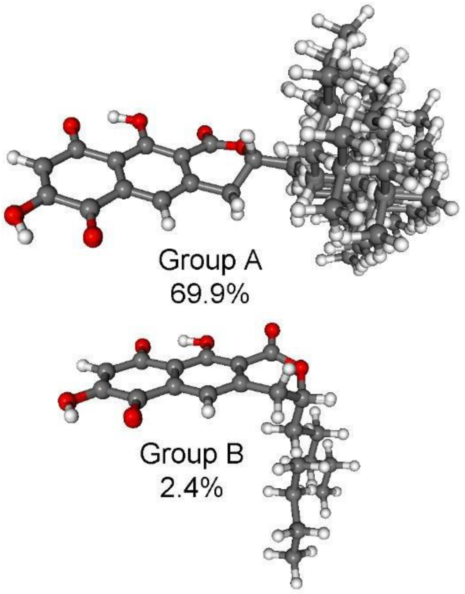

Figure S101. Thirty low-energy overlapped solution conformers of (*R*)-**3** (≥ 1% Boltzmann population) belonging to two groups based on the conformation of the hetero-ring [28 conformers with a sum Boltzmann population of 69.9% for group A and two conformers with 2.4% for group B; level of optimization: ωB97X/TZVP PCM/MeCN].

Table S14. Cartesian coordinates and energies of the low-energy conformers calculated at the ωB97X/TZVP PCM/MeCN level.

(L-Tyr[1],D-Tyr[2])-**1**, Conf A

N	2.701221	0.079288	0.773458
C	2.343830	0.817502	1.995333
C	3.075516	2.146288	1.841893
C	3.250970	2.363697	0.323712
C	3.361166	0.912902	-0.227688
C	4.589933	2.943442	-0.041835
C	5.443446	1.905264	-0.408663
N	4.795257	0.667674	-0.328838
C	5.032254	4.249547	-0.016229
C	6.351745	4.518546	-0.375957
C	7.198697	3.478287	-0.743667
C	6.759978	2.157513	-0.763763
O	2.195192	3.097895	-0.245430
H	2.851383	0.831905	-1.190023
C	2.452024	-1.245788	0.677836
O	2.114334	-1.912305	1.650503
N	0.149193	-0.004480	2.642997
C	0.812377	0.943389	1.962427
C	2.396083	-1.877075	-0.722362
O	0.262602	1.804496	1.272211
C	-1.260059	-0.363628	2.452844
C	-1.385525	-1.100026	1.107964
C	-2.226929	0.799896	2.681172
C	3.130269	-3.212814	-0.772081
C	4.614533	-2.985406	-0.617805
C	5.387321	-2.601054	-1.713861
C	6.731187	-2.294937	-1.576989
C	7.330888	-2.374511	-0.324209
C	6.581993	-2.765982	0.778212
C	5.234949	-3.064892	0.624323
O	8.654441	-2.061347	-0.239822
C	-3.673307	0.449735	2.410571
C	-4.227780	-0.781112	2.750960
C	-5.555084	-1.077305	2.469909
C	-6.351908	-0.126227	1.851596
C	-5.832258	1.120553	1.534764
C	-4.502006	1.392057	1.803984
O	-7.654747	-0.377441	1.491741
O	-1.439983	-2.323653	1.077617
N	-1.351301	-0.342971	-0.009002
C	-1.203757	-0.943078	-1.330649
C	0.303404	-0.978635	-1.622881
C	-1.973007	-0.175249	-2.395892
C	-3.449555	-0.054576	-2.102051
C	-4.206228	-1.152987	-1.708304
C	-5.554019	-1.013398	-1.437343
C	-6.203650	0.217854	-1.530391
C	-5.436666	1.299121	-1.954749
C	-4.086450	1.172746	-2.229239
Cl	-6.476652	-2.413194	-0.949243
O	-7.514004	0.392754	-1.248454
Cl	-6.189756	2.865867	-2.078707
N	0.969728	-1.990862	-1.007514
O	0.857659	-0.112363	-2.279752
H	2.665792	0.268908	2.880038
H	2.546033	2.980511	2.300874
H	4.054641	2.059009	2.312474
H	5.090545	-0.043377	-0.984954
H	4.358901	5.051817	0.265953
H	6.717798	5.538292	-0.373128
H	8.222722	3.697783	-1.024956
H	7.425308	1.351075	-1.052722
H	1.373601	2.798473	0.183612
H	0.727314	-0.741345	3.022927
H	2.808599	-1.215986	-1.481033
H	-1.455929	-1.135232	3.195605
H	-1.945540	1.649741	2.061525
H	-2.099316	1.118393	3.719995
H	2.910183	-3.683650	-1.732180
H	2.754553	-3.865082	0.018868
H	4.929957	-2.539305	-2.696932
H	7.327497	-2.000147	-2.432620
H	7.048336	-2.834785	1.755807
H	4.655570	-3.359820	1.493216
H	8.956103	-2.149196	0.669213
H	-3.625560	-1.542620	3.234620
H	-5.961415	-2.051478	2.721610
H	-6.465091	1.857321	1.052342
H	-4.096868	2.360501	1.528432
H	-7.919630	-1.251464	1.796859
H	-1.166923	0.648202	0.089474
H	-1.573187	-1.965542	-1.251183
H	-1.820293	-0.686395	-3.350322
H	-1.537428	0.819410	-2.506616
H	-3.757944	-2.134639	-1.609903
H	-3.527451	2.050079	-2.532750
H	-7.754210	-0.114127	-0.453165
H	0.432001	-2.585452	-0.387778

ωB97X Energy = -3265.42309446 a.u.

(L-Tyr[1],D-Tyr[2])-**1**, Conf B

N	2.642420	-0.040414	0.762543
C	2.249777	0.516357	2.067871
C	2.984903	1.849964	2.128929
C	3.199444	2.283363	0.663435
C	3.333246	0.926986	-0.086395
C	4.544360	2.911374	0.421158
C	5.414579	1.936058	-0.060877
N	4.771488	0.697416	-0.172879
C	4.977387	4.202094	0.641202

C	6.305076	4.521443	0.361744
C	7.168757	3.544419	-0.121983
C	6.739011	2.238369	-0.338820
O	2.153827	3.088838	0.177844
H	2.857154	0.985089	-1.067201
C	2.403790	-1.338636	0.470317
O	2.040676	-2.139065	1.326107
N	0.045345	-0.407772	2.483486
C	0.719450	0.650142	2.012522
C	2.391692	-1.760827	-1.007521
O	0.180019	1.622947	1.479154
C	-1.358436	-0.724023	2.204102
C	-1.455948	-1.219902	0.751305
C	-2.354293	0.375154	2.589465
C	3.105772	-3.090639	-1.223160
C	4.586780	-2.914022	-0.989940
C	5.399839	-2.384286	-1.992324
C	6.741341	-2.121694	-1.769548
C	7.297867	-2.393533	-0.523788
C	6.508627	-2.932250	0.484261
C	5.164443	-3.185006	0.245725
O	8.620879	-2.115598	-0.351778
C	-3.767592	-0.156423	2.584118
C	-4.614338	0.039239	1.499073
C	-5.904778	-0.475635	1.489695
C	-6.361488	-1.207827	2.577232
C	-5.529678	-1.415373	3.672363
C	-4.248543	-0.892936	3.666746
O	-7.615966	-1.739595	2.627986
O	-1.554450	-2.415093	0.503746
N	-1.345939	-0.279044	-0.212741
C	-1.156047	-0.635050	-1.614289
C	0.361471	-0.688146	-1.850538
C	-1.827520	0.363047	-2.549988
C	-3.303444	0.526650	-2.288078
C	-3.821697	1.765724	-1.939962
C	-5.176349	1.915051	-1.699648
C	-6.061848	0.845522	-1.791813
C	-5.524961	-0.394161	-2.138788
C	-4.176096	-0.553375	-2.387400
Cl	-5.817270	3.475882	-1.249413
O	-7.383756	0.944592	-1.545179
Cl	-6.592577	-1.765758	-2.245901
N	0.975696	-1.796247	-1.359145
O	0.967725	0.240873	-2.359237
H	2.546354	-0.155392	2.873149
H	2.442459	2.610174	2.689989
H	3.951398	1.692559	2.607223
H	5.089192	0.088068	-0.915162
H	4.290850	4.955598	1.011974
H	6.664542	5.531631	0.517296
H	8.199051	3.803115	-0.339869
H	7.417489	1.481971	-0.718169
H	1.324503	2.743824	0.554397
H	0.616276	-1.202679	2.737828
H	2.845852	-1.007076	-1.646491
H	-1.579045	-1.603095	2.808767
H	-2.265307	1.234972	1.926416
H	-2.085859	0.717719	3.591587
H	2.912807	-3.415607	-2.247391
H	2.693175	-3.841220	-0.546025
H	4.976171	-2.171970	-2.969540
H	7.368806	-1.713138	-2.553091
H	6.941242	-3.151567	1.455103
H	4.553706	-3.596039	1.042922
H	8.890559	-2.340340	0.543710
H	-4.267296	0.610575	0.642766
H	-6.559784	-0.305858	0.639949
H	-5.898832	-1.982573	4.518783
H	-3.609179	-1.055700	4.529354
H	-8.088532	-1.541866	1.813854
H	-1.124477	0.668564	0.071031
H	-1.578698	-1.631934	-1.744866
H	-1.662529	0.021889	-3.574887
H	-1.326331	1.328360	-2.461402
H	-3.172609	2.628874	-1.851964
H	-3.811027	-1.536676	-2.661384
H	-7.605423	1.856129	-1.315366
H	0.398721	-2.462510	-0.859368

ωB97X Energy = -3265.42185977 a.u.

(L-Tyr[1],D-Tyr[2])-**1**, Conf C

N	2.640596	-0.037052	0.767826
C	2.246857	0.528837	2.068898
C	2.983034	1.862274	2.121901
C	3.200537	2.285233	0.653783
C	3.334882	0.923456	-0.085847
C	4.546461	2.910468	0.409609
C	5.417200	1.930400	-0.061966
N	4.773093	0.691820	-0.165832
C	4.980083	4.202484	0.620415
C	6.308949	4.518315	0.342369
C	7.173259	3.536485	-0.130300
C	6.742807	2.229165	-0.337983
O	2.156272	3.087755	0.160560
H	2.861851	0.975232	-1.068520
C	2.401640	-1.337044	0.483693
O	2.036964	-2.131905	1.343908
N	0.041204	-0.390280	2.488634
C	0.716751	0.663379	2.010418
C	2.391220	-1.768297	-0.991521
O	0.178812	1.632344	1.468554
C	-1.362296	-0.708106	2.209719
C	-1.458461	-1.214221	0.760354
C	-2.358409	0.393968	2.586086
C	3.106678	-3.098588	-1.199239
C	4.587790	-2.919065	-0.968353
C	5.399242	-2.400893	-1.974486
C	6.743610	-2.136526	-1.754250
C	7.299426	-2.397440	-0.506404
C	6.508594	-2.924519	0.507322

C	5.166719	-3.177993	0.272219
O	8.611484	-2.159349	-0.224944
C	-3.771692	-0.137696	2.582982
C	-4.617255	0.049937	1.495608
C	-5.907534	-0.465404	1.488427
C	-6.365276	-1.189912	2.580667
C	-5.534651	-1.389355	3.678202
C	-4.253664	-0.866602	3.670300
O	-7.619643	-1.721720	2.633822
O	-1.557872	-2.411019	0.521160
N	-1.346633	-0.280226	-0.210180
C	-1.155684	-0.646338	-1.608971
C	0.362001	-0.701486	-1.843543
C	-1.826132	0.344898	-2.552699
C	-3.302131	0.511253	-2.292979
C	-3.819826	1.752851	-1.953097
C	-5.174568	1.904595	-1.714792
C	-6.060720	0.835106	-1.800955
C	-5.524398	-0.407092	-2.139702
C	-4.175437	-0.568786	-2.386182
Cl	-5.814771	3.468640	-1.274805
O	-7.382762	0.936615	-1.556043
Cl	-6.592881	-1.778586	-2.239170
N	0.975530	-1.806404	-1.344146
O	0.969007	0.223861	-2.358051
H	2.541879	-0.137637	2.879116
H	2.440202	2.626847	2.676615
H	3.948527	1.707418	2.603018
H	5.092911	0.074333	-0.900404
H	4.293228	4.959598	0.983162
H	6.668927	5.529375	0.490885
H	8.204616	3.792331	-0.346520
H	7.422163	1.468461	-0.706979
H	1.326066	2.745915	0.538096
H	0.611080	-1.183072	2.751512
H	2.845318	-1.018136	-1.634659
H	-1.583843	-1.582844	2.820280
H	-2.268604	1.248957	1.916909
H	-2.091074	0.743685	3.586028
H	2.913275	-3.430125	-2.221258
H	2.695537	-3.845524	-0.517181
H	4.976771	-2.197212	-2.953963
H	7.360288	-1.735507	-2.552282
H	6.953968	-3.129199	1.473803
H	4.554643	-3.580784	1.072676
H	9.057228	-1.810931	-1.002761
H	-4.269418	0.615219	0.635622
H	-6.561574	-0.301955	0.636693
H	-5.904592	-1.950613	4.528233
H	-3.615210	-1.023045	4.534750
H	-8.091430	-1.529817	1.817852
H	-1.124387	0.669090	0.067285
H	-1.578450	-1.644047	-1.732668
H	-1.660747	-0.004344	-3.574820
H	-1.324451	1.310574	-2.471387
H	-3.170216	2.616089	-1.869986
H	-3.810786	-1.554033	-2.653634
H	-7.603968	1.849676	-1.331900
H	0.398088	-2.469036	-0.840106

ωB97X Energy = -3265.42175381 a.u.

(L-Tyr[1],D-Tyr[2])-**1**, Conf D

N	2.640817	-0.034759	0.762387
C	2.244968	0.533723	2.061657
C	2.981035	1.867212	2.113156
C	3.199877	2.287766	0.644569
C	3.334510	0.924805	-0.093146
C	4.546016	2.912537	0.400656
C	5.416699	1.932353	-0.070575
N	4.772765	0.693474	-0.173753
C	4.979625	4.204670	0.610998
C	6.308425	4.520411	0.332692
C	7.172574	3.538512	-0.140214
C	6.742222	2.231059	-0.347215
O	2.156365	3.089988	0.149275
H	2.860958	0.974838	-1.075657
C	2.402285	-1.335430	0.481014
O	2.036778	-2.128184	1.342897
N	0.038412	-0.383985	2.480410
C	0.714976	0.668330	2.000702
C	2.393584	-1.770592	-0.993068
O	0.177921	1.636213	1.456074
C	-1.365249	-0.701060	2.201025
C	-1.459302	-1.212283	0.753343
C	-2.359744	0.404442	2.572064
C	3.108792	-3.101867	-1.195585
C	4.589283	-2.922283	-0.961396
C	5.403866	-2.401143	-1.967053
C	6.744785	-2.135688	-1.744149
C	7.299186	-2.395932	-0.494960
C	6.508416	-2.926023	0.516459
C	5.164826	-3.181741	0.277717
O	8.621702	-2.115542	-0.323078
C	-3.773882	-0.125085	2.580522
C	-4.257895	-0.822536	3.683978
C	-5.543528	-1.340326	3.704852
C	-6.372737	-1.165153	2.602771
C	-5.909598	-0.473145	1.491024
C	-4.619879	0.036679	1.485970
O	-7.648461	-1.645013	2.562071
O	-1.558607	-2.409819	0.517893
N	-1.345352	-0.281285	-0.219737
C	-1.152890	-0.650875	-1.617351
C	0.365055	-0.706009	-1.850079
C	-1.823219	0.338028	-2.563698
C	-3.299323	0.503577	-2.304155
C	-3.817288	1.743093	-1.957282
C	-5.172143	1.893033	-1.718285
C	-6.058362	0.824101	-1.811166
C	-5.521568	-0.416123	-2.156679
C	-4.172501	-0.576022	-2.403711

Cl	-5.812617	3.454547	-1.269408
O	-7.380612	0.924576	-1.568931
Cl	-6.589629	-1.787071	-2.265636
N	0.978376	-1.809618	-1.347462
O	0.972372	0.218352	-2.365939
H	2.538639	-0.131177	2.873649
H	2.437722	2.632708	2.666124
H	3.946100	1.713142	2.595410
H	5.092218	0.076830	-0.909223
H	4.292746	4.961929	0.973403
H	6.668396	5.531549	0.480726
H	8.203745	3.794404	-0.357261
H	7.421075	1.470807	-0.718112
H	1.325617	2.748463	0.525912
H	0.607457	-1.176649	2.745432
H	2.848738	-1.022204	-1.637587
H	-1.588905	-1.572943	2.814871
H	-2.272184	1.253896	1.895562
H	-2.087963	0.762496	3.567795
H	2.917782	-3.436122	-2.217191
H	2.695521	-3.846625	-0.512455
H	4.981881	-2.197862	-2.946913
H	7.373446	-1.733801	-2.530178
H	6.939316	-3.136271	1.490065
H	4.552815	-3.585866	1.077468
H	8.890038	-2.332285	0.574780
H	-3.621695	-0.959632	4.553263
H	-5.903620	-1.875525	4.577527
H	-6.571195	-0.338141	0.642100
H	-4.268328	0.577950	0.612117
H	-7.853727	-2.103227	3.382396
H	-1.123638	0.668836	0.055360
H	-1.575407	-1.648952	-1.739066
H	-1.657337	-0.013342	-3.584985
H	-1.322074	1.304133	-2.484121
H	-3.167899	2.606005	-1.869035
H	-3.807445	-1.559579	-2.676846
H	-7.601283	1.835065	-1.334259
H	0.400524	-2.471369	-0.842760

ωB97X Energy = -3265.42168256 a.u.

(L-Tyr[1],D-Tyr[2])-**1**, Conf E

N	2.689851	0.240100	0.730436
C	2.330900	1.216273	1.771713
C	3.098351	2.470804	1.372045
C	3.298541	2.377194	-0.155412
C	3.382902	0.843556	-0.405235
C	4.654846	2.845571	-0.606245
C	5.489401	1.738634	-0.743776
N	4.812528	0.554042	-0.430143
C	5.126164	4.121574	-0.834088
C	6.455826	4.287554	-1.217745
C	7.283778	3.178506	-1.356049
C	6.815673	1.889231	-1.119590
O	2.266501	3.004131	-0.876549
H	2.884143	0.582327	-1.340673
C	2.415814	-1.072564	0.900215
O	2.053390	-1.523949	1.981818
N	0.111838	0.557558	2.500605
C	0.803587	1.364531	1.684066
C	2.359812	-1.969686	-0.345884
O	0.279716	2.105214	0.848434
C	-1.308310	0.219374	2.368889
C	-1.459216	-0.751806	1.185025
C	-2.248261	1.428372	2.365986
C	3.045793	-3.311056	-0.112216
C	4.534660	-3.102067	0.022650
C	5.136203	-2.953871	1.267636
C	6.489702	-2.667913	1.385142
C	7.264485	-2.520774	0.241696
C	6.683655	-2.671747	-1.013519
C	5.332865	-2.961235	-1.112922
O	8.595839	-2.233394	0.287995
C	-3.692105	1.004322	2.488989
C	-4.561297	1.089367	1.408476
C	-5.889322	0.695758	1.514600
C	-6.363658	0.200623	2.721612
C	-5.509575	0.105509	3.815806
C	-4.190890	0.505437	3.692757
O	-7.655642	-0.199839	2.894135
O	-1.580805	-1.954204	1.381249
N	-1.365813	-0.218851	-0.054382
C	-1.192693	-1.059053	-1.235008
C	0.320298	-1.196733	-1.455576
C	-1.885242	-0.473449	-2.459523
C	-3.378822	-0.361980	-2.287505
C	-4.002042	0.878120	-2.266611
C	-5.371228	0.978549	-2.090998
C	-6.167756	-0.150111	-1.927542
C	-5.526646	-1.388468	-1.956681
C	-4.162461	-1.500893	-2.133858
Cl	-6.121580	2.549575	-2.034415
O	-7.493909	-0.001778	-1.734768
Cl	-6.506092	-2.820696	-1.753815
N	0.935092	-2.089619	-0.636736
O	0.923091	-0.491315	-2.248604
H	2.623642	0.848963	2.754937
H	2.583977	3.391073	1.646821
H	4.068661	2.456884	1.868177
H	5.098556	-0.276731	-0.931335
H	4.467490	4.976985	-0.729025
H	6.844699	5.279968	-1.412071
H	8.315988	3.317235	-1.658057
H	7.466426	1.028643	-1.230641
H	1.434130	2.824657	-0.403553
H	0.665835	-0.116165	3.011900
H	2.808572	-1.487287	-1.210998
H	-1.540226	-0.385983	3.244477
H	-2.106481	2.030920	1.469889
H	-1.964435	2.054870	3.215544
H	2.822440	-3.957792	-0.962907

H	2.636893	-3.778871	0.785614
H	4.536902	-3.056643	2.166427
H	6.940659	-2.556030	2.365940
H	7.299699	-2.566858	-1.898999
H	4.890049	-3.080646	-2.097452
H	8.883284	-2.150453	1.202157
H	-4.200934	1.477816	0.461142
H	-6.557955	0.781472	0.662556
H	-5.892694	-0.275596	4.755161
H	-3.538119	0.436638	4.557921
H	-8.147118	-0.083506	2.075512
H	-1.119437	0.761194	-0.132007
H	-1.614201	-2.034518	-0.989455
H	-1.652957	-1.115413	-3.312456
H	-1.457832	0.505782	-2.681875
H	-3.420449	1.785258	-2.383031
H	-3.716134	-2.488665	-2.155091
H	-7.909058	-0.869236	-1.645569
H	0.364116	-2.545467	0.064851

ωB97X Energy = -3265.42164411 a.u.

(L-Tyr^1,D-Tyr^2)-**1**, Conf F

N	2.638956	-0.031755	0.768078
C	2.242245	0.544370	2.063713
C	2.978942	1.877821	2.108262
C	3.200080	2.289475	0.637489
C	3.335405	0.921998	-0.091471
C	4.546935	2.912143	0.391875
C	5.418244	1.928099	-0.070272
N	4.773670	0.689204	-0.166517
C	4.980822	4.205419	0.594218
C	6.310575	4.518392	0.317178
C	7.175464	3.532574	-0.146043
C	6.744731	2.224008	-0.345136
O	2.157479	3.088853	0.135790
H	2.864449	0.966508	-1.075523
C	2.400380	-1.333921	0.493649
O	2.033750	-2.121955	1.359303
N	0.034928	-0.369891	2.485481
C	0.712411	0.679251	2.000223
C	2.393091	-1.776766	-0.978133
O	0.176381	1.643986	1.449002
C	-1.368401	-0.688543	2.206282
C	-1.461093	-1.207854	0.761393
C	-2.363447	0.418844	2.570103
C	3.109385	-3.108404	-1.174013
C	4.590094	-2.926422	-0.942538
C	5.402622	-2.415441	-1.951488
C	6.746561	-2.148823	-1.731455
C	7.300928	-2.400274	-0.481020
C	6.509007	-2.920075	0.535604
C	5.167512	-3.175796	0.300672
O	8.612553	-2.159515	-0.199783
C	-3.777488	-0.110958	2.579298
C	-4.262176	-0.804643	3.684842
C	-5.547603	-1.322887	3.706481
C	-6.375948	-1.151967	2.603075
C	-5.912128	-0.463780	1.489255
C	-4.622595	0.046526	1.483451
O	-7.651441	-1.632502	2.562982
O	-1.560640	-2.406654	0.532580
N	-1.345912	-0.282362	-0.216797
C	-1.152394	-0.659971	-1.612108
C	0.365708	-0.717161	-1.843256
C	-1.821423	0.323682	-2.564831
C	-3.297671	0.491512	-2.307623
C	-3.815268	1.733127	-1.967772
C	-5.170295	1.885120	-1.731028
C	-6.057044	0.816245	-1.819329
C	-5.520622	-0.426078	-2.157786
C	-4.171383	-0.588075	-2.402475
Cl	-5.810308	3.449312	-1.290901
O	-7.379505	0.918750	-1.579066
Cl	-6.589408	-1.796896	-2.260861
N	0.978155	-1.817948	-1.333413
O	0.973900	0.203729	-2.364312
H	2.534822	-0.115942	2.879831
H	2.435178	2.646873	2.655823
H	3.943218	1.726176	2.592834
H	5.094946	0.065857	-0.895467
H	4.293545	4.965609	0.949670
H	6.670800	5.530306	0.459133
H	8.207524	3.786201	-0.361522
H	7.424540	1.460148	-0.706714
H	1.326132	2.750045	0.513587
H	0.603351	-1.160770	2.756959
H	2.848264	-1.031478	-1.626137
H	-1.592650	-1.557038	2.824683
H	-2.275044	1.264722	1.889230
H	-2.093099	0.782146	3.564326
H	2.917547	-3.448345	-2.193565
H	2.697643	-3.849955	-0.486466
H	4.981350	-2.219221	-2.933002
H	7.364081	-1.753389	-2.531600
H	6.953209	-3.117365	1.504159
H	4.554525	-3.572770	1.103340
H	9.059221	-1.817349	-0.979863
H	-3.626638	-0.938421	4.555126
H	-5.908201	-1.855184	4.580726
H	-6.573049	-0.332146	0.639283
H	-4.270484	0.584718	0.607932
H	-7.857304	-2.087636	3.384872
H	-1.123931	0.669165	0.053243
H	-1.575242	-1.658544	-1.728480
H	-1.654845	-0.033869	-3.583865
H	-1.319816	1.289955	-2.490581
H	-3.165448	2.596088	-1.883280
H	-3.806603	-1.573265	-2.670016
H	-7.599878	1.830549	-1.349236
H	0.399667	-2.476314	-0.825006

ωB97X Energy = -3265.42158022 a.u.

(L-Tyr[1],D-Tyr[2])-**1**, Conf G

N	2.667808	0.071436	0.761618
C	2.296212	0.806196	1.981845
C	3.050208	2.124569	1.854690
C	3.256446	2.351398	0.342171
C	3.361604	0.903848	-0.217734
C	4.608467	2.918440	0.005992
C	5.458140	1.872542	-0.347843
N	4.794910	0.641376	-0.286844
C	5.064381	4.219444	0.047135
C	6.393925	4.475533	-0.283791
C	7.236923	3.427754	-0.638955
C	6.784336	2.111967	-0.674515
O	2.218195	3.100608	-0.239945
H	2.873934	0.836073	-1.192201
C	2.409516	-1.250839	0.651819
O	2.046649	-1.922210	1.612319
N	0.084472	-0.017128	2.544524
C	0.767361	0.953000	1.921134
C	2.373948	-1.869179	-0.754591
O	0.235862	1.847690	1.258972
C	-1.328155	-0.344498	2.330669
C	-1.456064	-1.042479	0.965760
C	-2.296006	0.818276	2.570737
C	3.076539	-3.221635	-0.798465
C	4.561398	-3.026883	-0.608043
C	5.366264	-2.645672	-1.681997
C	6.712060	-2.366533	-1.511226
C	7.281156	-2.470314	-0.245977
C	6.499893	-2.859084	0.834757
C	5.151366	-3.130882	0.647006
O	8.608060	-2.182894	-0.128290
C	-3.725180	0.333442	2.618781
C	-4.204055	-0.349636	3.733796
C	-5.506923	-0.818983	3.788877
C	-6.360455	-0.606770	2.711645
C	-5.904264	0.076286	1.591881
C	-4.596193	0.535170	1.551703
O	-7.653308	-1.040396	2.704555
O	-1.569413	-2.259721	0.896408
N	-1.354326	-0.253421	-0.127085
C	-1.177243	-0.813127	-1.462459
C	0.337288	-0.901388	-1.702939
C	-1.860935	0.033960	-2.528257
C	-3.347904	0.168325	-2.315708
C	-4.173629	-0.950822	-2.313994
C	-5.532624	-0.812553	-2.115440
C	-6.128564	0.433606	-1.921968
C	-5.289167	1.543089	-1.924869
C	-3.924238	1.415789	-2.116518
Cl	-6.563235	-2.222610	-2.095835
O	-7.450338	0.603545	-1.716520
Cl	-5.980439	3.118783	-1.657465
N	0.952917	-1.939929	-1.079145
O	0.940606	-0.048410	-2.333744
H	2.591776	0.245849	2.868507
H	2.524139	2.962286	2.311197
H	4.019047	2.018857	2.342408
H	5.095247	-0.068499	-0.942037
H	4.394139	5.027667	0.319486
H	6.770995	5.491141	-0.268288
H	8.268902	3.637261	-0.897883
H	7.446764	1.299694	-0.953532
H	1.387329	2.820927	0.184460
H	0.647296	-0.776079	2.904579
H	2.823565	-1.212435	-1.495626
H	-1.553594	-1.124812	3.056745
H	-2.180763	1.587481	1.808193
H	-2.020068	1.274199	3.524808
H	2.868814	-3.679311	-1.767633
H	2.668278	-3.871758	-0.022100
H	4.932798	-2.564653	-2.674491
H	7.333239	-2.074027	-2.349775
H	6.941950	-2.946986	1.822038
H	4.546445	-3.423158	1.499223
H	8.887168	-2.284212	0.786529
H	-3.550593	-0.513756	4.585491
H	-5.862416	-1.343985	4.669613
H	-6.584843	0.249303	0.765304
H	-4.250038	1.068260	0.671130
H	-7.849674	-1.503970	3.524070
H	-1.119796	0.722821	0.011687
H	-1.600014	-1.818301	-1.440258
H	-1.661138	-0.426621	-3.498854
H	-1.397120	1.021486	-2.552715
H	-3.766381	-1.943038	-2.470454
H	-3.309003	2.307894	-2.108575
H	-7.899951	-0.250244	-1.759079
H	0.380048	-2.530466	-0.488045

ωB97X Energy = -3265.42155548 a.u.

(L-Tyr[1],D-Tyr[2])-**1**, Conf H

N	2.686365	0.249051	0.739771
C	2.324497	1.259915	1.746231
C	3.096443	2.499326	1.308921
C	3.307295	2.353253	-0.213104
C	3.392040	0.812040	-0.408287
C	4.667857	2.804314	-0.669466
C	5.502715	1.692210	-0.756874
N	4.821206	0.521813	-0.406175
C	5.143166	4.070422	-0.939472
C	6.477219	4.220887	-1.314778
C	7.305463	3.106929	-1.402171
C	6.833183	1.827594	-1.123379
O	2.280759	2.954998	-0.962790
H	2.903116	0.518925	-1.339522
C	2.408420	-1.056811	0.950325
O	2.039116	-1.472677	2.043713

N	0.100285	0.633359	2.487620
C	0.798079	1.408108	1.645509
C	2.357923	-1.993580	-0.266548
O	0.280571	2.118765	0.780353
C	-1.320415	0.295167	2.362668
C	-1.470709	-0.719431	1.215680
C	-2.255306	1.506991	2.311038
C	3.056353	-3.321091	0.007409
C	4.544734	-3.091858	0.114554
C	5.159935	-2.874373	1.345567
C	6.506882	-2.560627	1.431778
C	7.266284	-2.454072	0.272963
C	6.675180	-2.678046	-0.965842
C	5.325916	-2.995374	-1.034275
O	8.583435	-2.131244	0.407958
C	-3.702221	1.095275	2.440299
C	-4.569454	1.167143	1.357277
C	-5.900950	0.787144	1.469162
C	-6.381015	0.319424	2.684736
C	-5.529126	0.238121	3.781759
C	-4.206919	0.624317	3.652900
O	-7.676708	-0.066379	2.863003
O	-1.601067	-1.912587	1.456986
N	-1.367414	-0.234741	-0.042699
C	-1.194120	-1.120354	-1.189522
C	0.319087	-1.265300	-1.403201
C	-1.885176	-0.583168	-2.436942
C	-3.377957	-0.454467	-2.269991
C	-4.170340	-1.579600	-2.067620
C	-5.533989	-1.449222	-1.897933
C	-6.165941	-0.206185	-1.924842
C	-5.360641	0.908133	-2.136536
C	-3.992077	0.789867	-2.304782
Cl	-6.524531	-2.863785	-1.634365
O	-7.491315	-0.039518	-1.741791
Cl	-6.098954	2.485792	-2.152160
N	0.933963	-2.129754	-0.554073
O	0.922598	-0.585946	-2.218155
H	2.611582	0.925105	2.742665
H	2.581173	3.428950	1.548240
H	4.063217	2.501646	1.812063
H	5.114507	-0.330954	-0.864495
H	4.484651	4.929835	-0.873069
H	6.869369	5.205104	-1.541512
H	8.341244	3.233720	-1.697140
H	7.484411	0.962824	-1.191811
H	1.445036	2.792893	-0.489402
H	0.649798	-0.021887	3.026847
H	2.802698	-1.535488	-1.146659
H	-1.557830	-0.276343	3.259237
H	-2.105705	2.076239	1.394711
H	-1.973066	2.162465	3.139050
H	2.828823	-4.000092	-0.816549
H	2.662715	-3.759361	0.926714
H	4.572022	-2.943674	2.255165
H	6.979741	-2.391194	2.391996
H	7.268163	-2.610750	-1.872434
H	4.875011	-3.168569	-2.006922
H	9.002276	-2.086991	-0.456795
H	-4.204949	1.534930	0.403256
H	-6.567878	0.862306	0.614832
H	-5.916686	-0.121334	4.727802
H	-3.556340	0.567062	4.520553
H	-8.166121	0.039139	2.041655
H	-1.115699	0.740346	-0.156708
H	-1.615529	-2.085431	-0.905956
H	-1.657378	-1.261996	-3.262178
H	-1.451950	0.383112	-2.700675
H	-3.731657	-2.570743	-2.044990
H	-3.403658	1.686679	-2.460281
H	-7.913068	-0.898955	-1.614350
H	0.363387	-2.561157	0.163020

ωB97X Energy = -3265.42151051 a.u.

(L-Tyr[1],D-Tyr[2])-**1**, Conf I

N	2.666955	0.072110	0.760952
C	2.294971	0.810398	1.978941
C	3.050125	2.127920	1.849030
C	3.258944	2.350088	0.336146
C	3.364578	0.900733	-0.218649
C	4.611954	2.915335	0.000659
C	5.462163	1.867293	-0.345779
N	4.797849	0.637154	-0.281599
C	5.068535	4.216188	0.037279
C	6.399349	4.469968	-0.290532
C	7.243010	3.420044	-0.637567
C	6.789635	2.104401	-0.668772
O	2.221874	3.097644	-0.250081
H	2.880121	0.830301	-1.194555
C	2.407203	-1.250117	0.653830
O	2.041622	-1.918881	1.615063
N	0.082119	-0.008909	2.542716
C	0.766350	0.958264	1.916326
C	2.373663	-1.871580	-0.751228
O	0.236252	1.850982	1.250343
C	-1.330571	-0.335919	2.328999
C	-1.458211	-1.038758	0.966521
C	-2.297557	0.828576	2.564247
C	3.077627	-3.223430	-0.792389
C	4.561715	-3.027688	-0.596333
C	5.370434	-2.650373	-1.665405
C	6.718022	-2.369796	-1.488187
C	7.279390	-2.470386	-0.219756
C	6.491341	-2.856533	0.857695
C	5.146526	-3.128385	0.664294
O	8.594316	-2.206601	0.022378
C	-3.726949	0.344790	2.615765
C	-4.206405	-0.328647	3.736377
C	-5.509459	-0.797034	3.795060
C	-6.362592	-0.593589	2.715830
C	-5.905745	0.079598	1.590368

C	-4.597483	0.537622	1.546643
O	-7.655663	-1.026618	2.712110
O	-1.573245	-2.256044	0.901479
N	-1.354589	-0.253738	-0.129125
C	-1.177414	-0.818379	-1.462375
C	0.337145	-0.906797	-1.702536
C	-1.861673	0.024239	-2.531346
C	-3.348507	0.159640	-2.318407
C	-3.924806	1.408164	-2.125814
C	-5.289627	1.536458	-1.933990
C	-6.128937	0.426946	-1.924358
C	-5.533010	-0.820253	-2.111029
C	-4.174144	-0.959541	-2.309768
Cl	-5.980880	3.113577	-1.675206
O	-7.450611	0.597899	-1.719113
Cl	-6.563507	-2.230216	-2.082505
N	0.952937	-1.944179	-1.076874
O	0.940386	-0.054822	-2.334772
H	2.589353	0.252199	2.867353
H	2.523848	2.967342	2.302159
H	4.018100	2.023055	2.338619
H	5.100508	-0.077201	-0.930742
H	4.397990	5.026052	0.303942
H	6.776985	5.485408	-0.278396
H	8.276133	3.627729	-0.893396
H	7.452954	1.290027	-0.939429
H	1.390255	2.819742	0.174045
H	0.643922	-0.766845	2.906407
H	2.823442	-1.216059	-1.493169
H	-1.557196	-1.113373	3.057770
H	-2.182374	1.594171	1.798059
H	-2.020707	1.288777	3.515987
H	2.873580	-3.681319	-1.762217
H	2.667255	-3.873949	-0.017438
H	4.943410	-2.571668	-2.660803
H	7.332380	-2.080322	-2.334970
H	6.941202	-2.937549	1.840243
H	4.536148	-3.419062	1.513216
H	9.037251	-1.961689	-0.795438
H	-3.553204	-0.485790	4.589581
H	-5.865427	-1.314425	4.680102
H	-6.586016	0.245824	0.762158
H	-4.250806	1.062978	0.661614
H	-7.852652	-1.482446	3.535812
H	-1.119090	0.722751	0.006285
H	-1.599702	-1.823653	-1.436299
H	-1.662568	-0.440528	-3.500105
H	-1.397778	1.011599	-2.560287
H	-3.309625	2.300333	-2.123251
H	-3.766926	-1.952609	-2.460771
H	-7.900251	-0.256085	-1.757382
H	0.380180	-2.533151	-0.484116

ωB97X Energy = -3265.42145324 a.u.

(L-Tyr^{1},D-Tyr^{2})-**1**, Conf J

N	3.010176	-1.073229	-1.115012
C	2.540686	-2.321673	-1.736020
C	3.268913	-3.406822	-0.954285
C	3.538795	-2.815286	0.444997
C	3.670441	-1.284950	0.175140
C	4.897331	-3.162837	0.986030
C	5.757914	-2.088862	0.782937
N	5.105856	-1.023927	0.142158
C	5.343303	-4.328546	1.575100
C	6.674470	-4.410860	1.976584
C	7.529459	-3.331745	1.776166
C	7.086514	-2.157678	1.175454
O	2.528237	-3.131231	1.372221
H	3.166681	-0.715726	0.958328
C	2.803884	0.112108	-1.733115
O	2.421710	0.175080	-2.896857
N	0.307515	-1.811367	-2.553552
C	1.016527	-2.328738	-1.537858
C	2.859310	1.392007	-0.886699
O	0.516635	-2.694435	-0.471696
C	-1.073565	-1.324186	-2.465402
C	-1.057682	-0.010145	-1.666448
C	-2.073415	-2.387528	-2.006875
C	3.492528	2.549948	-1.654104
C	3.564699	3.810698	-0.831828
C	2.647057	4.839871	-1.005624
C	2.693939	5.988908	-0.227164
C	3.672439	6.117526	0.749678
C	4.602489	5.100769	0.937388
C	4.541736	3.963165	0.151203
O	3.772935	7.218683	1.548962
C	-3.480290	-1.862939	-1.823700
C	-4.045159	-0.905022	-2.661136
C	-5.332915	-0.429866	-2.448963
C	-6.079567	-0.927259	-1.392279
C	-5.551883	-1.908214	-0.565037
C	-4.260042	-2.356035	-0.778758
O	-7.335437	-0.456245	-1.090362
O	-1.082718	1.066321	-2.252986
N	-0.927922	-0.107121	-0.327366
C	-0.642672	1.063472	0.495735
C	0.887685	1.182937	0.580775
C	-1.287029	0.962849	1.870913
C	-2.785411	0.780973	1.815513
C	-3.395883	-0.218989	2.560772
C	-4.766181	-0.403587	2.505532
C	-5.581228	0.386117	1.699575
C	-4.954424	1.405160	0.981439
C	-3.587315	1.600135	1.028002
Cl	-5.489763	-1.700802	3.417132
O	-6.912532	0.155307	1.657964
Cl	-5.933046	2.452404	-0.015882
N	1.464350	1.657694	-0.551122
O	1.520317	0.802112	1.552782
H	2.790771	-2.335255	-2.796289
H	2.705165	-4.336636	-0.886916

H	4.216246	-3.613900	-1.451918
H	5.389490	-0.102153	0.441931
H	4.662740	-5.158051	1.734563
H	7.044050	-5.312736	2.449815
H	8.563039	-3.402610	2.096343
H	7.758553	-1.320491	1.025226
H	1.678578	-3.059550	0.901684
H	0.857199	-1.404301	-3.297968
H	3.386124	1.239070	0.053475
H	-1.327519	-1.014443	-3.477852
H	-1.740114	-2.838755	-1.073876
H	-2.057261	-3.182498	-2.758299
H	2.923675	2.718448	-2.570813
H	4.495795	2.237746	-1.956337
H	1.878682	4.750640	-1.766942
H	1.970458	6.783225	-0.381326
H	5.366277	5.215043	1.697689
H	5.277264	3.178331	0.302589
H	3.076612	7.845678	1.332565
H	-3.481286	-0.498325	-3.493611
H	-5.745647	0.335055	-3.098688
H	-6.145349	-2.290018	0.258314
H	-3.845407	-3.103503	-0.109864
H	-7.618316	0.181762	-1.753705
H	-0.772020	-1.024235	0.073371
H	-1.030809	1.926487	-0.045809
H	-1.037975	1.872803	2.423097
H	-0.835273	0.135710	2.421543
H	-2.801752	-0.876315	3.184625
H	-3.159210	2.404596	0.441575
H	-7.235926	0.232676	0.743233
H	0.848451	1.866635	-1.328810

ωB97X Energy = -3265.42034705 a.u.

(L-Tyr[1],D-Tyr[2])-**1**, Conf K

N	3.011756	-1.070556	-1.114855
C	2.542106	-2.317320	-1.739268
C	3.268559	-3.404797	-0.959121
C	3.537662	-2.816384	0.441638
C	3.672184	-1.285893	0.174491
C	4.895085	-3.167151	0.983388
C	5.757942	-2.094743	0.781515
N	5.108213	-1.028182	0.141134
C	5.338393	-4.334232	1.571703
C	6.669223	-4.419583	1.973731
C	7.526496	-3.342069	1.774535
C	7.086218	-2.166594	1.174565
O	2.525513	-3.132347	1.367067
H	3.170092	-0.716872	0.958893
C	2.807116	0.116291	-1.730695
O	2.426080	0.181725	-2.894653
N	0.310135	-1.802821	-2.557579
C	1.017708	-2.324002	-1.542878
C	2.861855	1.394816	-0.881752
O	0.516364	-2.693073	-0.478537
C	-1.070638	-1.314811	-2.469155
C	-1.054375	-0.003817	-1.665227
C	-2.071796	-2.378975	-2.015536
C	3.492063	2.554756	-1.648699
C	3.553974	3.818226	-0.829728
C	4.542833	3.993024	0.133798
C	4.594834	5.137004	0.916388
C	3.641154	6.133084	0.741192
C	2.647086	5.979337	-0.216900
C	2.610493	4.829741	-0.989893
O	3.637392	7.282078	1.476951
C	-3.478401	-1.854021	-1.831136
C	-4.040651	-0.888984	-2.662130
C	-5.328233	-0.413921	-2.448536
C	-6.077358	-0.918568	-1.397073
C	-5.552280	-1.906534	-0.576519
C	-4.260618	-2.354233	-0.791422
O	-7.333166	-0.448386	-1.093615
O	-1.078028	1.074888	-2.247695
N	-0.925414	-0.106022	-0.326471
C	-0.639763	1.061152	0.501336
C	0.890667	1.180546	0.586171
C	-1.283514	0.954448	1.876372
C	-2.781751	0.771566	1.820506
C	-3.390929	-0.234752	2.558199
C	-4.761060	-0.420371	2.501953
C	-5.577197	0.374728	1.702451
C	-4.951640	1.399831	0.991830
C	-3.584745	1.595921	1.039541
Cl	-5.482939	-1.725343	3.403783
O	-6.908298	0.143053	1.659816
Cl	-5.931743	2.453574	0.002857
N	1.466704	1.657793	-0.544881
O	1.523747	0.797951	1.557225
H	2.793413	-2.328703	-2.799277
H	2.703810	-4.334180	-0.894232
H	4.216182	-3.611801	-1.456223
H	5.393917	-0.107086	0.440825
H	4.656066	-5.162485	1.730130
H	7.036752	-5.322602	2.446379
H	8.559796	-3.415321	2.095077
H	7.760089	-1.330712	1.025247
H	1.676565	-3.059720	0.895396
H	0.860874	-1.393775	-3.300139
H	3.390362	1.241779	0.057517
H	-1.323251	-1.001137	-3.480728
H	-1.739505	-2.834189	-1.084103
H	-2.055861	-3.171013	-2.770057
H	2.925766	2.717712	-2.568001
H	4.498074	2.247286	-1.946306
H	5.296763	3.224244	0.275836
H	5.376866	5.257024	1.659574
H	1.911505	6.764297	-0.348398
H	1.831294	4.720965	-1.737611
H	4.365099	7.271172	2.105477
H	-3.474837	-0.476361	-3.490349

H	-5.738778	0.356644	-3.092947
H	-6.147536	-2.293716	0.243006
H	-3.848014	-3.107267	-0.127523
H	-7.614474	0.194519	-1.752884
H	-0.770113	-1.024767	0.070786
H	-1.028299	1.926468	-0.036268
H	-1.034971	1.862271	2.432248
H	-0.830975	0.125285	2.423294
H	-2.795932	-0.896261	3.176786
H	-3.157599	2.405390	0.459333
H	-7.232722	0.228988	0.746259
H	0.850355	1.869107	-1.321597

ωB97X Energy = -3265.42032211 a.u.

(L-Tyr[1],D-Tyr[2])-**1**, Conf L

N	-1.654324	1.509449	1.011704
C	-3.039879	1.028292	0.962821
C	-3.767797	2.118615	0.187165
C	-2.716092	2.747931	-0.750485
C	-1.362413	2.527026	-0.002371
C	-2.801444	4.246557	-0.827660
C	-1.790132	4.807556	-0.055472
N	-1.007425	3.821952	0.565844
C	-3.704110	5.044346	-1.502434
C	-3.579745	6.427871	-1.407745
C	-2.561484	6.983773	-0.639141
C	-1.655366	6.184565	0.049557
O	-2.732068	2.181964	-2.040321
H	-0.604588	2.160778	-0.696818
C	-0.775449	0.964070	1.884939
O	-1.140309	0.260803	2.819554
N	-3.016364	-1.408287	1.005845
C	-2.994050	-0.320550	0.227337
C	0.717978	1.056494	1.542376
O	-2.860652	-0.370211	-0.999261
C	-2.745535	-2.783234	0.573293
C	-1.225160	-2.955674	0.426593
C	-3.564133	-3.260576	-0.633419
C	1.616455	1.374149	2.736371
C	3.064501	1.380746	2.311260
C	3.871565	0.258276	2.477028
C	5.163825	0.218871	1.975747
C	5.663712	1.318169	1.292955
C	4.889734	2.459316	1.138642
C	3.599687	2.480951	1.647703
O	6.931958	1.218239	0.769751
C	-5.049342	-3.147343	-0.395083
C	-5.787627	-2.103643	-0.941076
C	-7.150698	-1.983653	-0.704497
C	-7.795181	-2.919589	0.093906
C	-7.074442	-3.971870	0.648084
C	-5.716206	-4.076585	0.402017
O	-9.131290	-2.860421	0.363208
O	-0.589853	-3.557697	1.278270
N	-0.644576	-2.356692	-0.646223
C	0.781057	-2.047253	-0.631225
C	0.867042	-0.551025	-0.313113
C	1.523980	-2.387302	-1.921109
C	2.994869	-2.105895	-1.720327
C	3.794619	-3.018097	-1.039828
C	5.099121	-2.701072	-0.711773
C	5.661553	-1.467602	-1.041528
C	4.861582	-0.589723	-1.772954
C	3.550198	-0.889706	-2.096531
Cl	6.061945	-3.852757	0.174387
O	6.933719	-1.198522	-0.679998
Cl	5.536340	0.938408	-2.282697
N	1.022606	-0.259105	0.995081
O	0.713480	0.296863	-1.180912
H	-3.433144	0.904213	1.970682
H	-4.623556	1.746446	-0.375728
H	-4.121208	2.866506	0.896977
H	-0.018803	4.023136	0.608474
H	-4.486046	4.597754	-2.107341
H	-4.271754	7.072740	-1.936130
H	-2.468794	8.062241	-0.574806
H	-0.864214	6.624867	0.645669
H	-2.782826	1.216745	-1.918701
H	-3.005063	-1.248068	2.002387
H	0.906037	1.774166	0.746495
H	-3.009561	-3.407817	1.426031
H	-3.291281	-4.306715	-0.795630
H	-3.290174	-2.708405	-1.530349
H	1.441338	0.638669	3.524876
H	1.324808	2.350064	3.131954
H	3.483546	-0.609666	3.000278
H	5.784114	-0.662213	2.098828
H	5.285759	3.321619	0.612659
H	2.996301	3.373784	1.512858
H	7.189962	2.053850	0.366396
H	-5.290968	-1.367647	-1.565367
H	-7.710763	-1.163201	-1.142001
H	-7.589030	-4.700509	1.263559
H	-5.165162	-4.906747	0.833998
H	-9.522304	-2.101469	-0.079515
H	-1.236849	-1.772294	-1.225946
H	1.209181	-2.627249	0.186460
H	1.126548	-1.791321	-2.744220
H	1.357519	-3.441462	-2.148878
H	3.396149	-3.981007	-0.740343
H	2.954454	-0.159284	-2.630053
H	7.025944	-0.264695	-0.415492
H	1.043115	-1.026299	1.651787

ωB97X Energy = -3265.42024325 a.u.

(L-Tyr[1],D-Tyr[2])-**1**, Conf M

N	-1.653651	1.515341	1.008511
C	-3.040495	1.038127	0.955015
C	-3.763131	2.131099	0.178119
C	-2.706644	2.758991	-0.755149

C	-1.356574	2.535196	-0.001676
C	-2.789026	4.257813	-0.831562
C	-1.781013	4.816318	-0.053248
N	-1.003459	3.828656	0.571335
C	-3.686713	5.057762	-1.510368
C	-3.560609	6.440994	-1.413484
C	-2.545680	6.994405	-0.638721
C	-1.644618	6.192977	0.054055
O	-2.718511	2.193750	-2.045302
H	-0.595931	2.170301	-0.693744
C	-0.778675	0.966113	1.883265
O	-1.148006	0.262271	2.815715
N	-3.021178	-1.398454	0.996371
C	-2.996032	-0.310230	0.218493
C	0.715828	1.054743	1.544483
O	-2.861276	-0.359397	-1.007927
C	-2.750656	-2.773335	0.563411
C	-1.230238	-2.945995	0.418026
C	-3.569480	-3.250286	-0.643245
C	1.612826	1.365717	2.741370
C	3.061816	1.368667	2.319366
C	3.864551	0.242745	2.482858
C	5.157716	0.200445	1.984156
C	5.663054	1.300392	1.306411
C	4.893497	2.444857	1.154622
C	3.602407	2.469279	1.660893
O	6.932085	1.197934	0.785638
C	-5.054573	-3.140850	-0.401919
C	-5.717541	-4.075003	0.388129
C	-7.077686	-3.972509	0.639333
C	-7.799216	-2.916206	0.095279
C	-7.156303	-1.974475	-0.698424
C	-5.796401	-2.091666	-0.939086
O	-9.138527	-2.759641	0.302718
O	-0.595772	-3.546009	1.271835
N	-0.648408	-2.349631	-0.655561
C	0.777664	-2.041988	-0.639378
C	0.865989	-0.546954	-0.316158
C	1.520672	-2.379257	-1.929898
C	2.992179	-2.103176	-1.726115
C	3.551741	-0.887058	-2.096231
C	4.863443	-0.592344	-1.769126
C	5.659532	-1.475700	-1.040034
C	5.092987	-2.708974	-0.716719
C	3.788132	-3.020729	-1.048363
Cl	5.543517	0.935932	-2.271291
O	6.931812	-1.211804	-0.674900
Cl	6.050945	-3.867313	0.166061
N	1.017321	-0.260065	0.993636
O	0.717616	0.304359	-1.181498
H	-3.437219	0.914170	1.961543
H	-4.617794	1.761597	-0.388169
H	-4.117267	2.879056	0.887486
H	-0.014836	4.028462	0.620319
H	-4.466075	4.613062	-2.119965
H	-4.248690	7.087548	-1.944930

H	-2.451603	8.072652	-0.572693
H	-0.856036	6.631368	0.654963
H	-2.774059	1.228678	-1.924723
H	-3.010382	-1.238645	1.992999
H	0.907858	1.774376	0.751338
H	-3.015248	-3.398112	1.415829
H	-3.294500	-4.295468	-0.807797
H	-3.298076	-2.695756	-1.539525
H	1.433457	0.628575	3.527351
H	1.323705	2.341439	3.139252
H	3.472352	-0.625639	3.002234
H	5.774525	-0.683332	2.105417
H	5.293766	3.307689	0.632728
H	3.002599	3.364761	1.527782
H	7.193672	2.033929	0.385382
H	-5.166193	-4.908472	0.813110
H	-7.577720	-4.714188	1.253970
H	-7.729822	-1.158146	-1.121870
H	-5.300579	-1.351675	-1.559444
H	-9.469644	-3.469672	0.860500
H	-1.239637	-1.765172	-1.236282
H	1.204679	-2.625174	0.176590
H	1.126064	-1.778966	-2.751216
H	1.351299	-3.432043	-2.161822
H	2.959105	-0.152625	-2.627690
H	3.386309	-3.983732	-0.753682
H	7.025696	-0.279818	-0.404587
H	1.034267	-1.029629	1.647711

ωB97X Energy = -3265.42019970 a.u.

Table S15. Cartesian coordinates and energies of the low-energy conformers calculated at the ωB97X/TZVP PCM/MeOH level.

(*R*)-**3**, Conf A

C	-8.531139	-0.556650	-0.569876
C	-7.138999	-0.017023	-0.872312
C	-6.043363	-0.750015	-0.107228
C	-4.646913	-0.214997	-0.400290
C	-3.563904	-0.957889	0.372360
O	6.175999	-1.742450	-0.393800
O	1.569797	2.693675	-0.166788
O	-0.928273	2.722339	0.926326
O	4.084080	2.435545	-0.588198
C	-1.081289	-1.251442	0.798731
C	-2.161731	-0.462727	0.090227
O	-2.100306	0.916932	0.533739
C	-0.925969	1.559651	0.610218
C	1.405864	-1.397453	0.410884
C	0.252661	-0.623909	0.522045
C	0.309234	0.755718	0.357226
C	1.534242	1.381308	0.031212
C	3.992670	1.217714	-0.417522
C	5.179833	0.379181	-0.535605
C	5.119585	-0.940086	-0.322540
C	3.820435	-1.628594	-0.025160
C	2.604744	-0.788345	0.117913
C	2.689660	0.591903	-0.105713
O	3.825756	-2.834377	0.094669
H	-9.300225	-0.018500	-1.127774
H	-8.763410	-0.463350	0.494463
H	-8.605758	-1.615091	-0.833739
H	-6.939801	-0.093515	-1.946771
H	-7.096141	1.049622	-0.626801
H	-6.242082	-0.674196	0.968668
H	-6.083678	-1.818202	-0.352823
H	-4.444080	-0.295832	-1.474690
H	-4.606012	0.850266	-0.153406
H	-3.748815	-0.884839	1.449445
H	-3.592074	-2.021679	0.115384
H	5.870540	-2.650374	-0.229237
H	2.503270	2.928701	-0.382297
H	-1.090043	-2.286038	0.454268
H	-1.278681	-1.257982	1.876022
H	-1.972677	-0.464315	-0.989299
H	1.364738	-2.470881	0.547990
H	6.114762	0.874823	-0.764417

ωB97X Energy = -1148.25281313 a.u.

(*R*)-**3**, Conf B

C	-8.125955	-1.665663	-0.402839
C	-6.608549	-1.746186	-0.510390
C	-5.920390	-0.442404	-0.123738
C	-4.401860	-0.511732	-0.235578
C	-3.729821	0.794199	0.175562
O	5.367829	-2.877218	-0.051944
O	2.257920	2.702659	-0.439703
O	-0.140328	3.561030	0.538841
O	4.594415	1.686230	-0.721334
C	-1.447573	-0.188855	0.756316
C	-2.235278	0.802751	-0.073633
O	-1.780952	2.143108	0.244106
C	-0.471690	2.422583	0.324578
C	0.895060	-1.086340	0.500271
C	0.017013	-0.005011	0.488042
C	0.478636	1.275071	0.202836
C	1.838034	1.483444	-0.123337
C	4.148163	0.570647	-0.444303
C	5.039475	-0.583366	-0.432105
C	4.591890	-1.800101	-0.102348
C	3.143319	-2.049116	0.197726
C	2.224747	-0.882860	0.208902
C	2.714009	0.383884	-0.135040
O	2.793981	-3.186345	0.427878
H	-8.598374	-2.609478	-0.682700
H	-8.523302	-0.885762	-1.057989
H	-8.433088	-1.428220	0.619257
H	-6.236390	-2.552875	0.130413
H	-6.325595	-2.011574	-1.534823
H	-6.294049	0.367451	-0.761372
H	-6.196734	-0.178084	0.903976
H	-4.037764	-1.335304	0.387703
H	-4.125753	-0.756101	-1.268101
H	-4.166584	1.623262	-0.388741
H	-3.911588	0.996236	1.236178
H	4.806766	-3.635741	0.181953
H	3.222812	2.635485	-0.632918
H	-1.745197	-1.208016	0.509229
H	-1.661655	-0.028320	1.818693
H	-2.033453	0.634107	-1.137434
H	0.539361	-2.082851	0.730780
H	6.082287	-0.405589	-0.662373

ωB97X Energy = -1148.25235570 a.u.

(*R*)-**3**, Conf C

C	6.490749	-0.116878	2.821506
C	6.336579	-0.037359	1.308089
C	5.023860	-0.636293	0.815633
C	4.866019	-0.548939	-0.698883
C	3.627032	-1.255427	-1.244073
O	-5.937862	-1.496446	0.880059
O	-1.154153	2.670144	0.038988
O	1.149791	2.570362	-1.423520
O	-3.595556	2.550622	0.809294

C	1.098119	-1.402863	-1.319758
C	2.308700	-0.683216	-0.764348
O	2.265815	0.702054	-1.191651
C	1.129511	1.408585	-1.104530
C	-1.313558	-1.415313	-0.580048
C	-0.145847	-0.706430	-0.853121
C	-0.099557	0.672107	-0.676704
C	-1.225911	1.361300	-0.172953
C	-3.601609	1.332410	0.618354
C	-4.808979	0.562440	0.893403
C	-4.855548	-0.755514	0.668853
C	-3.650356	-1.514704	0.197632
C	-2.421208	-0.743103	-0.115606
C	-2.393988	0.637103	0.123179
O	-3.740679	-2.717531	0.082251
H	7.437550	0.315497	3.151611
H	6.460324	-1.155089	3.163160
H	5.683702	0.421681	3.325518
H	6.396903	1.007838	0.985887
H	7.171225	-0.556703	0.824853
H	4.965573	-1.686436	1.127870
H	4.194470	-0.120298	1.312963
H	4.851787	0.501441	-1.005631
H	5.745547	-0.996174	-1.172795
H	3.632766	-1.227367	-2.337896
H	3.646540	-2.310356	-0.951770
H	-5.711495	-2.418604	0.672576
H	-2.031771	2.955476	0.387316
H	1.096677	-2.441168	-0.986131
H	1.141963	-1.405289	-2.414139
H	2.262618	-0.686044	0.329526
H	-1.353728	-2.487708	-0.725494
H	-5.673527	1.108221	1.249477

ωB97X Energy = -1148.25218258 a.u.

(*R*)-**3**, Conf D

C	-8.087095	-1.480305	-0.312441
C	-6.595833	-1.284287	-0.555847
C	-6.140398	0.141308	-0.259159
C	-4.675302	0.418227	-0.588140
C	-3.698476	-0.402749	0.247742
O	5.935709	-2.150127	-0.219864
O	1.773559	2.710497	-0.275014
O	-0.725795	3.016901	0.776618
O	4.260290	2.197396	-0.623479
C	-1.267781	-0.922288	0.755587
C	-2.247217	-0.055340	-0.006712
O	-2.061641	1.325116	0.398923
C	-0.832043	1.851589	0.489002
C	1.203021	-1.321878	0.441298
C	0.128016	-0.437471	0.496607
C	0.322841	0.923464	0.288756
C	1.608701	1.416028	-0.029546
C	4.048832	1.001491	-0.409697
C	5.152435	0.050054	-0.464377
C	4.960137	-1.248487	-0.206662
C	3.593646	-1.798723	0.077061
C	2.461730	-0.841554	0.159578
C	2.685038	0.515206	-0.109301
O	3.480119	-2.994461	0.237161
H	-8.397434	-2.505608	-0.524109
H	-8.677533	-0.813797	-0.946914
H	-8.344427	-1.261286	0.727478
H	-6.034403	-1.992216	0.061811
H	-6.356753	-1.526663	-1.597577
H	-6.765758	0.835513	-0.830802
H	-6.322829	0.365870	0.798868
H	-4.496517	0.216238	-1.650954
H	-4.468629	1.480314	-0.432712
H	-3.907628	-0.266874	1.314714
H	-3.815676	-1.468617	0.032394
H	5.540093	-3.017894	-0.032012
H	2.729576	2.847469	-0.475691
H	-1.369740	-1.961888	0.442643
H	-1.491976	-0.873845	1.826539
H	-2.030690	-0.109503	-1.079753
H	1.054811	-2.380804	0.613004
H	6.136067	0.445304	-0.684179

ωB97X Energy = -1148.25198506 a.u.

(*R*)-**3**, Conf E

C	7.436500	0.913599	0.639062
C	7.189158	-0.562822	0.930084
C	6.048826	-1.170130	0.115134
C	4.682455	-0.558273	0.407389
C	3.562549	-1.260792	-0.350626
O	-6.200719	-1.494415	0.452608
O	-1.363063	2.678904	0.082500
O	1.130790	2.538738	-1.015119
O	-3.886384	2.568885	0.517609
C	1.067373	-1.429693	-0.775425
C	2.188896	-0.683165	-0.085005
O	2.202907	0.685677	-0.564498
C	1.065248	1.388281	-0.663216
C	-1.423865	-1.429842	-0.382967
C	-0.230442	-0.723751	-0.515637
C	-0.211692	0.660834	-0.388046
C	-1.399805	1.361272	-0.078477
C	-3.861682	1.343556	0.381502
C	-5.091273	0.573756	0.529707
C	-5.103439	-0.752184	0.352907
C	-3.845823	-1.517235	0.063894
C	-2.586841	-0.748533	-0.104287
C	-2.595942	0.639905	0.082019
O	-3.918036	-2.723285	-0.028553
H	8.308005	1.281934	1.184294
H	6.583615	1.531935	0.927627
H	7.617285	1.074280	-0.427707
H	8.104631	-1.126264	0.726341
H	6.978443	-0.695995	1.997062

H	6.270951	-1.060849	-0.953451
H	6.004196	-2.247030	0.311802
H	4.482418	-0.616262	1.484271
H	4.682038	0.502757	0.144754
H	3.753352	-1.223571	-1.428583
H	3.532550	-2.318150	-0.068490
H	-5.945755	-2.421380	0.309414
H	-2.281644	2.970331	0.292915
H	1.019884	-2.454371	-0.405422
H	1.264177	-1.473323	-1.851998
H	1.999065	-0.646541	0.993779
H	-1.441835	-2.507162	-0.491685
H	-5.996535	1.124936	0.750632

ωB97X Energy = -1148.25197382 a.u.

(*R*)-**3**, Conf F

C	8.209294	-0.943587	-0.718941
C	6.721277	-0.787643	-1.005618
C	5.894723	-0.587862	0.259227
C	4.405167	-0.421058	-0.018392
C	3.598527	-0.239001	1.262684
O	-5.902118	-2.160466	-0.233851
O	-1.808415	2.754514	-0.036042
O	0.869088	2.958549	-0.518035
O	-4.315163	2.238286	-0.174175
C	1.350039	-0.913012	0.233361
C	2.129593	0.083513	1.066318
O	2.061358	1.383091	0.423724
C	0.893325	1.855018	-0.034696
C	-1.133470	-1.324416	0.054696
C	-0.064648	-0.432245	0.100175
C	-0.286816	0.939220	0.038842
C	-1.605967	1.442276	-0.024196
C	-4.073448	1.029650	-0.138455
C	-5.171061	0.070456	-0.179103
C	-4.939322	-1.247052	-0.174100
C	-3.549755	-1.799255	-0.052427
C	-2.418288	-0.838218	-0.032296
C	-2.682114	0.537652	-0.043136
O	-3.415665	-3.001865	0.011571
H	8.782110	-1.086125	-1.637594
H	8.394443	-1.805660	-0.072393
H	8.604346	-0.058709	-0.212645
H	6.562743	0.064107	-1.675903
H	6.354556	-1.671282	-1.539142
H	6.047003	-1.442451	0.929678
H	6.263839	0.293974	0.796456
H	4.252450	0.443669	-0.672458
H	4.046727	-1.298349	-0.567503
H	3.654584	-1.145845	1.873129
H	4.035956	0.566005	1.860998
H	-5.483167	-3.036326	-0.187541
H	-2.783465	2.896633	-0.079157
H	1.376239	-1.895224	0.707134
H	1.801285	-1.009656	-0.758721
H	1.651624	0.184904	2.045998
H	-0.961545	-2.392961	0.091799
H	-6.174733	0.469805	-0.251328

ωB97X Energy = -1148.25195369 a.u.

(*R*)-**3**, Conf G

C	7.270345	-0.197515	1.957318
C	7.259992	-0.141464	0.433384
C	6.094560	-0.895822	-0.203630
C	4.722914	-0.320894	0.135551
C	3.598809	-1.045924	-0.594613
O	-6.121943	-1.660867	0.522849
O	-1.445260	2.690844	0.141660
O	1.003674	2.674955	-1.061050
O	-3.946587	2.476607	0.658796
C	1.096961	-1.298656	-0.925104
C	2.216884	-0.527065	-0.260222
O	2.161536	0.852883	-0.704001
C	0.995687	1.514240	-0.737836
C	-1.376023	-1.402239	-0.442045
C	-0.214761	-0.648720	-0.598162
C	-0.241464	0.731680	-0.433066
C	-1.441483	1.378393	-0.059282
C	-3.884114	1.258095	0.480198
C	-5.082538	0.441633	0.633755
C	-5.053358	-0.878371	0.418733
C	-3.776955	-1.590664	0.081321
C	-2.552240	-0.772209	-0.104766
C	-2.604656	0.609431	0.121078
O	-3.807559	-2.796380	-0.035339
H	6.399444	0.302430	2.387090
H	8.160321	0.287656	2.363502
H	7.265395	-1.233736	2.307785
H	7.233596	0.903419	0.104823
H	8.197503	-0.558308	0.053439
H	6.218899	-0.890414	-1.292044
H	6.132827	-1.947571	0.105789
H	4.544456	-0.385210	1.214164
H	4.701796	0.742545	-0.123451
H	3.743823	-0.978011	-1.678090
H	3.618916	-2.109721	-0.336471
H	-5.839120	-2.574413	0.349449
H	-2.365052	2.942284	0.394044
H	1.101976	-2.332690	-0.578958
H	1.252556	-1.310140	-2.009187
H	2.069363	-0.523795	0.825723
H	-1.358666	-2.476245	-0.579771
H	-6.000288	0.954021	0.893156

ωB97X Energy = -1148.25193463 a.u.

(*R*)-**3**, Conf H

C	8.172697	-0.883700	-0.349967
C	6.716559	-0.437932	-0.399052
C	6.049311	-0.477675	0.972696

C	4.622038	0.064709	0.994591
C	3.646188	-0.756925	0.158098
O	-6.083928	-1.887617	0.160711
O	-1.634465	2.712234	0.116608
O	0.943374	2.796064	-0.777433
O	-4.161715	2.371453	0.370508
C	1.223520	-1.159899	-0.478274
C	2.207654	-0.305773	0.292066
O	2.139021	1.053449	-0.210059
C	0.954231	1.647317	-0.414017
C	-1.281952	-1.386737	-0.299441
C	-0.150862	-0.574587	-0.341838
C	-0.269575	0.806785	-0.235869
C	-1.538450	1.396742	-0.035743
C	-4.017301	1.155031	0.229297
C	-5.181545	0.277765	0.269330
C	-5.056084	-1.046614	0.128703
C	-3.717266	-1.692227	-0.076769
C	-2.521531	-0.813743	-0.128628
C	-2.674618	0.570708	0.021729
O	-3.675101	-2.897681	-0.192055
H	8.756897	-0.238568	0.311792
H	8.635041	-0.855011	-1.338789
H	8.254897	-1.906600	0.027637
H	6.655448	0.581311	-0.796898
H	6.168399	-1.075298	-1.099752
H	6.053262	-1.509018	1.346372
H	6.656558	0.104277	1.674418
H	4.264320	0.087435	2.029608
H	4.620820	1.100709	0.641936
H	3.917830	-0.727523	-0.901337
H	3.689242	-1.805759	0.470645
H	-5.735102	-2.786735	0.039317
H	-2.588943	2.918379	0.256514
H	1.238507	-2.180784	-0.095164
H	1.515825	-1.195671	-1.533158
H	1.920946	-0.275029	1.349413
H	-1.192183	-2.462139	-0.389965
H	-6.147326	0.743335	0.419699

ωB97X Energy = -1148.25191236 a.u.

(*R*)-**3**, Conf I

C	5.640741	-2.913066	1.842431
C	5.613585	-2.157692	0.519958
C	4.722740	-0.921356	0.563882
C	4.683058	-0.176246	-0.766921
C	3.933869	1.154581	-0.725934
O	-4.990927	-2.901155	-0.196646
O	-1.980896	2.632900	0.927642
O	0.270466	3.767506	-0.103184
O	-4.244534	1.478555	1.262693
C	1.622582	0.190364	-1.213184
C	2.481846	1.040405	-0.302138
O	1.963583	2.395190	-0.296921
C	0.648862	2.625261	-0.165430
C	-0.652089	-0.842623	-0.875023
C	0.197015	0.254686	-0.750119
C	-0.255343	1.434544	-0.169711
C	-1.572341	1.516099	0.337485
C	-3.808821	0.457414	0.726094
C	-4.670290	-0.710682	0.585680
C	-4.237122	-1.821405	-0.021160
C	-2.826640	-1.945719	-0.516864
C	-1.942177	-0.758655	-0.402988
C	-2.416267	0.396563	0.231714
O	-2.479719	-3.002438	-0.997692
H	6.009732	-2.274387	2.649444
H	6.287601	-3.791104	1.789110
H	4.639025	-3.252285	2.119689
H	6.630398	-1.856589	0.245981
H	5.264519	-2.822954	-0.277331
H	3.711881	-1.227467	0.858782
H	5.079711	-0.243513	1.348937
H	5.708743	0.029246	-1.087971
H	4.254180	-0.826504	-1.537190
H	4.432234	1.834575	-0.028951
H	3.963348	1.627346	-1.712039
H	-4.445487	-3.576608	-0.633594
H	-2.914866	2.486629	1.209154
H	1.969853	-0.843346	-1.202610
H	1.704112	0.556676	-2.242458
H	2.409456	0.666185	0.724062
H	-0.303884	-1.759563	-1.334066
H	-5.683575	-0.625633	0.957290

ωB97X Energy = -1148.25170289 a.u.

(*R*)-**3**, Conf J

C	5.482444	-1.888074	2.629595
C	5.256453	-1.617371	1.147351
C	5.114044	-0.129335	0.842996
C	5.012588	0.205548	-0.644770
C	3.841535	-0.444471	-1.382000
O	-5.703638	-1.943979	0.266583
O	-1.250405	2.626034	0.754504
O	1.070458	3.096546	-0.602041
O	-3.681970	2.121088	1.388608
C	1.333820	-0.750603	-1.576428
C	2.476921	-0.118212	-0.811675
O	2.328251	1.323489	-0.854912
C	1.138967	1.893703	-0.612127
C	-1.080080	-1.135655	-0.954208
C	0.032683	-0.298000	-0.982746
C	-0.032078	0.980116	-0.438647
C	-1.215869	1.422617	0.194460
C	-3.590789	1.005709	0.870820
C	-4.737617	0.105138	0.884443
C	-4.679496	-1.098490	0.303570
C	-3.414164	-1.608824	-0.320071
C	-2.243347	-0.696939	-0.363801
C	-2.326974	0.562584	0.243766

O	-3.410656	-2.734243	-0.769187
H	4.650429	-1.505227	3.226935
H	6.394839	-1.398975	2.981121
H	5.575596	-2.956779	2.833266
H	6.091306	-2.023912	0.565327
H	4.362039	-2.155383	0.816133
H	4.244365	0.268034	1.379821
H	5.981165	0.398321	1.254303
H	4.948739	1.290263	-0.763600
H	5.936316	-0.105703	-1.142904
H	3.857098	-0.139002	-2.432511
H	3.942573	-1.533692	-1.371341
H	-5.403907	-2.753743	-0.179699
H	-2.151073	2.740150	1.140098
H	1.412151	-1.837422	-1.530010
H	1.390041	-0.457877	-2.630348
H	2.418819	-0.417770	0.240374
H	-1.035284	-2.127045	-1.387723
H	-5.645775	0.467628	1.349143

ωB97X Energy = -1148.25160429 a.u.

(*R*)-**3**, Conf K

C	6.858304	0.446220	-0.942566
C	6.731505	-0.926615	-0.291928
C	6.069672	-0.894803	1.086633
C	4.664601	-0.293595	1.116923
C	3.669625	-1.034643	0.230349
O	-6.102644	-1.671245	0.034078
O	-1.428368	2.696752	0.206855
O	1.167389	2.675384	-0.646955
O	-3.975799	2.478657	0.388780
C	1.242426	-1.294488	-0.465418
C	2.252478	-0.517112	0.351428
O	2.262249	0.859031	-0.107844
C	1.113135	1.518070	-0.315954
C	-1.273826	-1.400799	-0.328243
C	-0.103331	-0.645693	-0.330340
C	-0.154385	0.736006	-0.181403
C	-1.395012	1.382518	0.020208
C	-3.887386	1.258247	0.236245
C	-5.092517	0.437272	0.252440
C	-5.032532	-0.884286	0.053757
C	-3.722172	-1.592732	-0.124079
C	-2.485752	-0.771455	-0.156238
C	-2.570435	0.611538	0.050090
O	-3.734976	-2.798632	-0.242361
H	7.412599	0.387508	-1.881490
H	7.386231	1.142783	-0.284836
H	5.879950	0.879601	-1.165361
H	6.176263	-1.596210	-0.956791
H	7.726453	-1.368947	-0.185597
H	6.029221	-1.914580	1.485064
H	6.706580	-0.322196	1.769536
H	4.298352	-0.309239	2.148960
H	4.700767	0.758376	0.821118
H	3.966271	-0.977176	-0.821732
H	3.655511	-2.095982	0.500371
H	-5.797075	-2.584373	-0.098381
H	-2.374344	2.947633	0.329671
H	1.199539	-2.327728	-0.119371
H	1.552169	-1.307533	-1.515875
H	1.945616	-0.505169	1.403462
H	-1.236357	-2.475822	-0.453378
H	-6.036963	0.947586	0.392782

ωB97X Energy = -1148.25158392 a.u.

(*R*)-**3**, Conf L

C	8.043798	-1.091976	-0.328434
C	6.536222	-0.920228	-0.466289
C	5.813166	-1.034433	0.872272
C	4.290668	-0.978482	0.768777
C	3.772254	0.350073	0.222707
O	-5.594071	-2.568202	-0.002640
O	-2.066339	2.766266	0.224657
O	0.464789	3.357527	-0.606962
O	-4.494241	1.960580	0.386071
C	1.462841	-0.492324	-0.455032
C	2.270326	0.497368	0.357837
O	1.953833	1.840114	-0.089530
C	0.681961	2.215387	-0.290557
C	-0.958804	-1.179324	-0.324110
C	0.003562	-0.172125	-0.319207
C	-0.368397	1.158709	-0.165062
C	-1.726609	1.497173	0.033220
C	-4.122886	0.795221	0.229787
C	-5.104062	-0.283614	0.230802
C	-4.736519	-1.554070	0.029884
C	-3.295430	-1.937963	-0.131384
C	-2.284616	-0.850764	-0.156080
C	-2.690187	0.473554	0.053722
O	-3.025432	-3.113700	-0.245393
H	8.463030	-0.334461	0.339361
H	8.547765	-1.002172	-1.292904
H	8.289103	-2.072785	0.087773
H	6.323988	0.051683	-0.922423
H	6.139563	-1.676683	-1.153003
H	6.098121	-1.979400	1.347054
H	6.160775	-0.237278	1.540299
H	3.947940	-1.803031	0.134422
H	3.859759	-1.149480	1.761234
H	4.240883	1.175828	0.766876
H	4.037521	0.468150	-0.831952
H	-5.083127	-3.384651	-0.133343
H	-3.045369	2.789527	0.342390
H	1.653483	-1.508127	-0.108226
H	1.767297	-0.437393	-1.506005
H	1.981184	0.429321	1.412612
H	-0.671320	-2.215445	-0.452681
H	-6.142634	-0.007366	0.361770

ωB97X Energy = -1148.25154140 a.u.

(*R*)-**3**, Conf M

C	6.596960	-2.277300	1.436074
C	6.742043	-1.682367	0.039466
C	6.009536	-0.354929	-0.144193
C	4.494322	-0.456544	0.004795
C	3.801211	0.865863	-0.307296
O	-5.264182	-2.881822	-0.015189
O	-2.180283	2.694552	0.574053
O	0.187205	3.620539	-0.414355
O	-4.500518	1.646474	0.869222
C	1.509339	-0.103888	-0.870245
C	2.315087	0.848919	-0.012728
O	1.843257	2.200650	-0.244659
C	0.530750	2.474404	-0.272749
C	-0.819868	-1.030376	-0.597697
C	0.051758	0.055357	-0.552461
C	-0.409342	1.315255	-0.187541
C	-1.760907	1.496386	0.184834
C	-4.055122	0.550224	0.522295
C	-4.939104	-0.608538	0.475327
C	-4.493593	-1.803026	0.069932
C	-3.052443	-2.025121	-0.282444
C	-2.142049	-0.852614	-0.259308
C	-2.629205	0.390850	0.163807
O	-2.702265	-3.146416	-0.579743
H	5.561088	-2.544454	1.657095
H	6.926184	-1.564822	2.197949
H	7.198677	-3.182178	1.543223
H	7.803533	-1.529443	-0.176638
H	6.378049	-2.399545	-0.704606
H	6.393663	0.374086	0.579313
H	6.239332	0.045661	-1.137459
H	4.125664	-1.243074	-0.662953
H	4.238517	-0.765884	1.023835
H	4.250694	1.663571	0.291366
H	3.948818	1.131844	-1.359067
H	-4.705544	-3.623211	-0.303405
H	-3.138894	2.609405	0.790379
H	1.819861	-1.132123	-0.683939
H	1.692495	0.112161	-1.928520
H	2.146589	0.622572	1.046119
H	-0.464608	-2.010654	-0.890181
H	-5.976059	-0.451065	0.743663

ωB97X Energy = -1148.25152703 a.u.

(*R*)-**3**, Conf N

C	6.524472	-1.881270	1.528960
C	6.674261	-1.315929	0.121025
C	6.228388	0.141275	-0.006951
C	4.772481	0.412697	0.371248
C	3.771671	-0.365305	-0.476236
O	-5.833247	-2.148968	0.242117
O	-1.671111	2.710041	0.420123
O	0.793033	3.083604	-0.698258
O	-4.134860	2.163888	0.868834
C	1.325115	-0.847365	-0.949319
C	2.328940	-0.033198	-0.160883
O	2.135495	1.370443	-0.471641
C	0.905001	1.902271	-0.489269
C	-1.135169	-1.266685	-0.580133
C	-0.060684	-0.380619	-0.614054
C	-0.245583	0.964095	-0.312564
C	-1.518443	1.434927	0.082248
C	-3.938882	0.989847	0.546862
C	-5.042499	0.036946	0.574927
C	-4.860511	-1.244156	0.235518
C	-3.515681	-1.763915	-0.180263
C	-2.381391	-0.806751	-0.220140
C	-2.594958	0.532280	0.132649
O	-3.418796	-2.937601	-0.465277
H	7.049961	-1.257345	2.257713
H	6.936155	-2.890482	1.594342
H	5.476535	-1.934390	1.834669
H	7.722721	-1.382705	-0.184100
H	6.117422	-1.940409	-0.585280
H	6.872517	0.763082	0.624183
H	6.393587	0.474647	-1.037370
H	4.608729	0.175530	1.428159
H	4.575468	1.482284	0.262846
H	3.950221	-0.178249	-1.540997
H	3.894414	-1.440893	-0.316614
H	-5.451575	-2.997418	-0.039308
H	-2.615636	2.829813	0.678067
H	1.434620	-1.905746	-0.710658
H	1.516156	-0.726202	-2.020906
H	2.142639	-0.156362	0.912169
H	-0.995143	-2.312441	-0.824117
H	-6.012063	0.411151	0.878259

ωB97X Energy = -1148.25150316 a.u.

(*R*)-**3**, Conf O

C	-6.490164	2.724263	-0.037712
C	-6.761074	1.457849	0.767670
C	-6.016077	0.227117	0.254445
C	-4.497581	0.333194	0.358165
C	-3.801566	-0.944326	-0.100041
O	5.201450	2.943298	0.117439
O	2.235659	-2.722012	0.377189
O	-0.155058	-3.614957	-0.592821
O	4.539279	-1.647588	0.710207
C	-1.553616	0.107626	-0.695078
C	-2.302990	-0.922863	0.123010
O	-1.823167	-2.243960	-0.235893
C	-0.509484	-2.490152	-0.345772
C	0.772368	1.053529	-0.459346
C	-0.080403	-0.048118	-0.457911
C	0.416187	-1.323796	-0.215584
C	1.785375	-1.507976	0.083245

C	4.073640	-0.544989	0.413649
C	4.944161	0.624514	0.383602
C	4.456497	1.843322	0.126148
C	3.011650	2.054432	-0.218011
C	2.110536	0.874863	-0.191987
C	2.641938	-0.393408	0.075758
O	2.646650	3.176904	-0.492152
H	-7.105239	3.554436	0.315579
H	-6.716967	2.567303	-1.096195
H	-5.445983	3.036558	0.035351
H	-6.495984	1.626453	1.817343
H	-7.834593	1.247742	0.753323
H	-6.346724	-0.651728	0.818117
H	-6.293795	0.046283	-0.791049
H	-4.148190	1.179897	-0.239779
H	-4.221660	0.548388	1.397658
H	-4.207885	-1.799344	0.447991
H	-3.997073	-1.121449	-1.162757
H	4.625578	3.689753	-0.119063
H	3.196523	-2.632456	0.581254
H	-1.870060	1.112714	-0.415963
H	-1.783704	-0.030733	-1.757216
H	-2.086208	-0.775786	1.187043
H	0.388480	2.047576	-0.652224
H	5.988598	0.470429	0.623333

ωB97X Energy = -1148.25148924 a.u.

(*R*)-**3**, Conf P

C	6.585763	1.153744	1.288387
C	6.252435	-0.267784	1.728781
C	4.909996	-0.774974	1.204592
C	4.847032	-0.876237	-0.317543
C	3.603886	-1.591290	-0.842203
O	-6.042466	-1.238903	0.825837
O	-1.043568	2.636248	-0.147141
O	1.327723	2.315210	-1.460333
O	-3.517577	2.685516	0.519816
C	1.077444	-1.631593	-1.044803
C	2.292179	-0.921666	-0.486057
O	2.337021	0.422353	-1.028374
C	1.233973	1.184504	-1.054878
C	-1.364901	-1.476447	-0.433948
C	-0.152875	-0.844581	-0.702283
C	-0.049180	0.540737	-0.636045
C	-1.164970	1.318047	-0.250245
C	-3.571223	1.456749	0.431177
C	-4.821354	0.764001	0.720355
C	-4.920594	-0.564504	0.598839
C	-3.736735	-1.409857	0.232125
C	-2.459680	-0.720526	-0.081039
C	-2.377907	0.672009	0.047194
O	-3.880997	-2.612483	0.197964
H	5.789475	1.846433	1.575915
H	6.710287	1.222948	0.205445
H	7.513003	1.501502	1.748505
H	7.045766	-0.948596	1.400639
H	6.242746	-0.311826	2.821922
H	4.707938	-1.762445	1.635127
H	4.117582	-0.110428	1.568599
H	4.901723	0.120069	-0.763827
H	5.727093	-1.422879	-0.672619
H	3.658177	-1.681870	-1.931333
H	3.565029	-2.608211	-0.438142
H	-5.852280	-2.183389	0.697166
H	-1.921838	2.986888	0.133309
H	1.009614	-2.637776	-0.629631
H	1.174960	-1.725985	-2.131646
H	2.191396	-0.829094	0.600266
H	-1.449545	-2.554364	-0.494268
H	-5.671950	1.373492	0.997998

ωB97X Energy = -1148.25136154 a.u.

(*R*)-**3**, Conf Q

C	8.134196	-0.784140	0.672073
C	6.645953	-0.497474	0.828910
C	5.785227	-1.404352	-0.045592
C	4.279855	-1.231135	0.149899
C	3.762661	0.130398	-0.307177
O	-5.694072	-2.418380	0.220757
O	-1.936086	2.759688	0.367120
O	0.525169	3.299978	-0.691735
O	-4.366607	2.035743	0.739082
C	1.366528	-0.582866	-0.833317
C	2.283579	0.324146	-0.040035
O	1.988553	1.701657	-0.386584
C	0.721244	2.135752	-0.451359
C	-1.060928	-1.185120	-0.510851
C	-0.059474	-0.217417	-0.543467
C	-0.357337	1.114817	-0.280168
C	-1.673185	1.492137	0.071787
C	-4.064228	0.868677	0.480234
C	-5.084493	-0.171956	0.529654
C	-4.795240	-1.440167	0.217079
C	-3.398211	-1.865195	-0.126714
C	-2.348399	-0.817476	-0.192165
C	-2.673201	0.505862	0.133222
O	-3.194564	-3.039625	-0.344740
H	8.452435	-0.638667	-0.363821
H	8.364613	-1.816828	0.947511
H	8.737639	-0.127303	1.301866
H	6.353310	-0.629737	1.876584
H	6.454606	0.551940	0.585359
H	6.036238	-1.236806	-1.099953
H	6.046832	-2.445929	0.169018
H	3.764395	-2.023195	-0.401041
H	4.034239	-1.377482	1.208512
H	4.294548	0.930049	0.213926
H	3.949307	0.263434	-1.378180
H	-5.236068	-3.242956	-0.013970
H	-2.896246	2.812567	0.586737

H	1.542465	-1.625138	-0.567086
H	1.577951	-0.471798	-1.902459
H	2.080217	0.206954	1.030420
H	-0.832599	-2.221596	-0.725937
H	-6.090314	0.133049	0.789228

ωB97X Energy = -1148.25124423 a.u.

(*R*)-**3**, Conf R

C	6.334391	-1.795222	-1.226412
C	6.662479	-0.732255	-0.183968
C	5.889785	-0.896064	1.125356
C	4.367976	-0.862303	0.984300
C	3.852615	0.437118	0.371286
O	-5.473082	-2.588335	0.040383
O	-2.003649	2.788766	0.112416
O	0.542684	3.369441	-0.685477
O	-4.426392	1.964656	0.254931
C	1.572405	-0.457476	-0.338969
C	2.346056	0.573473	0.455643
O	2.030367	1.892995	-0.057089
C	0.761522	2.245169	-0.311364
C	-0.845517	-1.164710	-0.241955
C	0.106726	-0.148036	-0.256162
C	-0.281843	1.183980	-0.167931
C	-1.647502	1.516248	-0.017020
C	-4.040485	0.797661	0.156838
C	-5.010591	-0.290736	0.184886
C	-4.626364	-1.564577	0.046204
C	-3.178539	-1.939383	-0.070475
C	-2.178150	-0.843392	-0.119537
C	-2.601294	0.484210	0.024335
O	-2.894763	-3.115881	-0.130438
H	6.969562	-1.691083	-2.108361
H	5.295972	-1.726901	-1.560979
H	6.485965	-2.799795	-0.821073
H	7.732339	-0.766389	0.041679
H	6.476287	0.261670	-0.603667
H	6.174829	-1.847207	1.587654
H	6.197884	-0.108834	1.821735
H	4.037807	-1.715655	0.384077
H	3.921538	-0.994519	1.975649
H	4.297364	1.289963	0.893254
H	4.148257	0.514767	-0.679636
H	-4.951669	-3.404248	-0.044218
H	-2.985305	2.806708	0.206983
H	1.763031	-1.456279	0.054003
H	1.906230	-0.441121	-1.382383
H	2.025960	0.546294	1.503241
H	-0.545038	-2.202242	-0.319019
H	-6.054363	-0.020499	0.282912

ωB97X Energy = -1148.25117505 a.u.

(*R*)-**3**, Conf S

C	8.025986	-1.360266	0.177191
C	6.502676	-1.339792	0.197797
C	5.927094	-0.128591	-0.529955
C	4.402983	-0.119552	-0.627174
C	3.717584	-0.050773	0.735135
O	-5.783374	-2.338988	-0.070979
O	-1.910896	2.751631	0.151283
O	0.699918	3.126245	-0.556693
O	-4.394622	2.118757	0.194051
C	1.418664	-0.758496	-0.146466
C	2.224715	0.213358	0.690578
O	2.041768	1.550466	0.155983
C	0.817028	1.994077	-0.160968
C	-1.049832	-1.282633	-0.138591
C	-0.022147	-0.342474	-0.118263
C	-0.309895	1.016479	-0.052696
C	-1.650308	1.454260	0.041653
C	-4.097017	0.924852	0.113827
C	-5.149815	-0.084075	0.094629
C	-4.860230	-1.384710	-0.024464
C	-3.442003	-1.872168	-0.067948
C	-2.357534	-0.858065	-0.073308
C	-2.682257	0.499350	0.047825
O	-3.248484	-3.067600	-0.110043
H	8.423298	-2.228744	0.706568
H	8.434196	-0.463959	0.652165
H	8.402635	-1.391938	-0.848742
H	6.114676	-2.253834	-0.266129
H	6.156260	-1.350258	1.235988
H	6.263498	0.787120	-0.028684
H	6.344701	-0.095119	-1.541997
H	4.088634	0.738396	-1.228695
H	4.076789	-1.016925	-1.164299
H	3.860603	-0.984622	1.286149
H	4.176123	0.739669	1.338038
H	-5.324540	-3.193888	-0.127856
H	-2.891130	2.846497	0.204504
H	1.531197	-1.770417	0.245117
H	1.782947	-0.758196	-1.178276
H	1.831942	0.220872	1.712349
H	-0.827766	-2.340742	-0.200226
H	-6.172720	0.267334	0.142392

ωB97X Energy = -1148.25116656 a.u.

(*R*)-**3**, Conf T

C	5.358734	-1.761524	2.684656
C	5.304101	-0.978819	1.378706
C	4.551148	-1.727549	0.283518
C	4.570272	-1.035049	-1.079805
C	3.998422	0.383513	-1.101371
O	-5.318573	-2.425274	0.142126
O	-1.543134	2.696799	0.826849
O	0.752216	3.490356	-0.431945
O	-3.890501	1.844473	1.405618
C	1.554738	-0.275692	-1.428263
C	2.570259	0.485452	-0.602626

O	2.238137	1.897070	-0.636629
C	0.975095	2.306411	-0.448800
C	-0.816143	-0.975931	-0.928273
C	0.179503	-0.001993	-0.894089
C	-0.075984	1.248548	-0.342739
C	-1.333382	1.523370	0.241780
C	-3.650682	0.779132	0.832849
C	-4.677968	-0.253458	0.763996
C	-4.427593	-1.441798	0.202792
C	-3.110074	-1.739958	-0.449715
C	-2.050204	-0.702528	-0.383887
C	-2.334694	0.537570	0.202611
O	-2.969346	-2.815225	-0.990337
H	5.895315	-1.211008	3.459977
H	5.863532	-2.721049	2.544330
H	4.351867	-1.968064	3.057717
H	4.832589	-0.007495	1.560732
H	6.321423	-0.765149	1.032094
H	4.993389	-2.722678	0.168368
H	3.518121	-1.899210	0.610044
H	5.604186	-0.980055	-1.433724
H	4.038461	-1.656938	-1.806415
H	4.613353	1.047497	-0.488459
H	4.032525	0.775575	-2.122012
H	-4.908376	-3.168437	-0.331367
H	-2.458106	2.677472	1.194690
H	1.761877	-1.345635	-1.388723
H	1.625112	0.037167	-2.475759
H	2.504770	0.166246	0.443146
H	-0.621695	-1.947897	-1.364437
H	-5.632034	-0.034114	1.226303

ωB97X Energy = -1148.25116387 a.u.

(*R*)-**3**, Conf U

C	6.372774	-2.066974	1.084190
C	5.138287	-1.358098	1.630167
C	5.026456	0.103180	1.193894
C	4.979884	0.328123	-0.318936
C	3.863114	-0.415527	-1.050415
O	-5.703986	-2.052792	0.306632
O	-1.409350	2.686079	0.543834
O	0.969494	3.096529	-0.726380
O	-3.855528	2.167792	1.107496
C	1.376274	-0.814185	-1.329043
C	2.464526	-0.081457	-0.573854
O	2.281220	1.345326	-0.755403
C	1.067443	1.900068	-0.623806
C	-1.053733	-1.213487	-0.785187
C	0.036618	-0.348299	-0.840938
C	-0.088228	0.972423	-0.423793
C	-1.314016	1.436154	0.106104
C	-3.707901	1.010794	0.707338
C	-4.827924	0.078347	0.759127
C	-4.707968	-1.173671	0.303343
C	-3.401848	-1.698308	-0.215727
C	-2.255881	-0.757775	-0.293939
C	-2.403149	0.550511	0.185031
O	-3.346547	-2.859569	-0.557347
H	6.457231	-3.078109	1.487447
H	7.283636	-1.522989	1.350147
H	6.343612	-2.149352	-0.005197
H	4.239710	-1.908927	1.331107
H	5.157283	-1.391127	2.723387
H	4.140890	0.548620	1.659544
H	5.882881	0.656604	1.593648
H	4.881134	1.399173	-0.513108
H	5.932200	0.026216	-0.763980
H	3.921019	-0.200831	-2.121664
H	3.991058	-1.496974	-0.940468
H	-5.361914	-2.890454	-0.048291
H	-2.331503	2.808621	0.871828
H	1.479380	-1.889512	-1.179049
H	1.476445	-0.616877	-2.401729
H	2.363850	-0.286230	0.497571
H	-0.961119	-2.239263	-1.119838
H	-5.766570	0.453579	1.146657

ωB97X Energy = -1148.25115694 a.u.

(*R*)-**3**, Conf V

C	-6.866349	-0.037090	1.749389
C	-6.798421	-1.177297	0.738948
C	-5.931139	-0.869399	-0.479977
C	-4.457422	-0.645982	-0.153297
C	-3.621044	-0.453705	-1.413800
O	5.894763	-2.001703	0.359482
O	1.637361	2.759866	-0.040735
O	-1.059378	2.877195	0.374834
O	4.157593	2.335881	0.163093
C	-1.383072	-1.021975	-0.301865
C	-2.171359	-0.073537	-1.181381
O	-2.167821	1.241697	-0.567300
C	-1.030806	1.764356	-0.086334
C	1.108192	-1.341569	-0.052489
C	0.010249	-0.489263	-0.143621
C	0.182700	0.890293	-0.108983
C	1.481414	1.441300	-0.027011
C	3.958928	1.118961	0.150119
C	5.088281	0.200382	0.235254
C	4.902662	-1.124283	0.257129
C	3.536269	-1.727640	0.118577
C	2.372732	-0.808054	0.052311
C	2.587950	0.576194	0.036529
O	3.445714	-2.935391	0.079633
H	-7.212484	0.884381	1.272367
H	-5.890577	0.167050	2.195796
H	-7.556534	-0.271554	2.562460
H	-6.420181	-2.080756	1.230147
H	-7.810563	-1.415148	0.398365
H	-6.013501	-1.695632	-1.194798
H	-6.325049	0.018762	-0.988795

H	-4.344680	0.231192	0.490283
H	-4.088263	-1.505946	0.416747
H	-3.625848	-1.371331	-2.010603
H	-4.070635	0.324772	-2.037797
H	5.507151	-2.892456	0.324818
H	2.605619	2.937360	0.020395
H	-1.363223	-2.016210	-0.749923
H	-1.859047	-1.108339	0.679581
H	-1.667368	0.022927	-2.148422
H	0.975202	-2.416163	-0.069227
H	6.075546	0.636581	0.319543

ωB97X Energy = -1148.25114263 a.u.

(*R*)-**3**, Conf W

C	7.498719	-0.490557	1.282989
C	6.176760	0.146095	1.698959
C	4.943595	-0.547716	1.120994
C	4.840917	-0.453267	-0.398898
C	3.633743	-1.179054	-0.989786
O	-6.011895	-1.559779	0.706882
O	-1.248485	2.671869	0.109216
O	1.110279	2.611139	-1.270727
O	-3.717698	2.514673	0.778771
C	1.113029	-1.360887	-1.179348
C	2.287407	-0.626492	-0.567891
O	2.243785	0.759340	-0.993378
C	1.094603	1.448913	-0.953162
C	-1.329050	-1.410206	-0.546710
C	-0.160002	-0.684441	-0.764273
C	-0.140074	0.693401	-0.578100
C	-1.295089	1.364141	-0.116334
C	-3.700985	1.298648	0.574987
C	-4.909786	0.511825	0.789278
C	-4.930418	-0.804211	0.550198
C	-3.696816	-1.544517	0.124801
C	-2.464501	-0.755525	-0.126437
C	-2.465085	0.622848	0.124746
O	-3.767721	-2.746984	-0.006883
H	7.669473	-0.409341	0.207316
H	7.517015	-1.552739	1.543563
H	8.341073	-0.009815	1.784750
H	6.104334	0.135929	2.790522
H	6.167016	1.200524	1.401421
H	4.950454	-1.604018	1.418013
H	4.052982	-0.104657	1.578079
H	4.815942	0.599723	-0.697265
H	5.738097	-0.881533	-0.853745
H	3.683884	-1.154024	-2.082559
H	3.657082	-2.233006	-0.693988
H	-5.765816	-2.476994	0.499940
H	-2.142959	2.943433	0.423890
H	1.112115	-2.400766	-0.850803
H	1.205007	-1.356982	-2.270721
H	2.190994	-0.633475	0.522578
H	-1.349066	-2.481949	-0.700928
H	-5.795560	1.043629	1.112815

ωB97X Energy = -1148.25110950 a.u.

(*R*)-**3**, Conf X

C	6.631093	-1.836618	-1.535712
C	6.888950	-0.570874	-0.725137
C	5.982915	-0.422944	0.495642
C	4.502526	-0.275412	0.156450
C	3.654748	-0.047643	1.403258
O	-5.773226	-2.230486	-0.149682
O	-1.771221	2.763345	-0.183335
O	0.913586	2.990843	-0.623411
O	-4.265155	2.192240	-0.335285
C	1.447422	-0.820805	0.352287
C	2.186167	0.234088	1.150004
O	2.110506	1.495422	0.435931
C	0.945906	1.917528	-0.076643
C	-1.024228	-1.290495	0.149706
C	0.027043	-0.376760	0.163839
C	-0.218915	0.984608	0.022175
C	-1.545366	1.457922	-0.093596
C	-4.002633	0.992608	-0.223246
C	-5.082491	0.012679	-0.218774
C	-4.827433	-1.297696	-0.133002
C	-3.429708	-1.816223	0.035356
C	-2.315962	-0.834577	0.014202
C	-2.604348	0.533188	-0.079497
O	-3.274831	-3.010584	0.169017
H	7.353747	-1.938006	-2.348054
H	5.634280	-1.836104	-1.982373
H	6.711916	-2.725828	-0.903774
H	7.930705	-0.566133	-0.390827
H	6.769669	0.307676	-1.368845
H	6.118346	-1.290275	1.153534
H	6.301037	0.452905	1.071442
H	4.371904	0.564914	-0.533909
H	4.156286	-1.171185	-0.367506
H	3.709717	-0.923126	2.058219
H	4.058681	0.793654	1.974608
H	-5.339722	-3.094350	-0.047062
H	-2.747719	2.883968	-0.252698
H	1.482011	-1.776681	0.876640
H	1.923635	-0.958019	-0.623155
H	1.679677	0.378242	2.109796
H	-0.833464	-2.351660	0.250777
H	-6.092106	0.388331	-0.326571

ωB97X Energy = -1148.25106088 a.u.

(*R*)-**3**, Conf Y

C	-6.418544	-2.112572	2.279418
C	-5.944506	-1.053288	1.292575
C	-4.577121	-1.370298	0.698499
C	-4.094382	-0.315257	-0.289381
C	-2.725434	-0.643834	-0.873110

O	5.551415	-1.999268	0.391105
O	0.865970	2.234186	1.430409
O	-1.148827	3.207031	-0.115663
O	3.110413	1.452997	2.389729
C	-0.901323	0.050784	-2.523191
C	-2.245961	0.372971	-1.897454
O	-2.186718	1.701718	-1.314356
C	-1.120959	2.107490	-0.608454
C	1.306907	-0.633148	-1.516706
C	0.188040	0.195283	-1.502723
C	0.050124	1.180737	-0.531186
C	1.028081	1.321919	0.479174
C	3.202102	0.605779	1.498507
C	4.360011	-0.279897	1.458424
C	4.504169	-1.187367	0.486182
C	3.450015	-1.387956	-0.562014
C	2.274807	-0.480920	-0.549445
C	2.145179	0.466897	0.473187
O	3.612005	-2.271621	-1.375146
H	-6.495569	-3.090322	1.796135
H	-7.398900	-1.866222	2.692425
H	-5.719039	-2.209661	3.114115
H	-6.673985	-0.952832	0.481560
H	-5.902290	-0.078599	1.790619
H	-3.845534	-1.469841	1.509457
H	-4.617407	-2.345919	0.199099
H	-4.821598	-0.219943	-1.104282
H	-4.056067	0.658564	0.208919
H	-1.989733	-0.738936	-0.067244
H	-2.764933	-1.614722	-1.377947
H	5.409167	-2.578175	-0.376640
H	1.647143	2.169769	2.029188
H	-0.713826	0.736344	-3.354863
H	-0.912123	-0.960630	-2.930356
H	-2.998781	0.476794	-2.679329
H	1.419322	-1.395189	-2.277886
H	5.112854	-0.144746	2.224586

ωB97X Energy = -1148.25104539 a.u.

(*R*)-**3**, Conf Z

C	-5.284443	3.168719	0.334770
C	-5.635930	1.970672	1.210492
C	-4.694899	0.781175	1.029181
C	-4.729878	0.182443	-0.375386
C	-3.976090	-1.139876	-0.508238
O	4.946742	2.930281	-0.069241
O	2.026877	-2.715757	0.668350
O	-0.273490	-3.766339	-0.345402
O	4.299552	-1.577330	0.994274
C	-1.699632	-0.109170	-1.022883
C	-2.503213	-1.053964	-0.155242
O	-1.979975	-2.397544	-0.313091
C	-0.659226	-2.627062	-0.275150
C	0.588286	0.904063	-0.718076
C	-0.249486	-0.207007	-0.650707
C	0.238708	-1.433208	-0.212962
C	1.582156	-1.551396	0.211206
C	3.831211	-0.513603	0.582342
C	4.677806	0.671638	0.513532
C	4.208925	1.830735	0.037864
C	2.775569	1.986766	-0.376269
C	1.902881	0.786591	-0.328120
C	2.414723	-0.419329	0.167954
O	2.400678	3.079769	-0.740997
H	-5.930755	4.020786	0.554938
H	-5.394182	2.939709	-0.727639
H	-4.249802	3.482165	0.502457
H	-5.618169	2.276023	2.260803
H	-6.662502	1.650827	1.000301
H	-3.675364	1.100054	1.278032
H	-4.958165	0.000795	1.751924
H	-5.772485	0.003033	-0.656763
H	-4.343739	0.902930	-1.102869
H	-4.432158	-1.889886	0.144546
H	-4.057953	-1.513259	-1.533246
H	4.376595	3.638604	-0.412582
H	2.974044	-2.588121	0.912439
H	-2.048710	0.915239	-0.887320
H	-1.839473	-0.369439	-2.077795
H	-2.375793	-0.782831	0.897687
H	0.212048	1.857530	-1.067486
H	5.708675	0.562067	0.825592

ωB97X Energy = -1148.25092674 a.u.

(*R*)-**3**, Conf AA

C	6.642513	-0.548170	1.896021
C	6.772779	-0.565613	0.377227
C	5.827654	-1.552222	-0.309594
C	4.339680	-1.336181	-0.029844
C	3.834202	0.032233	-0.477077
O	-5.653598	-2.334273	0.288178
O	-1.791960	2.768881	0.367308
O	0.650976	3.267726	-0.752072
O	-4.225667	2.089570	0.800178
C	1.410865	-0.629573	-0.942476
C	2.367110	0.253231	-0.168413
O	2.089992	1.638847	-0.497208
C	0.830223	2.098150	-0.524907
C	-1.018102	-1.186601	-0.557129
C	0.000848	-0.238416	-0.611123
C	-0.263529	1.097559	-0.331534
C	-1.561872	1.498268	0.057775
C	-3.953117	0.918595	0.526393
C	-4.991964	-0.102397	0.596389
C	-4.736069	-1.373794	0.268120
C	-3.356670	-1.823808	-0.112996
C	-2.289004	-0.796015	-0.201421
C	-2.579041	0.531224	0.140194
O	-3.181110	-3.000987	-0.340365
H	7.396679	0.098853	2.348480

H	6.770314	-1.552386	2.310467
H	5.663192	-0.181596	2.213966
H	6.611766	0.443320	-0.015652
H	7.798990	-0.830540	0.106327
H	5.993956	-1.511046	-1.391467
H	6.091985	-2.567807	0.003931
H	3.780407	-2.123914	-0.542839
H	4.138813	-1.465174	1.039274
H	4.395165	0.822803	0.028219
H	3.994376	0.162046	-1.552855
H	-5.217872	-3.165933	0.036531
H	-2.744616	2.839027	0.612902
H	1.573414	-1.677064	-0.688630
H	1.595144	-0.514421	-2.016167
H	2.190828	0.132272	0.906442
H	-0.816045	-2.225907	-0.784366
H	-5.984584	0.220525	0.883667

ωB97X Energy = -1148.25085697 a.u.

(*R*)-**3**, Conf AB

C	8.172754	-0.403444	0.171151
C	6.693796	-0.037241	0.154169
C	5.827404	-1.148744	-0.431223
C	4.346887	-0.795143	-0.567194
C	3.645975	-0.618371	0.776519
O	-6.035047	-1.907430	0.062460
O	-1.643379	2.747674	0.070166
O	0.995375	2.821337	-0.643039
O	-4.176433	2.381175	0.206997
C	1.291209	-1.104237	-0.122504
C	2.189372	-0.201536	0.698003
O	2.156541	1.131605	0.124315
C	0.988193	1.694673	-0.215734
C	-1.219640	-1.361811	-0.113782
C	-0.097233	-0.536731	-0.115203
C	-0.237742	0.846598	-0.092517
C	-1.523481	1.427993	-0.014785
C	-4.011014	1.163365	0.105827
C	-5.166925	0.274467	0.093071
C	-5.017091	-1.054037	0.045474
C	-3.662561	-1.687377	-0.076363
C	-2.474044	-0.798057	-0.058477
C	-2.652863	0.590727	-0.013781
O	-3.601862	-2.893704	-0.173577
H	8.780235	0.399676	0.593360
H	8.537649	-0.603295	-0.839959
H	8.345245	-1.302490	0.769156
H	6.370016	0.195822	1.173040
H	6.546472	0.876218	-0.432751
H	6.217086	-1.408040	-1.421414
H	5.931294	-2.051112	0.183857
H	4.245549	0.122778	-1.155775
H	3.851894	-1.588109	-1.135446
H	3.686348	-1.555514	1.341496
H	4.163735	0.131438	1.380073
H	-5.669979	-2.805601	-0.007390
H	-2.606484	2.946076	0.148068
H	1.293871	-2.111946	0.294933
H	1.657798	-1.168112	-1.151326
H	1.786631	-0.123327	1.713016
H	-1.112232	-2.439121	-0.138149
H	-6.144659	0.732841	0.170731

ωB97X Energy = -1148.25084406 a.u.

(*R*)-**3**, Conf AC

C	5.790093	-3.145538	-1.657570
C	5.128204	-2.456707	-0.470983
C	4.377066	-1.191587	-0.868630
C	3.718344	-0.490120	0.313450
C	2.947973	0.757196	-0.106930
O	-4.525368	-3.113617	0.207754
O	-1.707938	2.398123	-1.418487
O	0.197853	3.952132	-0.237509
O	-3.720300	0.924159	-2.008545
C	1.417424	0.884434	1.958696
C	2.440522	1.573172	1.073606
O	1.875370	2.822924	0.596927
C	0.605221	2.893750	0.170254
C	-0.639623	-0.456654	1.376644
C	0.145548	0.677906	1.192833
C	-0.228660	1.656937	0.278945
C	-1.391537	1.487817	-0.504973
C	-3.391103	0.116429	-1.137054
C	-4.203106	-1.073130	-0.909125
C	-3.838558	-1.998371	-0.014357
C	-2.625168	-1.819263	0.849416
C	-1.784797	-0.615559	0.628462
C	-2.185827	0.343189	-0.310043
O	-2.382538	-2.668635	1.678828
H	6.321625	-4.048895	-1.351498
H	6.510832	-2.481625	-2.142432
H	5.047377	-3.432278	-2.406933
H	4.433391	-3.149305	0.016287
H	5.886537	-2.203827	0.277969
H	5.069572	-0.498645	-1.360812
H	3.612137	-1.443472	-1.612871
H	3.045999	-1.195098	0.814572
H	4.486357	-0.216732	1.046734
H	3.600644	1.408640	-0.695352
H	2.112523	0.476136	-0.756535
H	-4.073446	-3.604675	0.914501
H	-2.524597	2.081307	-1.871864
H	1.220248	1.512056	2.832688
H	1.806398	-0.065246	2.325255
H	3.289181	1.892106	1.679153
H	-0.349926	-1.217819	2.090372
H	-5.083586	-1.196729	-1.526822

ωB97X Energy = -1148.25083253 a.u.

(*R*)-**3**, Conf AD

C	6.268566	-2.465630	-0.160088
C	6.623037	-1.082100	0.373407
C	6.032545	0.064048	-0.449134
C	4.508081	0.057444	-0.565759
C	3.808491	0.127184	0.788404
O	-5.621848	-2.417555	-0.067364
O	-1.886466	2.775826	0.105446
O	0.722642	3.209393	-0.570689
O	-4.352759	2.079002	0.134167
C	1.536276	-0.651036	-0.101824
C	2.309903	0.353042	0.727499
O	2.097560	1.677081	0.172143
C	0.864625	2.085150	-0.160909
C	-0.917416	-1.238593	-0.107555
C	0.085200	-0.271737	-0.092476
C	-0.237985	1.080303	-0.051027
C	-1.590413	1.484228	0.021443
C	-4.023275	0.892098	0.077740
C	-5.049167	-0.144262	0.066372
C	-4.724581	-1.438628	-0.028127
C	-3.293914	-1.889349	-0.050176
C	-2.236295	-0.847408	-0.061891
C	-2.597259	0.502843	0.033228
O	-3.069017	-3.079882	-0.068823
H	6.780047	-3.248314	0.403820
H	6.558412	-2.565327	-1.210075
H	5.195310	-2.662233	-0.093722
H	6.298577	-0.997890	1.415759
H	7.711103	-0.969739	0.389056
H	6.349469	1.016527	-0.010599
H	6.459086	0.031305	-1.457483
H	4.198574	0.911694	-1.174741
H	4.187643	-0.839084	-1.105577
H	3.969208	-0.797279	1.351483
H	4.242457	0.934277	1.387290
H	-5.139721	-3.260634	-0.105987
H	-2.869416	2.845513	0.147912
H	1.671148	-1.653984	0.305486
H	1.909220	-0.655985	-1.130464
H	1.908044	0.364941	1.745688
H	-0.667312	-2.291374	-0.150009
H	-6.081261	0.181070	0.098341

ωB97X Energy = -1148.25074579 a.u.
